# Tuning Emission Lifetimes of Ir(C^N)_2_(acac) Complexes with Oligo(phenyleneethynylene) Groups

**DOI:** 10.1021/acs.inorgchem.2c03934

**Published:** 2023-01-27

**Authors:** Ross Davidson, Yu-Ting Hsu, Mark A. Fox, Juan A. Aguilar, Dmitry Yufit, Andrew Beeby

**Affiliations:** Department of Chemistry, University of Durham, South Road, Durham DH1 3LE, England, U.K.

## Abstract

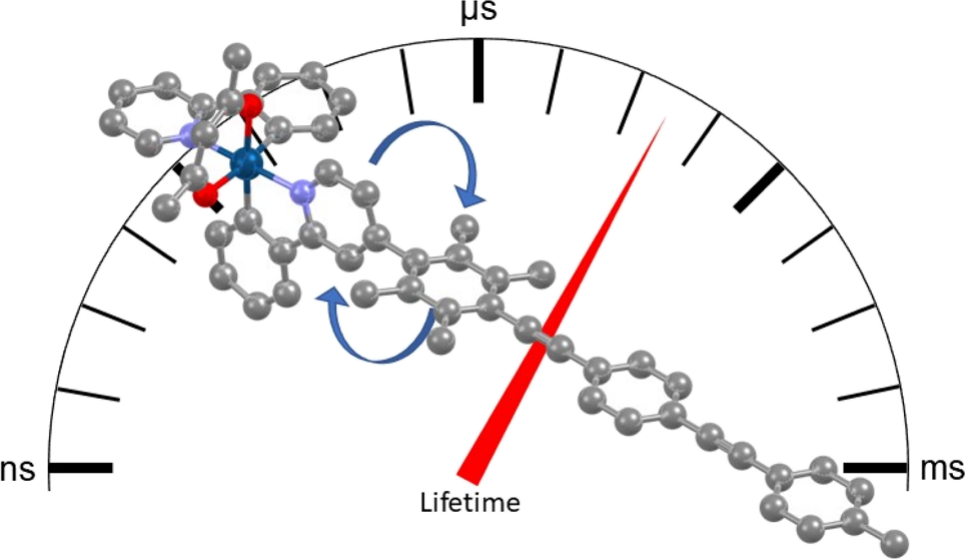

Emissive compounds with long emission lifetimes (μs to ms) in the visible region are of interest for a range of applications, from oxygen sensing to cellular imaging. The emission behavior of Ir(ppy)_2_(acac) complexes (where ppy is the 2-phenylpyridyl chelate and acac is the acetylacetonate chelate) with an oligo(*para*-phenyleneethynylene) (OPE3) motif containing three para-rings and two ethynyl bridges attached to acac or ppy is examined here due to the accessibility of the long-lived OPE3 triplet states. Nine Ir(ppy)_2_(acac) complexes with OPE3 units are synthesized where the OPE3 motif is at the acac moiety (aOPE3), incorporated in the ppy chelate (pOPE3) or attached to ppy via a durylene link (dOPE3). The aOPE3 and dOPE3 complexes contain OPE3 units that are decoupled from the Ir(ppy)_2_(acac) core by adopting perpendicular ring–ring orientations, whereas the pOPE3 complexes have OPE3 integrated into the ppy ligand to maximize electronic coupling with the Ir(ppy)_2_(acac) core. While the conjugated pOPE3 complexes show emission lifetimes of 0.69–32.8 μs similar to the lifetimes of 1.00–23.1 μs for the non-OPE3 Ir(ppy)_2_(acac) complexes synthesized here, the decoupled aOPE3 and dOPE3 complexes reveal long emission lifetimes of 50–625 μs. The long lifetimes found in aOPE3 and dOPE3 complexes are due to intramolecular reversible electronic energy transfer (REET) where the long-lived triplet-state metal to ligand charge transfer (^3^MLCT) states exchange via REET with the even longer-lived triplet-state localized OPE3 states. The proposed REET process is supported by changes observed in excitation wavelength-dependent and time-dependent emission spectra from aOPE3 and dOPE3 complexes, whereas emission spectra from pOPE3 complexes remain independent of the excitation wavelength and time due to the well-established ^3^MLCT states of many Ir(ppy)_2_(acac) complexes. The long lifetimes, visible emission maxima (524–526 nm), and photoluminescent quantum yields of 0.44–0.60 for the dOPE3 complexes indicate the possibility of utilizing such compounds in oxygen-sensing and cellular imaging applications.

## Abstract 2

A combined synthetic, computational, and photophysical study that demonstrates that by modifying the conjugation between Ir(ppy)_2_(acac) and oligo(*para*-phenyleneethynylene) (OPE), it is possible to vary the emission lifetime from 0.69 to 625 μs by exploiting the long-lived triplet state of OPE3.

## Introduction

Compounds capable of long emission lifetimes (μs to ms) in the visible region are of interest because of their use as emitters in cellular imaging,^1,2^ oxygen sensing,^3^ photon up-conversion,^4^ and photocatalysis.^5,6^ Some of the longest-emission lifetime materials available that have been produced are based on either organic room-temperature phosphorescent materials or lanthanide complexes. However, both typically have poor photoluminescent quantum yields (PLQYs, Φ) due to the spin-forbidden nature of the singlet–triplet transition.

Coordination compounds with strong spin orbit coupling are able to overcome this limitation and give typically high Φ_P_, one of the most famous examples being acetylacetonatobis(2-phenylpyridine)iridium [Ir(C^N)_2_(acac)].^7^ This and related complexes have become popular due to the predictable way in which the electronic behavior of the system can be tuned. Adding electron-withdrawing groups to the phenyl ring induces a stabilization of the highest occupied molecular orbital (HOMO) and a concomitant blue shifting; conversely, adding electron-withdrawing groups to the pyridine ring lowers the energy of the lowest unoccupied molecular orbital (LUMO) and brings about a red shift, while the acetylacetonate (acac) remains a passive ancillary ligand.^8−17^

Despite the vast range of iridium complexes that have been made, few examples have had emission lifetimes that exceeded 10 μs at room temperature in solution. Most of these long-lived examples occurred because of a predominately ^3^LC emission that enhanced organic phosphorescence due to iridium facilitating spin–orbit coupling. An example of this is the formation of an Ir(C^N)(acac) complex, where 2-phenylpyridyl was replaced by 2-(pyren-1′-yl)pyridyl, resulting in an emission of 37 μs.^18^

Few long-lived examples result from reversible electronic energy transfer (REET), where a triplet sensitizer is tethered (but only weakly coupled) to a phosphorescent emitter that has a triplet state (T_*n*_) thermally comparable to that of the sensitizer. This shuttling of the energy between the emitter and sensitizer results in emission lifetimes increasing by 1–2 orders of magnitude.^19,20^ The triplet sensitizers commonly used are naphthalenediimides (attached via an alkyne) for both Ir(III) and Pt(II) complexes,^21,22^ pyrenes (attached via an ethane) for Ir(III) complexes,^23^ and fluorenes (attached directly) for Ir(III) complexes.^24−26^

The work of Medina-Rodríguez and co-workers involved one–two pyrenes tethered to a 2,2′-bipyridine via an ethane linker.^23^ This ligand was, in turn, coordinated with an Ir(C^N)_2_ complex to give Ir(C^N)_2_(bpy-R). The use of an ethane linker led to the pyrene being electronically decoupled from the metal complex and having only a 700 cm^–1^ difference between the complex T_1_ and that of the parent pyrene. REET was observed, resulting in room-temperature emission lifetimes of 225–480 μs, with the bis(pyrene) system having twice the emission lifetime of the single pyrene system. This was due to REET being an entropically driven phenomenon, and therefore, by increasing the “reservoir”, the effect was increased.

**Figure 1:**
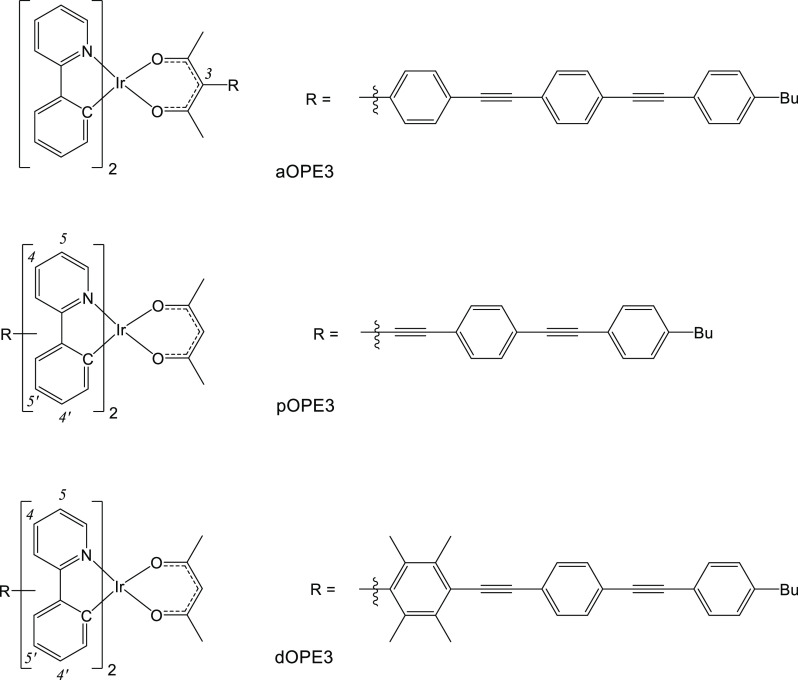
Types of iridium-OPE3 complexes targeted in this study. The R group in pOPE3 and dOPE3 is substituted at the 4, 5, 4′, or 5′ position of the ppy chelate.

In this study, we examine various approaches for controlling REET to tune the emission lifetime, independently of the emission energy and PLQY (Φ). The simple rod molecule bis(phenylethynyl)benzene (BPEB) has T_1_ at 2.53 eV close to that of Ir(ppy)_2_(acac) at 2.55 eV, so a BPEB-type framework could be used as a triplet sensitizer.^27^ Therefore, an oligo(*para*-phenyleneethynylene) motif (OPE3) as a generic model of BPEB is employed here as the triplet sensitizer for three different architectures: (i) attaching OPE3 to the ancillary acac ligand (acac is typically passive in the photophysics of the system) to electronically decouple the sensitizer from the emitter, (ii) integrating OPE3 with the ppy ligand at different positions (the HOMO is most affected by modifications made to the phenylene ring, whereas the LUMO is governed by pyridyl modifications), and (iii) attaching OPE3 to ppy via duryl groups to electronically decouple the sensitizer from the emitter. These three iridium-OPE3 complex types are abbreviated here as aOPE3, pOPE3, and dOPE3, respectively, and depicted in Figure 1.

## Results and Discussion

### Synthesis

To assess the impact that an OPE3-substituted acac chelate has on the photophysics of an Ir(ppy)_2_(acac) complex (aOPE3, Figure 1), acac chelates were targeted with aryl substitutions at the C3 position. This has the benefit of maintaining a symmetrical ligand, and the aryl group is twisted out of the plane of the acac moiety, resulting in the substituents being electronically decoupled. The modular construction of this complex motif allowed for both the acac and ppy ligands to be varied systematically. Initially, the known complex (ppy)_2_Ir(acac-C_6_H_4_I)^28,29^ was used as a precursor to aOPE3, but it was found to be unstable during Sonogashira couplings, and the products produced were shown to be unstable when using silica chromatography for purification. A different approach was then adopted by synthesizing the ligands prior to coordination, followed by recrystallization to achieve pure compounds.

**Figure 2:**
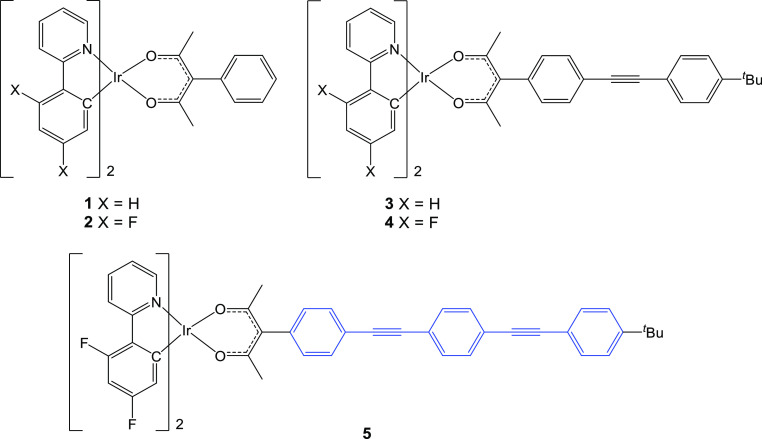
Substituted acac complexes **1–5**. In the aOPE3 complex, blue highlights the OPE3 component.

The substituted acac complexes **1–5** shown in Figure 2 were produced by first coupling 4-hydroxy-3-(4′-iodophenyl)pent-3-en-2-one^30^ with either 1-(*tert*-butyl)-4-ethynylbenzene or 1-(*tert*-butyl)-4-[(4′-ethynylphenyl)ethynyl]benzene^31^ to give the corresponding ligands (3-(4′-((4″-(*tert*-butyl)phenyl)ethynyl)phenyl)-4-hydroxypent-3-en-2-one (**L**^**2**^H) and 3-(4′-((4″-((4‴-(*tert*-butyl)phenyl)ethynyl)phenyl)ethynyl)phenyl)-4-hydroxypent-3-en-2-one (**L**^**3**^H). These ligands and 4-hydroxy-3-phenylpent-3-en-2-one (**L**^**1**^H) were treated with *t*-BuOK and reacted with either [(ppy)_2_IrCl]_2_ to give complexes **1** and **3** or [(F_2_ppy)_2_IrCl]_2_ to give the complexes **2**, **4**, and **5**. Unfortunately, this method prevented the isolation of pure (ppy)_2_Ir(**L**^**3**^) (aOPE3 shown in Figure 1) since the complex did not produce crystals. The difluoro derivative (F_2_ppy)_2_Ir(**L**^**3**^) **5** is the only aOPE3 complex successfully synthesized in this study.

**Figure 3:**
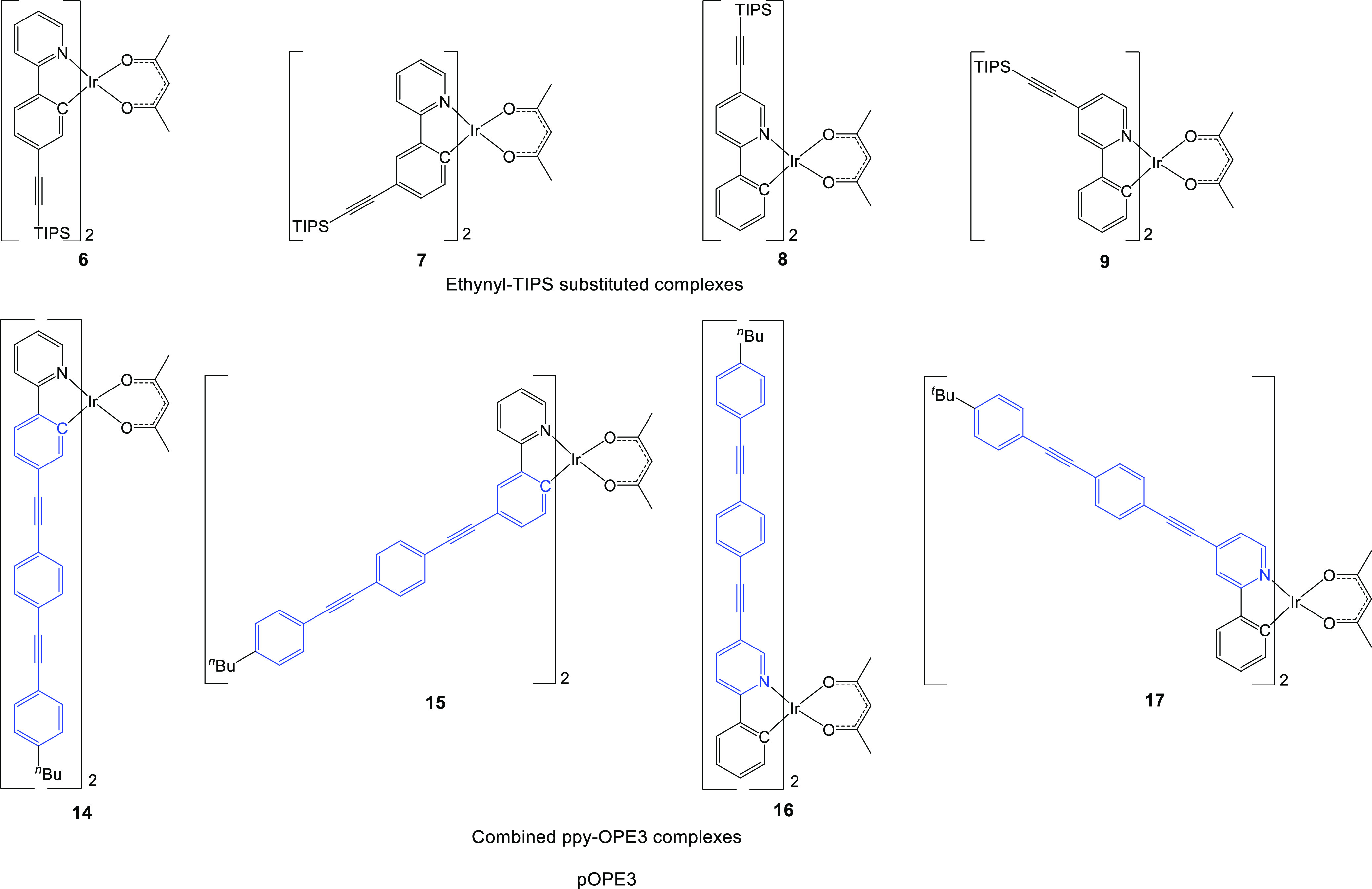
Substituted ppy complexes **6–9** and **14–17**. In the pOPE3 complexes, blue highlights the OPE3 component.

A series of complexes where the OPE3 motif is integrated with the ppy ligand to maximize electronic coupling to the rest of the complex were then targeted (pOPE3, Figure 3). Unlike the acac chelate, the position of substitution on the ppy ligand is a factor that must be considered, as any modifications to the pyridyl group directly impact the LUMO, while modifications to the phenylene group alter the HOMO. The complexes were prepared using a known approach,^27,32^ by treating the corresponding ethynyl-triisopropylsilyl (TIPS)-substituted ppy ligands (2-(4′-((triisopropylsilyl)ethynyl)phenyl)pyridine **L**^**4**^H,^33^ 2-{3′-[(triisopropylsilyl)ethynyl]phenyl}pyridine **L**^**5**^H, 2-phenyl-5-[(triisopropylsilyl)ethynyl]pyridine **L**^**6**^H, and 2-phenyl-4-[(triisopropylsilyl)ethynyl]pyridine **L**^**7**^H^27^ with IrCl_3_·3H_2_O and heating in 2-ethoxyethanol to give the corresponding dimer that was then reacted with acetylacetone to give the ethynyl-TIPS complexes **6–9**. These complexes were, in turn, coupled with 1-butyl-4-[(4′-iodophenyl)ethynyl]benzene via a Sonogashira coupling to give the corresponding pOPE3 complexes **14–17**.

**Figure 4:**
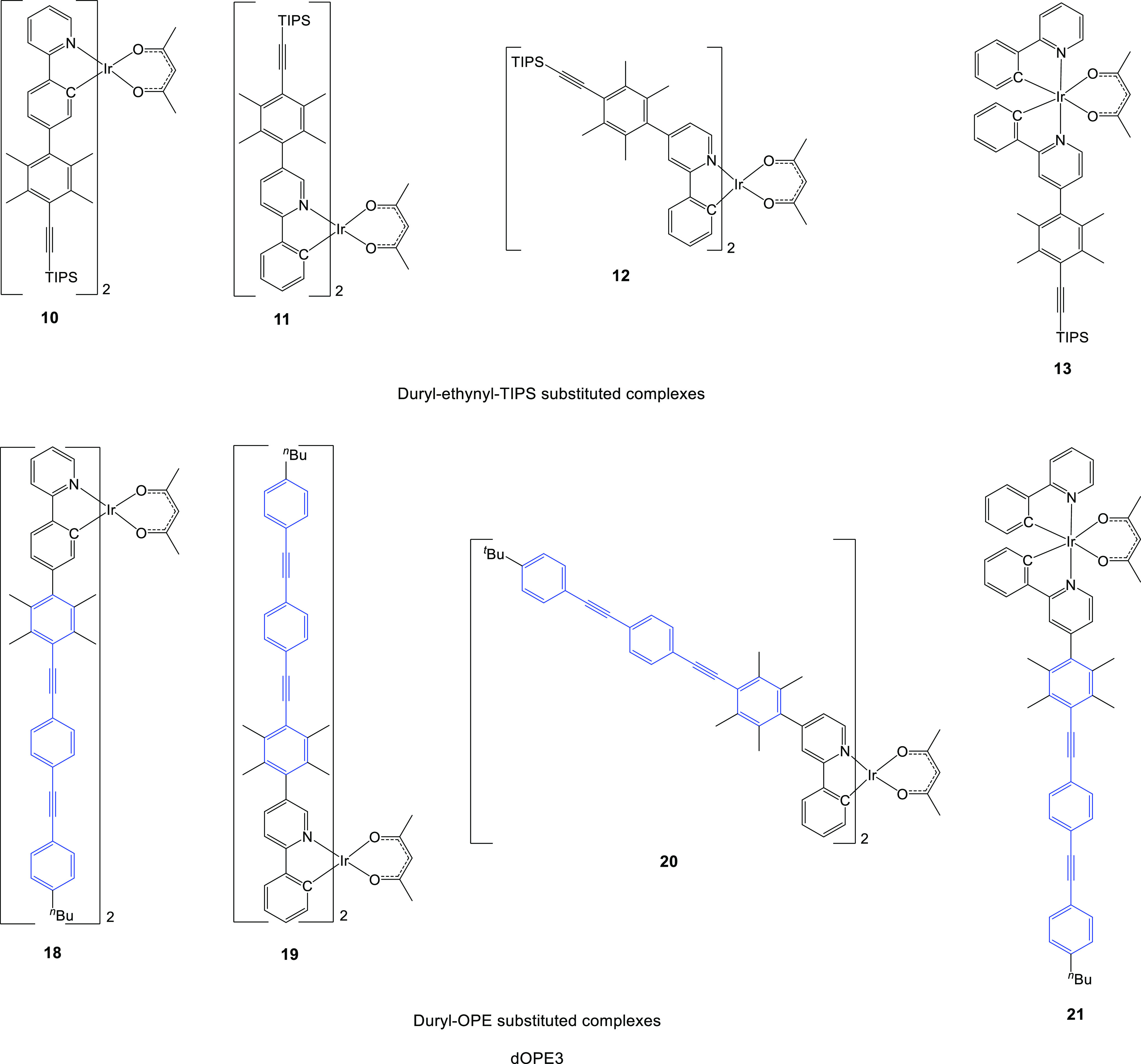
Substituted duryl–ppy complexes **10–13** and **18–21**. In the dOPE3 complexes, blue highlights the OPE3 component. Complex **11** could not be isolated in the pure form.

To provide a series of complexes, where OPE3 is attached to the ppy ligand but also electronically decoupled, a series of durylene-linked complexes dOPE3 (Figure 4) were synthesized. Using the same synthetic approach as that used for the pOPE3 complexes, duryl-ethynyl-TIPS ligands were first formed. The ligands were synthesized by coupling the respective 4,4,5,5-tetramethyl-1,3,2-dioxaborolane (BPIN)-substituted 2-phenylpyridine with diiododurene through a Suzuki–Miyaura reaction, followed by coupling the ethyne TIPS-CCH via a Sonogashira reaction to give ligands **L**^**8–10**^H. Unfortunately, 2-(4′-iodo-2′,3′,5′,6′-tetramethyl-[1,1′-biphenyl]-3-yl)pyridine was not produced in significant quantities under these conditions, possibly due to the formation of a palladium complex with the pyridine ring during the reaction. This ligand, if formed in sufficient amounts, would have produced a target dOPE3 complex with the OPE3 moiety substituted at the 4′-position of the phenyl ring as for complex **15**.

Each of the ligands **L**^**8–10**^H made was coordinated with iridium followed by acetylacetone as before to give the duryl-ethynyl-TIPS-substituted complexes **10**–**12**. Complex **11** could not be isolated in its pure form here and was used as a crude starting material for the next reaction step. The dOPE3 complexes **18–20** were prepared from the TIPS complexes **10**–**12** by the usual Sonogashira coupling procedures as described for the syntheses of the pOPE3 complexes.

To explore whether one OPE3 unit instead of two OPE3 units in a dOPE3 complex significantly influences the lifetimes of these dOPE complexes, a tris-heteroleptic complex precursor was synthesized. This mixed ligand system was achieved using the Edkins method,^32,34^ reacting a 1:1 mixture of ppyH:**L**^**10**^H with 1 equiv of IrCl_3_·3H_2_O under standard conditions to give the dimer mixture. This was treated with acetylacetone and K_2_CO_3_, yielding the three complexes Ir(ppy)_2_(acac), Ir(**L**^**10**^)_2_(acac) (**12**), and the desired complex Ir(**L**^**10**^)(ppy)(acac) (**13**) with a 21% yield. The mono-TIPS complex **13** was then converted to the tris-heteroleptic dOPE complex **21** where the OPE unit was substituted at the pyridyl ring. The Edkins method was attempted with **L**^**8**^H to obtain another tris-heteroleptic dOPE complex with the OPE unit at the phenylene ring of ppy, but the difference in solubility between **L**^**8**^H and ppyH in ethoxyethanol resulted in only Ir(ppy)_2_(acac) and complex **10** being formed.

### Molecular Structures

The structures of complexes **1–3**, **6**, **10**, and **14** were confirmed by single-crystal X-ray crystallography. Each complex shows a slightly distorted octahedral coordination of the Ir atom stabilized by a pair of intramolecular C–H···O contacts between the *ortho*-H atoms of the pyridinyl ring and the O atoms of the acac chelate. Various π···π intermolecular interactions between aromatic fragments and C–H···O/π weak hydrogen bonds are the main features found in packing motifs of all studied complexes.

**Figure 5:**
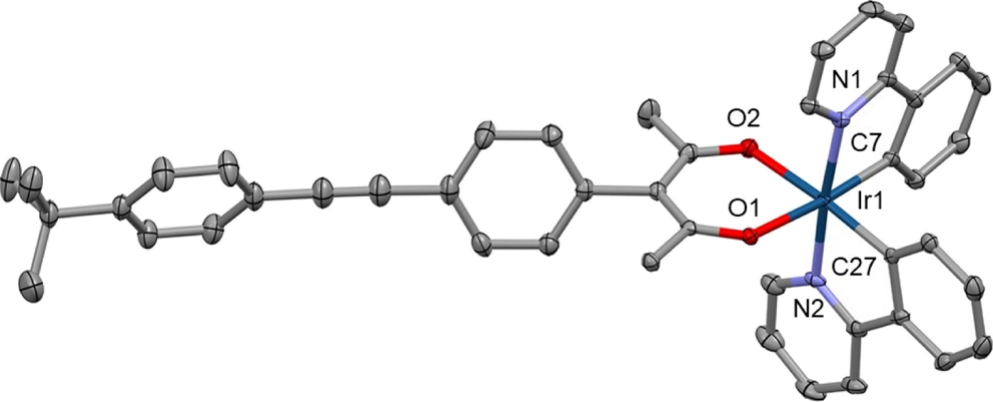
Crystal structure of **3**. Solvent molecule and disorder removed for clarity and thermal ellipsoids displayed at 50% probability.

**Figure 6:**
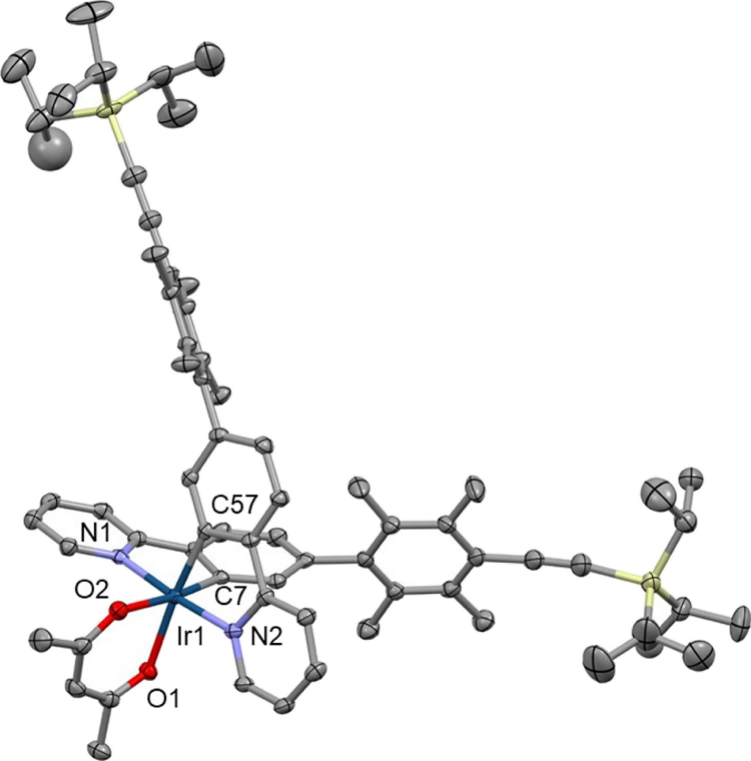
Crystal structure of **10**. Solvent molecule and disorder removed for clarity and thermal ellipsoids displayed at 50% probability.

**Figure 7:**
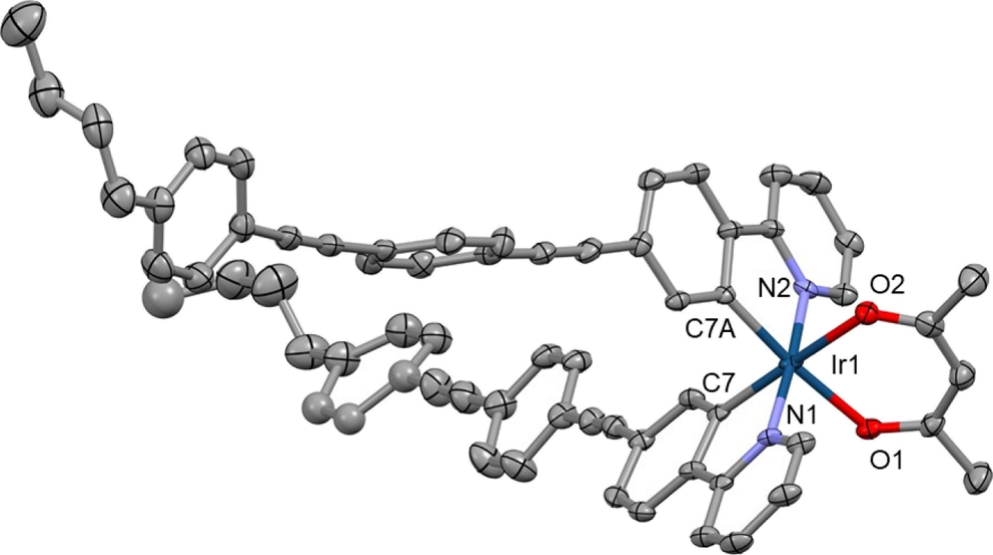
Crystal structure of **14**. Solvent molecule and disorder removed for clarity and thermal ellipsoids displayed at 50% probability.

The aryl groups attached to the acac ligand in complexes **1–3** are perpendicular to the plane of the acac chelate due to steric factors, so these aryl groups do not contribute to the π-conjugation of the acac chelate. Complex **10** has duryl rings attached to the phenylenes in the ppy chelates which are perpendicular to the phenylpyridyl planes, so these aryl groups do not contribute to the π-conjugation of the ppy chelates. By contrast, in complex **14**, there are perpendicular and planar orientations between the ppy planes and the bis(ethynylphenylene) groups showing that high conformational lability and extended π-conjugation between the ppy chelate and the OPE motif exist in the planar conformers (Figures 5–7).

### Computations

Electronic structure calculations were carried out with the B3LYP functional using the LANL2DZ pseudopotential for iridium and the 3-21G* basis set for other atoms combined with the integral equation formalism-polarizable continuum model method using dichloromethane (DCM) as the solvent on all optimized ground-state geometries of iridium complexes **1–21**. Comparison of the predicted emission wavelengths among the three model chemistries B3LYP/3-21G*:LANL2DZ, B3LYP/6-31G(d):LANL2DZ, and CAM-B3LYP/6-31G(d):LANL2DZ by time-dependent density functional theory (TD-DFT) on the optimized ground-state geometries with the observed emission wavelengths in the complexes here shows the model chemistry B3LYP/3-21G*:LANL2DZ to have good agreement with experimental data (see Table S3) compared to other model chemistries. The model chemistry B3LYP/3-21G*:LANL2DZ has indeed been shown to be suitable for related iridium complexes elsewhere.^32,35,36^

An analysis of the orbital contributions summarized in Table 1 (see also Tables S4–S8) for the substituted acac complexes **1–4** revealed the typical Ir(ppy)_2_(acac)/Ir(F_2_ppy)_2_(acac) arrangements with the HOMO consisting of both the iridium(d) and the phenylate(π) characters, while the LUMO was dominated at the pyridyl(π*) unit. Complex **5** exhibited a significant difference in the LUMO where the orbital was exclusively localized at the OPE component of the acac ligand rather than at the pyridyl(π*) unit.

**Table 1:** Molecular Group Contributions (%) in Frontier Orbitals for Complexes **1–21**^a^

		HOMO molecular group contributions (%)						LUMO molecular group contributions (%)				
complex	HOMO energy (eV)	Ir	acac	Ph	Py	R	LUMO energy (eV)	Ir	acac	Ph	Py	R
**1**	–5.11	47	7	40	6		–1.51	5	1	28	66	
**2**	–5.47	48	8	38	6		–1.65	5	1	27	67	
**3**	–5.13	47	6	41	6		–1.52	5	2	28	65	
**4**	–5.49	48	8	38	6		–1.66	5	1	28	66	
**5**	–5.49	49	7	38	6		–1.96	0	100	0	0	
**6**	–5.27	48	6	39	6	1	–1.81	3	0	36	50	11
**7**	–5.20	40	5	38	5	12	–1.63	5	1	30	64	0
**8**	–5.19	46	6	40	6	2	–1.91	3	0	24	59	14
**9**	–5.20	47	6	41	6	0	–1.99	4	1	9	66	20
**10**	–5.16	48	6	40	6	0	–1.54	4	1	29	65	1
**11**	–5.13	48	6	40	6	0	–1.55	5	1	27	65	2
**12**	–5.14	48	6	40	6	0	–1.55	5	1	27	65	2
**13**	–5.13	48	6	40	6	0	–1.54	5	1	26	66	2
**14**	–5.27	43	6	39	7	5	–2.11	1	0	25	23	51
**15**	–5.12	31	4	33	4	28	–1.84	1	0	16	4	79
**16**	–5.19	43	6	39	7	5	–2.24	1	0	12	33	54
**17**	–5.19	48	6	41	5	0	–2.35	3	1	3	38	55
**18**	–5.17	48	6	40	6	0	–1.84	0	0	0	0	100
**19**	–5.13	47	6	41	6	0	–1.90	0	0	0	1	99
**20**	–5.14	47	6	41	6	0	–1.90	0	0	0	1	99
**21**	–5.13	48	6	40	6	0	–1.90	0	0	0	0	100

a R represents the substituent(s) at the ppy ligand(s).

Although used as synthetic precursors, the electronic structures of the TIPS-ethynyl (**6–9**) complexes were analyzed to contrast the difference in behaviors resulting from the substitution of an alkyne compared to the formation of an analogous complex containing an OPE3 unit, i.e., pOPE3. The frontier orbitals for TIPS-ethynyl complexes (see also Tables S9–S12) revealed that the addition of the ethynyl-TIPS group to the ppy ligand had a significant effect on the LUMOs of complexes **6**, **7**, **8**, and **9** with the phenylene units contributing 36% (**6**)–9% (**9**) to the LUMOs in addition to stabilizing the orbital and a negligible contribution to the HOMO of the complexes (Table 1). The frontier orbitals in **6** are intriguing as the phenylene units significantly contribute almost equally to both the LUMO (36%) and HOMO (39%), whereas the related complexes **7–9** have less phenylene character in their LUMOs.

For the pOPE3 complexes **14–17**, the phenylenes in ppy units still contribute to the LUMOs 25% (**14**)–3% (**17**) along with the bis(phenyleneethynylene) units at 51% (**14**)–79% (**15**). The frontier orbitals in complex **15** differ markedly from those in other pOPE3 complexes where the OPE3 units have a 79% contribution to the LUMO and a 28% contribution to the HOMO.

The durTIPS complexes (**10**, **12**, and **13**) have a near-identical frontier orbital composition to the parent complex Ir(ppy)_2_(acac), with the HOMO being localized to the iridium and phenylene, while the LUMO is localized at the pyridyl unit with negligible differences between the isomers. The dOPE3 complexes (**18**, **19**, **20**, and **21**) also showed a HOMO localized to the iridium and phenylene, but both the LUMO and the LUMO + 1 (LUMO only for **21**) were localized exclusively on the OPE3 substituent.

TD-DFT calculations were also performed on these complexes to predict the lowest energy triplet transitions S_0_ ← T_*n*_ in their emissions based on the mirroring of the corresponding predicted S_0_ → T_*n*_ transitions. While complexes **1–4** are expected to give the iridium–phenylene to pyridyl(π*) transitions (^3^MLCT), complex **5** is predicted to have the lowest energy transition from ligand-centered OPE(π) to OPE(π*) at the OPE motif. A similar ^3^LC transition at the pyrene was predicted by Spaenig et al. for their pyrene-substituted acac complex.^28^

All TIPS complexes except **6** are predicted to have lowest energy transitions from iridium–phenylene to pyridyl(π*) (^3^MLCT) with varying minor contributions from the phenylene unit in the pyridyl(π*) state. By contrast, **6** is expected to have a lowest energy iridium–phenylene to pyridyl-phenylene (π*) transition which may be regarded as a mixed triplet-state metal to ligand charge transfer (^3^MLCT) and ^3^LC(phenylene) transition.

All pOPE3 complexes except **15** are shown computationally to have lowest energy transitions from iridium–phenylene to pyridyl-OPE3(π*) (^3^MLCT) with minor contributions from the phenylene unit in the π* state. Complex **15** differs from other pOPE3 complexes as the OPE3 motif in **15** dominates the frontier orbitals especially the LUMO where there is little pyridyl character. The lowest energy transition expected in **15** is therefore iridium-OPE3 to OPE3(π*) which is ^3^MLCT admixed with ^3^LC(OPE). As predicted from the calculated frontier orbitals for all dOPE3 complexes **18–21**, the lowest energy transitions are iridium–phenylene to OPE3(π*) (^3^MLCT).

### Electrochemistry

Cyclic voltammograms were recorded for all the complexes in 0.1 M tetrabutylammonium hexafluorophosphate in DCM and referenced against ferrocene (i.e., *E*_1/2_ FeCp_2_/[FeCp_2_]^+^ = 0.00 V). Each of the iridium complexes (**1**–**21**) showed a single oxidation wave usually attributed to the Ir(III)/Ir(IV) couple (see Table S66). No reduction waves were observed within the solvent working range. For the modified acac complexes **1**–**5**, the difluoro substituents at the ppy ligands had the most significant impact on the oxidation potentials, with **1** and **3** having *E*_1/2_(ox) = 0.39–0.41 V, while the fluorinated analogues **2**, **4**, and **5** had *E*_1/2_(ox) = 0.71–0.72 V. The higher HOMO energies in the fluorinated analogues based on their higher oxidation potentials are expected as the phenylene units contribute to the HOMOs in typical Ir(ppy)_2_(acac) complexes, and the electron-withdrawing fluoro-substituents are attached to the phenylene units. All other complexes show oxidation potentials in the 0.35–0.53 V range, suggesting that the HOMO energies in these complexes are similar to those in complexes **1** and **3**.

### Electronic Absorbance

The electronic absorbance spectra for complexes **1**–**21** were recorded in DCM (see Figures S70–S75). Based on literature data of closely related complexes, each of these complexes exhibited low-intensity bands (*ca.* 400–550 nm) attributed to ^3^MLCTs, with higher-intensity bands (*ca.* 350–400 nm) associated with ^1^MLCTs, and the remaining higher-energy transitions with substantially higher intensities were attributed to π → π* transitions.

For the substituted acac series (**1–5**), two notable differences were observed in the electronic absorbance. The first was that the low-energy band attributed to ^3^MLCTs for complexes **2**, **4**, and **5** was blue-shifted by *ca.* 30 nm as compared to that of complexes **1** and **3** with the difference attributed to the fluorine groups on the phenylene unit stabilizing the HOMO. The second difference was that in the π → π* region, as the length of the acac ligand increased, i.e., **2** → **4** → **5**, the extinction coefficient (ε) of the π → π* transitions increased, and the transitions red-shifted. This was attributed to the superposition of the ppy ligand absorbance with the extended conjugation at the acac ligand going from a phenyl group to an OPE3 group. Similar observations were reported by Favale et al. for their isocyanide-based complexes and Spaenig et al. for their substituted acac complexes.^28,37^

The ethynyl-TIPS-substituted complexes (**6–9**) displayed similar absorbance profiles to Ir(ppy)_2_(acac) with complexes **8** and **9** red-shifting by *ca.* 20 nm at the low energy transitions because of the stabilization of the LUMO resulting from the extension of the conjugation of the pyridyl ring to the ethynyl unit, while complexes **6** and **7** had substituents on the phenylene ring resulting in a negligible effect on the LUMO. The differences in complexes **6–9** were further exaggerated in their pOPE3 analogues (**14–17**) with complexes **16** and **17** showing a *ca.* 40 nm red shift of the low-energy transitions as compared to complexes **14** and **15**. However, in the π → π* region, complexes **15** and **17** displayed a complex superposition of the ppy and OPE3 absorptions, while complexes **14** and **16** exhibited a single broad absorbance feature. These differences could be attributed to the OPE3 unit being para to the ppy axis (i.e., 4 and 4′ positions in Figure 1) in complexes **14** and **16**, resulting in an extension of the ligand conjugation, while in complexes **15** and **17**, the OPE unit was meta to the ppy axis (5 and 5′ positions), resulting in a break in conjugation between the substituent and the ligand. Analogous behaviors were observed by Yan et al. in their oligofluorene-substituted complexes.^26^

Both the duryl-ethynyl-TIPS (**10**, **12**, and **13**) and the dOPE3 complexes (**18**–**21**) displayed almost identical absorption spectra in the ^3^MLCT and ^1^MLCT regions like the spectrum of the parent Ir(ppy)_2_(acac) complex. The similarities are due to the duryl substituent electronically decoupling from the Ir(ppy)_2_(acac) center. The dOPE3 complexes (**18**–**21**) differ slightly from the TIPS complexes where new transitions at 300–400 nm are present due to the OPE3 units. In the case of complex **21**, the extinction coefficient was almost half that of the related isomer **20**, as there is one OPE3 unit, instead of two, in **21**.

### Steady-State Emissions

The steady-state emissions of the complexes were recorded in degassed solutions by using a range of excitation wavelengths to probe the excitation dependence of the complexes; the corresponding data are summarized in Table 2 (spectra provided in Supporting Information, Figures S76–S105).

**Table 2:** Photophysical Data for Complexes **1–10** and **12–21** Recorded in Dichloromethane^a^

			lifetime (τ, μs), excitation (nm)					
paper code	λ_Emission_ (nm), RT, DCM	PLQY (Φ)	337	405	*T*_1_^c^ (eV)	*k*_r_ (10^5^s^–1^)	*k*_nr_ (10^5^s^–1^)	pure radiative lifetime (τ_0,μs_)
Ir(ppy)_2_(acac)^7^	520	0.71		1.90	2.55	3.73	1.52	2.67
Ir(F_2_ppy)_2_(acac)^40^	484	0.63		0.87		7.22	4.24	1.38
**1**	527	0.48	1.13	1.13	2.53	4.24	4.60	2.35
**2**	487	0.14	0.22	0.22	2.66	6.36	39.09	1.57
**3**	528	0.40	0.92	0.92	2.46	4.34	6.52	2.30
**4**	490	0.11	0.19	0.19	2.64	5.78	46.84	1.72
**5**^b^	389, 484		180	N/A				
**6**	540	0.55	23.1	3.10	2.34	1.76	1.46	5.67
**7**	529	0.68	1.95	1.92	2.50	3.57	1.64	2.80
**8**	553	0.63	1.97	1.99	2.29	3.16	1.92	3.16
**9**(27)	597	0.57	1.00	1.00	2.25	5.70	4.30	1.75
**10**	522	0.55		2.23	2.48	2.46	2.01	4.06
**12**(27)	521	0.66		1.01	2.46	6.53	3.37	1.53
**13**	524	0.56		1.38	2.44	4.02	3.22	2.49
**14**	567	0.34	5.95	5.47	2.23	0.60	1.20	16.51
**15**	539	0.55	32.8	31.9	2.33	0.17	0.14	58.0
**16**	576	0.55	11.5	3.55	2.21	1.54	1.26	6.45
**17**(27)	611	0.38	0.69	0.69	2.13	5.51	8.99	1.82
**18**	526	0.46	49.5	1.06	2.38	4.31	5.13	2.32
**19**	524	0.44	81.9	9.30	2.40	0.47	0.62	21.1
**20**(27)	524	0.49	625	13.5	2.40	0.36	0.38	27.5
**21**	524	0.60	181.8	18.0	2.42	0.33	0.21	30.0

a All lifetime recorded at room temperature. The radiative *k*_r_ and non-radiative *k*_nr_ values were calculated according to the equations *k*_r_ = Φ/τ and *k*_nr_ = (1 – Φ)/τ, respectively, from the quantum yield Φ and the lifetime τ.

b The complex was found to be too unstable under irradiation to obtain an accurate Φ (see the Supporting Information for further details).

c Recorded in 2-methyltetrahydrofuran at 77 K.

The complexes with substituted acac ligands, **1–4**, showed a broad emission band at room temperature that, upon cooling to 77 K, resolved into two clearly defined bands with a third low-energy shoulder, resulting from the vibronically structured emission as reported by Lamansky et al. for Ir(ppy)_2_(acac) and Ir(F_2_ppy)_2_(acac).^38^ The fluorinated complexes, **2** and **4**, had emissions blue-shifted by *ca.* 40 nm with respect to complexes **1** and **3**, as was observed with the respective parent complexes, Ir(ppy)_2_(acac) and Ir(F_2_ppy)_2_(acac).^39^ When the excitation wavelength (λ_ex_) was varied from λ_ex_ = 330 to 450 nm, complexes **1** and **2** only displayed a variation in the emission intensity, which could be attributed to the varied absorbance at these wavelengths. However, when complexes **3** and **4** were excited, λ_ex_ < 350 nm, additional structured emissions were observed at λ_emis_ = 350–400 nm; this was attributed to the fluorescence of the phenylene–ethynylene–phenylene (OPE2) moiety of the acac ligand. Additionally, the PLQYs (Φ) of complexes **1–4** were *ca.* 20% lower than those of their respective parent complexes, which could be attributed to the increased size of the ancillary ligand, resulting in more degrees of freedom facilitating additional non-radioactive decay processes.

**Figure 8:**
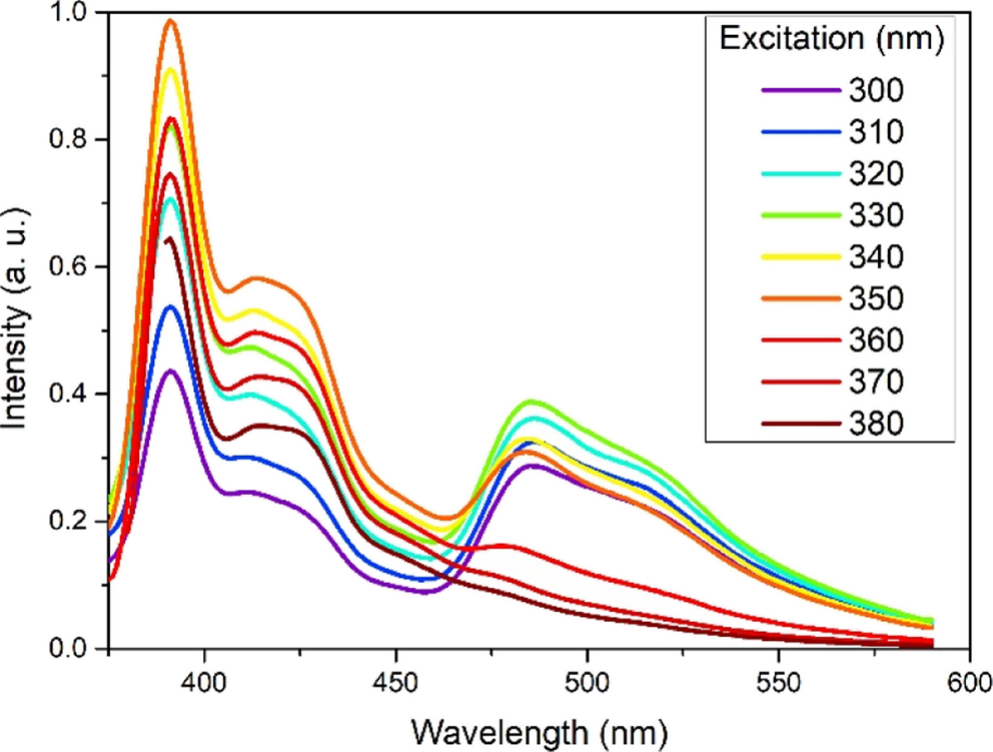
Emission behavior of complex **5** on varying the excitation wavelength, recorded in DCM at room temperature.

Complex **5** displayed significantly different behavior from that of complexes **1–4** (see Figures S87–S91): when excited at λ_ex_ > 360 nm, a structured emission occurred at λ_emis_ = 390–416 nm with a weak shoulder at λ_emis_ = 450–600 nm (Figure 8). However, when excited at λ_ex_ < 360 nm, a combination of both the previously observed emission at λ_emis_ = 390–416 nm and a strong emission similar to that of the parent complex Ir(ppy)_2_(acac) was observed at λ_emis_ = 460–600 nm. Upon aeration, no emission was observed. Although the emission at λ_emis_ = 390–416 nm was thought to originate from the OPE3 fluorescence, the oxygen quenching indicated that the emission originated from an ^3^LC state. Such a phenomenon was observed by Spaenig et al. for a pyrene-substituted acac complex^28^ as a result of a triplet–triplet energy transfer. The triplet energy of BPEB, a model of OPE3, is 2.53 eV,^27^ close to that of Ir(F_2_ppy)_2_(acac) (2.56 eV);^40^ this small difference between the ^3^LC and the ^3^MLCT energies (Δ*E*_TT_ = 30 meV) accounted for the lower extent of quenching than that of the Spaenig et al. system with Δ*E*_TT_ = 450 meV as the small Δ*E*_TT_ did not significantly hinder the reverse process. The threshold of 360 nm (3.44 eV) for the emission behavior to change was also critical as it coincided with the *S*_1_ energy of BPEB (*S*_1_ = 3.44 eV).^27^ This suggested that excitations above this level could populate *S*_1_ and, through intersystem crossing, could populate the triplet states.

Each of the TIPS complexes **6**–**9** displayed a different emission behavior as a result of the substitution position (see Figure S78). Complexes **6**–**8** exhibited a structured emission at λ_emis_ = 475–700 nm at room temperature which became further resolved upon cooling to 77 K (see Figure S83), suggesting that these complexes had an admixture of the ^3^LC and the ^3^MLCT emission characteristics, consistent with the TD-DFT calculations and the reported properties of the structurally related complexes.^34^ Complex **9** in comparison exhibited only a broad featureless emission at λ_emis_ = 500–750 which was not resolved at low temperatures, indicating a pure ^3^MLCT emission character as was previously reported.^27^ In addition to the emission character, there was a notable shift in the highest energy emission, i.e., λ_emis_**7** < **6** < **8** < **9**. Complex **7** had the emission most similar to that of the parent Ir(ppy)_2_(acac) owing to the combination of the substitutions occurring on the phenylene ring, i.e., HOMO-dominated, and the substitution was in the meta position (5′ in Figure 1) relative to the ppy axis, resulting in a break in conjugation. For complexes **6** and **8**, the substitution was para (4′ and 5 positions, respectively) to the ppy axis, linearly extending the conjugation of the ppy ligand. Finally, for complex **9**, the substitution was on the pyridine, i.e., LUMO-dominated, and in the meta position, resulting in the LUMO being exclusively stabilized, favoring ^3^MLCT.

**Figure 9:**
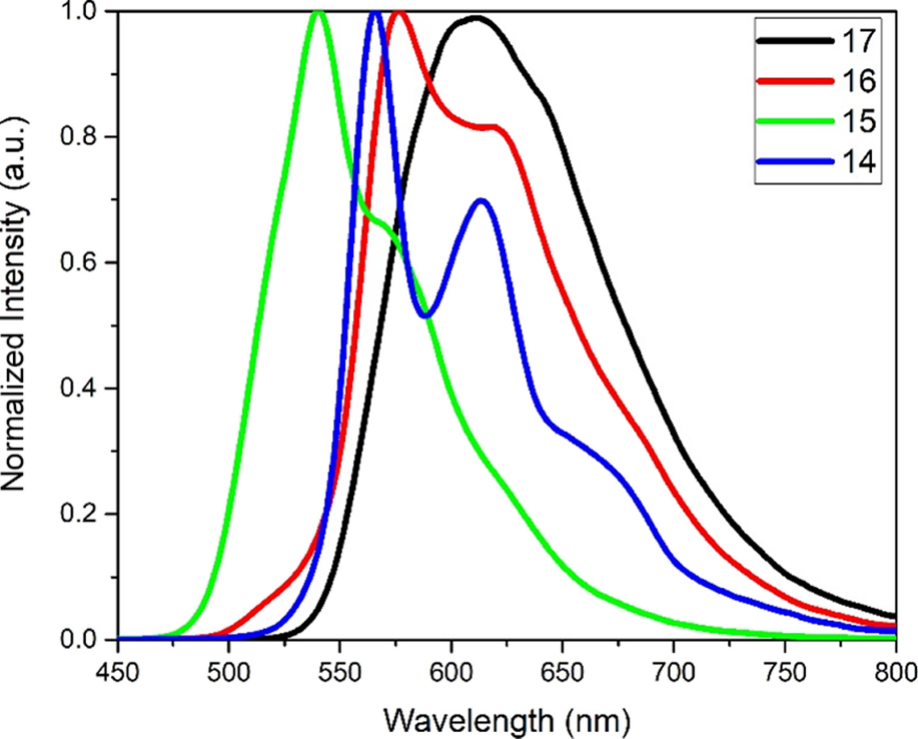
Steady-state emission spectra for complexes **14**–**17** recorded in DCM at room temperature.

The pOPE3 complexes (**14**–**17**) exhibited similar behavior to the TIPS complexes (**6**–**9**) (see Figure 9 and Figures S80 and S85) but red-shifted, e.g., complex **14** with λ_max_ = 566 nm, as compared to the shorter analogue **6** with λ_max_ = 543 nm, because of the extended conjugation of the ligand. As was observed with the acac series (**1–5**), the increase in the ligand length was accompanied with a decrease in the quantum yields, Φ. Both sets of complexes, **6**–**9** and **14**–**17**, did not display any additional emission features with varied excitation. Unlike the aryl groups at the acac chelates in complexes **1**–**5**, the OPE3 motifs in the pOPE3 complexes were electronically coupled to the metal center preventing REET.

**Figure 10:**
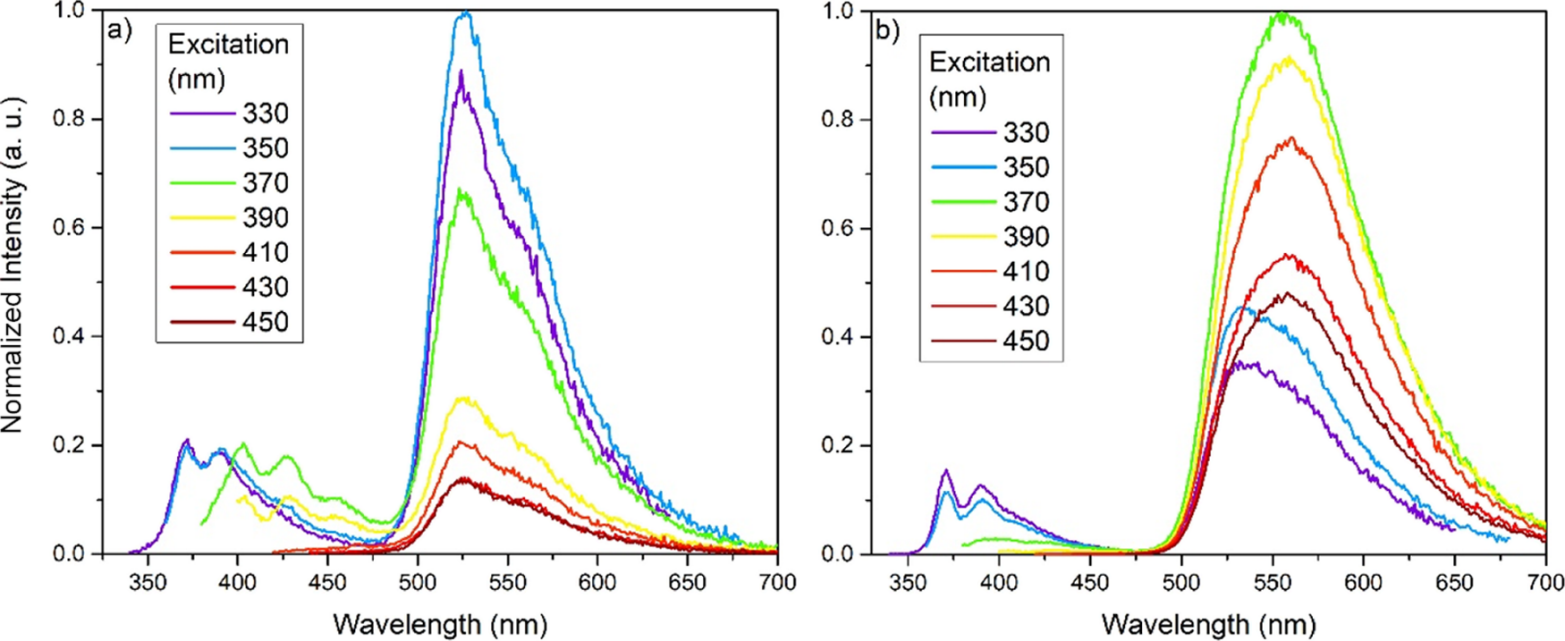
Emission behavior of complexes (a) **18** and (b) **20** upon varying the excitation wavelength recorded in MeTHF at room temperature.

Each of the duryl-substituted complexes (**10**, **12**, **13**, and **18**–**21**) displayed an identical emission at λ_max_ = 524 nm when λ_ex_ > 410 nm, matching that of the parent complex Ir(ppy)_2_(acac) at 520 nm, owing to the duryl group electronically decoupling the substituents from the metal center.^27^ The duryl-TIPS complexes (**10**, **12**, and **13**) displayed no additional emissive features when the excitation was varied. The dOPE3 complexes showed a slight lowering of the T_1_ energy with respect to the duryl-TIPS analogues (20–80 meV), consistent with the TD-DFT calculations, and they displayed additional features with higher energy excitation. When λ_ex_ < 360 nm, an additional structured emission at 350–450 nm was observed for complexes **18** and **20** (see Figure 10), most likely to be fluorescence from OPE3 units. Complex **21** exhibited a similar emission but red-shifted to λ_emis_ = 375–475 when 350 < λ_ex_ < 410 nm, and complex **19** displayed a combination of the two features dependent on the excitation wavelength used.

### Time-Dependent Emission

The emission lifetimes of the peak emission for each complex were recorded in degassed DCM (summarized in Table 2, traces in Figures S106–S129). Because of the excitation wavelength dependence observed for some of the complexes, the emission lifetimes were recorded at both excitation wavelengths at 337 and 405 nm.

**Figure 11:**
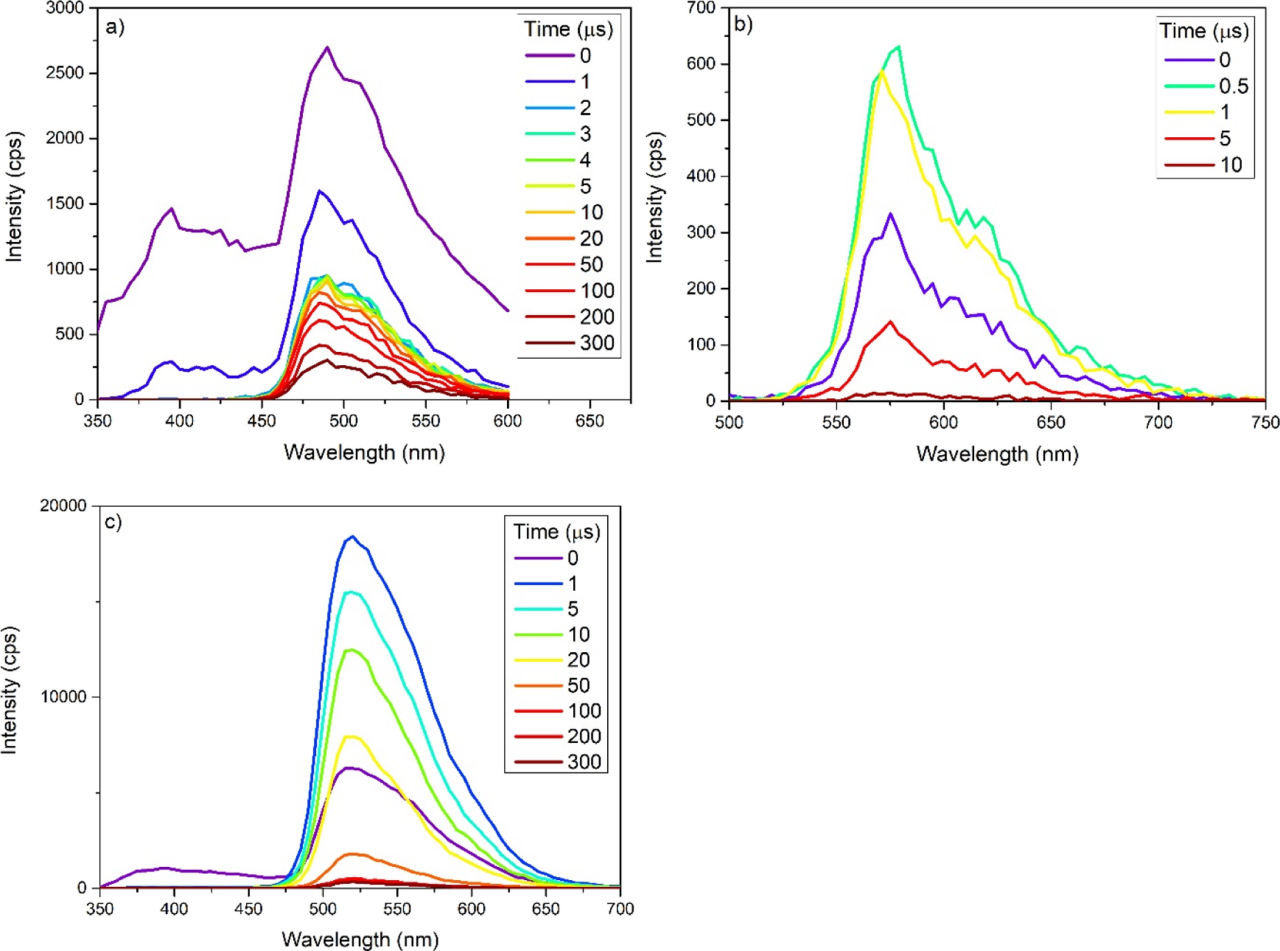
Emission spectra of complexes (a) **5**, (b) **16**, and (c) **20** at specific time intervals after the initial emission, recorded in DCM excited at 337 nm.

**Figure 12:**
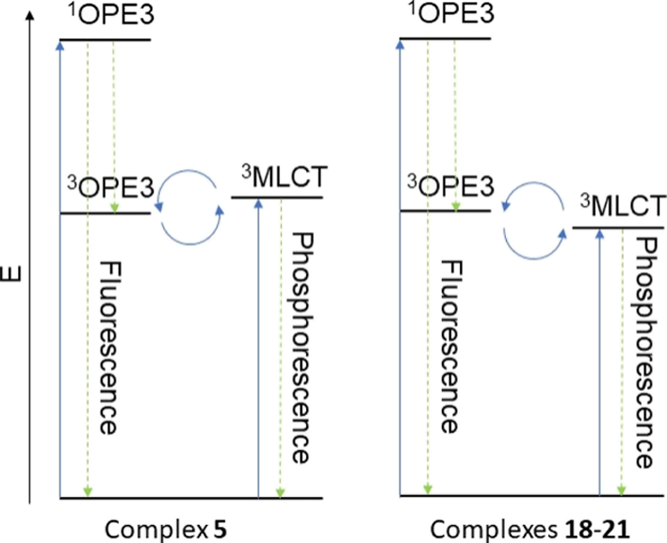
Energy level diagram depicting the relevant spectroscopic states for complexes **5** and **18–21**.

The substituted acac complexes, **1–4**, displayed excitation-independent lifetimes that were significantly shorter (τ = 0.19–1.13 μs) than those of the respective parent complexes Ir(F_2_ppy)_2_(acac) (τ = 0.87 μs) and Ir(ppy)_2_(acac) (τ = 1.9 μs). The shorter lifetimes were consistent with the decreased Φ, which could be explained by the increased *k*_nr_ resulting from the increased degrees of freedom with longer ancillary ligands. As previously discussed, complex **5** displayed negligible emission for excitations of λ_ex_ > 360 nm; therefore, only the data for a λ_ex_ = 337 nm excitation were recorded, revealing the exceptionally long τ_337_ = 180 μs, significantly longer than that of the pyrene analogue (40 μs) reported by Spaenig et al.^28^ To further elucidate this behavior, a time-resolved spectrum was obtained (see Figures 11 and S134). This showed that the 350–450 peak, attributed to the OPE3 unit, occurred for <0.1 μs. Concurrently, the 450–600 nm peak nearly halved in intensity, followed by a slow decay of the 450–600 nm peak. Such behavior is consistent with an initial fluorescent emission followed by a phosphorescent emission regulated by REET (see Figure 12).

The TIPS complexes (**7–9**) displayed excitation-independent emission lifetimes of τ_405/337_ = 1.00 (**9**)–1.95 μs (**7**), close to those of the parent complex Ir(ppy)_2_(acac) and consistent with emissions containing the ^3^MLCT character. However, complex **6** surprisingly displayed an excitation wavelength dependence of τ_405_ = 3.1 μs and τ_337_ = 23.1 μs. The longer lifetime of 23.1 μs could be due to the extension of the conjugation of the ppy ligand at the phenylene favoring the ^3^LC character emission, resulting in the increased emission lifetime in agreement with computations showing substantial phenylene character in the LUMO for **6** compared to **7–9**. The effect of the extended conjugation is more prevalent for the pOPE analogues, **14**–**17**. Complex **17** showed no excitation dependence with τ_405/337_ = 0.69 μs coupled with the broad featureless emission, indicating that the complex was exclusively ^3^MLCT in character. Complexes **14**–**16** also displayed little excitation dependence with τ_405_ = 5.47 (**14**), 31.9 (**15**), and 3.55 (**16**) μs, while τ_337_ = 5.95 (**14**), 32.8 (**15**), and 11.5 (**16**) μs. However, complexes **14** and **16** show evidence of a double exponential decay. To further elucidate this behavior, time-dependent emission spectra of complex **16** were recorded with λ_ex_ = 337 nm (see Figures 11 and S133). Unlike in the case of complex **5**, the entire emission decayed concurrently without any evidence of a fluorescent feature. According to the pure radioactive lifetime and the structured emissions of complexes **14–16**, the conjugation resulted in an increase in the ^3^LC emission character, and this character was most prominent in **15** with a notably long lifetime of 32 μs. The MO and TD-DFT computations described earlier confirmed the large contributions of the OPE3 character on both frontier orbitals and the prevalence of the ^3^LC transition in **15** compared to other pOPE3 complexes. Similar lifetimes were observed by Edkins et al., where 1- and 2-(2′-pyridyl)pyrene was used in place of 2-phenylpyridine, resulting in varied ^3^LC contributions in the emissions with lifetimes of 2.7 and 37 μs, respectively.^18^

The duryl-TIPS complexes **10**, **12**, and **13** displayed excitation wavelength independence with lifetimes of τ_405/337_ = 1.01 (**12**)–2.23 (**10**) μs, similar to those of the parent complex Ir(ppy)_2_(acac) at 1.90 μs. In contrast, the dOPE3 complexes, **18**–**21**, revealed excitation wavelength dependence. Each dOPE3 complex displayed significantly longer emission lifetimes when excited at λ_ex_ = 337 nm than at λ_ex_ = 405 nm, e.g., complex **18** τ_405_ = 1.06 and τ_337_ = 49.5 μs. As mentioned previously, the durylene linker acts to electronically decouple the OPE3 unit from the Ir(ppy)_2_(acac) moiety. The triplet state of BPEB with *T*_1_ = 2.53 eV (in benzene) and Ir(ppy)_2_(acac) *T*_1_ = 2.55 eV would result in Δ*E*_TT_ = 20 meV if each of these components was completely isolated; however, the measured *T*_1_ energies were found to be 2.38–2.42 eV for the dOPE3 complexes, **18–21**. Based on the similarity of emission profiles between these complexes and Ir(ppy)_2_(acac), the assumption is made that the lower *T*_1_ energy is attributed to the OPE3 motif, as is suggested by the TD-DFT results. Therefore, Δ*E*_TT_ was likely to be closer to 130–170 meV, making the reversible energy transfer still thermally accessible. This was further supported by an examination of the time-resolved emission of complexes **18**–**21** (see Figure 11 for **20** and Figures S135–S141); all of them display the OPE3 fluorescence (350–450 nm) < 0.1 μs with the remaining emission consisting exclusively of the Ir(ppy)_2_(acac) phosphorescence with λ_max_ = 524 nm rather than the emission occurring from the organic chromophore ^3^LC, as has been observed in many iridium complexes with long emission lifetimes (see Figure 12).

The inclusion of the duryl group resulted in a system where the OPE3 unit and the Ir(ppy)_2_(acac) unit were electronically independent. Differences in the emission lifetimes were observed between positional isomers, i.e., **19** τ_405_ = 9.30 and τ_337_ = 81.9 μs and **20** τ_405_ = 13.5 and τ_337_ = 625 μs. There are examples of isomers having an impact on REET where the positional difference resulted in a change to either the ^3^MLCT or the ^3^LC state.^26,41^ However, the TD-DFT results for complexes **18–21** reveal that the triplet energies and MO compositions are very similar. Transition dipole moments (see Figures S65–S69) were calculated on several complexes to gain some insight, but there was no correlation with the different emission lifetimes. For complexes **19–21**, the OPE3 unit is at the pyridyl unit (4 and 5 positions in Figure 1), whereas for complex **18** with shorter lifetimes, the OPE3 unit is at the phenylene unit of the ppy chelate (4′ position). These positions suggest that the shorter distance between the OPE3 motif and the pyridyl group facilitates the REET process perhaps due to better matching of the triplet energies between the OPE3 and the Ir(ppy)_2_(acac) units at these positions.

Another consideration for the lifetime differences was that for complex **20**, the OPE3 units along the N_pyridine_–Ir–N_pyridine_ axis resulted in a *C*_2_ point group, and the higher symmetry increased the degeneracies of complex **20** with respect to those of the other positional isomers. Higher symmetry has previously been shown to extend the emission lifetime and increase Φ in lanthanide complexes.^42^ However, the lifetimes for complex **21** at τ_405_ = 18.0 and τ_337_ = 181.8 μs are longer than the lifetimes for **18** and **19**, thus suggesting that the position of the OPE3 unit rather than the symmetry is the main factor driving longer emission lifetimes.

The difference in the number of chromophores using a relationship by McClenaghan et al., where an increase in the emission lifetime is linked to the number of pyrenyl units, was τ = 2.73*n*_pyrenyl units_ + 0.87.^43^ On the basis of this chromophore number relationship, the 443 μs difference between **20** and **21** is largely attributed to the number of chromophores where the longer lifetimes in **20** are aided by the higher symmetry in the latter complex.

## Conclusions

Nine iridium complexes with acac and two ppy ligands were synthesized with the OPE3 motif attached or incorporated at either the acac or the ppy ligand. These systems were explored to examine how the triplet state of an OPE3 unit can influence their emission lifetimes and wavelengths, either concurrently or separately by electronically coupling or decoupling the OPE3 unit from the iridium system.

When the OPE3 motif is incorporated as part of the ppy ligand and thus electronically coupled with the iridium system, their emission lifetimes from 0.69 to 32.8 μs depend on the OPE3 contribution within the ppy-OPE3 ligand but are independent of the excitation wavelengths used. By contrast, when the iridium complexes are electronically decoupled from the OPE3 unit, their emission lifetimes are shown to be long at 50–625 μs at an excitation wavelength of 337 nm and 1.06–18.0 μs at 405 nm. Time-dependent emission measurements on these decoupled systems revealed two distinct emissions, fluorescence from the OPE3 moiety followed by phosphorescence from the iridium system. These observations are explained by intramolecular REET with the OPE3 unit acting as a triplet sensitizer with a suitable excitation wavelength leading to long emission lifetimes in the decoupled iridium-OPE3 systems.

## Supporting Information Available

Details of synthetic procedures and characterization, NMR spectra, crystallographic data, photostability study, physical measurements, computational details and analysis, cyclic voltammetry details, photophysics setup, steady state, low temperature, excitation, time-dependent emission, and lifetime decay data (PDF)
(PDF)
