# Supplementary material for: Tuning Emission Lifetimes of Ir(C^N)2(acac) Complexes with Oligo(phenyleneethynylene) Groups
Source: Inorg Chem. 2023 Jan 27;62(6):2793–805. doi: 10.1021/acs.inorgchem.2c03934 (PMC9930119; doi:10.1021/acs.inorgchem.2c03934)
Supplement: Supplementary file 1 — ic2c03934_si_001.pdf [file ic2c03934_si_001.pdf]

Supporting Information - Synthesis

Tuning Emission Lifetimes of Ir(C^N)$_2$(acac) Complexes with Oligo(phenyleneethynylene) Groups

Ross Davidson,[†*] Yu-Ting Hsu,[†] Mark A. Fox,[†] Juan A. Aguilar,[†] Dmitry Yufit[†], Andrew Beeby[†*]

†Department of Chemistry, University of Durham, South Road, Durham, DH1 3LE, England, UK

*To whom correspondence should be addressed. Email: Ross Davidson (ross.davidson@durham.ac.uk) and Andrew Beeby (andrew.beeby@durham.ac.uk).

Table of Contents

S1. Synthesis of reported compounds ……………………………….... S3

S2. NMR spectra of reported compounds …………………………..... S19

S3. Crystallographic data ……………………………………………..... S43

S4. Substituted acac complex photodegradation …………………..... S46

References …………………………………………………………..... S49

S1. Synthesis of reported compounds

General details. NMR spectra were recorded in deuterated solvent solutions on Varian VNMRS-600 spectrometer and referenced against solvent resonances (^{1}H, ^{13}C). Electrospray mass spectra (ESMS) data were recorded on a TQD mass spectrometer (Waters Ltd, UK) in acetonitrile or MALDI TOF MS data were recorded on an Bruker Autoflex II ToF/ToF. Purity of compounds were determined using microanalyses, performed by Elemental Analysis Service, London Metropolitan University, UK.

4-hydroxy-3-phenylpent-3-en-2-one (**L^{1}**H),[1] 2-(2′,4′-difluorophenyl)pyridine (F_2ppyH),[2] di-μ-chlorotetrakis[2-(2′-pyridinyl-κ*N*)phenyl-κ*C*]di-iridium [{Ir(ppy)$_2$(μ-Cl)}$_2$][3], di-μ-chlorotetrakis[3,5-difluoro-2-(2′-pyridinyl-κ*N*)phenyl-κ*C*]di-iridium ({Ir(F_2ppy)$_2$Cl}$_2$)[4], 2-(3-bromophenyl)pyridine[5], 2-(4′-bromophenyl)pyridine[6], 5-bromo-2-phenylpyridine[7], 2-(4′-(4″,4″,5″,5″-tetramethyl-1″,3″,2″-dioxaborolan-2″-yl)phenyl)pyridine[8], 2-(4′-((triisopropylsilyl)ethynyl)phenyl)pyridine (**L^{4}**H),[9] 2-phenyl-4-((triisopropylsilyl)ethynyl)pyridine (**L**^{7}H),[12] 2-phenyl-4-(2′,3′,5′,6′-tetramethyl-4′-((triisopropylsilyl)ethynyl)phenyl)pyridine (**L^{10}**H),[10] Ir(**L**^{7})$_2$(acac) (**9**),[10] Ir(ppy-Dur-≡-TIPS)$_2$(acac) (**12**),[10] Ir(ppy-≡-C_6H_4-≡-C_6H_4-^{t}Bu)$_2$(acac) (**17**),[10] and Ir(ppy-Dur-≡-C_6H_4-≡-C_6H_4-^{t}Bu)$_2$(acac) (**20**).[10] For information on atom labels see NMR spectra of reported compounds.

Acetylacetone based ligand general synthesis (L²H and L³H)

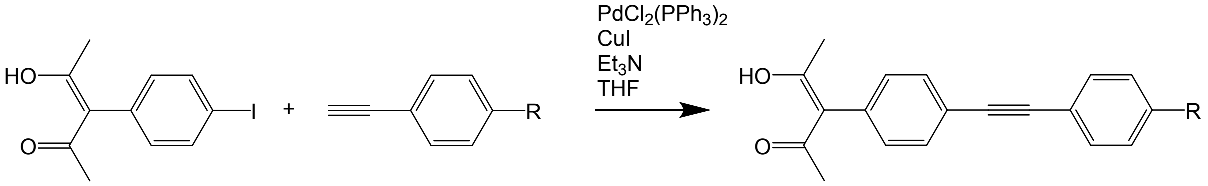

1-(tert-butyl)-4-ethynylbenzene (for **L**^{2}H) or 1-(tert-butyl)-4-((4′-ethynylphenyl)ethynyl)benzene (for **L**^{3}H) (1.66 mmol) was added to a solution containing 4-hydroxy-3-(4-iodophenyl)pent-3-en-2-one (500 mg, 1.66 mmol), CuI (15 mg, 0.08 mmol), $PdCl_2(PPh_3)_2$ (56 mg, 0.08 mmol), Et_3N (5.0 mL) in THF (20 mL) degassed by freeze-pump-thaw three times. The solution was stirred at room temperature for 16 hours before the solvent was removed under vacuo. The residue was purified using silica chromatography using a gradient from DCM:Hexane (1:1) to neat DCM.

3-(4′-((4″-(tert-butyl)phenyl)ethynyl)phenyl)-4-hydroxypent-3-en-2-one (**L**^{2}H). White solid. **Yield**: 468 mg (85%). **^{1}H NMR** (599.42 MHz; $CDCl_3$, 25 ^{0}C): δ_H 7.55 (d, $^3J_{HH}$ = 7.7 Hz, 2H, H$_a$), 7.48 (d, $^3J_{HH}$ = 7.8 Hz, 2H, H$_c$), 7.39 (d, $^3J_{HH}$ = 7.8 Hz, 2H, H$_d$), 7.16 (d, $^3J_{HH}$ = 7.8 Hz, 2H, H$_b$), 1.91 (s, 6H, H$_f$), 1.33 (s, 9H, H$_e$) ppm. **^{13}C{^{1}H} NMR** (150.72 MHz; $CDCl_3$, 25 ^{0}C): δ_C 190.7, 151.7, 136.7, 131.9, 131.3, 131.1, 125.3, 122.7, 120.0, 114.7, 90.2, 88.2, 34.8, 31.1, 24.1 ppm. **MS-ASAP**: m/z 333.2 [M+H]$^+$. **Elem. Anal.** for $C_{23}H_{24}O_2\cdot½CH_2Cl_2$: C, 79.01; H, 6.99 %. **Found:** C, 79.01; H, 6.84 %.

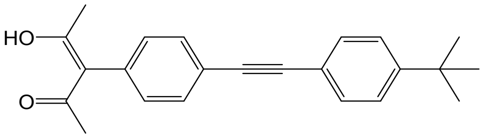

3-(4′-((4″-((4‴-(tert-butyl)phenyl)ethynyl)phenyl)ethynyl)phenyl)-4-hydroxypent-3-en-2-one (**L**^{3}H). White solid. **Yield**: 308 mg (43%). **^{1}H NMR** (599.42 MHz; $CDCl_3$, 25 ^{0}C): δ_H 7.55 (d, $^3J_{HH}$ = 7.8 Hz, 2H, H$_a$), 7.52-7.49 (m, 4H, H$_c$+H$_d$), 7.46 (d, $^3J_{HH}$ = 7.8 Hz, 2H, H$_e$), 7.37 (d, $^3J_{HH}$ = 7.8 Hz, 2H, H$_f$), 7.17 (d, $^3J_{HH}$ = 7.8 Hz, 2H, H$_b$), 1.90 (s, 6H, H$_h$), 1.32 (s, 9H, H$_g$) ppm. **^{13}C{^{1}H} NMR** (150.72 MHz; $CDCl_3$, 25 ^{0}C): δ_C 190.7, 151.8, 137.1, 132.0, 131.4, 131.3, 131.1, 123.5, 122.6, 122.3, 119.9, 114.6, 91.5, 90.5, 89.7, 88.3, 34.8, 31.1, 24.1 ppm. **MS-ASAP**: m/z 433.2 [M+H]$^+$. **Elem. Anal.** for $C_{31}H_{28}O_2\cdot¼CH_2Cl_2$: C, 82.71; H, 6.15 %. **Found**: C, 82.56; H, 6.15 %.

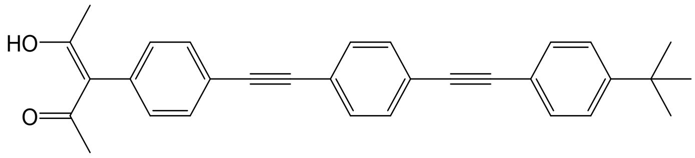

Substituted acac ligand complex general synthesis (Complexes 1-5)

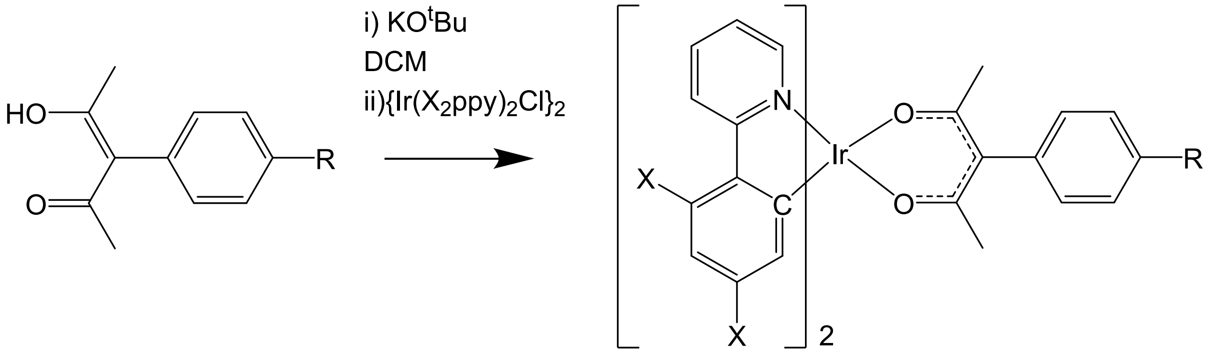

L[1-3]H (1 eq) was dissolved in DCM and the solution was cooled to 0°C before potassium *tert*-butoxide (1 eq) was added. The solution was stirred for two hours before {Ir(ppy)$_2$Cl}$_2$ or {Ir(F$_2$ppy)$_2$Cl}$_2$ (0.5 eq) was added, the temperature was maintained at 0°C before being allowed to warm to room temperature and the stirring continued for an additional 2 hours before the solution was poured into a saturated ammonium chloride solution, the organic layer was extracted, dried over $MgSO_4$ and the solvent removed under vacuo. The yellow solid was sonicated in methanol and the solid was collected by filtration to yield a yellow powder. Purification was achieved by crystallisation using slow evaporation of a DCM/MeOH solution. Note: all procedures were performed in the absence of light or at low light levels.

Ir(ppy)$_2$(L^1) (**1**). **Yield**: 353 mg (92%) **^{1}H NMR** (599.42 MHz; TCE-d$_2$, 25 ^{0}C): δ_H 8.65 (dt, $^3J_{HH}$ = 5.5 Hz, $^4J_{HH}$ = 1.2 Hz, 2H, H$_a$), 7.89 (dt, $^3J_{HH}$ = 8.1, $^4J_{HH}$ =1.0 Hz, 2H, H$_d$), 7.82 (td, $^3J_{HH}$ =8.1, $^4J_{HH}$ =1.5 Hz, 2H, H$_c$), 7.61 (dd, $^3J_{HH}$ =7.7 Hz, $^4J_{HH}$ =1.3 Hz, 2H, H$_e$), 7.34 (t, $^3J_{HH}$ =7.5 Hz, 2H, H$_j$), 7.29-7.24 (m, 3H, H$_b$+H$_k$), 7.15 (d, $^3J_{HH}$ = 7.4 Hz, 2H, H$_i$), 6.92 (td, $^3J_{HH}$ = 7.4 Hz, $^4J_{HH}$ =1.2 Hz, 2H, H$_f$), 6.73 (td, $^3J_{HH}$ =7.4 Hz, $^4J_{HH}$ =1.3 Hz, 2H, H$_g$), 6.28 (dd, $^3J_{HH}$ = 7.7 Hz, 1.2 Hz, 2H, H$_h$), 1.57 (s, 6H, H$_m$) ppm. **^{13}C{^{1}H} NMR** (150.72 MHz; TCE-d$_2$, 25 ^{0}C): δ_C 183.9, 168.2, 148.2, 147.8, 145.4, 143.0, 137.3, 133.7, 132.2, 128.6, 126.4, 124.0, 121.8,

120.5, 118.6, 115.9, 67.3, 29.7 ppm. **MS-MALDI**: m/z 676.2 $[M]^+$. **Elem. Anal.** for $C_{33}H_{27}IrN_2O_2$: C, 58.65; H, 4.03; N, 4.15 %. **Found**: C, 58.52; H, 4.02; N, 4.18 %

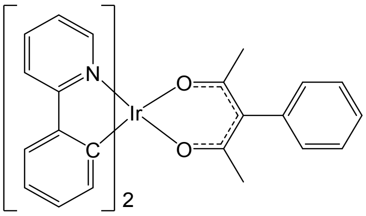

Ir(F$_2$ppy)$_2$(L^1) (**2**). **Yield**: 381 mg (90%). **^{1}H NMR** (599.42 MHz; TCE-d$_2$, 25 ^{0}C): δ_H 8.58 (d, $^3J_{HH}$ = 6.1 Hz, 2H, H_a), 8.27 (d, $^3J_{HH}$ = 8.3 Hz, 2H, H_d), 7.87 (td, $^3J_{HH}$ = 7.9, $^4J_{HH}$ = 1.6 Hz, 2H, H_c), 7.35-7.30 (m, 4H, H_h+H_i), 7.27 (t, $^3J_{HH}$ = 7.2 Hz, 1H, H_b), 7.11 (d, $^3J_{HH}$ = 8.0 Hz, 2H, H_g), 6.41 (td $^3J_{HF}$ = 9.1 Hz, $^4J_{HH}$ = 2.3 Hz, 2H, H_e), 5.70 (dd, $^3J_{HF}$ = 8.8 Hz, $^4J_{HH}$ = 2.4 Hz, 2H, H_f), 1.56 (s, 6H, H_k) ppm. **^{13}C{^{1}H} NMR** (150.72 MHz; TCE-d$_2$, 25 ^{0}C): δ_C 184.1, 165.0, 152.3, 148.1, 142.3, 138.2, 132.1, 129.0, 128.7, 126.6, 122.7, 121.9, 115.8, 115.1, 97.0, 67.4, 29.5 ppm. **^{19}F NMR** (376.57 MHz; TCE-d$_2$): δ_F -108.8 (d, J^3_{HF} = 9.8 Hz, 2F), -110.3 (d, J^3_{HF} =9.7 Hz, 2F) ppm. **MS-MALDI**: m/z. 747.8 $[M]^+$. **Elem. Anal**. for $C_{33}H_{23}F_4IrN_2O_2$: C, 53.01; H, 3.10; N, 3.75 %. **Found**: C, 52.79; H, 3.16; N, 3.73 %.

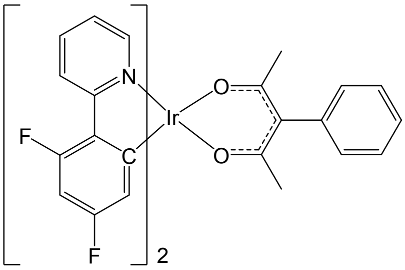

Ir(ppy)$_2$(L^2) (**3**). **Yield**: 281 mg (75%). **^{1}H NMR** (599.42 MHz; TCE-d$_2$, 25 ^{0}C): δ_H 8.63 (dt, $^3J_{HH}$ = 5.3 Hz, $^4J_{HH}$ = 1.3 Hz, 2H, H_a), 7.89 (dd, $^3J_{HH}$ = 8.3 Hz, $^4J_{HH}$ = 1.2 Hz, 2H, H_d), 7.82 (td, $^3J_{HH}$ = 8.2 Hz, $^4J_{HH}$ = 1.5 Hz, 2H, H_c), 7.61 (dd, $^3J_{HH}$ = 7.8 Hz, $^4J_{HH}$ = 1.3 Hz, 2H, H_e), 7.51 (d, $^3J_{HH}$ = 7.6 Hz, 2H, H_k), 7.49 (d, $^3J_{HH}$ = 7.6 Hz, 2H, H_j), 7.39 (d, $^3J_{HH}$ = 7.8 Hz, 2H, H_i), 7.29 (td, $^3J_{HH}$ = 7.2 Hz, $^4J_{HH}$ = 1.4 Hz, 2H, H_b), 7.14 (d, $^3J_{HH}$ = 8.2 Hz, 2H, H_l), 6.92 (td, $^3J_{HH}$ = 7.4 Hz, $^4J_{HH}$ = 1.2 Hz, 2H, H_f), 6.73 (td, $^3J_{HH}$ = 7.4 Hz, $^4J_{HH}$ = 1.3 Hz, 2H, H_g), 6.27 (dd, $^3J_{HH}$ = 7.7 Hz, $^3J_{HH}$ = 1.2 Hz, 2H, H_h), 1.59 (s, 6H, H_n), 1.33 (s, 9H, H_m) ppm. **^{13}C{^{1}H} NMR** (150.72 MHz; TCE-d$_2$, 25 ^{0}C): δ_C 183.7, 168.2, 150.7, 148.2, 145.4, 137.3, 133.6,

132.3, 132.0, 131.4, 128.7, 125.5, 31.2, 29.7 ppm (not all ^{13}C signals observed due to low solubility). **MS-MALDI**: m/z 832.2 $[M]^+$. **Elem. Anal.** for $C_{45}H_{39}IrN_2O_2\cdot\frac{1}{4}CH_2Cl_2$: C, 63.72; H, 4.67; N, 3.28 %. **Found**: C, 63.72; H, 4.61; N, 3.30 %.

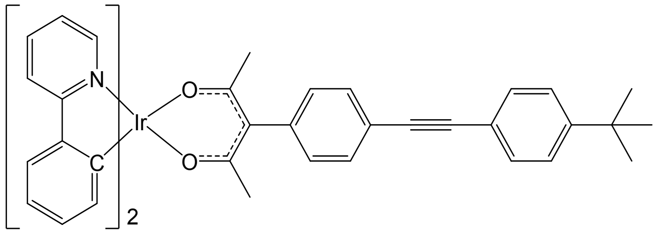

Ir(F₂ppy)₂(L²) (**4**). **Yield**: 343 mg (76 %). **^{1}H NMR** (599.42 MHz; TCE-d$_2$, 25 ^{0}C): δ_H 8.57 (d, $^3J_{HH}$ = 5.1 Hz, 2H, H_a), 8.28 (d, $^3J_{HH}$ = 8.4 Hz, 2H, H_d), 7.88 (td, $^3J_{HH}$ = 7.9 Hz, $^3J_{HH}$ = 7.6 Hz, $^4J_{HH}$ =1.6 Hz, 2H, H_c), 7.51 (d, $^3J_{HH}$ = 8.2 Hz, 2H, H_h), 7.49 (d, $^3J_{HH}$ = 8.2 Hz, 2H, H_j), 7.39 (d, $^3J_{HH}$ = 8.8 Hz, 2H, H_g), 7.32 (d, $^3J_{HH}$ = 8.3 Hz, 2H) 7.11 (d, $^3J_{HH}$ = 8.3 Hz, 2H, H_j), 6.42 (dd, $^3J_{HF}$ = 11.8 Hz, $^3J_{HF}$ = 9.2 Hz, $^4J_{HH}$ = 2.5 Hz, 2H, H_e), 5.71 (dd, $^3J_{HF}$ = 8.8 Hz, $^4J_{HH}$ =2.4 Hz, 2H, H_f), 1.58 (s, 6H, H_l), 1.33 (s, 9H, H_k) ppm. **^{13}C{^{1}H} NMR** (150.72 MHz; TCE-d$_2$, 25 ^{0}C): δ_C 183.9, 165.0, 161.6, 152.2, 151.8, 148.0, 142.7, 138.2, 132.2, 131.4, 129.0, 125.5, 122.7, 121.9, 121.4, 120.0, 97.11, 89.7, 88.9, 34.8, 31.2, 29.5 ppm. **^{19}F NMR** (376.57 MHz; TCE-d$_2$): δ_F -108.7 (d $^3J_{FH}$ =9.8 Hz, 2F), -110.3 (d, $^3J_{FH}$ = 9.8 Hz, 2F) ppm. **MS-MALDI**: m/z 904.2 $[M]^+$. **Elem. Anal.** for $C_{45}H_{35}F_4IrN_2O_2\cdot\frac{1}{4}CH_2Cl_2$: C, 58.74; H, 3.87; N, 3.03 %. **Found**: C, 58.64; H, 3.84; N, 2.98 %.

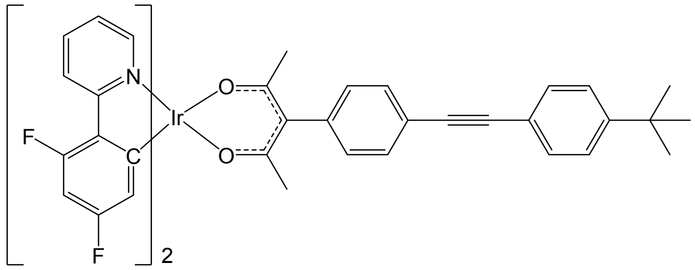

Ir(F₂ppy)₂(L³) (**5**). **Yield**: 143 mg (31%). **^{1}H NMR** (599.42 MHz; TCE-d$_2$, 25 ^{0}C): δ_H 8.56 (dd, J^3_{HH} = 5.6 Hz, J^4_{HH} = 1.5 Hz, 2H, H_a), 8.28 (d, J^3_{HH} = 8.5 Hz, 2H, H_d), 7.88 (t, J^3_{HH} = 8.1 Hz, 2H, H_c), 7.57-7.52 (m, 6H, H_i+H_j+H_k), 7.50 (d, J^3_{HH} = 8.1 Hz, 2H, H_h), 7.40 (d, J^3_{HH} = 8.1 Hz, 2H, H_g), 7.32 (td, J^3_{HH} = 7.2 Hz, J^4_{HH} = 1.3 Hz, 2H, H_b), 7.13 (d, J^3_{HH} = 8.1 Hz, 2H, H_l), 6.41 (td, J^3_{HF} = 9.1 Hz, J^4_{HH} = 2.4 Hz, 2H, H_e), 5.70 (dd, J^3_{HF} = 8.8 Hz, J^4_{HH} = 2.3 Hz,

2H, H_f), 1.58 (s, 6H, H_n), 1.33 (s, 9H, H_m) ppm. **$^{13}C\{^1H\}$ NMR** (150.72 MHz; TCE-d_2, 25 ^0C): δ_C 183.8, 165.0, 161.4, 152.1, 148.0, 138.2, 132.5, 132.3, 131.5, 125.5, 122.8, 121.9, 121.0, 119.7, 115.2, 97.1, 91.4, 89.4, 34.8, 31.2, 29.5 ppm (not all ^{13}C signals observed due to low solubility). **^{19}F NMR** (376.57 MHz; TCE-d_2): δ_F -108.7 (d, J^3_{FH} = 9.9 Hz, 2F), -110.2 (d, J^3_{FH} =9.8 Hz, 2F) ppm. **MS-MALDI**: m/z 1003.8 $[M]^+$. **Elem. Anal.** for $C_{53}H_{39}F_4IrN_2O_2{\cdot}CH_2Cl_2$: C, 59.61; H, 3.71; N, 2.57 %. **Found**: C, 59.94; H, 3.68; N, 2.50 %.

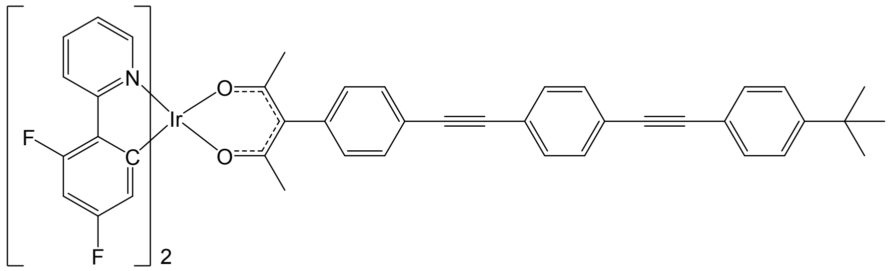

Triisopropylsilyl acetylene Sonogashira coupling of PPy based ligands general method (**L**^{5}H, **L**^{6}H, **L**^{8}H, and **L**^{9}H)

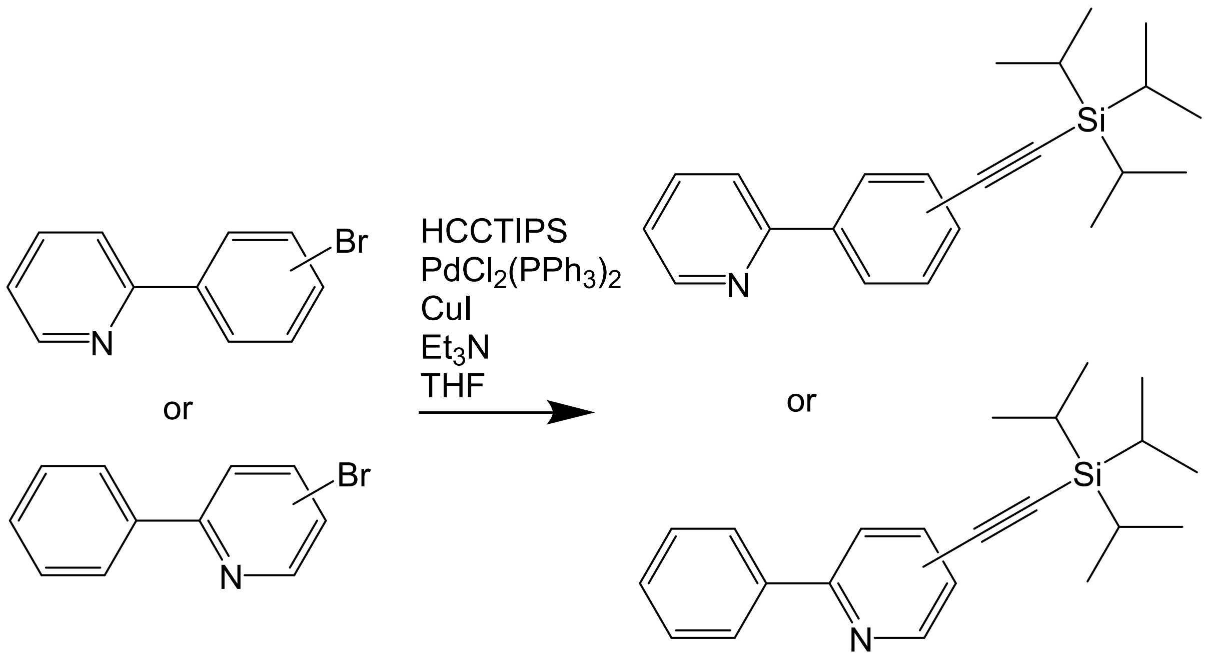

THF (dry, 50 mL) and Et_3N (15 mL) were added to bromo substituted ppy (2.17 mmol), $Pd(PPh_3)_2Cl_2$ (150 mg, 0.21 mmol) and CuI (40 mg, 0.21 mmol). The solution was degassed by three freeze-pump-thaw cycles before triisopropylsilyl acetylene (0.49 mL, 0.40 g, 2.2

mmol) was added. The solution was heated to reflux for 16 hours before the solvent was removed. The product was purified via column chromatography on silica gel eluted with a solvent gradient from neat hexane to neat DCM.

2-(3'-((triisopropylsilyl)ethynyl)phenyl)pyridine (**L**^{5}H). Producing and yellow oil. **Yield**: 612 mg (84%). **^{1}H NMR** (599.42 MHz; CDCl$_3$, 25 ^{0}C): δ_H 8.69 (dd, ^{3}J = 4.9 Hz, ^{4}J = 1.8 Hz, 1H, H$_a$), 8.09 (t, $^{4}J_{HH}$ = 1.7 Hz, 1H, H$_e$), 7.92 (dt, $^{3}J_{HH}$ = 7.8 Hz, $^{4}J_{HH}$ = 1.5 Hz, 1H, H$_f$), 7.75 (td, $^{3}J_{HH}$ = 7.6 Hz, $^{4}J_{HH}$ = 1.8 Hz, 1H, H$_c$), 7.72 (dt, $^{3}J_{HH}$ = 8.0 Hz, $^{4}J_{HH}$ = 1.2 Hz, 1H, H$_d$), 7.52 (dt, $^{3}J_{HH}$ = 7.6 Hz, $^{4}J_{HH}$ = 1.4 Hz, 1H, H$_h$), 7.40 (t, $^{3}J_{HH}$ = 7.7 Hz, 1H, H$_g$), 7.24 (ddd, $^{3}J_{HH}$ = 7.3 Hz, $^{3}J_{HH}$ = 4.8 Hz, $^{4}J_{HH}$ = 1.3 Hz, 1H, H$_b$), 1.14 (pseudo singlet, 21H, H$_i$+H$_j$) ppm. **^{13}C{^{1}H} NMR** (150.72 MHz; CDCl$_3$, 25 ^{0}C): δ_C 156.6, 149.7, 139.4, 136.7, 132.5, 130.3, 128.6, 126.8, 124.0, 122.3, 120.6, 106.8, 90.7, 18.6, 11.3 ppm. **MS-ASAP**: m/z 336.203 [M+H]$^{+}$. **Elem. Anal.** for $C_{22}H_{29}N_{Si}$: C, 78.75; H, 8.71; N, 4.17 %. **Found**: C, 78.54; H, 8.64; N, 5.36 %.

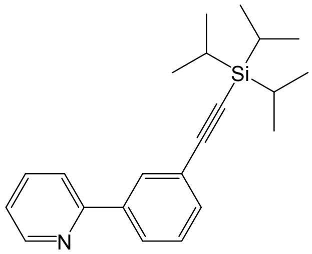

2-phenyl-5-((triisopropylsilyl)ethynyl)pyridine (**L**^{6}H). Producing a white solid. **Yield**: 654 mg (90%). **^{1}H NMR** (599.42 MHz; CDCl$_3$, 25 ^{0}C): δ_H 8.77 (d, $^{4}J_{HH}$ = 2.1 Hz, 1H, H$_a$), 8.00 (d, $^{3}J_{HH}$ = 7.8 Hz, 2H, H$_d$), 7.80 (dd, $^{3}J_{HH}$ = 8.2 Hz, $^{4}J_{HH}$ = 2.2 Hz, 1H, H$_b$), 7.68 (dd, $^{3}J_{HH}$ = 8.2 Hz, $^{4}J_{HH}$ = 0.9 Hz, 1H, H$_c$), 7.48 (t, $^{3}J_{HH}$ = 7.8 Hz, 2H, H$_e$), 7.43-7.41 (m, 1H, H$_f$), 1.16 (pseudo singlet, 21H, H$_g$+H$_h$) ppm. **^{13}C{^{1}H} NMR** (150.72 MHz; CDCl$_3$, 25 ^{0}C): δ_C 156.1, 152.6, 139.6, 138.6, 129.2, 128.8, 126.9, 119.5, 118.8, 103.7, 95.1, 18.6, 11.2 ppm. **MS-ASAP**: m/z 336.195 [M+H]$^{+}$. **Elem. Anal.** for $C_{22}H_{29}N_{Si}$: C, 78.75; H, 8.71; N, 4.17 %. **Found**: C, 78.46; H, 8.81; N, 3.89 %.

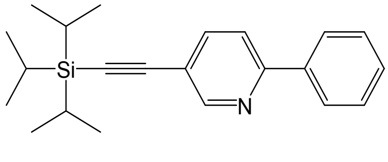

2-(2'',3'',5'',6''-tetramethyl-4''-((triisopropylsilyl)ethynyl)-[1',1''-biphenyl]-4-yl)pyridine (**L**^{8}H)

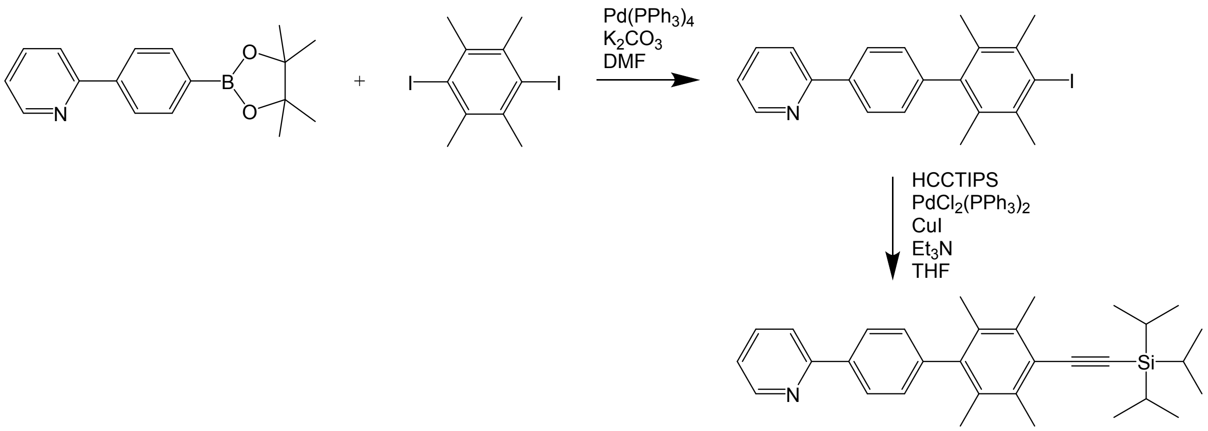

DMF (dry, 50mL) was added to 2-(4-(4,4,5,5-tetramethyl-1,3,2-dioxaborolan-2-yl)phenyl)pyridine (1.0 g, 3.55 mmol), 1,4-diiodo-2,3,5,6-tetramethylbenzene (4.09 g, 10.6 mmol) and K_2CO_3 (0.98 g, 7.1 mmol). The solution was degassed by three freeze-pump-thaw cycles before $Pd(PPh_3)_4$ (0.41 g, 0.35 mmol) was added. The solution was heated to 110 °C for 16 hours. Once cooled, the solution was poured into water and extracted with dichloromethane (DCM). The organic layer was collected and dried over magnesium sulfate before the solvent was removed. The product was purified via column chromatography on silica gel eluted with a solvent gradient from neat hexane to neat DCM. Final purification achieved by crystallisation from the evapouration of a DCM/methanol solution to give white crystals. Yielding 2-(4″-iodo-2″,3″,5″,6″-tetramethyl-[1′,1″-biphenyl]-4′-yl)pyridine*:* 0.99 g (68 %). THF (dry, 50 mL) and Et_3N (15 mL) were added to 2-(4″-iodo-2″,3″,5″,6″-tetramethyl-[1′,1″-biphenyl]-4′-yl)pyridine (0.99 g, 2.39 mmol), $Pd(PPh_3)_2Cl_2$ (161 mg, 0.23 mmol) and CuI (43 mg, 0.23 mmol). The solution was degassed by three freeze-pump-thaw cycles before triisopropylsilyl acetylene (0.56 mL, 0.46 g, 2.5 mmol) was added. The solution was heated to reflux for 16 hours before the solvent was removed. The product was purified via column chromatography on silica gel eluted with a solvent gradient from neat hexane to neat DCM. Final purification achieved by crystallisation from the evaporation of a DCM/methanol solution to give white crystals. **Yield**: 1.10 g (99 %). **^{1}H NMR** (599.42 MHz; $CDCl_3$, 25 ^{0}C): δ_H 8.72 (dt, $^{3}J_{HH}$ = 4.7 Hz, $^{4}J_{HH}$ = 1.4 Hz, 1H, H_a), 8.05 (d, $^{3}J_{HH}$ = 8.0 Hz, 2H, H_e), 7.80-7.76 (m, 2H, H_e+H_d), 7.26-7.24 (m, 1H, H_b), 7.20 (d, $^{3}J_{HH}$ = 8.0 Hz, 2H, H_f), 2.51 (s, 6H, H_g), 1.95 (s, 6H, H_h), 1.17 (pseudo singlet, 21H, H_i+H_j) ppm. **^{13}C{^{1}H} NMR** (150.72 MHz; $CDCl_3$, 25 ^{0}C): δ_C 157.3, 149.7, 143.1, 141.7, 137.7, 136.7, 136.3, 131.8, 129.6, 126.9, 123.0, 122.0, 120.4, 105.8, 98.3, 18.7, 18.5, 17.9, 11.4 ppm. **MS-ASAP**: m/z 468.276

[M+H]$^+$. **Elem. Anal.** for $C_{32}H_{41}NSi$: C, 82.17; H, 8.84; N, 2.99 %. **Found**: C, 81.93; H, 8.79; N, 3.04 %.

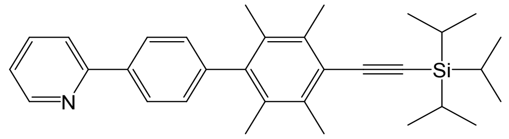

2-phenyl-5-(2′,3′,5′,6′-tetramethyl-4′-((triisopropylsilyl)ethynyl)phenyl)pyridine (**L**^{9}H)

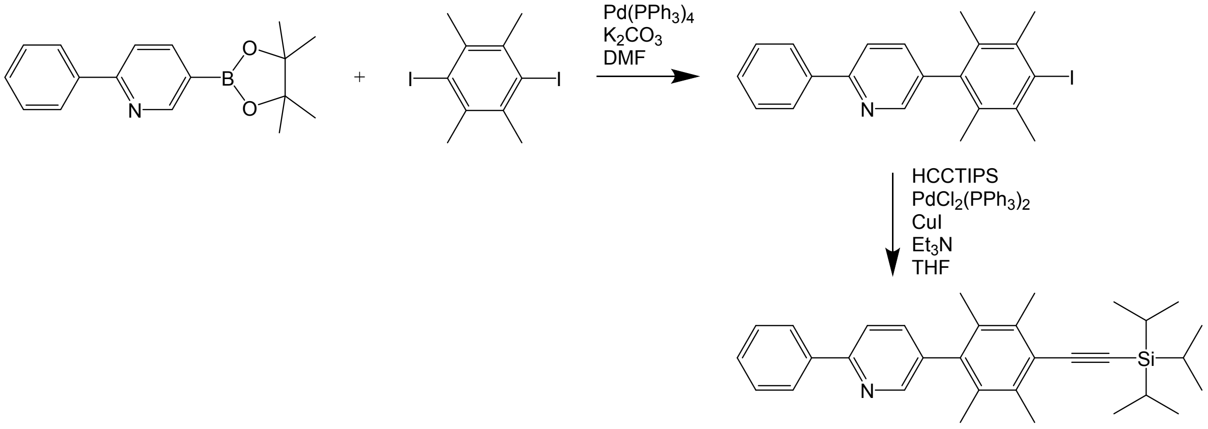

Using the same procedure as per **L**^{8}H except 2-phenyl-5-(4′,4′,5′,5′-tetramethyl-1′,3′,2′-dioxaborolan-2′-yl)pyridine was used in place of 2-(4′-(4′,4′,5′,5′-tetramethyl-1′,3′,2′-dioxaborolan-2′-yl)phenyl)pyridine. Final purification achieved by crystallisation from the evapouration of a DCM/methanol solution to give white crystals. **Overall Yield**: 435 mg (43%). **^{1}H NMR** (599.42 MHz; $CDCl_3$, 25 ^{0}C): δ_H 8.45 (d, $^4J_{HH}$ = 2.2 Hz, 1H, H$_a$), 8.08 (d, $^3J_{HH}$ = 8.1 Hz, 2H, H$_d$), 7.83 (dd, $^3J_{HH}$ = 8.1 Hz, $^4J_{HH}$ = 0.9 Hz, 1H, H$_c$), 7.52-7.49 (m, 3H, H$_b$+H$_e$), 7.44 (t, $^3J_{HH}$ = 7.9 Hz, 1H, H$_f$), 2.51 (s, 6H, Hg), 1.96 (s, 6H, H$_h$), 1.17 (pseudo singlet, 21H, H$_i$+H$_j$) ppm. **^{13}C{^{1}H} NMR** (150.72 MHz; $CDCl_3$, 25 ^{0}C): δ_C 155.5, 149.9, 139.0, 138.0, 137.6, 136.5, 136.2, 132.2, 129.0, 128.8, 126.7, 123.8, 120.0, 105.4, 98.9, 18.7, 18.6, 18.0, 11.3 ppm. **MS-ASAP**: m/z 468.289 [M+H]$^+$. **Elem. Anal.** For $C_{32}H_{41}NSi\cdot\frac{1}{4}H_2O$: C, 81.38; H, 8.86; N, 2.97 %. **Found**: C,81.48; H, 8.77; N, 2.97 %.

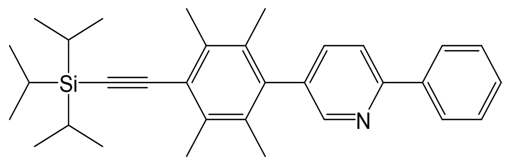

Ir(TIPS-ethynyl-ppy)$_2$(acac) complex general synthesis (complexes **6-8**, **10** and **11**)

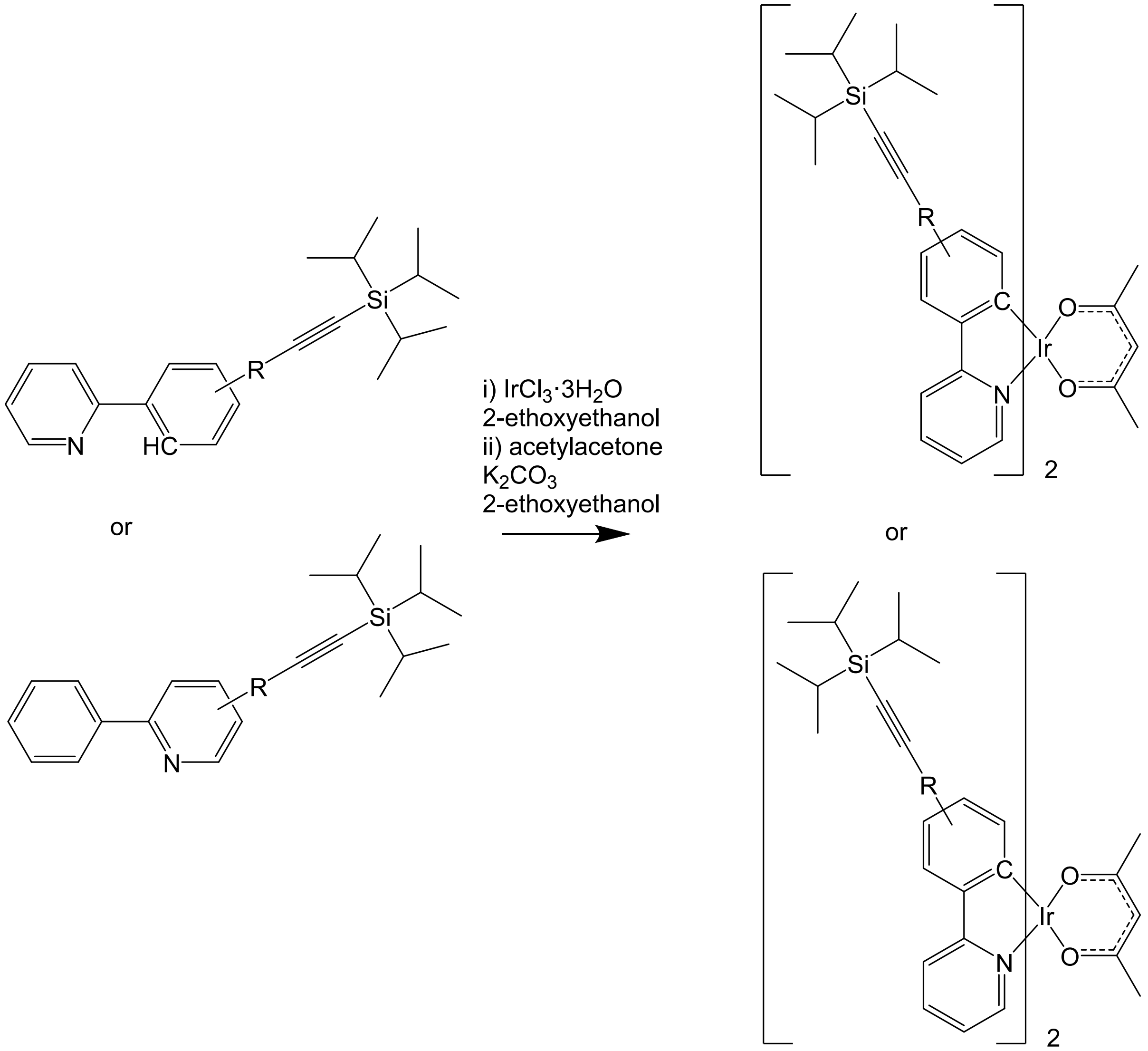

$IrCl_3$·3H_2O (523 mg, 1.49 mmol) was added to a solution containing **L**^{n}H (2.98 mmol), ethoxyethanol (30 mL) and water (15 mL). The solution was heated to 110 °C for 12 hours before being cooled and poured into water (300 mL), forming a red/orange/green precipitate. The precipitate was dissolved in DCM and dried over $MgSO_4$ before being passed through a silica plug. The green band was collected and the solvent removed. The orange residue was dissolved in ethoxyethanol (20 mL), acetylacetone (5 mL) and K_2CO_3 (276 mg, 2.0 mmol) were added and the solution heated to 90 °C for 12 hours. The solution was cooled and poured into water (300 mL), forming an orange/green precipitate that was collected via filtration. The precipitate was dissolved in DCM and dried over $MgSO_4$ before the product was purified via silica chromatography eluted by DCM, collecting the emissive band. The

solvent was removed, leaving a bright orange/green solid. Crystals were grown by evaporation of a DCM/MeOH solution.

Ir(L⁴)₂(acac) (**6**). Bright orange solid. **Yield**: 1.30 g (87 %). **^{1}H NMR** (599.42 MHz; $CDCl_3$, 25 ^{0}C): δ_H 8.49 (dd, $^3J_{HH}$ = 5.69 Hz, $^4J_{HH}$ = 1.63 Hz, 2H, H_a), 7.81 (dt, $^3J_{HH}$ = 8.26 Hz, $^3J_{HH}$ = 1.14 Hz, 2H, H_d), 7.71 (ddd, $^3J_{HH}$ = 8.2 Hz, $^3J_{HH}$ =7.4 Hz, $^4J_{HH}$ =1.6 Hz, 2H, H_c), 7.45 (d, $^3J_{HH}$ = 8.0 Hz, 2H, H_e), 7.14 (ddd, $^3J_{HH}$ = 7.27 Hz, $^3J_{HH}$ = 5.64 Hz, $^4J_{HH}$ = 1.41 Hz, 2H, H_b), 6.92 (dd, $^3J_{HH}$ = 8.0, 1.6 Hz, 2H, H_f), 6.26 (d, $^4J_{HH}$ = 1.56 Hz, 2H, H_g), 5.20 (s, 1H, H_j), 1.77 (s, 6H, H_k), 1.01 (pseudo singlet, 42H, H_h+H_i) ppm. **^{13}C{^{1}H} NMR** (150.72 MHz; $CDCl_3$, 25 ^{0}C): δ_C 184.6, 167.7, 148.2, 146.0, 145.2, 136.7, 136.3, 124.6, 123.4, 123.0, 121.7, 118.8, 108.8, 100.3, 90.3, 28.6, 18.6, 11.3 ppm. **MS-MALDI**: m/z 1003.8 $[M]^+$. **Elem. Anal.** for $C_{49}H_{63}IrN_2O_2Si_2$: C, 61.28; H, 6.61; N, 2.92 %. **Found**: C, 61.23; H, 6.55; N, 2.88 %.

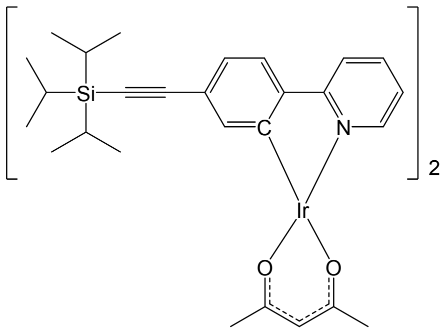

Ir(L⁵)₂(acac) (**7**). Bright orange solid. **Yield**: 1.13 g (76%). **^{1}H NMR** (599.42 MHz; $CDCl_3$, 25 ^{0}C): δ_H 8.47 (dd, $^3J_{HH}$ = 5.7 Hz, $^4J_{HH}$ J = 1.6 Hz, 2H, H_a), 7.92 (dt, $^3J_{HH}$ = 8.3 Hz, $^4J_{HH}$ = 1.1 Hz, 2H, H_d), 7.82 (ddd, $^3J_{HH}$ = 8.2 Hz, $^3J_{HH}$ = 7.5 Hz, $^4J_{HH}$ = 1.6 Hz, 2H, H_c), 7.67 (d, $^4J_{HH}$ = 1.7 Hz, 2H, H_e), 7.24 (ddd, $^3J_{HH}$ = 7.3 Hz, $^3J_{HH}$ = 5.6 Hz, $^4J_{HH}$ = 1.4 Hz, 2H, H_b), 6.76 (dd, $^3J_{HH}$ = 7.9 Hz, $^4J_{HH}$ = 1.7 Hz, H_f), 6.19 (d, $^3J_{HH}$ = 7.9 Hz, 2H, H_g), 5.29 (s, 1H, H_j), 1.80 (s, 6H, H_k), 1.10 (pseudo singlet, 42H, H_h+H_i) ppm. **^{13}C{^{1}H} NMR** (150.72 MHz; $CDCl_3$, 25 ^{0}C): δ_C 184.9, 167.2, 149.8, 145.2, 137.4, 132.9, 131.8, 126.7, 122.2, 118.8, 115.6, 108.3, 100.3, 87.8, 28.2, 18.3, 11.3 ppm. **MS-MALDI**: m/z 960.2 $[M]^+$. **Elem. Anal.** for $C_{49}H_{63}IrN_2O_2Si_2$: C, 61.28; H, 6.61; N, 2.92 %. **Found**: C, 61.25; H, 6.50; N, 2.88 %.

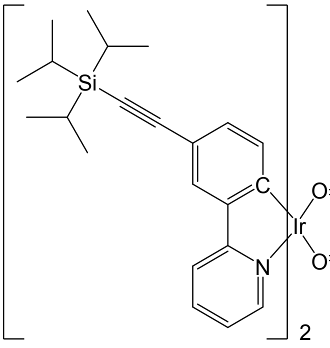

Ir(L⁶)₂(acac) (**8**). Bright orange solid. **Yield**: 1.15 g (78%). **^{1}H NMR** (599.42 MHz; $CDCl_3$, 25 ^{0}C): δ_H 8.57 (d, $^4J_{HH}$ = 2.0 Hz, 2H, H_a), 7.76-7.73 (m, 4H, H_b+H_c), 7.51 (dd, $^3J_{HH}$ = 7.7 Hz, $^4J_{HH}$ = 1.3 Hz, 2H. H_d), 6.81 (td, $^3J_{HH}$ = 7.8 Hz, $^4J_{HH}$ = 1.2 Hz, 2H, H_e), 6.71 (td, $^3J_{HH}$ = 7.8 Hz, $^4J_{HH}$ = 1.2 Hz, 2H, H_f), 6.28 (dd, $^3J_{HH}$ = 7.7 Hz, $^4J_{HH}$ = 1.2 Hz, 2H, H_g), 5.23 (s, 1H, H_j), 1.79 (s, 6H, H_k), 1.13 (pseudo singlet, 42H, H_i+H_h) ppm. **^{13}C{^{1}H} NMR** (150.72 MHz; $CDCl_3$, 25 ^{0}C): δ_C 184.8, 167.9, 151.1, 148.4, 143.9, 133.2, 129.6, 124.3, 120.9, 117.7, 117.5, 102.7, 95.3, 28.6, 18.6, 11.2 ppm. **MS-MALDI**: m/z 960.3 $[M]^+$. **Elem. Anal.** for $C_{49}H_{63}IrN_2O_2Si_2$: C, 61.28; H, 6.61; N, 2.92 %. **Found**: C, 61.32; H, 6.53; N, 2.83 %.

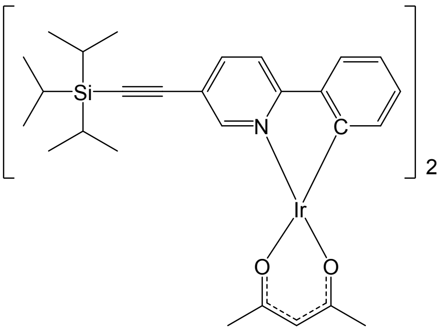

Ir(L⁸)₂(acac) (**10**). Green solid. **Yield**: 0.89 g (73 %). ^{1}**H NMR** (599.42 MHz; $CDCl_3$, 25 ^{0}C): δ_H 8.42 (dd, $^3J_{HH}$ = 5.7 Hz, $^4J_{HH}$ =1.6 Hz, 2H, H_a), 7.77 (d, $^3J_{HH}$ = 8.1 Hz, 2H, H_d), 7.63 (t, $^3J_{HH}$ = 7.7 Hz, 2H, H_c), 7.51 (d, $^3J_{HH}$ = 7.8 Hz, 2H, H_e), 7.03 (ddd, $^3J_{HH}$ = 7.3, $^3J_{HH}$ = 5.7 Hz, $^4J_{HH}$ = 1.4 Hz, 2H, H_b), 6.45 (dd, $^3J_{HH}$ = 7.8 Hz, $^4J_{HH}$ = 1.6 Hz, 2H, H_f), 5.95 (d, $^3J_{HH}$ = 1.6 Hz, 2H, H_g), 5.26 (s, 1H, H_n), 2.40 (s, 6H, H_h or H_i), 2.34 (s, 6H, H_h or H_i), 1.96 (s, 6H, H_j or H_k), 1.81 (s, 6H, H_o), 1.35 (s, 6H, H_j or H_k), 1.12 (pseudo singlet, 42H, H_l+H_m) ppm. **^{13}C{^{1}H} NMR** (150.72 MHz; $CDCl_3$, 25 ^{0}C): δ_C 184.4, 168.3, 148.1, 146.9, 143.3, 142.9, 142.2, 136.8, 135.7, 133.7, 132.2, 131.6, 123.3, 122.0, 121.4, 121.0, 118.1, 105.9, 100.3,

97.7, 28.7, 18.7, 18.4, 18.1, 11.3 ppm. **MS-MALDI**: m/z 1224.0 $[M]^+$. **Elem. Anal.** for $C_{69}H_{87}IrN_2O_2Si_2$: C, 67.61; H, 7.24; N, 2.29 %. **Found**: C, 67.39; H, 7.05; N, 2.25 %.

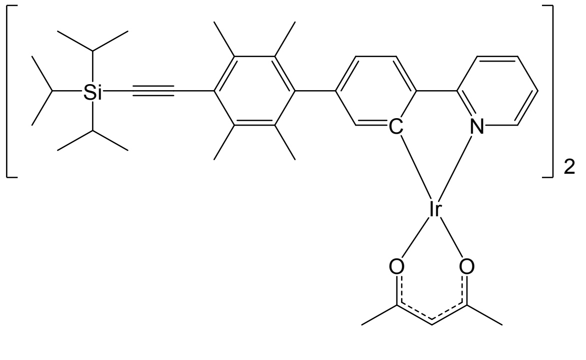

Ir(L⁹)₂(acac) (11). The solvent was removed, leaving a bright green solid. **Yield**: 400 mg (33%).[1] **MS(MALDI)**: m/z 1224.5 $[M]^+$.

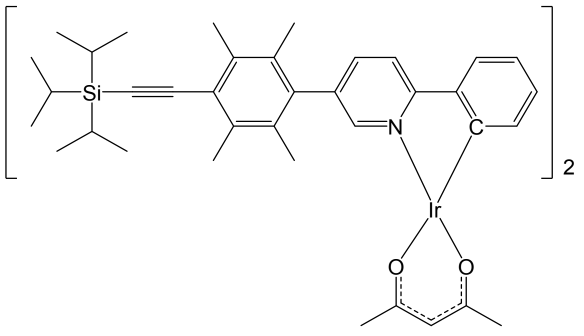

Ir(L¹⁰)(ppy)(acac) **(13)**.

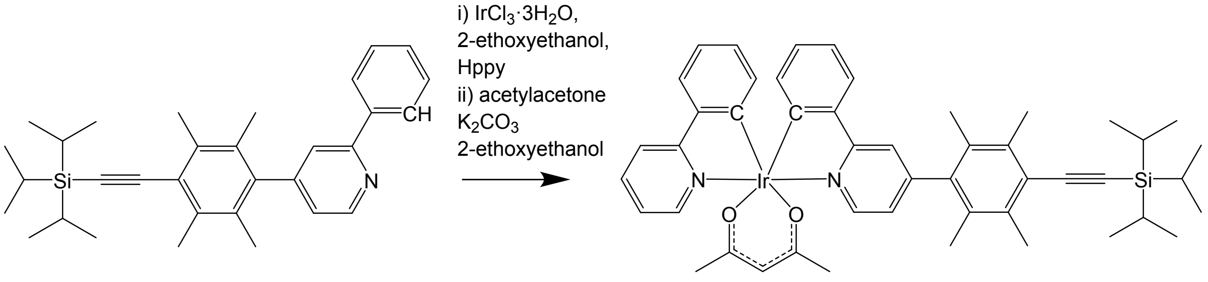

L¹⁰H (500 mg, 1.07 mmol), 2-phenylpyridine (0.15 mL, 165 mg, 1.07 mmol), and $IrCl_3{\cdot}3H_2O$ (376 mg, 1.07 mmol), were suspended in a mixture of 2-ethoxyethanol (24 mL) and water (8 mL), and heated at 110°C for 16 hours. During this time a yellow-green

[1] Could only be isolated with purity of ca 90%, therefore full characterisation was not possible, as such **11** was only used as an intermediate.

precipitate formed. The solution was allowed to cool to room temperature, and water (50 mL) was added, precipitating a bright yellow-green solid, which was filtered, washed with water and dissolved in DCM. The solution was washed with water, separated, dried over $MgSO_4$ and passed through a short silica plug eluted by DCM. The yellow-green band was collected, and solvent removed. The dimer mixture was dissolved in 2-ethoxyethanol (20 mL), acetylacetone (0.30 mL, 300 mg, 3.00 mmol) and K_2CO_3 (414 mg, 3.00 mmol) were added and the suspension heated to 90°C for 6 hours. The solution was allowed to cool to room temperature, and water (30 mL) was added, precipitating a bright green solid, which was filtered, washed with water, and dissolved in DCM. The solution was washed with water, separated, dried over $MgSO_4$. The residue was purified by silica chromatography eluted by a solvent gradient from DCM:hexane (1:1) to neat DCM), yielding the compound Ir($\mathbf{L^{10}}$)$_2$(acac) as the fore-running fraction (288 mg, 21%), which appeared as a bright yellow-green solid. The second fraction yielded the title product, also appearing as a bright yellow-green solid. **Yield**: 214 mg (22%). **^{1}H NMR** (599.42 MHz; $CDCl_3$, 25 ^{0}C): δ_H 8.55 (d, $^3J_{HH}$ = 5.67 Hz, 2H, H_a+H_h), 7.85 (d, $^3J_{HH}$ = 8.1 Hz, 1H, H_k), 7.73 (t, $^3J_{HH}$ = 7.9 Hz, 1H, H_j), 7.59 (s, 1H, H_c), 7.55 (d, $^3J_{HH}$ = 7.7 Hz, 1H, H_l), 7.47 (d, $^3J_{HH}$ = 7.6 Hz, 1H, H_d), 7.15 (t, $^3J_{HH}$ = 6.6 Hz, 1H, H_i), 6.92 (d, $^3J_{HH}$ = 5.8 Hz, 1H, H_b), 6.82 (t, $^3J_{HH}$ = 7.4 Hz, 1H, H_m), 6.78 (t, $^3J_{HH}$ = 7.4 Hz, 1H, H_e), 6.73 (t, $^3J_{HH}$ = 7.4 Hz, 1H, H_n), 6.69 (t, $^3J_{HH}$ = 7.4 Hz, 1H, H_f), 6.34 (d, $^3J_{HH}$ = 7.7 Hz, 1H, H_o), 6.31 (d, $^3J_{HH}$ = 7.7 Hz, 1H, H_g), 5.26 (s, 1H, H_v), 2.55-2.54 (m, 6H, H_q+H_p), 2.09 (s, 3H, H_s or H_r), 2.04 (s, 3H, H_s or H_r), 1.84 (s, 3H, H_x or H_y), 1.80 (s, 3H, H_x or H_y), 1.19 (pseudo singlet, 21H, H_u+H_t) ppm. **^{13}C{^{1}H} NMR** (150.72 MHz; $CDCl_3$, 25 ^{0}C): δ_C 184.6, 168.6, 151.6, 148.1, 148.0, 147.6, 147.5, 144.7, 144.6, 139.0, 136.8, 136.7, 133.0, 132.8, 131.1, 129.1, 124.0, 123.9, 123.8, 122.5, 121.4, 120.7, 119.2, 118.3, 105.3, 100.4, 99.2, 28.8, 18.7, 18.6, 18.5, 18.0, 17.9, 11.4 ppm. **MS-MALDI**: m/z 912.2 $[M]^+$. **Elem. Anal.** for $C_{48}H_{56}IrN_2O_2Si\cdot\frac{3}{4}H_2O$: C, 62.21; H, 6.25; N, 3.02 %. **Found**: C, 62.20; H, 5.96; N, 2.89 %.

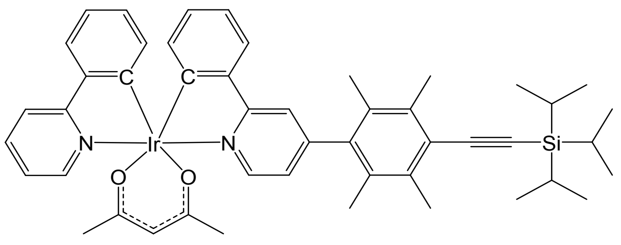

Sonogashira coupling general method

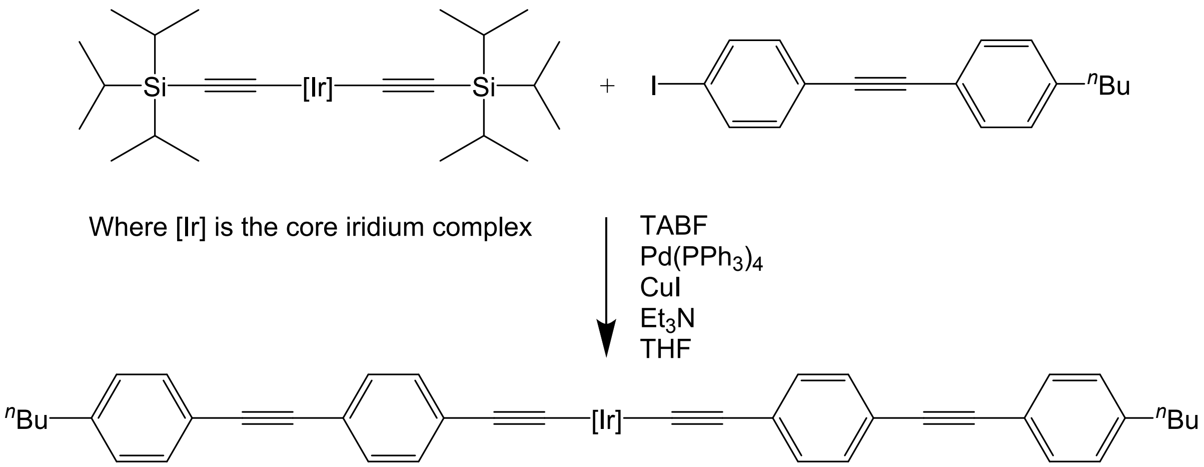

THF (50 mL) and Et_3N (5 mL) were added to the complex (**6 - 8**, **10**, and **11**) (1eq), 1-butyl-4-((4-iodophenyl)ethynyl)benzene (3 eq), $Pd(PPh_3)_4$ (10 mol%) and CuI (10 mol%). The solution was degassed by three freeze-pump-thaw cycles, before tetrabutylammonium fluoride (1.0 M in THF, 3 eq) was added. The solution was stirred at room temperature for 12 hours before the solvent was removed. The residue was purified by silica chromatography eluted by DCM.

Ir(L^{11})$_2$(acac) (**14**). A bright orange powder. Crystals grown in by vapour diffusion of n-pentane into a THF solution. **Yield**: 199 mg (86%). **^{1}H NMR** (599.42 MHz; $CDCl_3$, 25 ^{0}C): δ_H 8.50 (dd, $^3J_{HH}$ = 5.3 Hz, $^4J_{HH}$ = 1.4 Hz, 2H, H$_a$), 7.86 (d, $^3J_{HH}$ = 8.1 Hz, 2H, H$_d$), 7.76 (td, $^3J_{HH}$ = 7.8 Hz, $^4J_{HH}$ =1.5 Hz, 2H, H$_c$), 7.53 (d, $^3J_{HH}$ = 8.0 Hz, 2H, H$_e$), 7.42-7.41 (m, 8H, H$_h$+H$_i$), 7.35 (d, $^3J_{HH}$ = 7.9 Hz, 4H, H$_j$), 7.18 (ddd, $^3J_{HH}$ = 7.2 Hz, $^3J_{HH}$ = 5.6 Hz, $^4J_{HH}$ =1.3 Hz, 2H, H$_b$), 7.15 (d, $^3J_{HH}$ = 7.9 Hz, 4H, H$_k$), 7.01 (dd, $^3J_{HH}$ = 7.9 Hz, $^4J_{HH}$ = 1.5 Hz, 2H, H$_f$), 6.41 (d, $^3J_{HH}$ = 1.6 Hz, 2H, H$_g$), 5.22 (s, 1H, H$_p$), 2.61 (t, $^3J_{HH}$ = 7.8 Hz, 4H, H$_l$), 1.78 (s, 6H, H$_q$), 1.59 (h, $^3J_{HH}$ = 7.4 Hz, 4H, H$_m$), 1.35 (h, $^3J_{HH}$ = 7.4 Hz, 4H, H$_n$), 0.92 (t, $^3J_{HH}$ = 7.4 Hz, 6H, H$_o$) ppm. **^{13}C{^{1}H} NMR** (150.72 MHz; $CDCl_3$, 25 ^{0}C): δ_C 184.6, 167.6, 148.2, 146.3, 145.5, 143.5, 137.0, 135.7, 131.4, 131.3, 131.2, 128.4, 124.7, 123.3, 122.8, 121.9, 120.1, 119.0, 100.3, 92.6, 91.2, 89.3, 88.5, 35.5, 33.3, 28.7, 22.2, 13.9 ppm. **MS-MALDI**: m/z 1112.3 [M]$^+$. **Elem. Anal.** for $C_{67}H_{55}IrN_2O_2 \cdot ½H_2O$: C, 71.76; H, 5.03; N, 2.50 %. **Found**: C, 71.72; H, 4.84; N, 2.44 %.

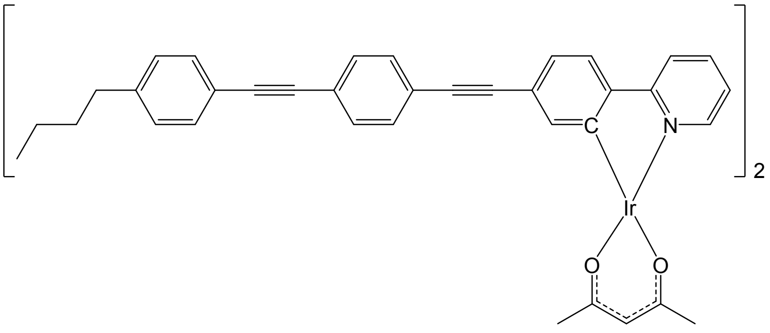

Ir(L^{12})$_2$(acac) (**15**). A bright green powder. **Yield**: 148 mg (64%). **^{1}H NMR** (599.42 MHz; $CDCl_3$, 25 ^{0}C): δ_H 8.50 (dd, $^3J_{HH}$ = 5.7 Hz, $^4J_{HH}$ = 1.6 Hz, 2H, H_a), 7.94 (dt, $^3J_{HH}$ = 8.2 Hz, $^4J_{HH}$ = 1.2 Hz, 2H, H_d), 7.85 (ddd, $^3J_{HH}$ = 8.2 Hz, $^3J_{HH}$ = 7.5 Hz, $^4J_{HH}$ = 1.6 Hz, 2H, H_c), 7.76 (d, $^4J_{HH}$ = 1.8 Hz, 2H, H_e), 7.46 (pseudo singlet, 8H, H_h+H_i), 7.43 (d, $^3J_{HH}$ = 8.0 Hz, 4H, H_j), 7.26 (ddd, $^3J_{HH}$ = 7.3 Hz, $^3J_{HH}$ = 5.7 Hz, $^4J_{HH}$ = 1.4 Hz, 2H, H_b), 7.17 (d, $^3J_{HH}$ = 8.0 Hz, 4H, H_k), 6.84 (dd, $^3J_{HH}$ = 7.9 Hz, $^4J_{HH}$ = 1.8 Hz, 2H, H_f), 6.28 (d, $^3J_{HH}$ = 7.8 Hz, 2H, H_g), 5.30 (s, 1H, H_l), 2.62 (dd, $^3J_{HH}$ = 8.6 Hz, $^3J_{HH}$ = 7.0 Hz, 4H, H_n), 1.80 (s, 6H, H_m), 1.61-1.56 (m, 4H, H_o), 1.38-1.32 (m, 4H, H_p), 0.92 (t, $^3J_{HH}$ = 7.4 Hz, 6H, H_q) ppm.[2] **MS-MALDI**: m/z 1112.3 $[M]^+$. **Elem. Anal.** for $C_{67}H_{55}IrN_2O_2\cdot½CH_2Cl_2$: C, 70.20; H, 4.89; N, 2.43 %. **Found**: C,70.02; H, 4.77; N, 2.40 %.

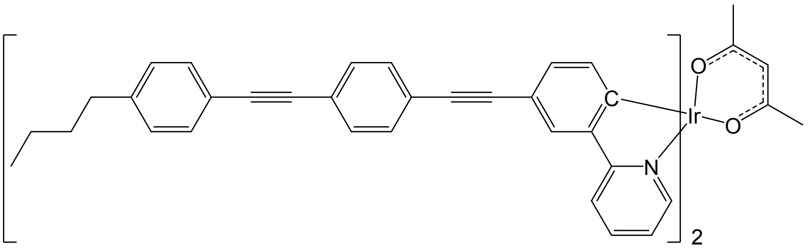

Ir(L^{13})$_2$(acac) (**16**). A dark red powder. **Yield**: 194 mg (84%). **^{1}H NMR** (599.42 MHz; $CDCl_3$, 25 ^{0}C): δ_H 8.65 (d, $^4J_{HH}$ = 1.7 Hz, 2H, H_a), 7.90-7.86 (m, 4H, H_b+H_c), 7.59 (dd, $^3J_{HH}$ = 7.8 Hz, $^4J_{HH}$ = 1.3 Hz, 2H, H_d), 7.54-7.51 (m, 8H, H_i+H_h), 7.44 (d, $^3J_{HH}$ = 8.0 Hz, 4H, H_j), 7.19 (d, $^3J_{HH}$ = 8.0 Hz, 4H, H_k), 6.88 (td, $^3J_{HH}$ = 7.4 Hz, $^4J_{HH}$ = 1.3 Hz, 2H, H_e), 6.73 (td, $^3J_{HH}$ = 7.4 Hz, $^4J_{HH}$ = 1.3 Hz, 2H, H_f), 6.30 (dd, $^3J_{HH}$ = 7.7 Hz, $^4J_{HH}$ = 1.2 Hz, 2H, H_g), 5.36 (s, 1H, H_l), 2.63 (t, $^3J_{HH}$ = 7.3 Hz, 4H, H_n), 1.87 (s, 6H, H_m), 1.62-1.57 (m, 4H, H_o), 1.39-1.32 (m, 4H, H_p), 0.93 (t, J = 7.4 Hz, 6H, H_q) ppm. **^{13}C{^{1}H} NMR** (150.72 MHz; $CDCl_3$, 25 ^{0}C): δ_C

[2] Solubility too low for a suitable ^{13}C NMR spectrum.

185.5, 168.1, 150.9, 148.6, 144.8, 144.5, 140.0, 133.6, 132.0, 131.9, 131.8, 129.7, 129.0, 124.9, 124.5, 122.3, 121.5, 120.3, 118.5, 118.3, 101.1, 93.3, 92.3, 88.5, 87.3, 35.9, 33.8, 28.8, 22.7, 14.0 ppm. **MS-MALDI**: m/z 1112.2 $[M]^+$. **Elem. Anal.** for $C_{67}H_{55}IrN_2O_2 \cdot 1\frac{1}{4}CH_2Cl_2$: C, 67.27; H, 4.76; N, 2.30 %. **Found**: C, 67.71; H, 4.76; N, 1.91 %.

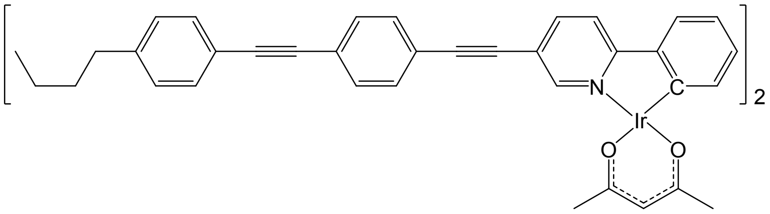

Ir(L^14)_2(acac) (**18**). A bright yellow-green powder. **Yield**: 119 mg (53%). **^{1}H NMR** (599.42 MHz; $CDCl_3$, 25 ^{0}C): δ_H 8.45 (dd, $^3J_{HH}$ = 5.7 Hz, $^4J_{HH}$ = 1.4 Hz, 2H, H_a), 7.80 (d, $^3J_{HH}$ = 8.2 Hz, 2H, H_d), 7.65 (td, $^3J_{HH}$ = 7.9 Hz, $^4J_{HH}$ = 1.6 Hz, 2H, H_c), 7.55 (d, $^3J_{HH}$ = 8.8 Hz, 2H, H_e), 7.49 (pseudo singlet, 8H, H_l+H_m), 7.45 (d, $^3J_{HH}$ = 7.8 Hz, 4H, H_n), 7.17 (d, $^3J_{HH}$ = 7.9 Hz, 4H, H_o), 7.05 (ddd, $^3J_{HH}$ = 7.2 Hz, $^3J_{HH}$ = 5.6 Hz, $^4J_{HH}$ = 1.3 Hz, 2H, H_b), 6.50 (dd, $^3J_{HH}$ = 7.8 Hz, $^4J_{HH}$ = 1.5 Hz, 2H, H_f), 6.01 (d, $^4J_{HH}$ = 1.5 Hz, 2H, H_g), 5.28 (s, 1H, H_t), 2.63 (t, $^3J_{HH}$ = 7.8 Hz, 4H, H_p), 2.46 (s, 6H, H_i or H_h), 2.40 (s, 6H, H_i or H_h), 2.01 (s, 6H, H_j or H_k), 1.83 (s, 6H, H_u), 1.61 (h, $^3J_{HH}$ = 7.2 Hz, 4H, H_q), 1.41 (s, 6H, H_j or H_k), 1.36 (h, $^3J_{HH}$ = 7.2 Hz, 4H, H_r), 0.94 (t, $^3J_{HH}$ = 7.4 Hz, 6H, H_s) ppm. **^{13}C{^{1}H} NMR** (150.72 MHz; $CDCl_3$, 25 ^{0}C): δ_C 184.5, 168.3, 148.2, 146.9, 143.5, 143.0, 142.1, 137.5, 136.9, 135.5, 133.8, 132.4, 131.7, 131.5, 131.4, 131.1, 128.4, 128.8, 123.4, 122.8, 121.5, 121.4, 120.1, 118.2, 91.2, 90.7, 88.6, 35.6, 33.3, 28.7, 22.3, 18.5, 18.2, 17.2, 13.9 ppm, solubility too low to observe all alkyne signals. **MS-MALDI**: m/z 1376.4 $[M]^+$. **Elem. Anal.** for $C_{87}H_{79}IrN_2O_2 \cdot CH_2Cl_2$: C, 72.31; H, 5.59; N, 1.92 %. **Found**: C, 72.40; H, 5.84; N, 2.06 %.

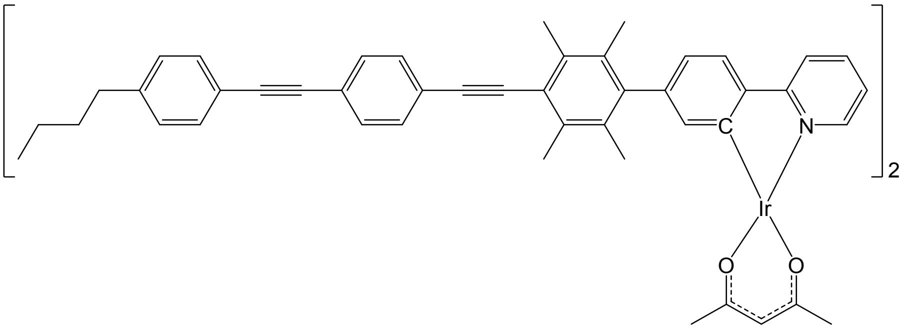

Ir(L^{15})$_2$(acac) (**19**). A bright yellow-green powder. **Yield**: 85 mg (38%). **^{1}H NMR** (599.42 MHz; $CDCl_3$, 25 ^{0}C): δ_H 8.28 (d, $^3J_{HH}$ = 1.9 Hz, 2H, H_a), 7.96 (d, $^3J_{HH}$ = 8.1 Hz, 2H, H_b), 7.65 (dd, $^3J_{HH}$ = 7.8 Hz, $^4J_{HH}$ = 1.3 Hz, 2H, H_d), 7.58-7.53 (m, 10H, H_c+H_l+H_m), 7.46 (d, $^3J_{HH}$ = 8.0 Hz, 4H, H_n), 7.21 (d, $^3J_{HH}$ = 8.0 Hz, 4H, H_o), 6.90 (td, $^3J_{HH}$ = 7.4 Hz, $^4J_{HH}$ = 1.4 Hz, 2H, H_e), 6.76 (td, $^3J_{HH}$ = 7.4 Hz, $^4J_{HH}$ = 1.4 Hz, 2H, H_f), 6.46 (dd, $^3J_{HH}$ = 7.8 Hz, $^4J_{HH}$ = 1.3 Hz, 2H, H_g), 5.23 (s, 1H, H_t), 2.64 (t, $^3J_{HH}$ = 7.8 Hz, 4H, H_p), 2.56 (s, 12H, H_i+H_h), 2.14 (s, 6H, H_j or H_k), 1.99 (s, 6H, H_j or H_k), 1.67 (s, 6H, H_u), 1.63-1.60 (m, 4H, H_q), 1.40-1.34 (m, 4H, H_r), 0.94 (t, $^3J_{HH}$ = 7.3 Hz, 6H, H_s) ppm. **^{13}C{^{1}H} NMR** (150.72 MHz; $CDCl_3$, 25 ^{0}C): δ_C 184.7, 166.4, 1485, 147.4, 145.1, 143.9, 138.3, 137.4, 136.2, 135.9, 133.3, 132.4, 131.4, 131.2, 128.5, 123.7, 123.4, 123.0, 120.8, 120.0, 118.1, 100.1, 96.9, 91.3, 90.3, 88.3, 35.5, 33.3, 28.0, 22.2, 18.3, 18.0, 17.4, 13.6 ppm. **MS-MALDI**: m/z 1376.5 $[M]^+$. **Elem. Anal.** for $C_{87}H_{80}IrN_2O_2\cdot$¾$CH_2Cl_2$: C, 73.11; H, 5.70; N, 1.94 %. **Found**: C, 7328; H, 5.83; N, 1.74 %.

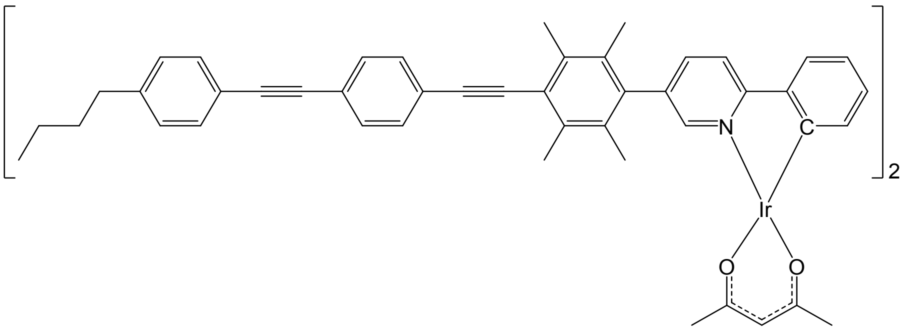

Ir(L^{16})(ppy)(acac) (**21**)

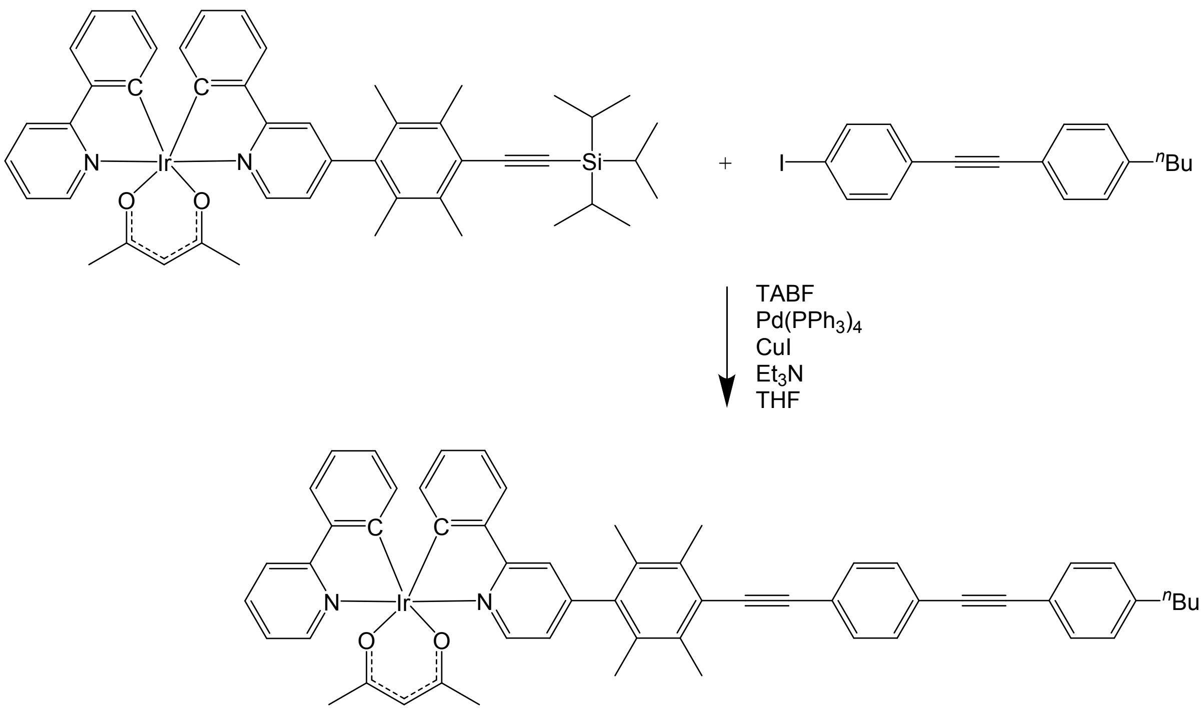

THF (50 mL) and Et_3N (5 mL) were added to the complex (**13**) (1eq), 1-butyl-4-((4-iodophenyl)ethynyl)benzene (1.5 eq), $Pd(PPh_3)_4$ (10 mol%) and CuI (10 mol%). The solution was degassed by three freeze-pump-thaw cycles, before tetrabutylammonium fluoride (1.0 M in THF, 1.5 eq) was added. The solution was stirred at room temperature for 12 hours before the solvent was removed. The residue was purified by silica chromatography eluted by DCM. A bright yellow-green powder. **Yield**: 104 mg (48%). **^{1}H NMR** (599.42 MHz; $CDCl_3$, 25 ^{0}C): δ_H 8.57-8.55 (m, 2H, H_a+$H_{a'}$), 7.85 (d, $^3J_{HH}$ = 8.1 Hz, 1H, $H_{d'}$), 7.73 (t, $^3J_{HH}$ = 7.9 Hz, 1H, $H_{c'}$), 7.62 (s, 1H, H_c), 7.56-7.52 (m, 5H, H_h+H_i+($H_{e'}$ or H_d)), 7.49 (d, $^3J_{HH}$ = 7.8 Hz, 1H, $H_{e'}$ or H_d), 7.46 (d, $^3J_{HH}$ = 7.6 Hz, 2H, H_j), 7.18-7.14 (m, 3H, $H_{b'}$+H_k), 6.94 (d, $^3J_{HH}$ = 6.0 Hz, 1H, H_b), 6.82 (t, $^3J_{HH}$ = 7.5 Hz, 1H, H_e or $H_{f'}$), 6.78 (t, $^3J_{HH}$ = 7.5 Hz, 1H, H_e or $H_{f'}$), 6.73 (t, $^3J_{HH}$ = 7.4 Hz, 1H, H_f or $H_{g'}$), 6.69 (t, $^3J_{HH}$ = 7.5 Hz, 1H, H_f or $H_{g'}$), 6.34 (d, $^3J_{HH}$ = 7.7 Hz, 1H, H_g or $H_{h'}$), 6.31 (d, $^3J_{HH}$ = 7.8 Hz, 1H, H_g or $H_{h'}$), 5.28 (s, 1H, H_l), 2.63 (t, $^3J_{HH}$ = 7.8 Hz, 2H, H_n), 2.58 (s, 3H, H_s or $H_{s'}$), 2.57 (s, 3H, H_s or $H_{s'}$), 2.11 (s, 3H, H_r or $H_{r'}$), 2.06 (s, 3H, H_r or $H_{r'}$), 1.84 (s, 3H, H_m or $H_{m'}$), 1.79 (s, 3H, H_m or $H_{m'}$), 1.63-1.59 (m, 2H, H_o), 1.38-1.34 (m, 2H, H_p), 0.94 (t, $^3J_{HH}$ = 7.3 Hz, 3H, H_q) ppm. **^{13}C{^{1}H} NMR** (150.72 MHz; $CDCl_3$, 25 ^{0}C): δ_C 184.6, 168.6, 151.6, 148.2, 148.0, 147.7, 147.5, 144.8, 144.6, 143.6, 139.3, 136.7, 136.4, 133.0, 132.8, 131.5, 131.2, 129.1, 128.5, 123.9, 123.8, 123.4, 123.2, 122.5, 121.4, 120.7, 120.1, 119.2, 118.3, 100.4, 97.3, 91.4, 90.0, 88.5, 35.6, 33.3, 28.8, 28.7, 22.3, 18.5, 18.0, 17.9, 13.9 ppm. **MS-MALDI**: m/z 988.2 $[M]^+$. **Elem. Anal.** for $C_{57}H_{51}IrN_2O_2\cdot½CH_2Cl_2$: C, 67.00; H, 5.08; N, 2.72 %. **Found**: C, 66.51; H, 4.99; N, 3.08 %.

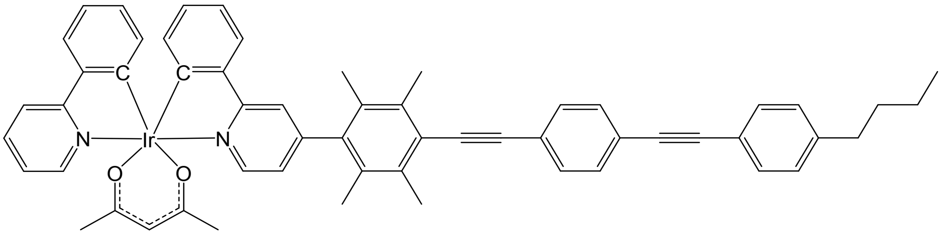

C
C
N
Ir
N
O
O

S2. NMR spectra of reported compounds

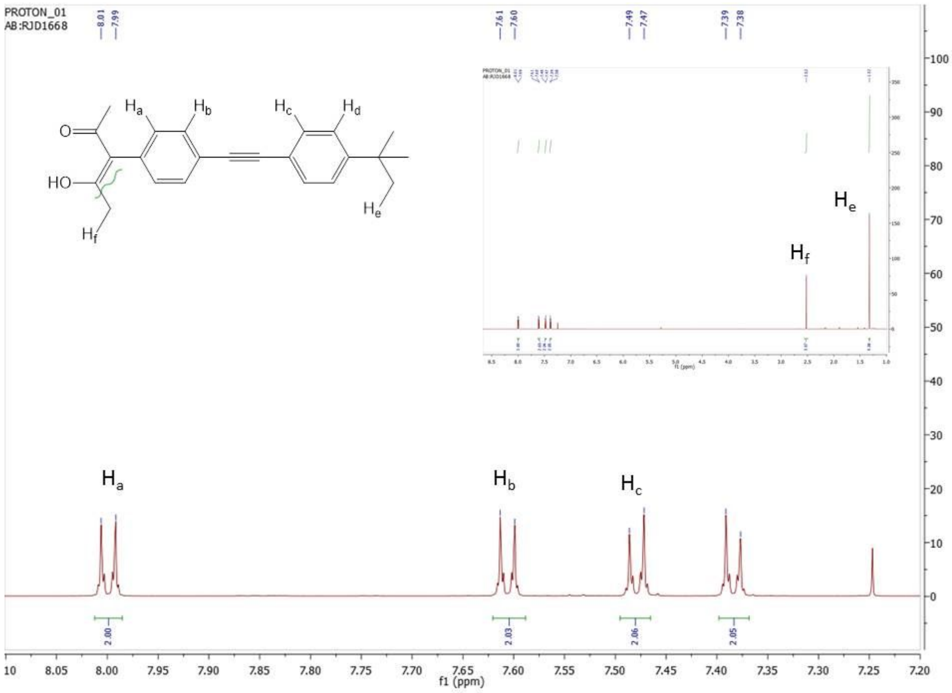

Figure S1. 600 MHz ^{1}H NMR spectrum of $\mathbf{L^2}$H, recorded in $CDCl_3$ at 25 ^{0}C.

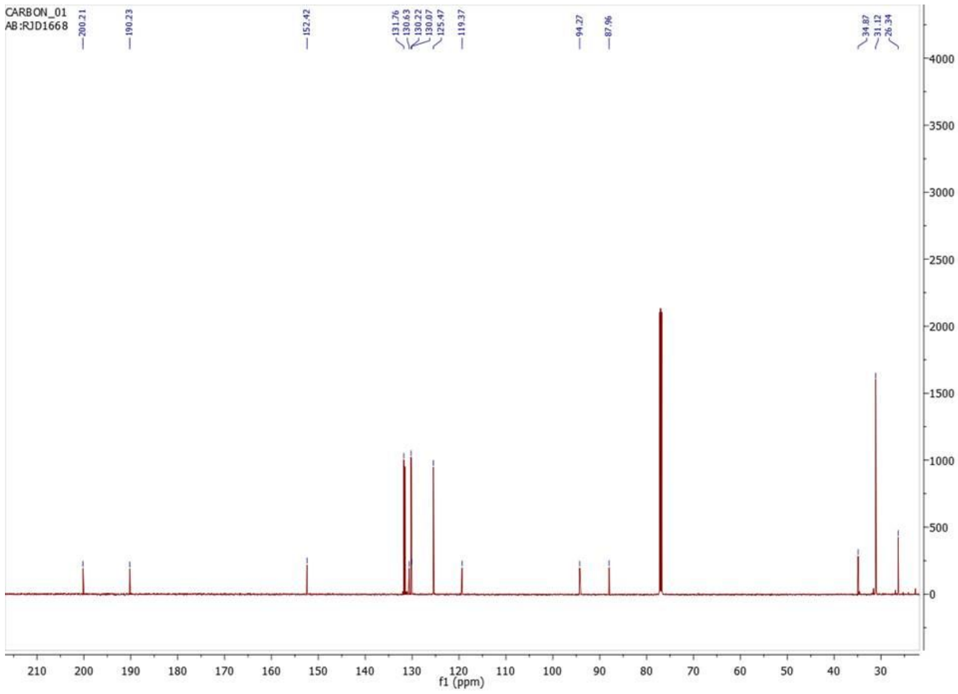

Figure S2. 151 MHz $^{13}C\{^{1}H\}$ NMR spectrum of $\mathbf{L^2}$H, recorded in $CDCl_3$ at 25 ^{0}C.

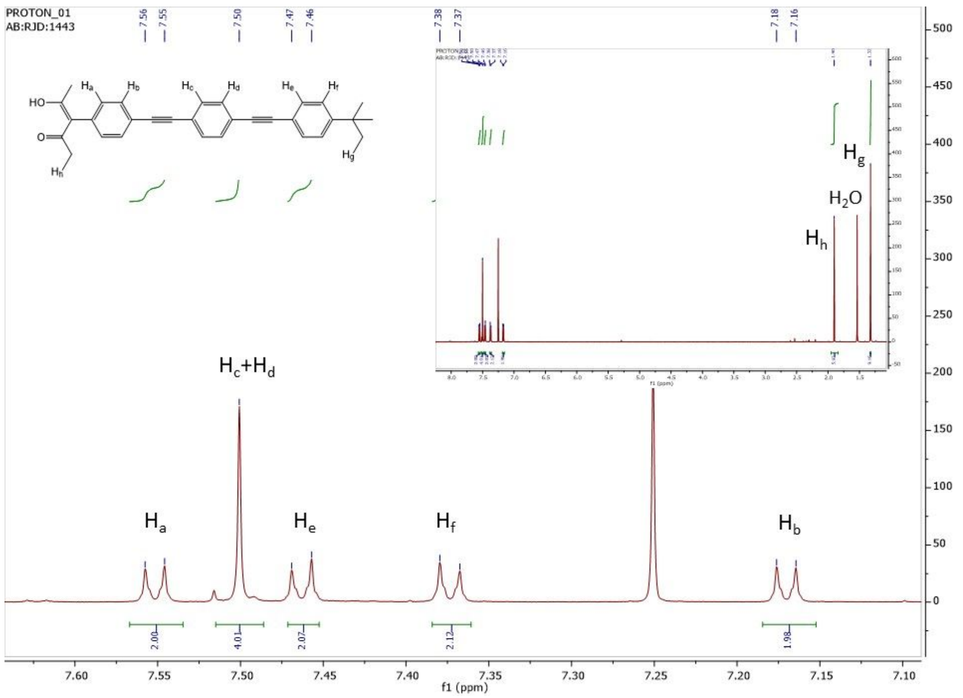

Figure S3. 600 MHz ^{1}H NMR spectrum of **L^3**H, recorded in $CDCl_3$ at 25 ^{0}C.

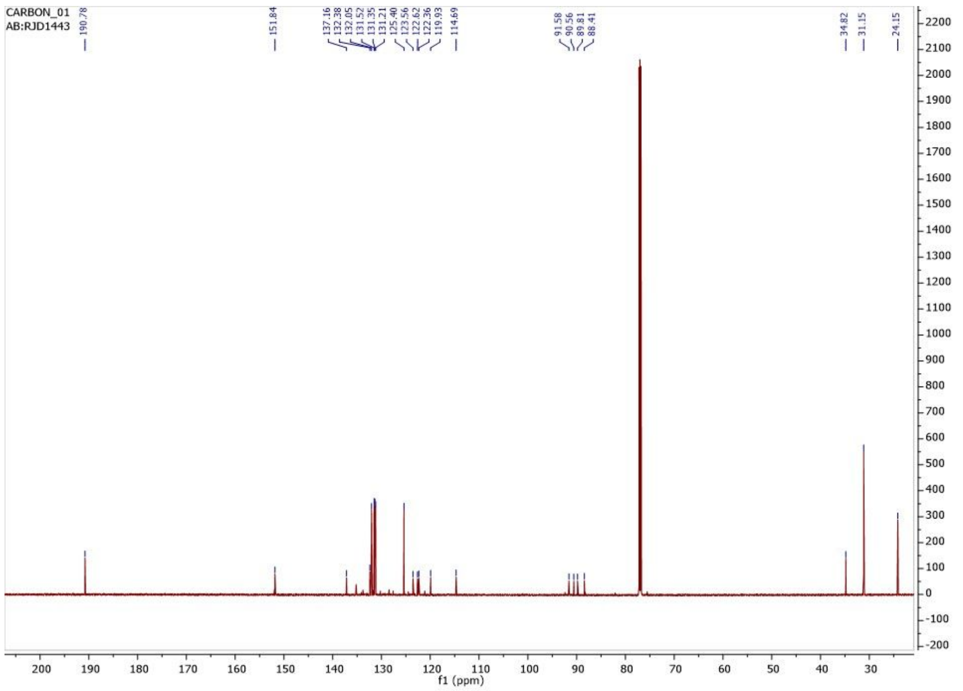

Figure S4. 151 MHz $^{13}C\{^1H\}$ NMR spectrum of **L^3**H, recorded in $CDCl_3$ at 25 ^{0}C.

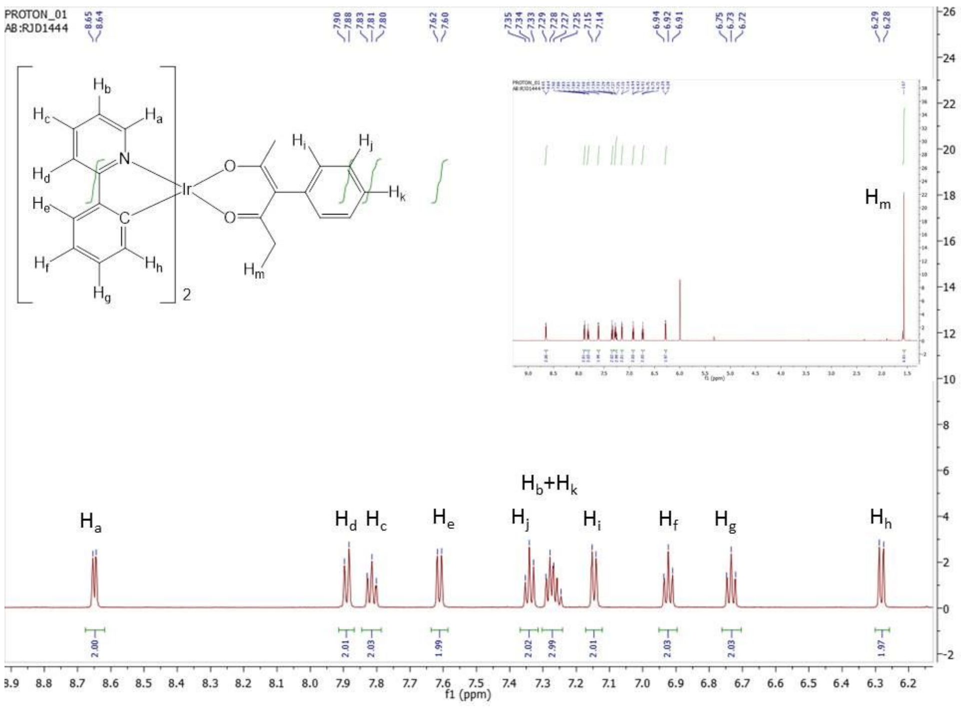

Figure S5. 600 MHz ^{1}H NMR spectrum of **1**, recorded in TCE-d_2 at 25 ^{0}C.

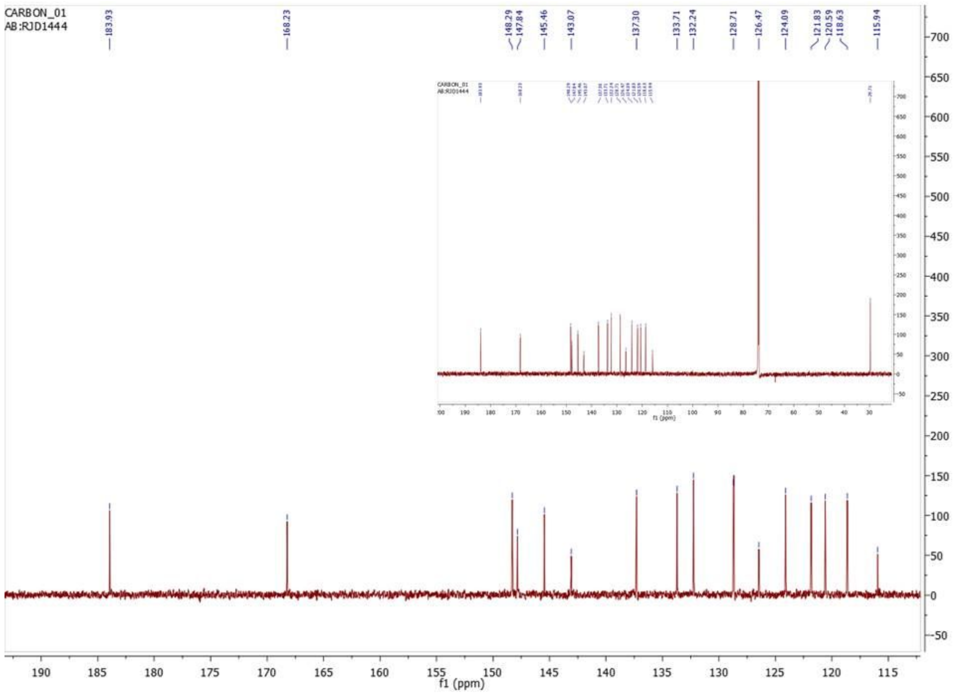

Figure S6. 151 MHz $^{13}C\{^{1}H\}$ NMR spectrum of **1**, recorded in TCE-d_2 at 25 ^{0}C.

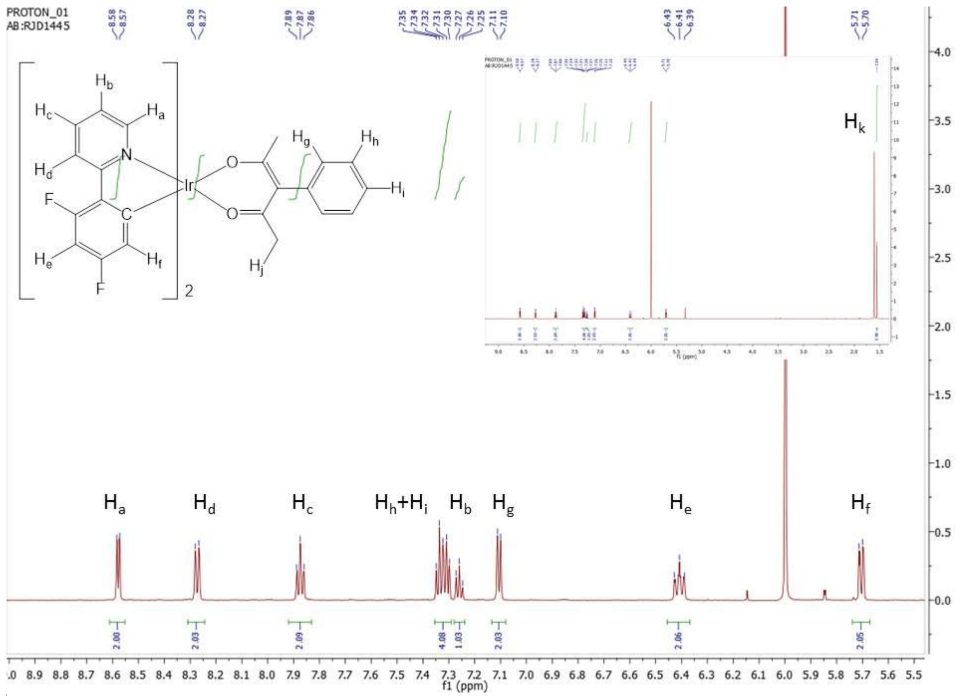

Figure S7. 151 MHz ^{1}H NMR spectrum of **2**, recorded in TCE-d_2 at 25 ^{0}C.

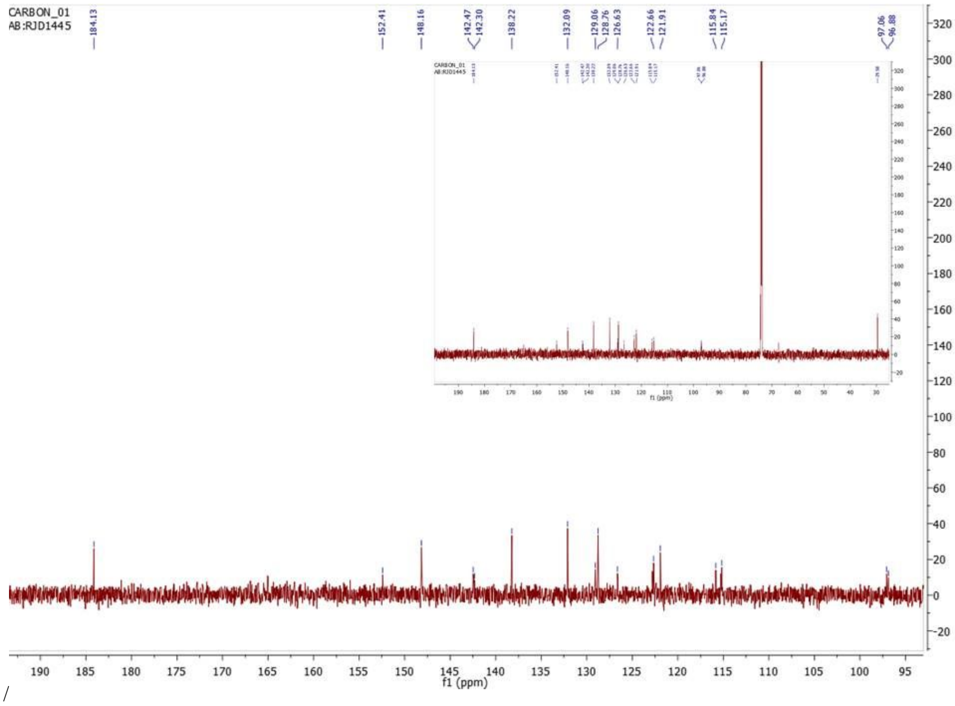

Figure S8. 151 MHz $^{13}C\{^{1}H\}$ NMR spectrum of **2**, recorded in TCE-d_2 at 25 ^{0}C.

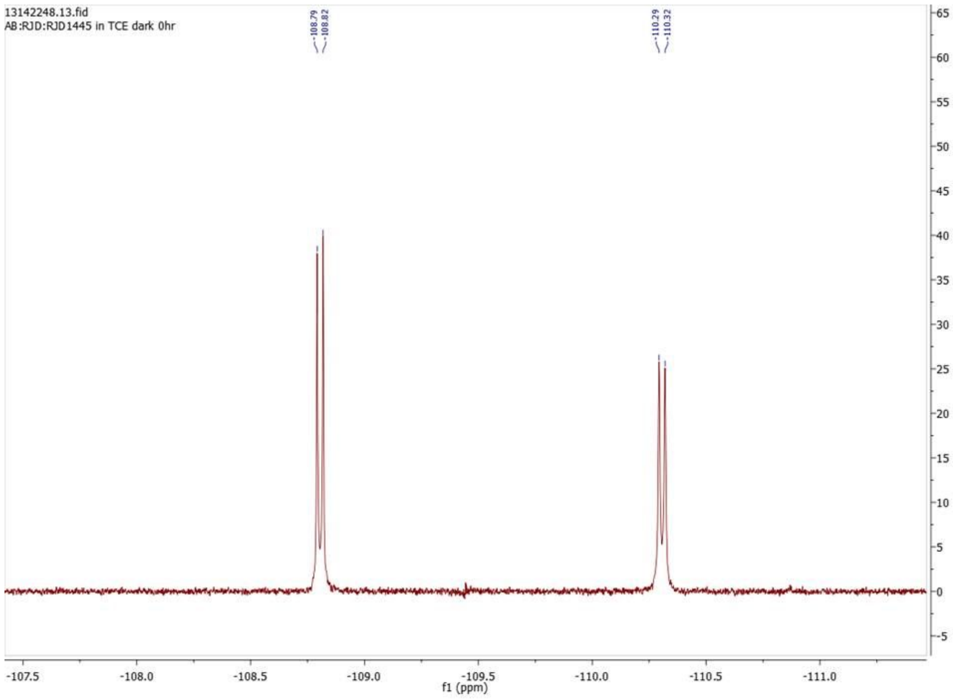

Figure S9. 376 MHz ^{19}F NMR spectrum of **2**, recorded in TCE-d_2 at 25 ^0C.

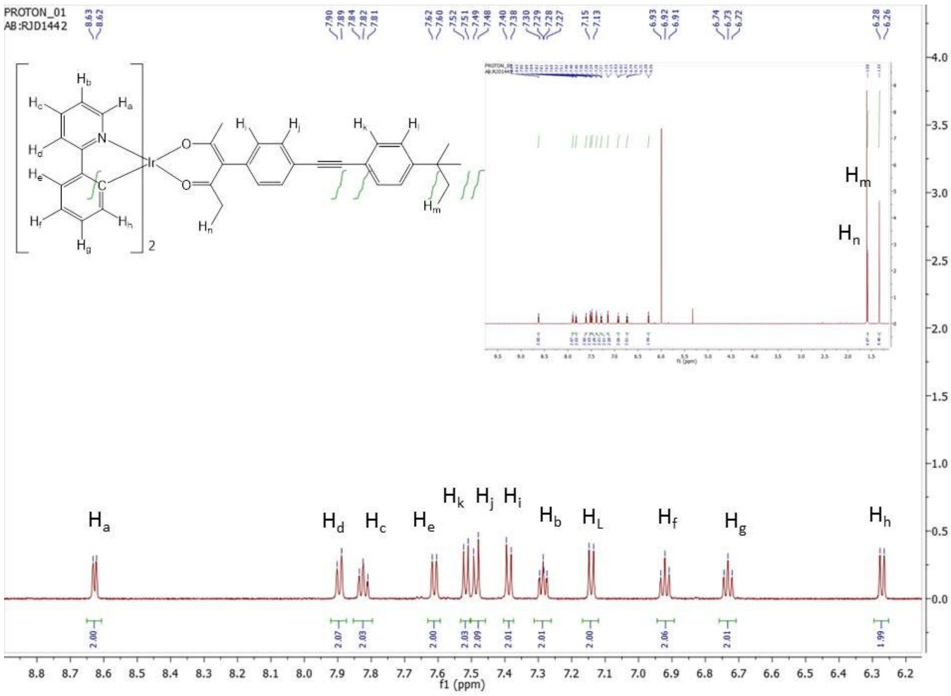

Figure S10. 376 MHz ^1H NMR spectrum of **3**, recorded in TCE-d_2 at 25 ^0C.

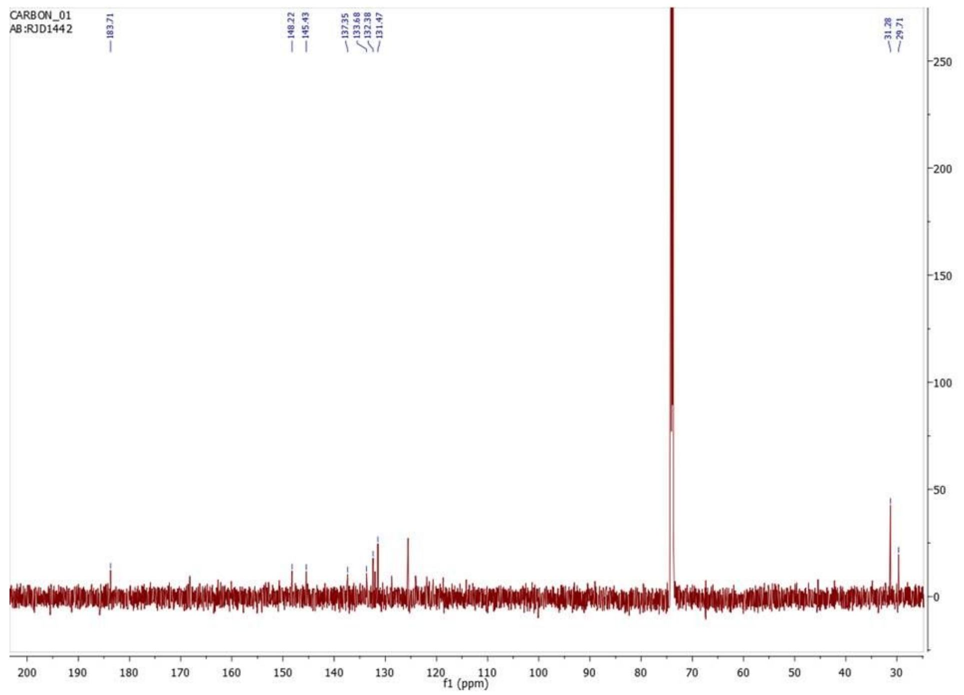

Figure S11. 151 MHz $^{13}C\{^{1}H\}$ NMR spectrum of **3**, recorded in TCE-d_2 at 25 ^{0}C.

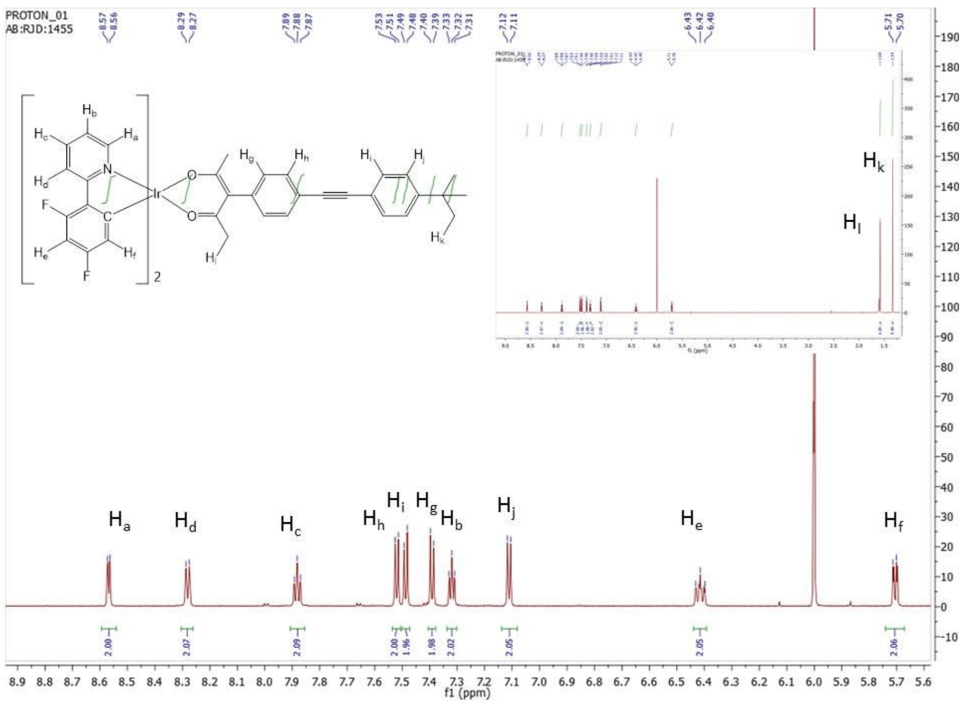

Figure S12. 600 MHz ^{1}H NMR spectrum of **4**, recorded in TCE-d_2 at 25 ^{0}C.

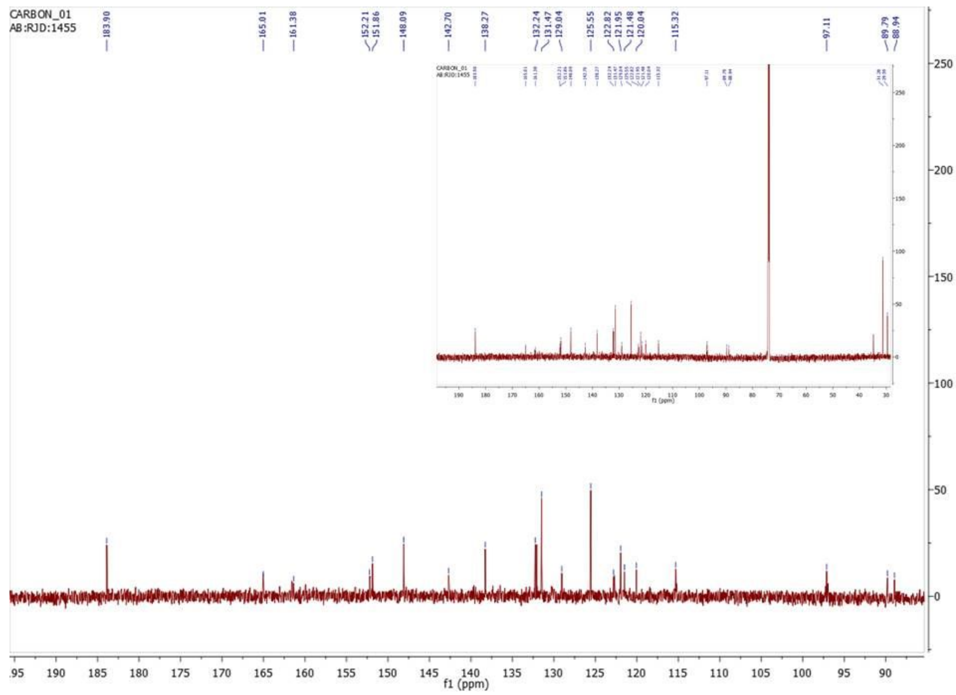

Figure S13. 600 MHz $^{13}C\{^{1}H\}$ NMR spectrum of **4**, recorded in TCE-d_2 at 25 ^{0}C.

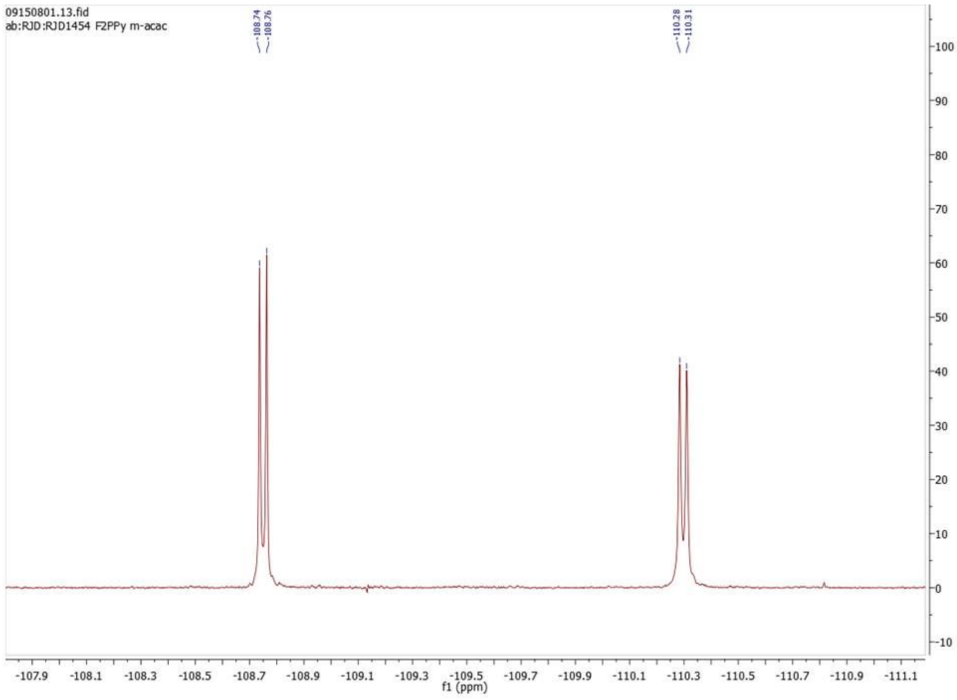

Figure S14. 376 MHz ^{19}F NMR spectrum of **4**, recorded in TCE-d_2 at 25 ^{0}C.

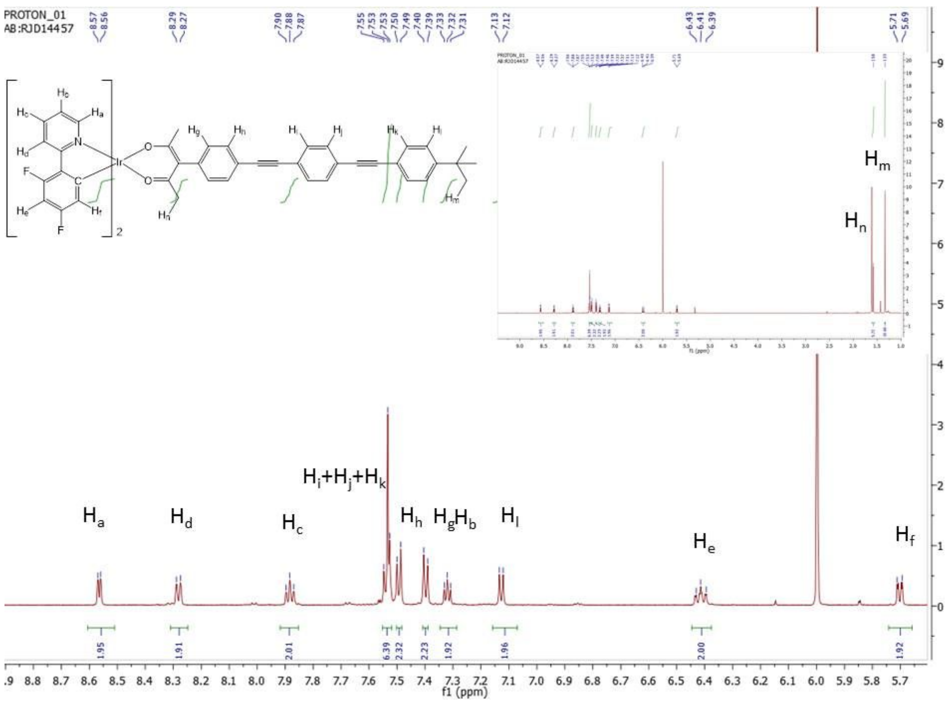

Figure S15. 600 MHz ^{1}H NMR spectrum of **5**, recorded in TCE-d_2 at 25 ^{0}C.

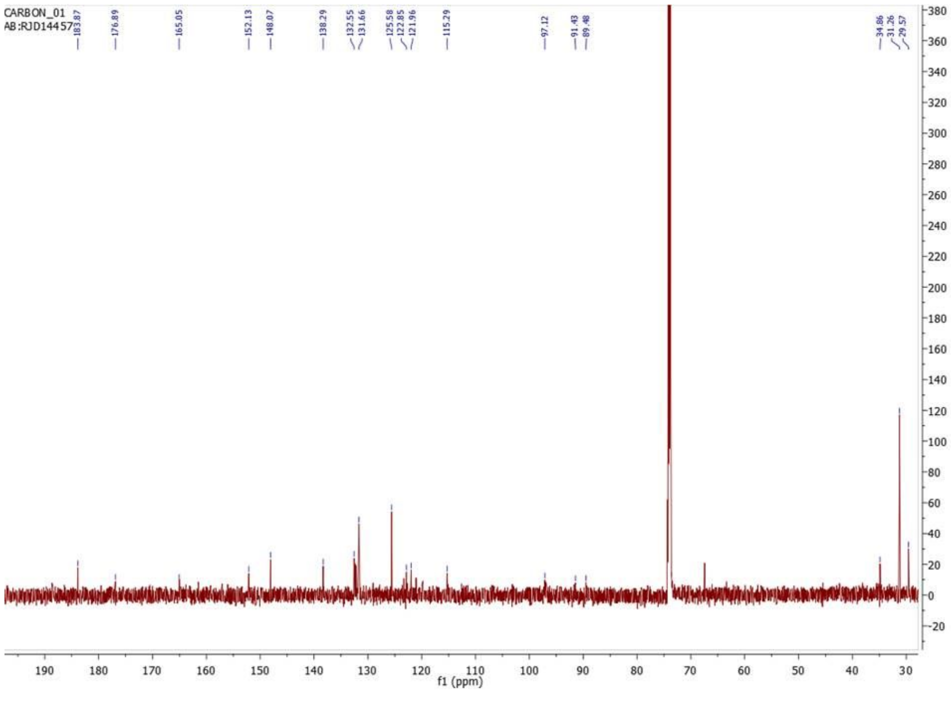

Figure S16. 151 MHz $^{13}C\{^{1}H\}$ NMR spectrum of **5**, recorded in TCE-d_2 at 25 ^{0}C.

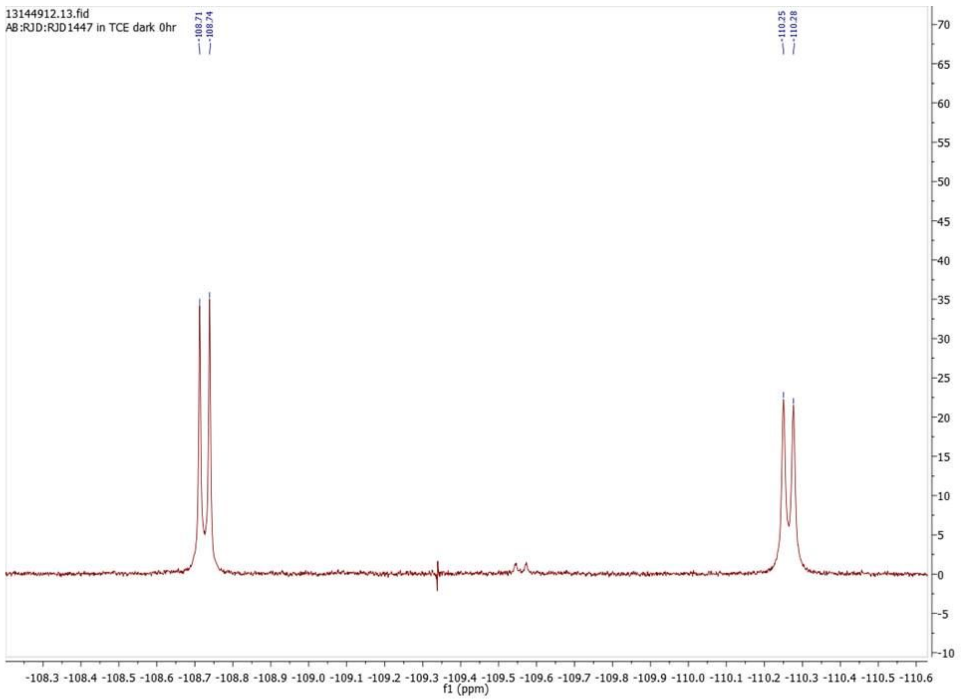

Figure S17. 376 MHz ^{19}F NMR spectrum of **5**, recorded in TCE-d_2 at 25 ^{0}C.

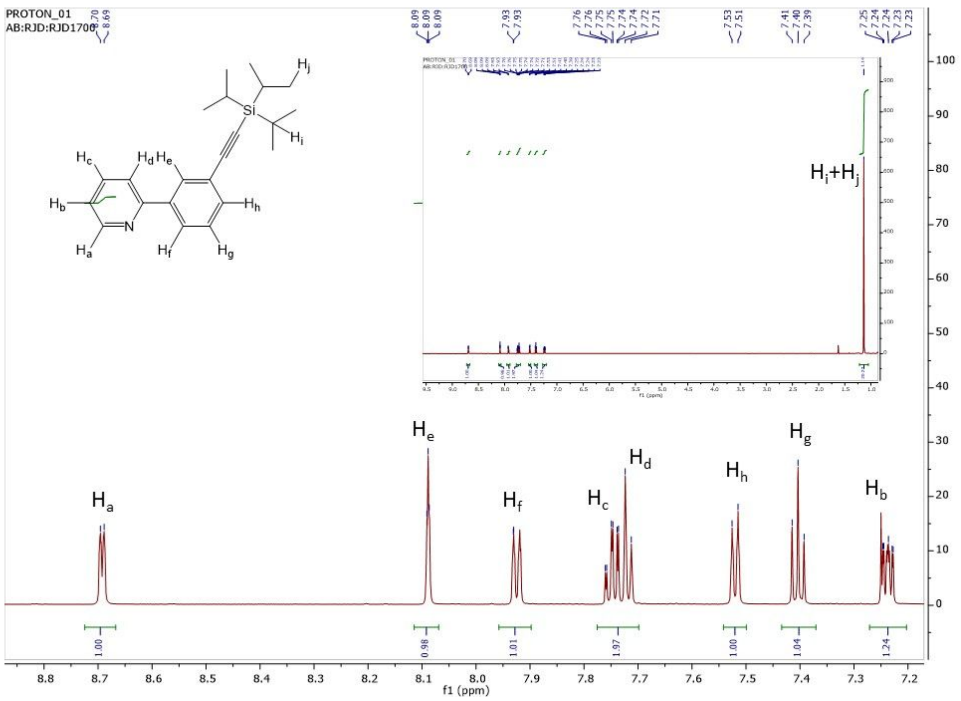

Figure 18. 600 MHz ^1H NMR spectrum of **L^5**H, recorded in $CDCl_3$ at 25 ^{0}C.

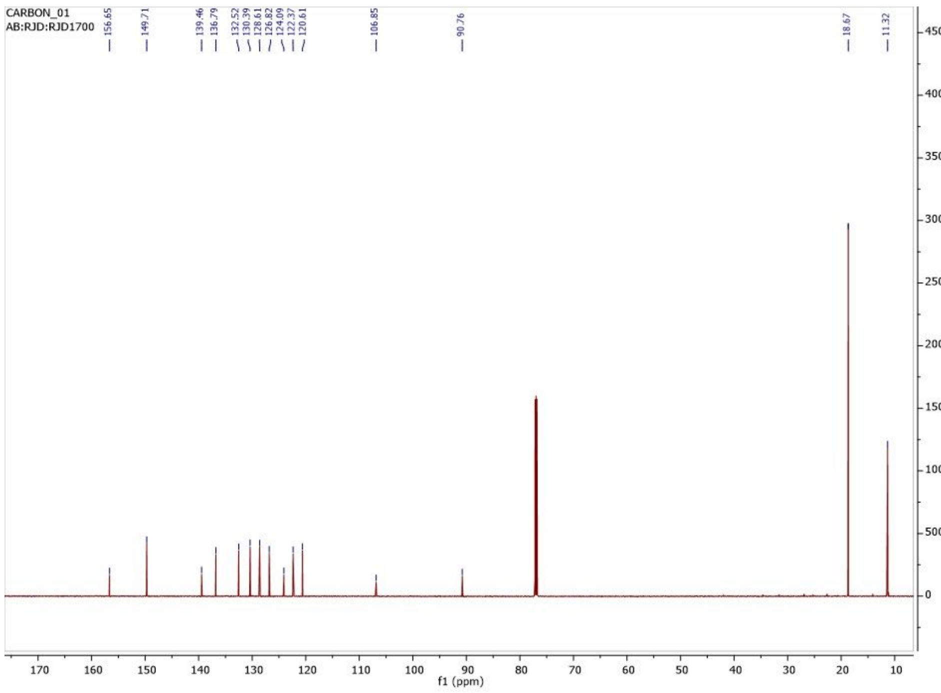

Figure S19. 151 MHz $^{13}C\{^{1}H\}$ NMR spectrum of **L**^{5}H, recorded in $CDCl_3$ at 25 ^{0}C.

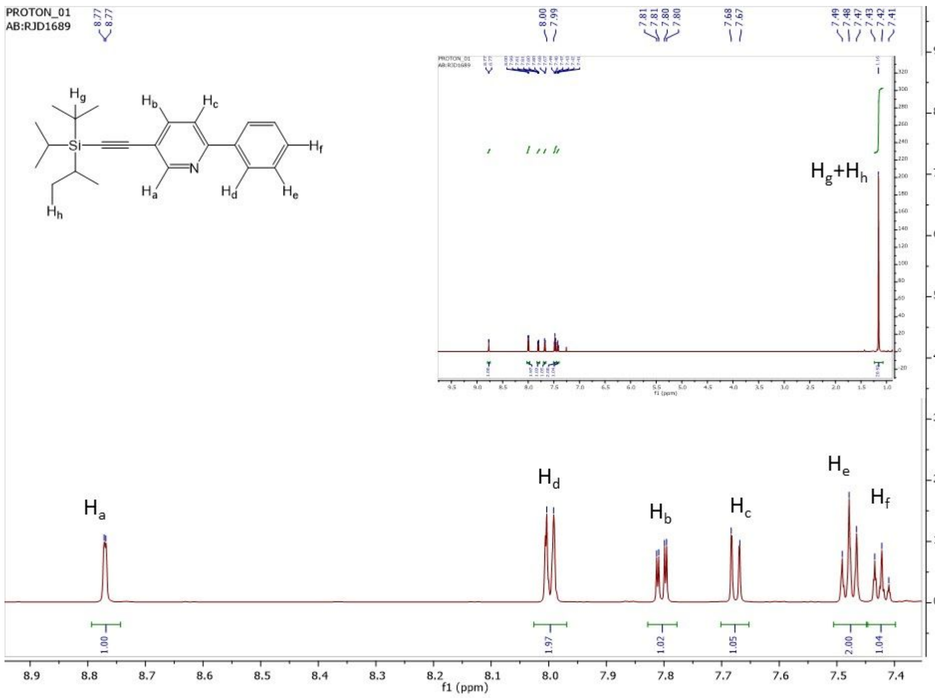

Figure S20. 600 MHz ^{1}H NMR spectrum of **L**^{6}H, recorded in $CDCl_3$ at 25 ^{0}C.

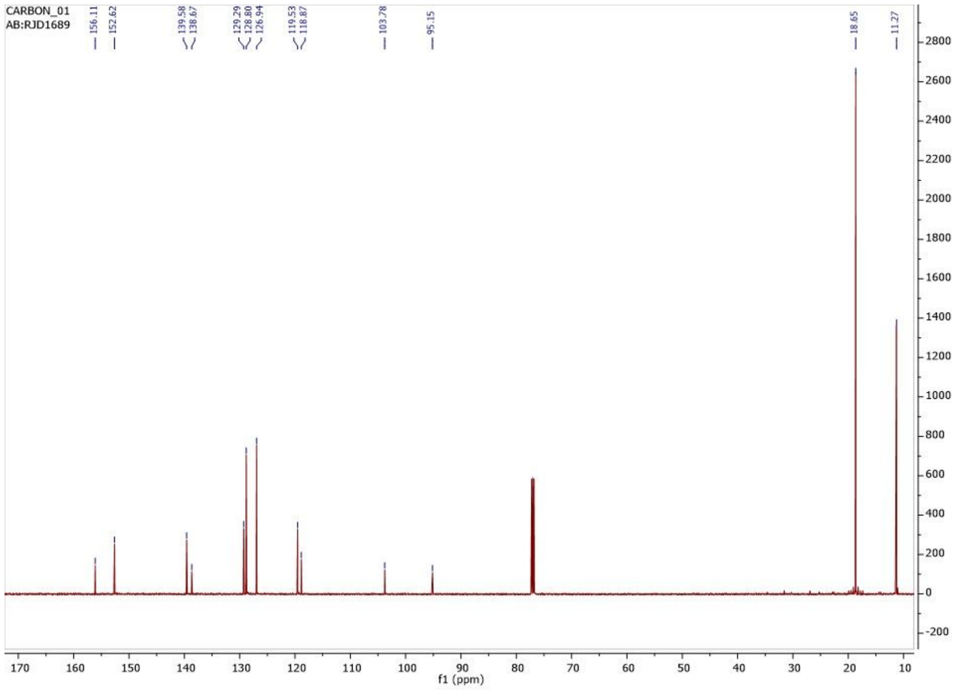

Figure S21. 151 MHz $^{13}C\{^{1}H\}$ NMR spectrum of $\mathbf{L^6}H$, recorded in $CDCl_3$ at 25 ^{0}C.

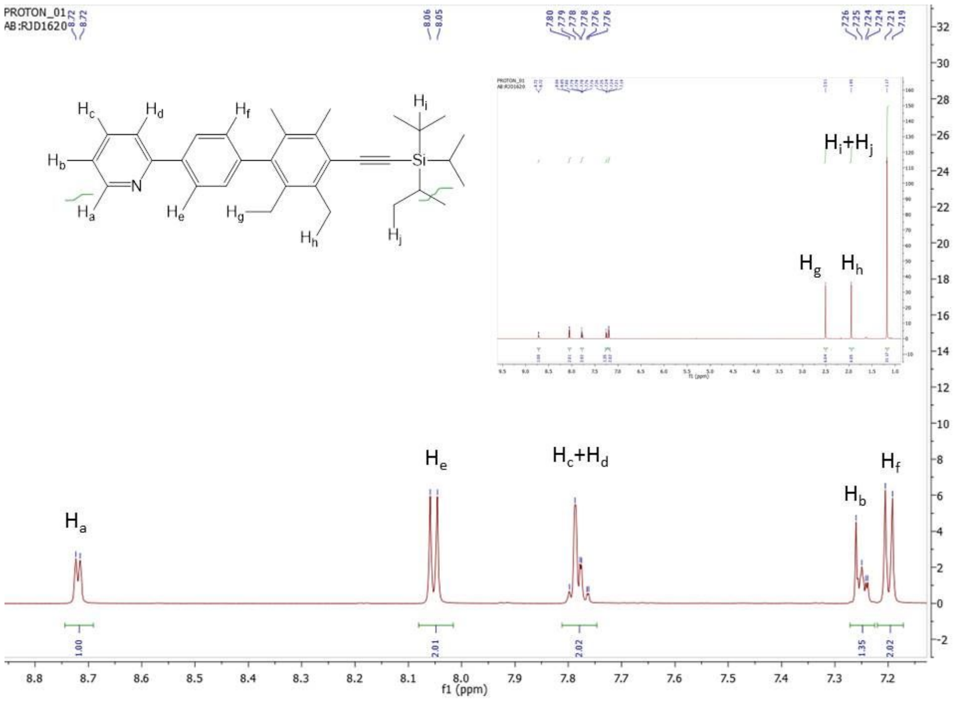

Figure S22. 600 MHz ^{1}H NMR spectrum of $\mathbf{L^8}H$ recorded in $CDCl_3$ at 25 ^{0}C.

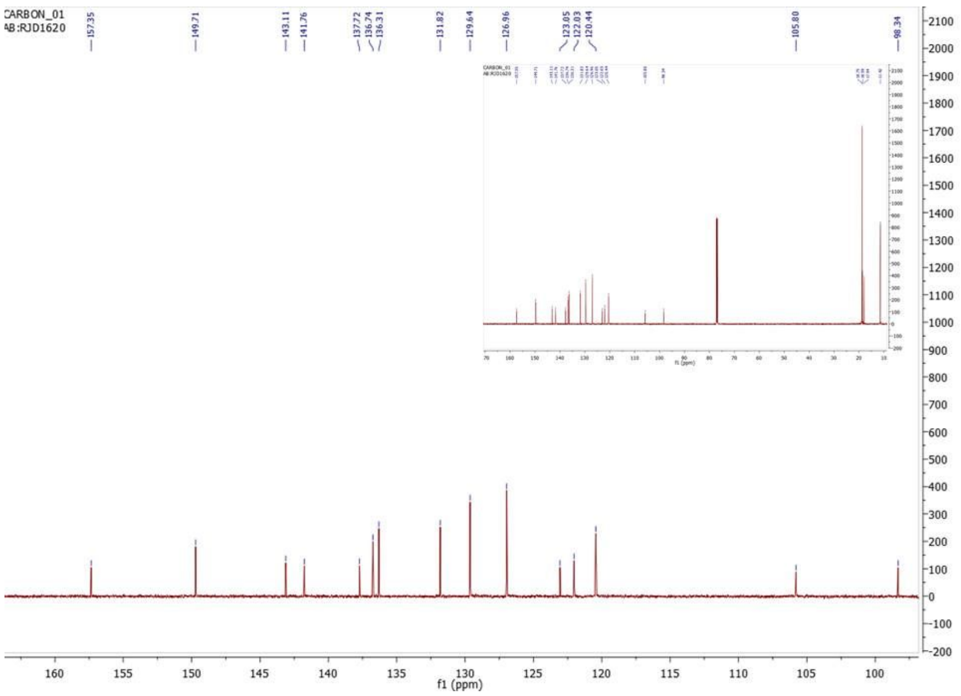

Figure S23. 151 MHz $^{13}C\{^{1}H\}$ NMR spectrum of **L⁸**H recorded in $CDCl_3$ at 25 ^{0}C.

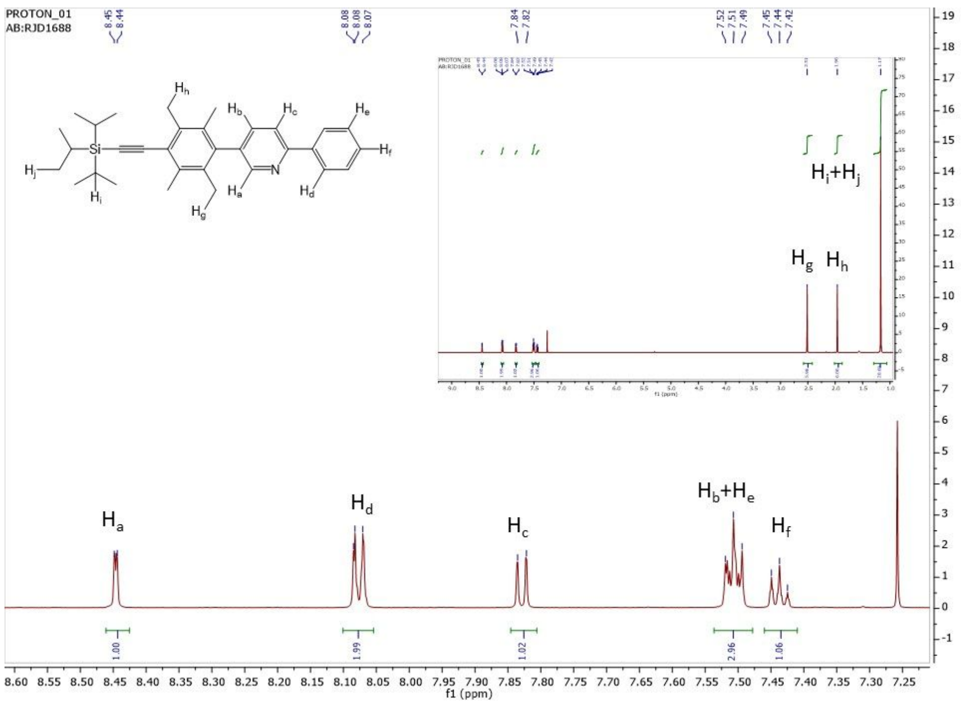

Figure S24. 600 MHz ^{1}H NMR spectrum of **L⁹**H recorded in $CDCl_3$ at 25 ^{0}C.

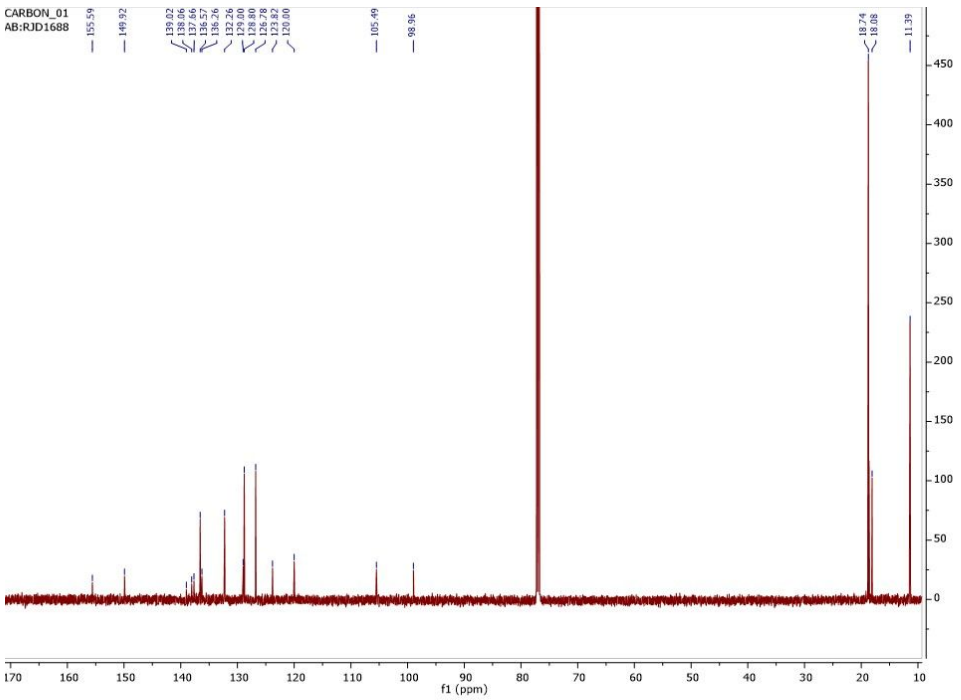

Figure S25. 151 MHz $^{13}C\{^{1}H\}$ NMR spectrum of $\mathbf{L^9}$H recorded in $CDCl_3$ at 25 ^{0}C.

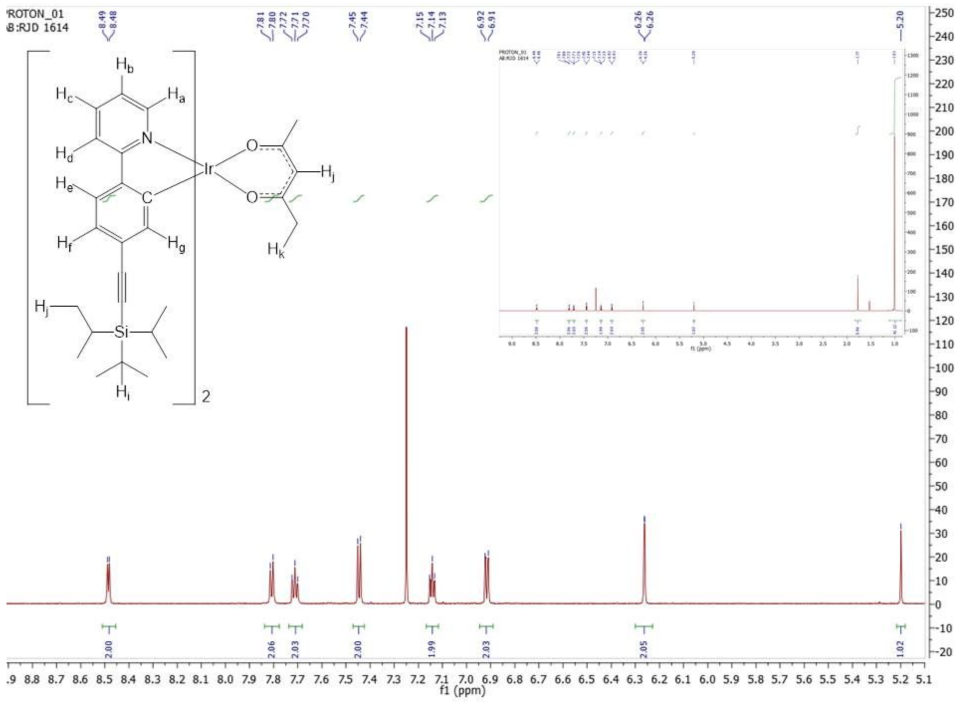

Figure S26. 600 MHz ^{1}H NMR spectrum of **6**, recorded in $CDCl_3$ at 25 ^{0}C.

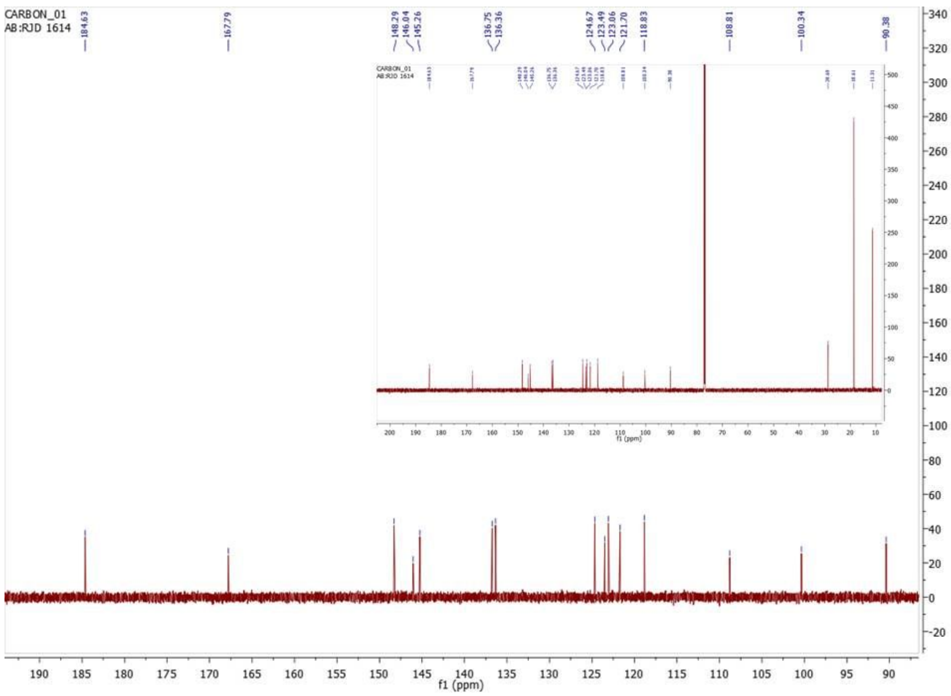

Figure S27. 151 MHz $^{13}C\{^{1}H\}$ NMR spectrum of **6**, recorded in $CDCl_3$ at 25 ^{0}C.

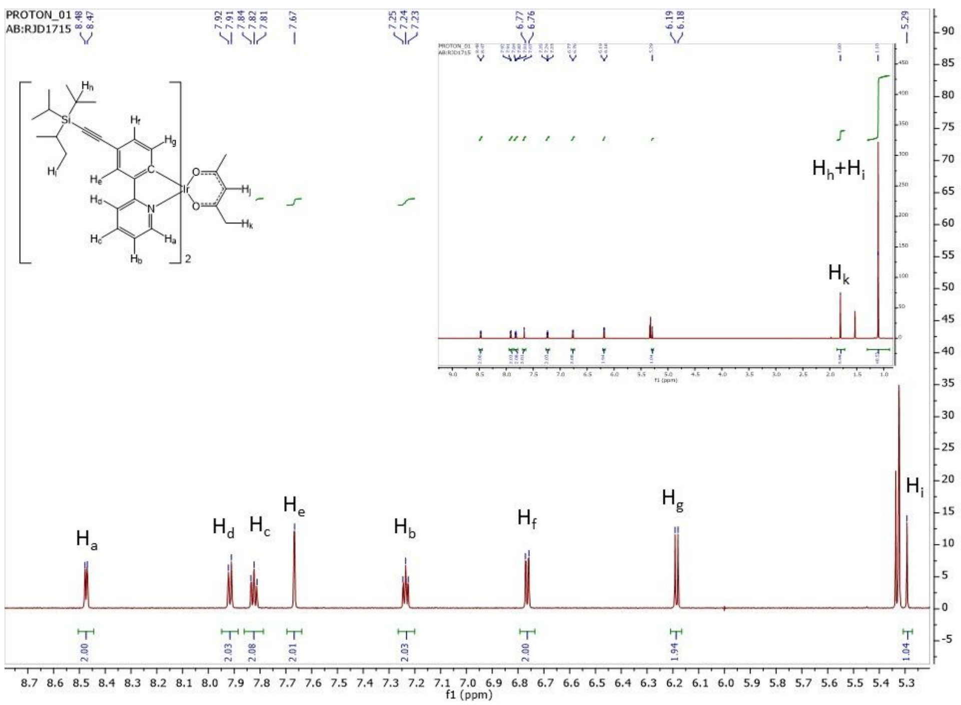

Figure S28. 600 MHz ^{1}H NMR spectrum of **7**, recorded in CD_2Cl_2 at 25 ^{0}C.

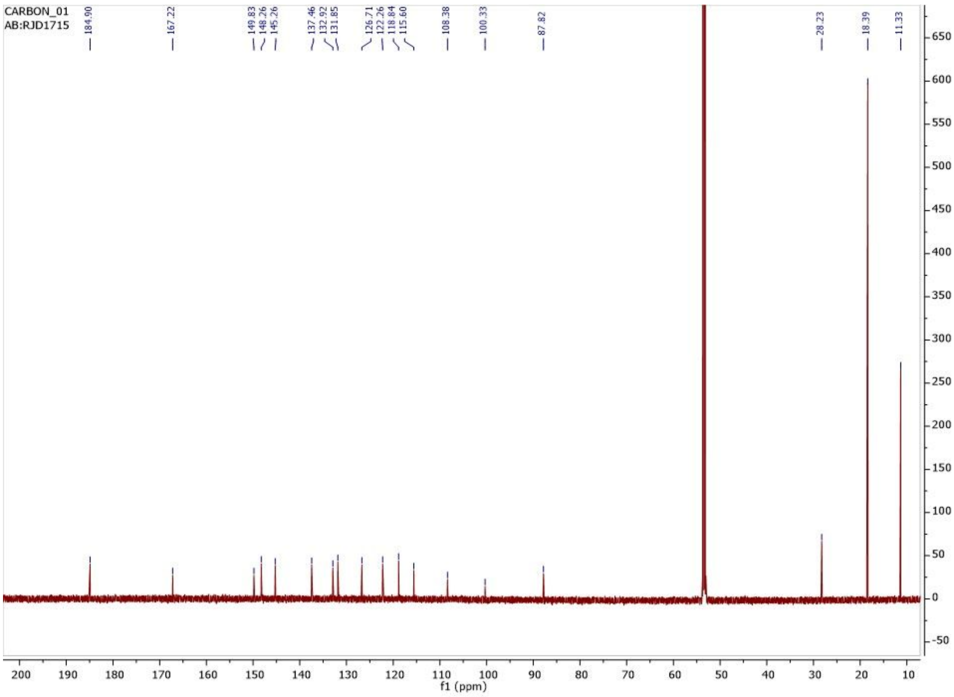

Figure S29. 151 MHz $^{13}C\{^{1}H\}$ NMR spectrum of **7**, recorded in CD_2Cl_2 at 25 ^{0}C.

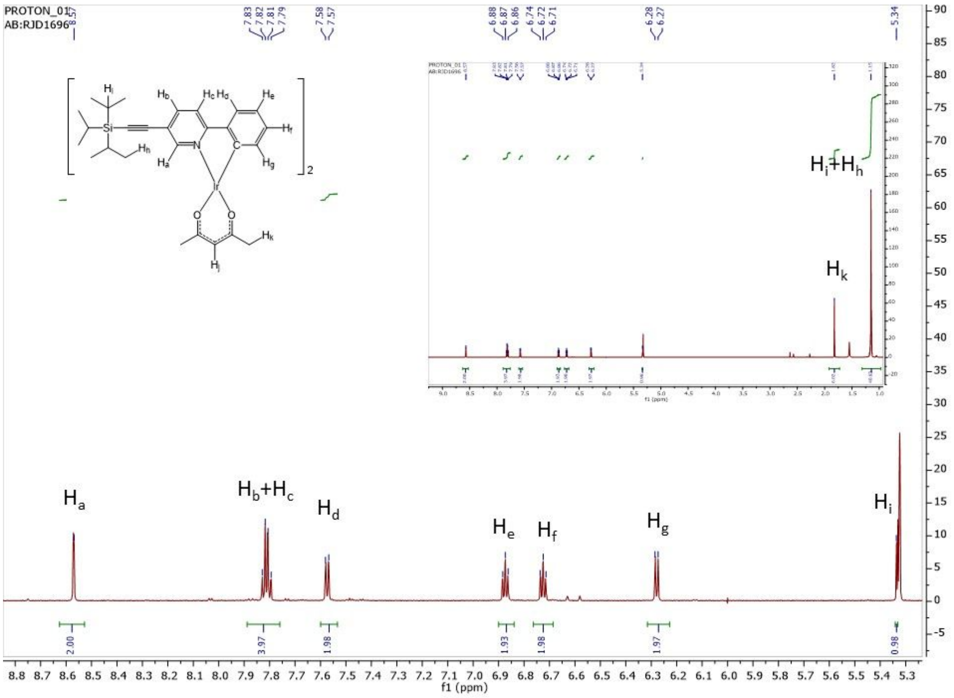

Figure S30. 600 MHz ^{1}H NMR spectrum of **8**, recorded in CD_2Cl_2 at 25 ^{0}C.

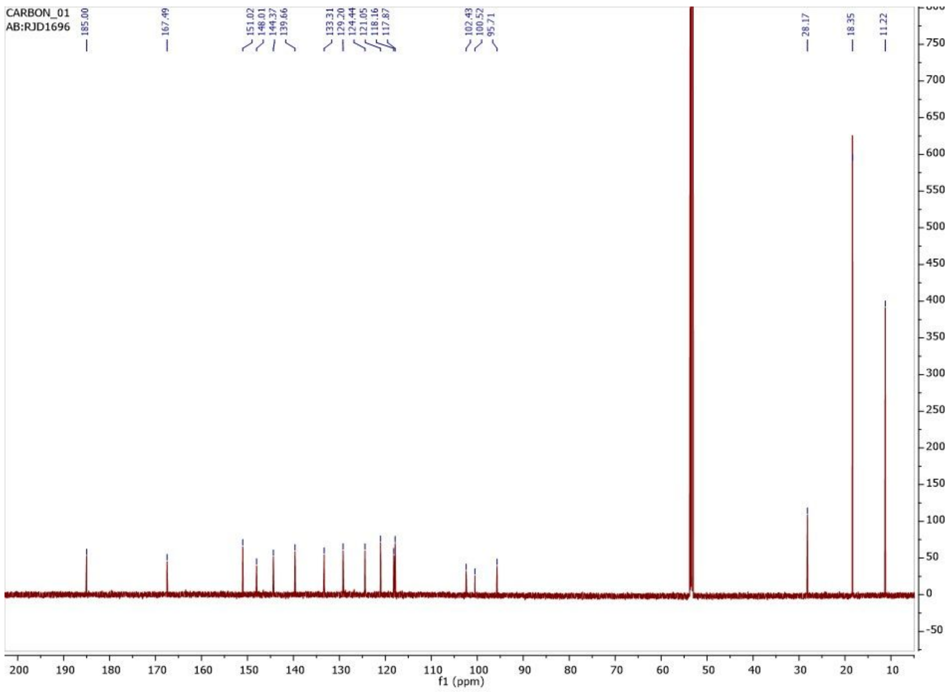

Figure S31. 151 MHz $^{13}C\{^{1}H\}$ NMR spectrum of **8**, recorded in CD_2Cl_2 at 25 ^{0}C.

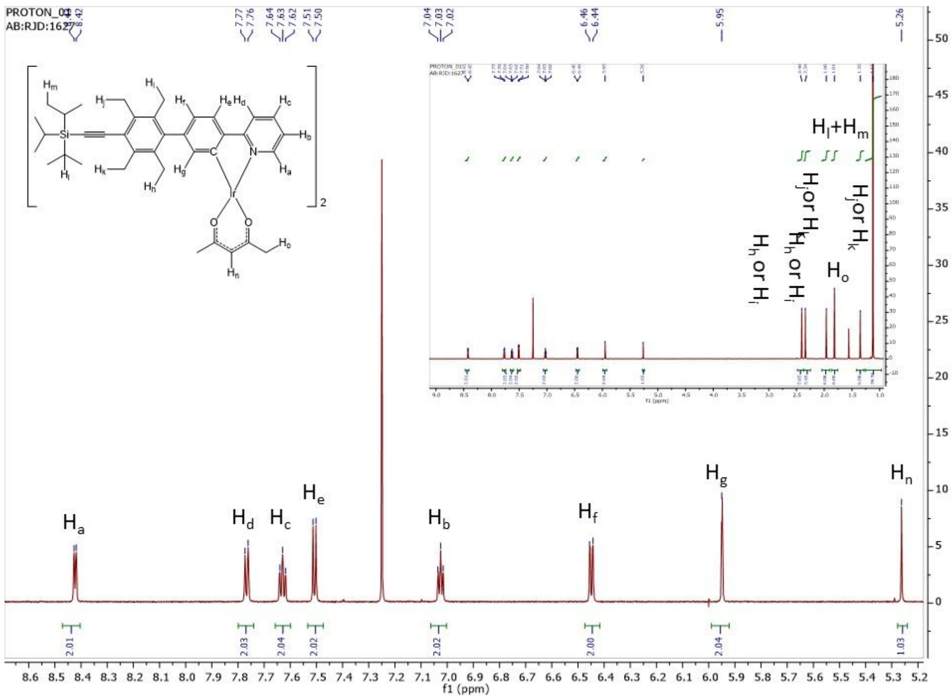

Figure S32. 600 MHz ^{1}H NMR spectrum of **10**, recorded in $CDCl_3$ at 25 ^{0}C.

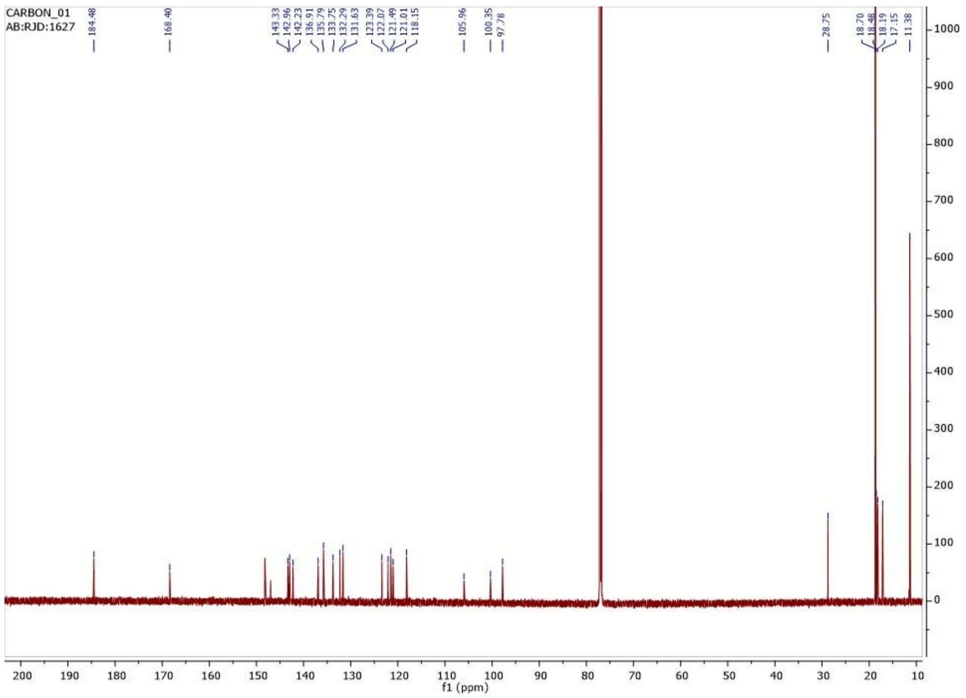

Figure S33. 151 MHz $^{13}C\{^{1}H\}$ NMR spectrum of **10**, recorded in $CDCl_3$ at 25 ^{0}C.

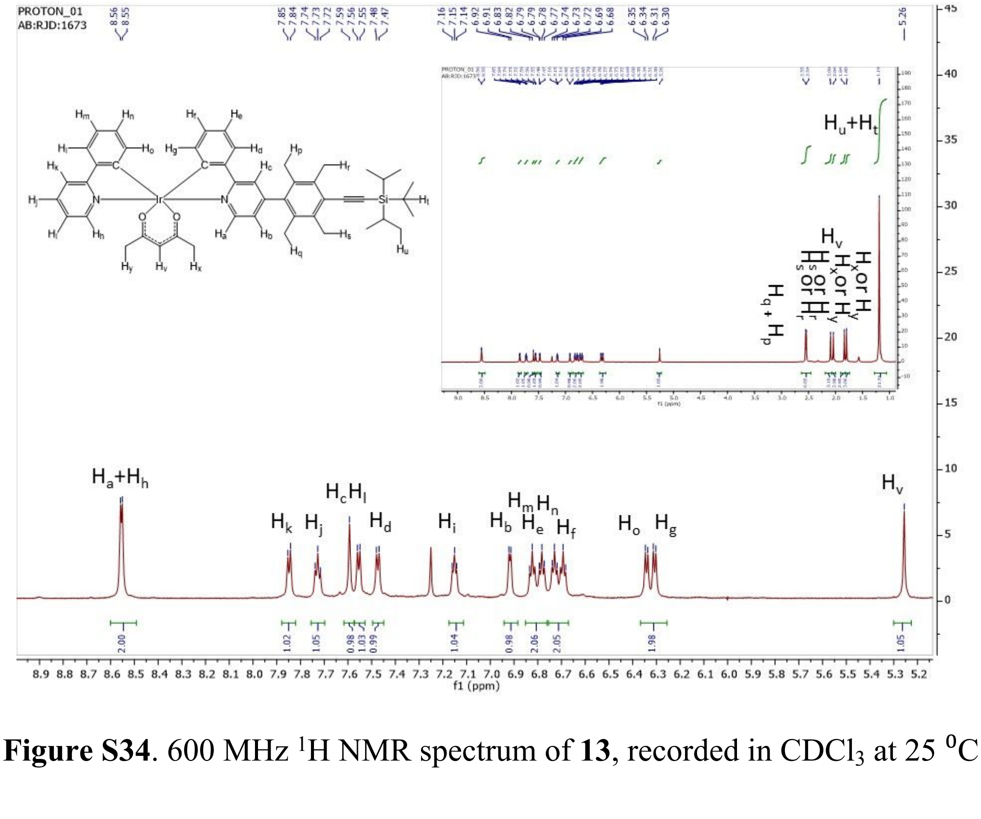

Figure S34. 600 MHz ^{1}H NMR spectrum of **13**, recorded in $CDCl_3$ at 25 ^{0}C.

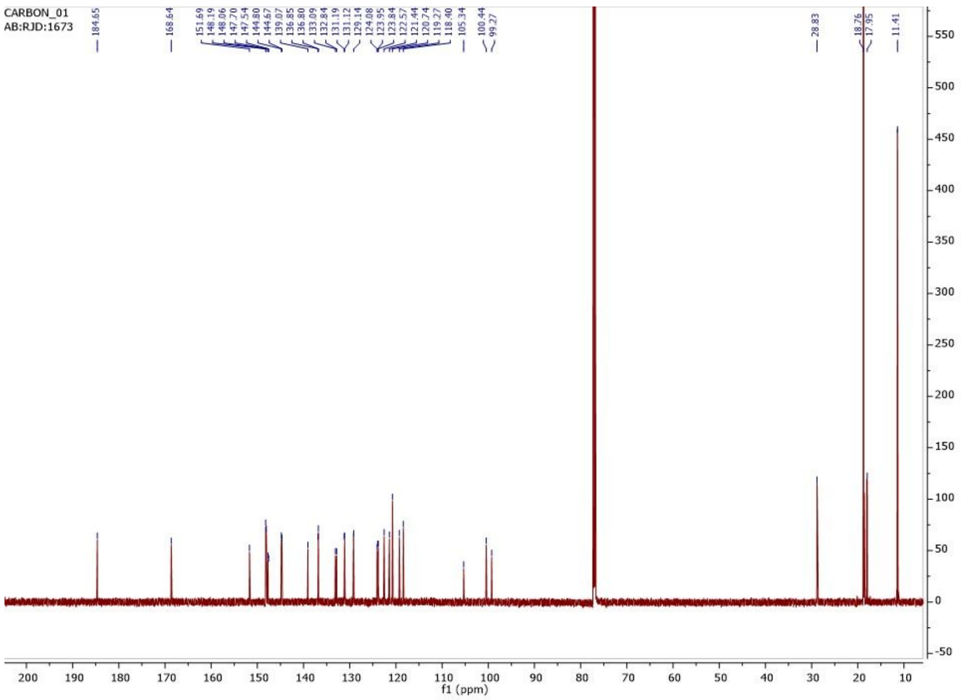

Figure S35. 151 MHz $^{13}C\{^{1}H\}$ NMR spectrum of **13**, recorded in $CDCl_3$ at 25 ^{0}C.

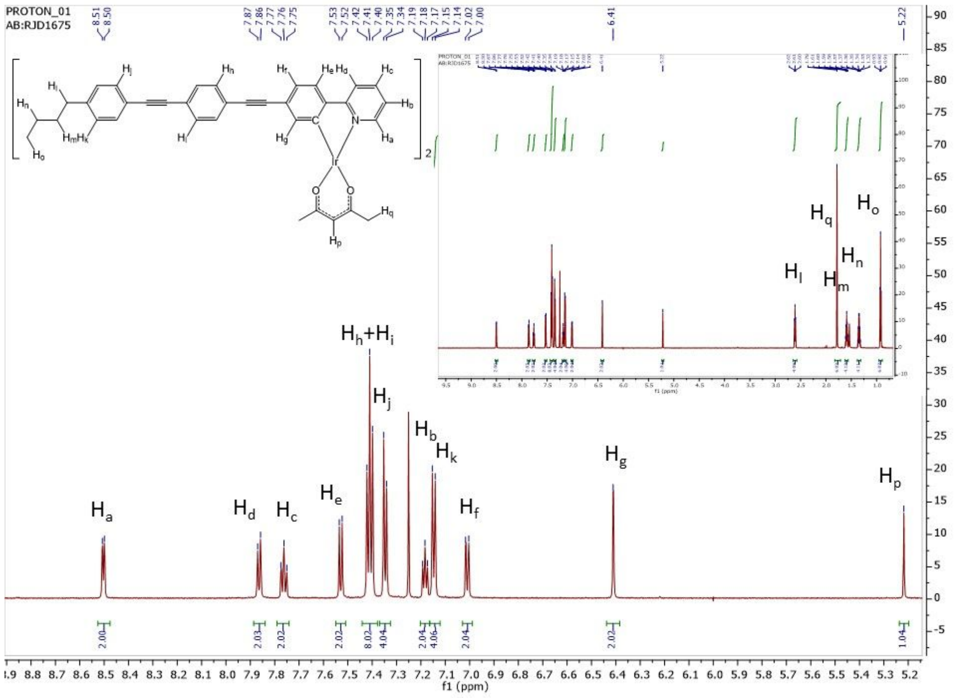

Figure S36. 600 MHz ^{1}H NMR spectrum of **14**, recorded in $CDCl_3$ at 25 ^{0}C.

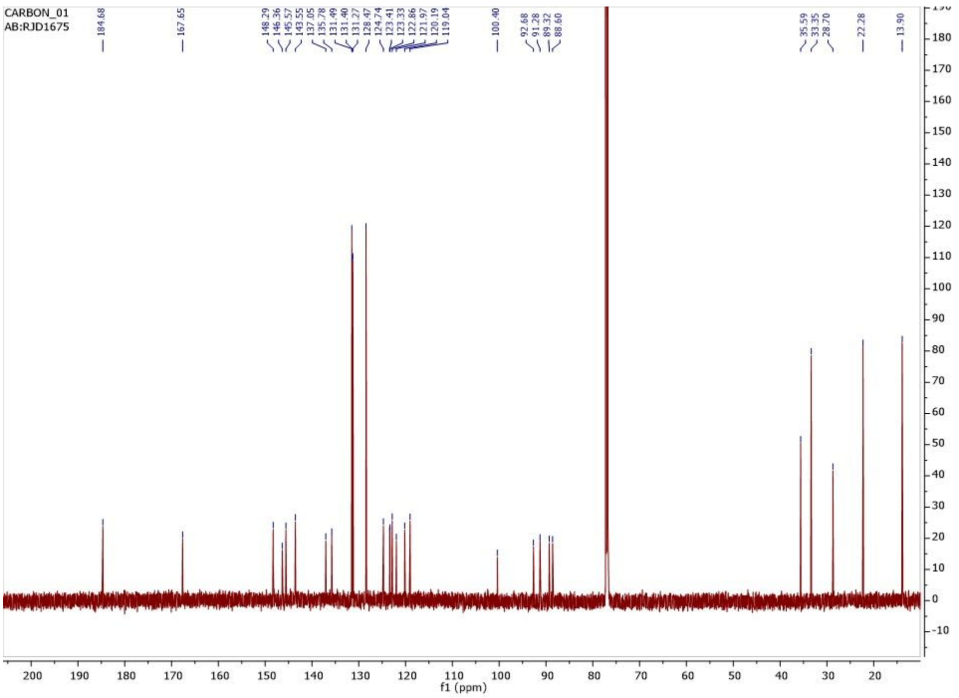

Figure S37. 151 MHz $^{13}C\{^1H\}$ NMR spectrum of **14**, recorded in $CDCl_3$ at 25 ^{0}C.

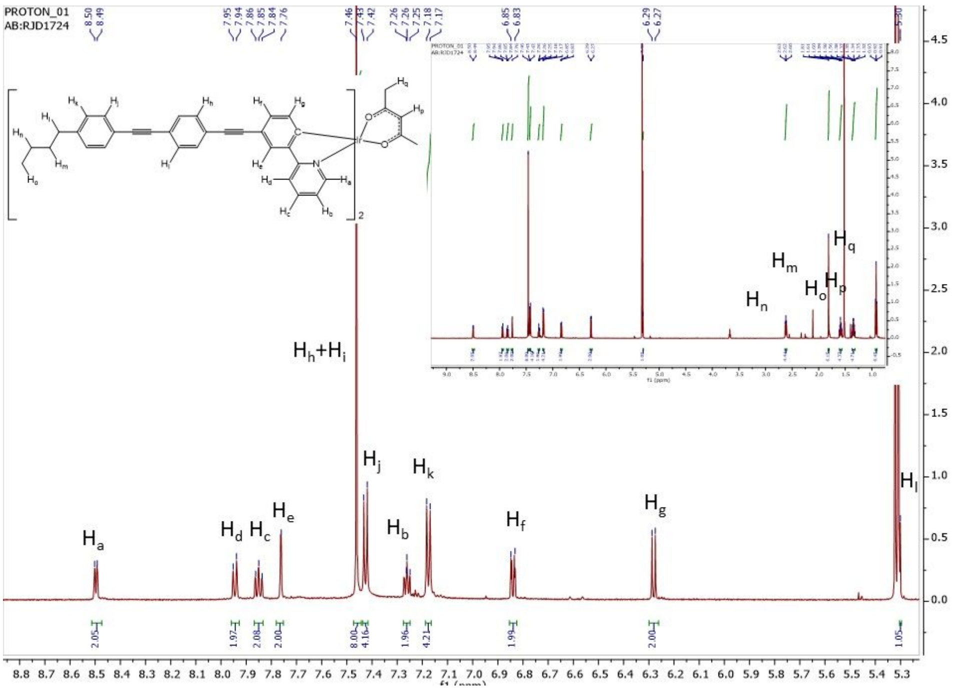

Figure S38. 600 MHz ^1H NMR spectrum of **15**, recorded in $CDCl_3$ at 25 ^{0}C.

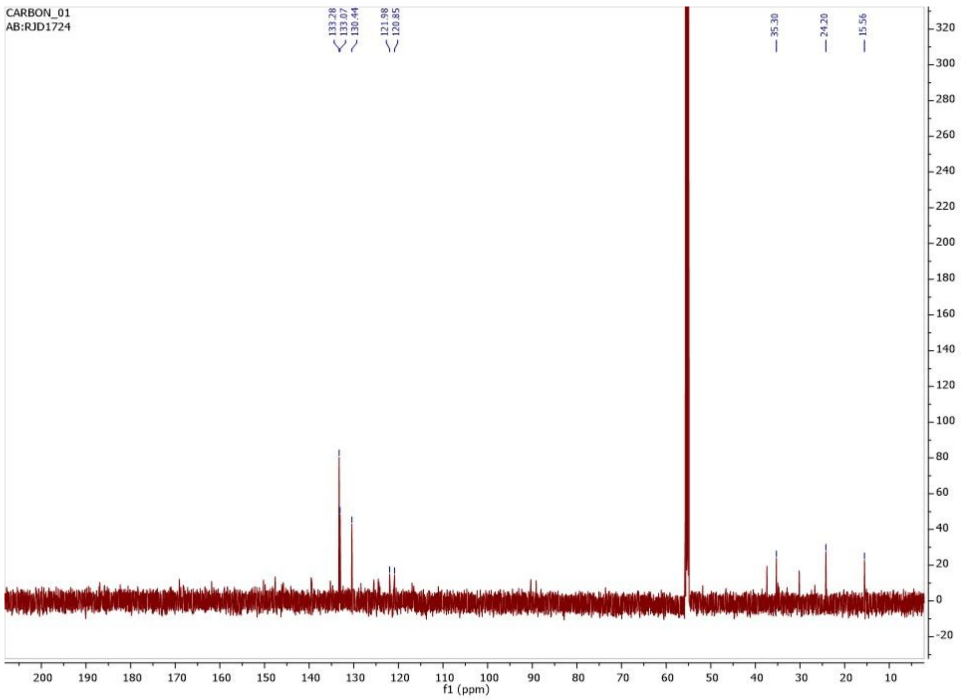

Figure S39. 151 MHz $^{13}C\{^{1}H\}$ NMR spectrum of **15**, recorded in $CDCl_3$ at 25 ^{0}C.

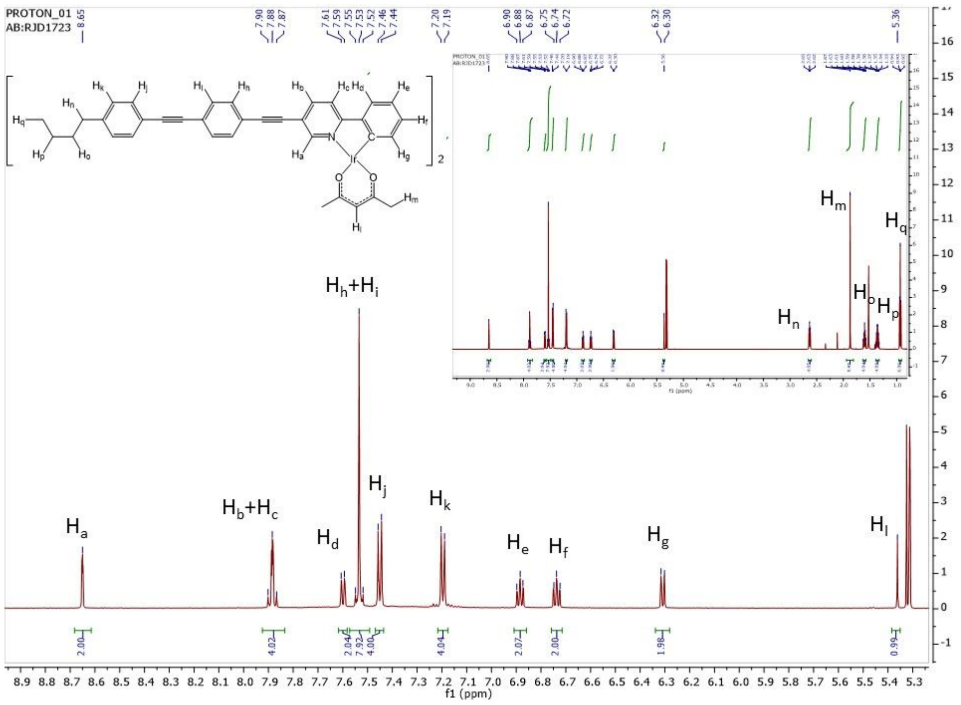

Figure S40. 600 MHz ^{1}H NMR spectrum of **16**, recorded in CD_2Cl_2 at 25 ^{0}C.

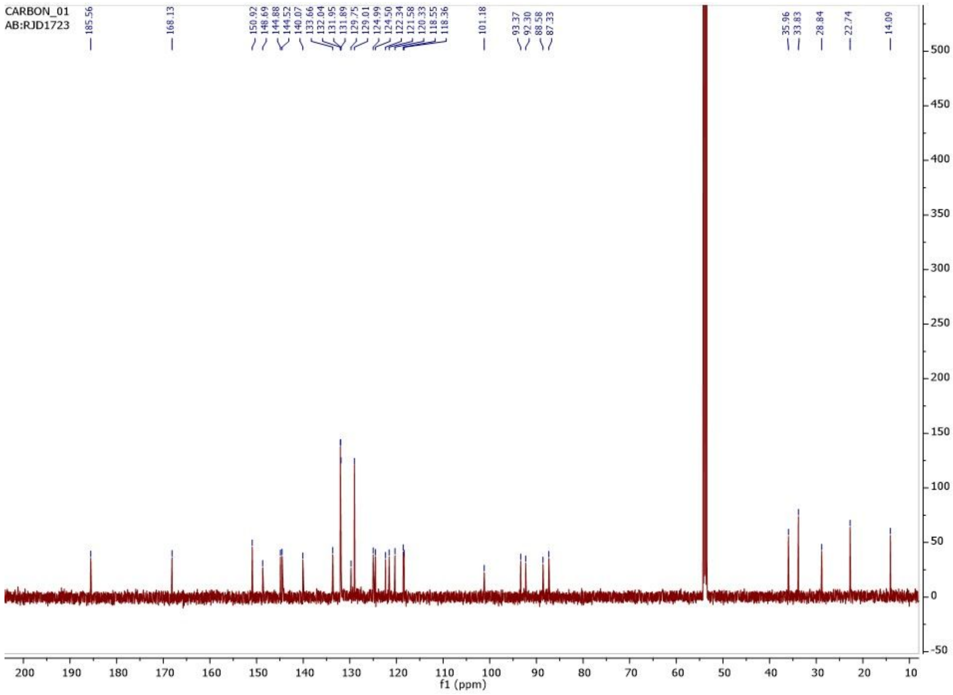

Figure S41. 151 MHz $^{13}C\{^{1}H\}$ NMR spectrum of **16**, recorded in CD_2Cl_2 at 25 ^0C.

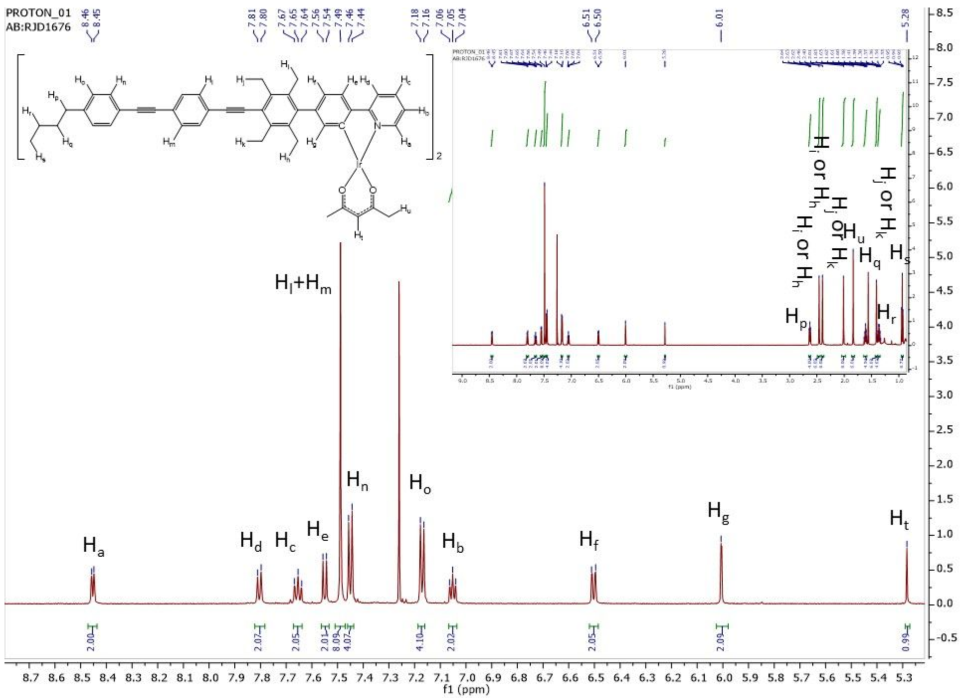

Figure S42. 600 MHz ^{1}H NMR spectrum of **18**, recorded in $CDCl_3$ at 25 ^0C.

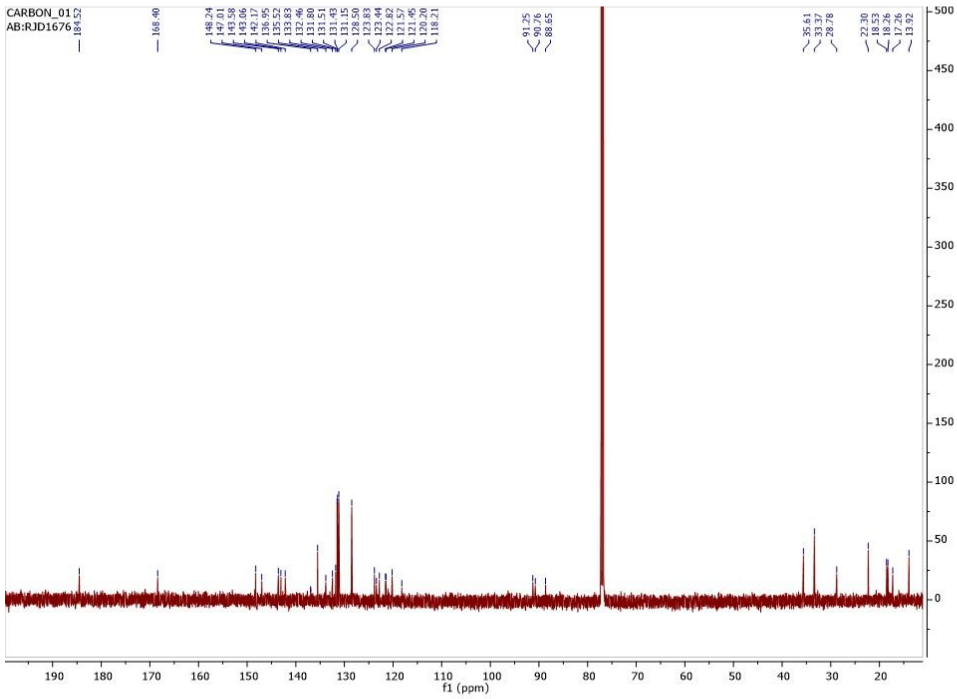

Figure S43. 151 MHz $^{13}C\{^{1}H\}$ NMR spectrum of **18**, recorded in $CDCl_3$ at 25 ^{0}C.

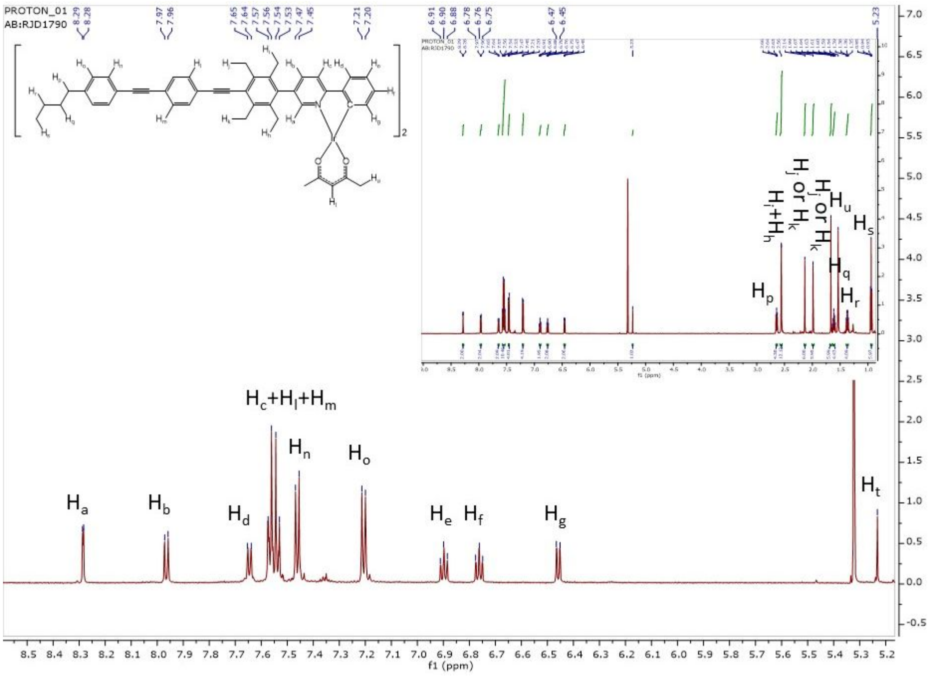

Figure S44. 600 MHz ^{1}H NMR spectrum of **19**, recorded in CD_2Cl_2 at 25 ^{0}C.

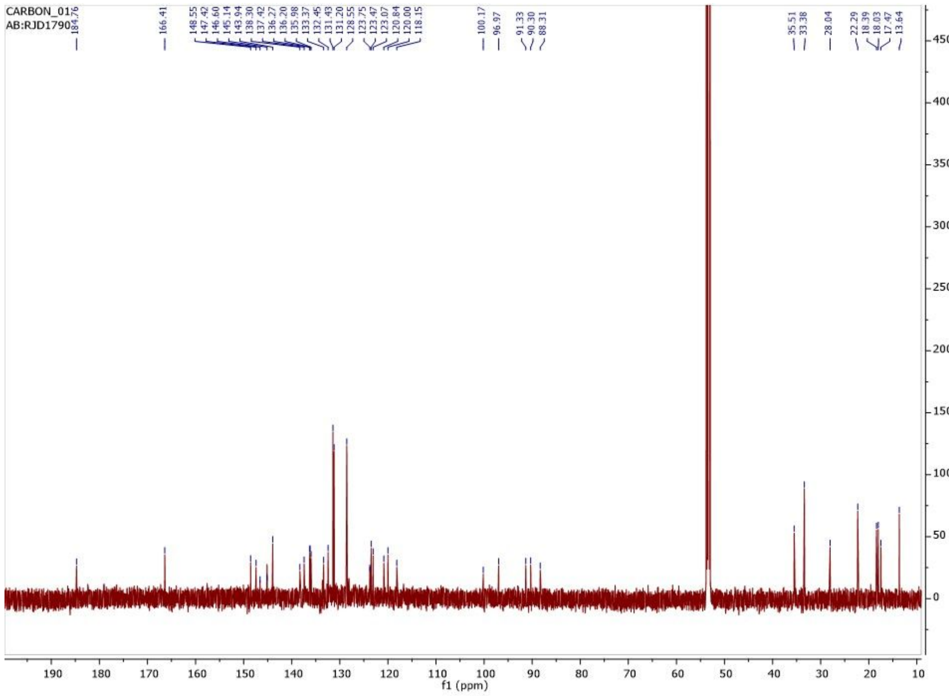

Figure S45. 151 MHz $^{13}C\{^1H\}$ NMR spectrum of **19**, recorded in CD_2Cl_2 at 25 ^0C.

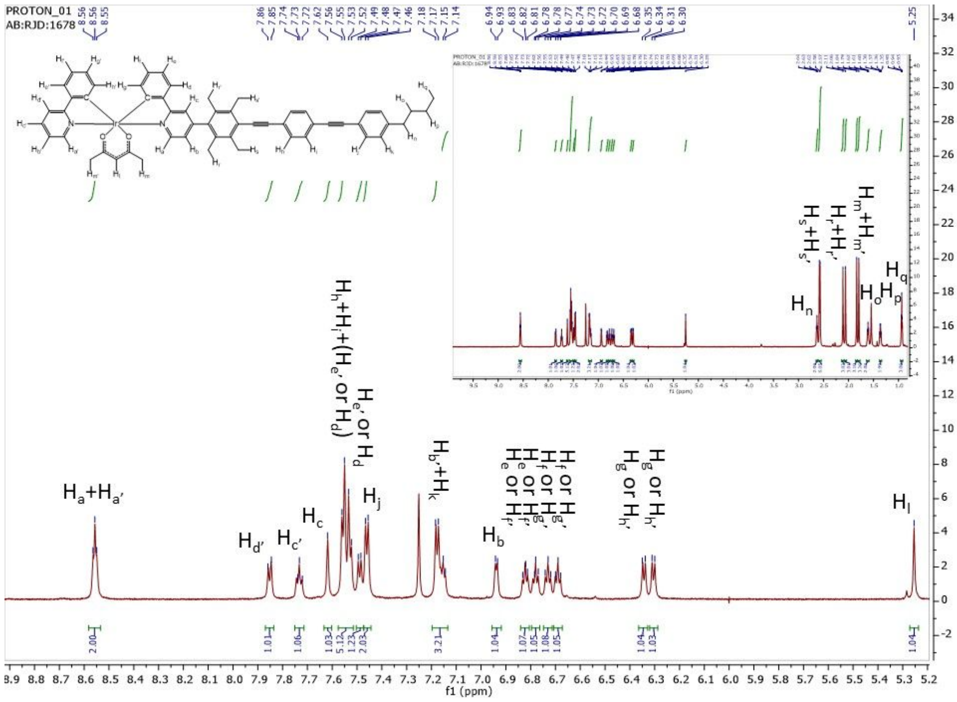

Figure S46. 600 MHz ^1H NMR spectrum of **21**, recorded in $CDCl_3$ at 25 ^0C.

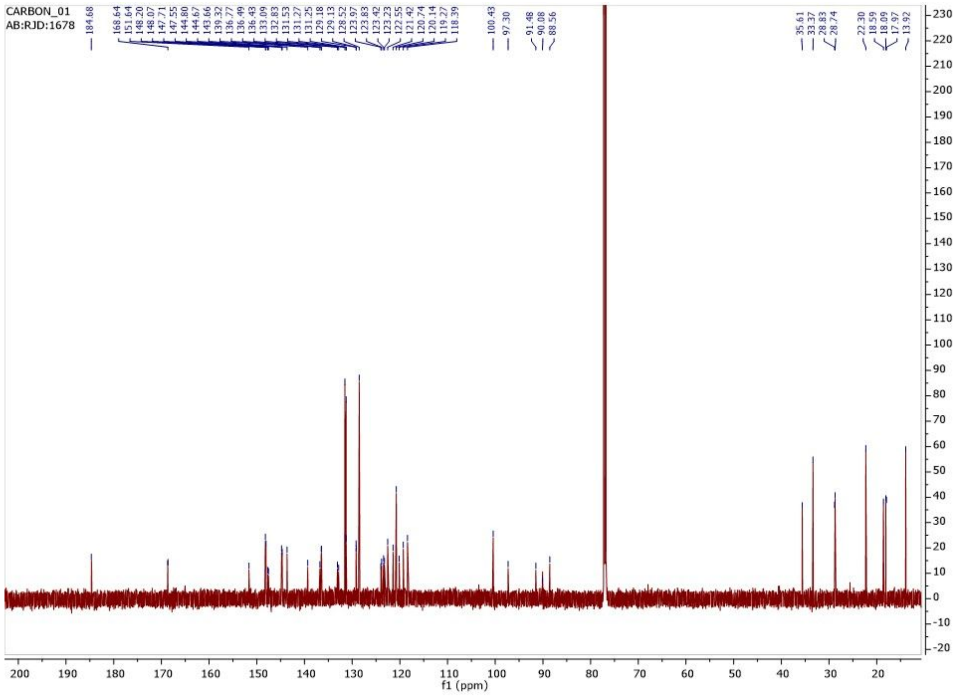

Figure S47. 151 MHz $^{13}C\{^{1}H\}$ NMR spectrum of **21**, recorded in $CDCl_3$ at 25 ^{0}C.

S3. X-ray Crystallography

The X-ray single crystal data have been collected using λMoKα radiation (λ =0.71073Å) on a Bruker D8Venture (Photon100 CMOS detector, IμS-microsource, focusing mirrors; compounds **3**, **6** and **14**) and Agilent XCalibur (Sapphire-3 CCD detector, fine-focus sealed tube, graphite monochromator; compounds **1**, **2** and **10**) diffractometers equipped with Cryostream (Oxford Cryosystems) open-flow nitrogen cryostats at the temperature 120.0(2)K. All structures were solved by direct method and refined by full-matrix least squares on F^2 for all data using Olex2[11] and SHELXTL[12] software. All non-disordered non-hydrogen atoms were refined in anisotropic approximation; all hydrogen atoms were placed in the calculated positions and refined in riding mode. Disordered atoms in structures **2**, **6**, **10** and **14** were refined isotropically with fixed site occupation factors and appropriate restraints, mainly of the bond lengths values. Crystal **10** contains severely disordered solvent molecules (most probably DCM and methanol; total 111 e/unit cell) which could not be properly modeled and refined. Their contribution was taken into account by MASK procedure of Olex2 package. Crystal data and parameters of refinement are listed in Table S1-2. Crystallographic data for the structure have been deposited with the Cambridge Crystallographic Data Centre as supplementary publication CCDC-2207294-2207299.

Table S1. Crystal data and structure refinement for structures **1-3**.

	**1**	**2**	**3**
Empirical formula	$C_{33}H_{27}IrN_2O_2$	$C_{33}H_{23}N_2O_2F_4Ir$	$C_{45}H_{39}IrN_2O_2$ x 2 CH_2Cl_2
Formula weight	675.77	747.73	1001.83
Temperature/K	120.0	120.0	120.0
Crystal system	monoclinic	monoclinic	monoclinic
Space group	$P2_1/n$	$P2_1/c$	$P2_1/n$
a/Å	9.4004(4)	7.42910(13)	21.1740(12)
b/Å	23.7254(10)	23.9757(4)	9.6431(5)
c/Å	11.5148(4)	15.0691(3)	22.6909(12)
α/°	90.00	90.00	90.00
β/°	92.671(3)	96.7654(17)	112.8043(19)
γ/°	90.00	90.00	90.00
Volume/Å^3	2565.33(17)	2665.38(9)	4270.9(4)
Z	4	4	4
$\rho_{calc,}$ g/cm^3	1.750	1.863	1.558
μ/mm^{-1}	5.239	5.073	3.417
F(000)	1328.0	1456.0	2000.0
Reflections collected	28802	42464	92016
Independent reflections, R_{int}	5890 [R_{int} = 0.1243, R_{sigma} = 0.1114]	7780 [R_{int} = 0.0360, R_{sigma} = 0.0266]	12455 [R_{int} = 0.0426, R_{sigma} = 0.0271]
Data/restraints/parameters	5890/0/352	7780/0/380	12455/6/510
Goodness-of-fit on F^2	0.966	1.045	1.023
Final R_1 indexes [I≥2σ (I)]	R_1 = 0.0485, wR_2 = 0.0722	R_1 = 0.0202, wR_2 = 0.0440	R_1 = 0.0241, wR_2 = 0.0498
Final wR_2 indexes [all data]	R_1 = 0.0883, wR_2 = 0.0853	R_1 = 0.0248, wR_2 = 0.0456	R_1 = 0.0346, wR_2 = 0.0533
Largest diff. peak/hole / e Å^{-3}	2.19/-1.53	0.9/-0.7	1.16/-1.22

Table S2. Crystal data and structure refinement for structures **6**, **10**, and**14**.

	**6**	**10**	**14**
Empirical formula	$C_{49}H_{63}IrN_2O_2Si_2$	$C_{69}H_{87}IrN_2O_2Si_2$	$C_{67}H_{55}IrN_2O_2$
Formula weight	960.39	1224.78	1112.33
Temperature/K	120.0	120.0	120.0
Crystal system	triclinic	triclinic	triclinic
Space group	P-1	P-1	P-1
a/Å	15.4768(16)	12.8672(6)	7.8098(5)
b/Å	16.8918(18)	16.4131(6)	19.1703(13)
c/Å	18.818(2)	18.8744(8)	19.5022(13)
α/°	77.173(4)	66.166(4)	115.593(2)
β/°	76.040(4)	75.268(4)	100.820(2)
γ/°	89.145(4)	67.770(4)	94.795(2)
Volume/$Å^3$	4651.3(9)	3350.9(3)	2541.4(3)
Z	4	2	2
$\rho_{calc,}$ g/cm^3	1.371	1.214	1.454
μ/mm^{-1}	2.961	2.069	2.676
F(000)	1968.0	1272.0	1128.0
Reflections collected	91814	53823	52919
Independent reflections, R_{int}	24699 [R_{int} = 0.0840, R_{sigma} = 0.0851]	19528 [R_{int} = 0.0626, R_{sigma} = 0.0811]	13527 [R_{int} = 0.0619, R_{sigma} = 0.0753]
Data/restraints/parameters	24699/40/1021	19528/15/705	13527/1/643
Goodness-of-fit on F^2	1.132	1.009	1.008
Final R_1 indexes [I≥2σ (I)]	R_1 = 0.0842, wR_2 = 0.2162	R_1 = 0.0435, wR_2 = 0.0861	R_1 = 0.0398, wR_2 = 0.0783
Final wR_2 indexes [all data]	R_1 = 0.1275, wR_2 = 0.2425	R_1 = 0.0583, wR_2 = 0.0917	R_1 = 0.0617, wR_2 = 0.0829
Largest diff. peak/hole / e $Å^{-3}$	7.16/-5.45	1.00/-0.97	1.68/-1.25

S4. Substituted acac complex photodegradation

The photodegradation experienced by the substituted complexes **3** and **5** was investigated using and ^{1}H NMR study. Complex **3** was chosen for the study as the photodegradation as its NMR spectra was simpler than that of **5**, it was soluble in a greater range of solvents than **5** and its degradation time scale occurred over a longer time scale simplifying measurements. C_6D_6 was chosen for the NMR solvent because it is a non-coordinating, non-polar, aprotic solvent that is photostable and is capable of dissolving complex **3**, such criteria was used to eliminate any potential reactions with the solvent. The solution was irradiated by a xenon lamp (300 W) with a UV cut-off filter and ^{1}H NMR spectra recorded at regular intervals (see Figures S48 and S49).

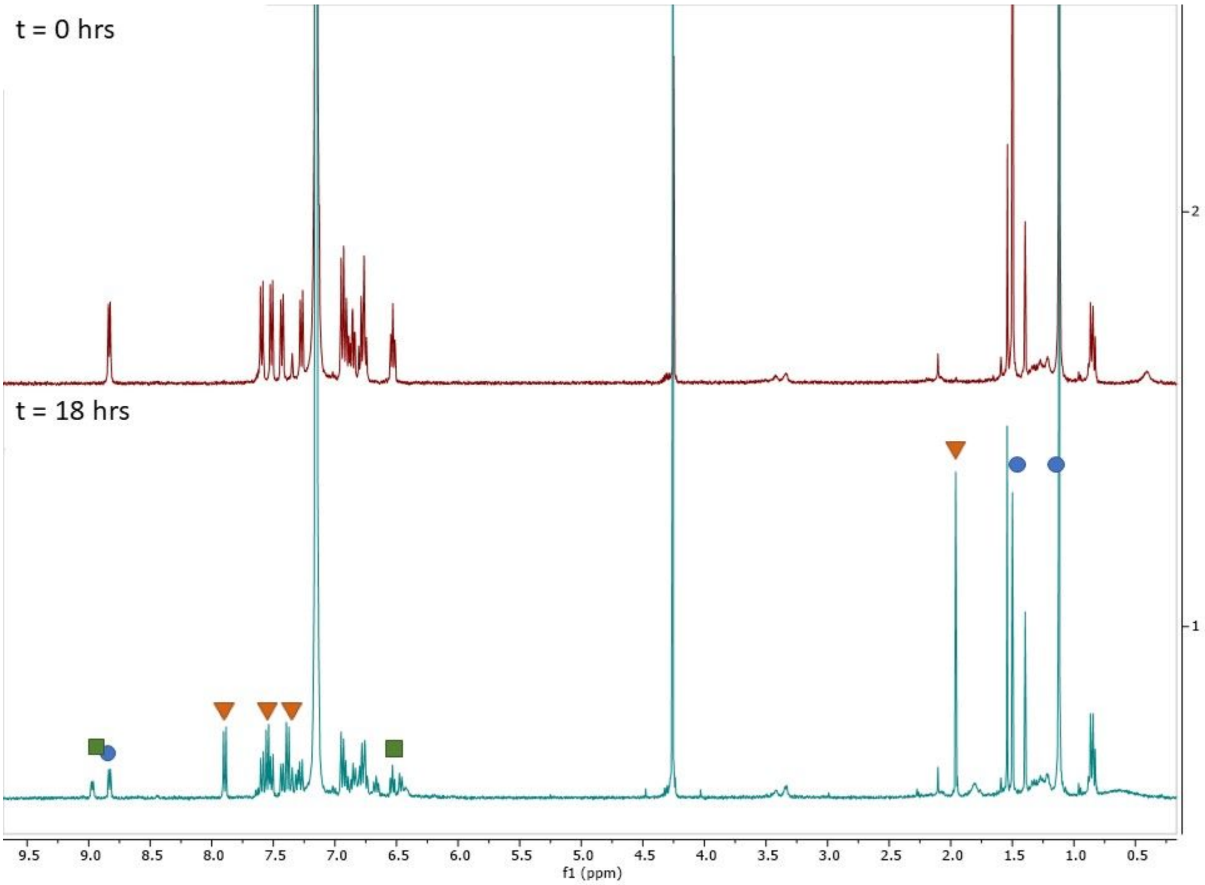

Figure S48. 400 MHz ^{1}H spectra acquired at different time intervals to study the photodegradation of complex **3** recorded in C_6D_6: blue circles = complex **3** signals, red triangles = free **L**^{2}H signals, and green squares = unidentified species signals.

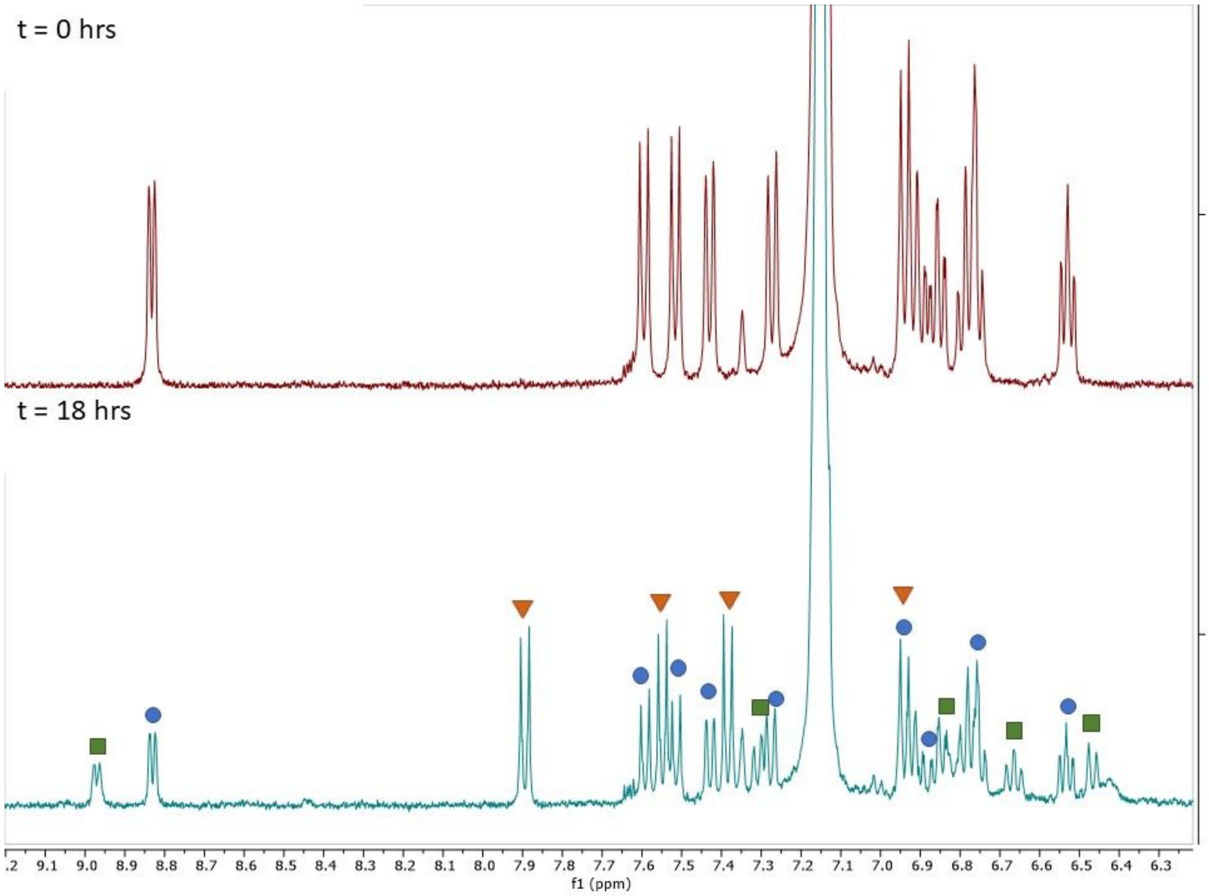

Figure S49. 400 MHz ^1H spectra acquired at time intervals Photodegradation of complex **3** recorded in C_6D_6: blue circles = complex **3** signals, red triangles = free **L**^{2}H signals, and green squares = unidentified species signals.

The NMR study shows the growth of signals associated with the free **L**^{2}H indicating the photodegradation occurs through the dissociation of the acac ligand and subsequent formation of a new iridium complex that appears to have ppy ligands coordinated. It is unlikely that a Ir(ppy)$_2$ species would remain stable with two ligand vacancies therefore it would readily coordinate to any ligating species. Benzene has limited coordination ability therefore it is proposed that with the water present in the solution a Ir(ppy)$_2$(H_2O)$_2$ or [Ir(OH)(ppy)$_2$]$_2$ complex is formed (see Figure S50).

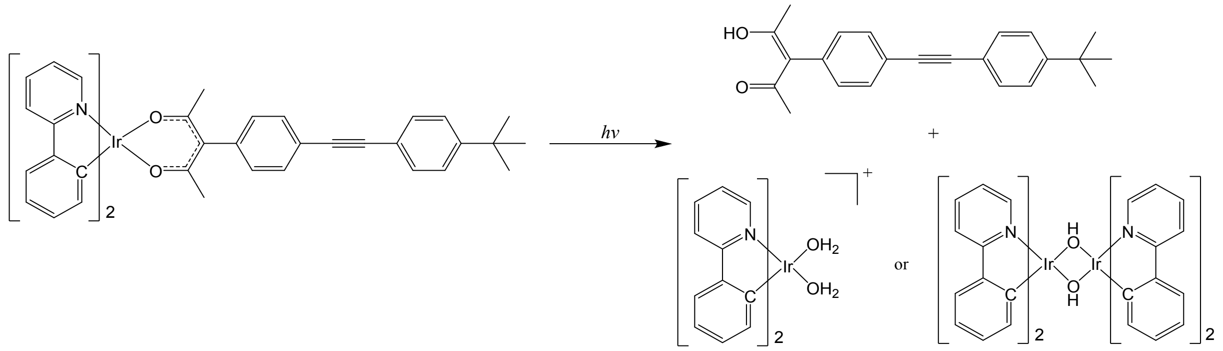

Figure S50. Proposed photodegradation of complex **3**.

References

1. Xiao, D.; Martini, L. A.; Snoeberger, R. C.; Crabtree, R. H.; Batista, V. S. Inverse Design and Synthesis of acac-Coumarin Anchors for Robust TiO2 Sensitization. *Journal of the American Chemical Society* **2011,** 133 (23), 9014-9022 DOI: 10.1021/ja2020313.
2. Bessho, T.; Yoneda, E.; Yum, J.-H.; Guglielmi, M.; Tavernelli, I.; Imai, H.; Rothlisberger, U.; Nazeeruddin, M. K.; Grätzel, M. New Paradigm in Molecular Engineering of Sensitizers for Solar Cell Applications. *Journal of the American Chemical Society* **2009,** 131 (16), 5930-5934 DOI: 10.1021/ja9002684.
3. Lowry, M. S.; Hudson, W. R.; Pascal, R. A.; Bernhard, S. Accelerated Luminophore Discovery through Combinatorial Synthesis. *Journal of the American Chemical Society* **2004,** 126 (43), 14129-14135 DOI: 10.1021/ja047156+.
4. Coppo, P.; Plummer, E. A.; De Cola, L. Tuning iridium(iii) phenylpyridine complexes in the “almost blue” region. *Chemical Communications* **2004,** (15), 1774-1775 DOI: 10.1039/B406851C.
5. You, Y.; An, C.-G.; Lee, D.-S.; Kim, J.-J.; Park, S. Y. Silicon-containing dendritic tris-cyclometalated Ir(iii) complex and its electrophosphorescence in a polymer host. *Journal of Materials Chemistry* **2006,** 16 (48), 4706-4713 DOI: 10.1039/B611288A.
6. Sandee, A. J.; Williams, C. K.; Evans, N. R.; Davies, J. E.; Boothby, C. E.; Köhler, A.; Friend, R. H.; Holmes, A. B. Solution-Processible Conjugated Electrophosphorescent Polymers. *Journal of the American Chemical Society* **2004,** 126 (22), 7041-7048 DOI: 10.1021/ja039445o.
7. Lee, K. H.; Kang, H. J.; Park, J. K.; Seo, J. H.; Kim, Y. K.; Yoon, S. S. Red-phosphorescent OLEDs employing iridium (III) complexes based on 5-benzoyl-2-phenylpyridine derivatives. *Thin Solid Films* **2010,** 518 (22), 6188-6194 DOI: https://doi.org/10.1016/j.tsf.2010.03.146.
8. Shao, S.; Ma, Z.; Ding, J.; Wang, L.; Jing, X.; Wang, F. Spiro-Linked Hyperbranched Architecture in Electrophosphorescent Conjugated Polymers for Tailoring Triplet Energy Back Transfer. *Advanced Materials* **2012,** 24 (15), 2009-2013 DOI: https://doi.org/10.1002/adma.201104544.
9. Tobisu, M.; Takahira, T.; Ohtsuki, A.; Chatani, N. Nickel-Catalyzed Alkynylation of Anisoles via C–O Bond Cleavage. *Organic Letters* **2015,** 17 (3), 680-683 DOI: 10.1021/ol503707m.

10. Davidson, R.; Hsu, Y.-T.; Griffiths, G. C.; Li, C.; Yufit, D.; Pal, R.; Beeby, A. Highly Linearized Twisted Iridium(III) Complexes. *Inorganic Chemistry* **2018,** 57 (22), 14450-14462 DOI: 10.1021/acs.inorgchem.8b02818.
11. Dolomanov, O. V.; Bourhis, L. J.; Gildea, R. J.; Howard, J. A. K.; Puschmann, H. OLEX2: a complete structure solution, refinement and analysis program. *Journal of Applied Crystallography* **2009,** 42, 339-341 DOI: 10.1107/s0021889808042726.
12. Sheldrick, G. M. *SHELXL: Suite of Programs for Crystal Structure Analysis*, Tammanstrasse 4: Gottingen, 1998.
