# Supplementary material for: Tuning Emission Lifetimes of Ir(C^N)2(acac) Complexes with Oligo(phenyleneethynylene) Groups
Source: Inorg Chem. 2023 Jan 27;62(6):2793–805. doi: 10.1021/acs.inorgchem.2c03934 (PMC9930119; doi:10.1021/acs.inorgchem.2c03934)
Supplement: Supplementary file 2 — ic2c03934_si_002.pdf [file ic2c03934_si_002.pdf]

Supporting Information - Photophysics

Tuning Emission Lifetimes of Ir(C^N)$_2$(acac) Complexes with Oligo(phenyleneethynylene) Groups

Ross Davidson,[†]* Yu-Ting Hsu,[†] Mark A. Fox,[†] Juan A. Aguilar,[†] Dmitry Yufit[†], Andrew Beeby[†]*

†Department of Chemistry, University of Durham, South Road, Durham, DH1 3LE, England, UK

*To whom correspondence should be addressed. Email: Ross Davidson (ross.davidson@durham.ac.uk) and Andrew Beeby (andrew.beeby@durham.ac.uk).

Table of Contents

S5 Computational Study …………………………………………………… S53

Orbital Contributions ……………….……………………………... S55

Electronic absorbance assignment ……………………………... S73

Singlet and triplet state energies ……………………………...… S91

Transition dipole moments ……………………………………….. S95

S6. Physical Measurements ……………………………………………. S99

Electrochemical data ……………………………………………… S98

UV-Visible electronic absorbance ……………………………… S101

Steady-state emission ……………………………………………... S104

Low temperature emission ……………………………..……… S107

Excitation dependence …………………………………………….. S110

Lifetime trace …………………………………………………….. S120

OPE3 substituted complex excitation ………………………… S132

Time resolved spectra ……………………………………………... S134

References ……………………………………………………………... S140

S5 Computational Study

Density functional theory (DFT) calculations were carried out using the Gaussian 09 package (Gaussian, Inc)[1], all results were displayed using GaussView[2] and GaussSum[3]. For the sake of computational efficiency, the complexes containing either triisopropylsilyl or butyl groups were modelled using trimethylsilyl or methyl groups respectively. All structures were geometrically optimised in a DCM solvent field using the IEF-PCM method.

A comparison was made between the calculated and measured T_1 energies of the complexes used in this study for three model chemistries B3LYP/3-21G*:LANL2DZ, B3LYP/6-31G(d):LANL2DZ and CAM-B3LYP/6-31G(d):LANL2DZ. B3LYP/3-21G*:LANL2DZ had the lowest mean absolute error (m.a.e.) of 0.0818 eV between measured and calculated values (Table S3) so all calculations here employed this model chemistry.

Table S3. A comparison of the $\Delta E = |T_1^{measured} - T_1^{calculated}|$ for different computational methods.

**Complex**	**Method**		
	B3LYP 3-21G*:LANL2DZ	B3LYP 6-31G(d):LANL2DZ	CAM-B3LYP 6-31G(d):LANL2DZ
**1**	0.0622	0.0324	0.2843
**2**	0.1342	0.0858	0.2734
**3**	0.1348	0.1024	0.2012
**4**	0.1238	0.0036	0.0213
**6**	0.0979	0.0679	
**7**	0.0621	0.0630	
**8**	0.0756	0.0622	
**9**	0.0910	0.0750	
**10**	0.1318	0.1039	
**12**	0.1276	0.1048	
**13**	0.1523	0.1294	
**14**	0.0200	0.1228	
**15**	0.0334	0.1381	
**16**	0.0503	0.1177	
**17**	0.0492	0.0004	
**18**	0.0164	0.1361	
**19**	0.0316	0.1567	
**20**	0.0301	0.1500	
**21**	0.0490	0.1694	
**m.a.e.**	0.0818	0.1012	0.1950

Orbital Contributions

Using GaussSum[3] and the 'groups' method the orbital contribution by each of molecular components was determined, see table S4-S24 for the summarised results.

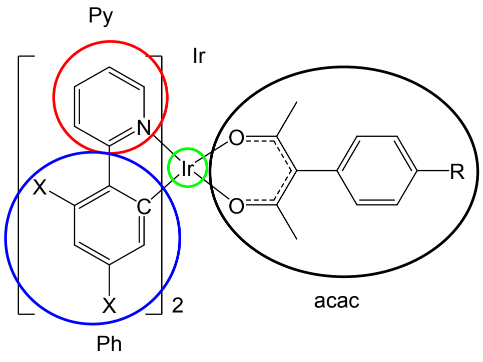

Figure S51. Molecular components by group.

Table S4. Orbital contributions for **1**

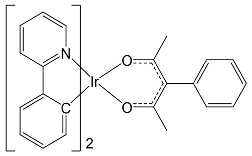

Orbital	Energy (eV)	Symmetry	Molecular Components Contributions (%)			
			Ir	acac	Ph	Py
L+3	-0.95	B	2	0	12	86
L+2	-0.97	A	2	84	3	11
L+1	-1.48	A	4	2	24	70
LUMO	-1.51	B	5	1	28	67
HOMO	-5.11	A	47	7	40	6
H-1	-5.45	B	40	50	3	7
H-2	-5.98	A	70	7	10	13
H-3	-6.18	B	1	3	72	24

Table S5. Orbital contributions for **2**

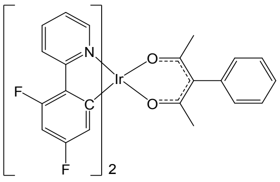

Orbital	Energy (eV)	Symmetry	Molecular Components Contributions (%)			
			Ir	acac	Ph	Py
L+3	-1.01	B	2	0	12	85
L+2	-1.08	A	2	93	1	3
L+1	-1.62	A	4	3	24	70
LUMO	-1.65	B	5	1	27	67
HOMO	-5.47	A	48	8	38	6
H-1	-5.65	B	35	57	3	6
H-2	-6.24	A	61	8	17	14
H-3	-6.37	B	2	9	58	31

Table S6. Orbital contributions for **3**

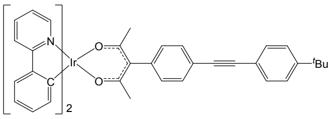

Orbital	Energy (eV)	Symmetry	Molecular Components Contributions (%)			
			Ir	acac	Ph	Py
L+3	-0.99	A	2	89	2	7
L+2	-1.43	A	0	99	0	1
L+1	-1.50	A	4	2	24	70
LUMO	-1.52	A	5	2	28	66
HOMO	-5.13	A	47	6	41	6
H-1	-5.49	A	41	49	4	7
H-2	-5.89	A	0	99	0	0
H-3	-6.01	A	70	6	11	13

Table S7. Orbital contributions for **4**

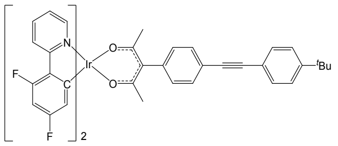

Orbital	Energy (eV)	Symmetry	Molecular Components Contributions (%)			
			Ir	acac	Ph	Py
L+3	-1.12	A	2	93	1	3
L+2	-1.45	A	0	100	0	0
L+1	-1.63	A	4	2	24	69
LUMO	-1.66	A	5	1	28	67
HOMO	-5.49	A	48	8	38	6
H-1	-5.68	A	36	55	3	6
H-2	-5.91	A	0	99	0	0
H-3	-6.25	A	61	7	18	14

Table S8. Orbital contributions for **5**

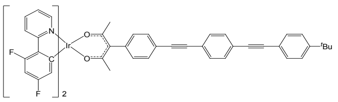

Orbital	Energy (eV)	Symmetry	Molecular Components Contributions (%)			
			Ir	acac	Ph	Py
L+3	-1.13	A	2	93	1	3
L+2	-1.63	A	4	2	24	69
L+1	-1.66	A	5	1	28	67
LUMO	-1.96	A	0	100	0	0
HOMO	-5.49	A	48	7	38	6
H-1	-5.69	A	33	58	3	6
H-2	-5.74	A	3	97	0	0
H-3	-6.26	A	61	7	18	14

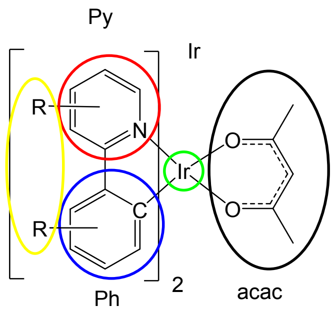

Figure S52. Molecular components by group.

Table S9. Orbital contributions for **6**

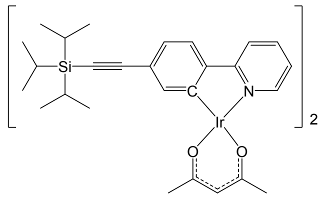

**Orbital**	**Energy (eV)**	**Symmetry**	**Molecular Components Contributions (%)**				
			**Ir**	**acac**	**Ph**	**Py**	**R**
L+3	-1.08	B	3	0	8	86	2
L+2	-1.12	A	1	83	2	12	1
L+1	-1.77	A	4	2	32	53	10
LUMO	-1.81	B	3	0	36	49	11
HOMO	-5.27	A	47	6	39	6	1
H-1	-5.59	B	42	45	5	7	1
H-2	-6.05	A	50	6	22	12	10
H-3	-6.05	B	0	4	51	19	25

Table S10. Orbital contributions for **7**.

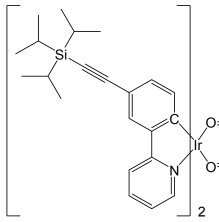

Orbital	Energy (eV)	Symmetry	Molecular Components Contributions (%)				
			Ir	acac	Ph	Py	R
L+3	-1.06	B	2	0	15	81	1
L+2	-1.13	A	2	92	2	4	0
L+1	-1.62	A	4	3	25	68	0
LUMO	-1.63	B	5	1	30	64	0
HOMO	-5.20	A	39	5	38	5	12
H-1	-5.64	B	42	48	4	7	0
H-2	-6.02	B	1	1	60	7	31
H-3	-6.15	A	71	7	9	13	0

Table S11. Orbital contributions for **8**.

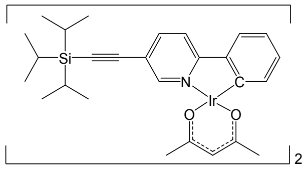

Orbital	Energy (eV)	Symmetry	Molecular Components Contributions (%)				
			Ir	acac	Ph	Py	R
L+3	-1.17	B	4	0	3	90	3
L+2	-1.18	A	1	24	1	69	5
L+1	-1.87	A	3	1	22	60	14
LUMO	-1.91	B	3	0	24	58	14
HOMO	-5.19	A	46	6	40	6	2
H-1	-5.59	B	42	46	4	7	0
H-2	-6.06	A	61	7	15	14	3
H-3	-6.12	B	0	4	53	28	15

Table S12. Orbital contributions for **9**

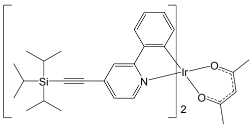

Orbital	Energy (eV)	Symmetry	Molecular Components Contributions (%)				
			Ir	acac	Ph	Py	R
L+3	-1.10	A	1	19	21	56	2
L+2	-1.21	B	1	0	29	67	4
L+1	-1.98	B	5	1	10	65	19
LUMO	-1.99	A	4	1	9	66	20
HOMO	-5.20	A	47	6	41	6	0
H-1	-5.56	B	43	43	4	8	2
H-2	-6.01	A	65	6	9	14	5
H-3	-6.26	B	1	3	74	22	0

Table S13. Orbital contributions for **10**.

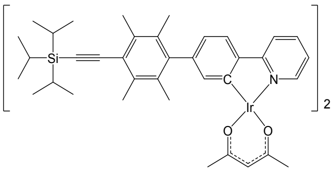

Orbital	Energy (eV)	Symmetry	Molecular Components Contributions (%)				
			Ir	acac	Ph	Py	R
L+3	-0.97	B	2	0	12	85	1
L+2	-1.06	A	2	92	2	4	0
L+1	-1.52	A	4	3	24	68	1
LUMO	-1.54	B	4	1	29	65	1
HOMO	-5.16	A	47	6	40	6	0
H-1	-5.53	B	44	43	4	8	1
H-2	-5.97	A	56	6	9	10	19
H-3	-6.07	B	0	2	3	0	95

Table S14. Orbital contributions for **11**.

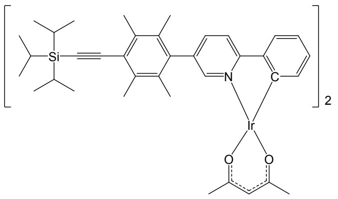

Orbital	Energy (eV)	Symmetry	Molecular Components Contributions (%)				
			Ir	acac	Ph	Py	R
L+3	-1.03	B	2	0	12	82	4
L+2	-1.05	A	2	86	3	9	0
L+1	-1.53	A	4	3	22	69	2
LUMO	-1.55	B	5	1	27	66	2
HOMO	-5.13	A	47	6	40	6	0
H-1	-5.52	B	44	44	4	7	0
H-2	-5.99	A	69	7	11	13	1
H-3	-6.17	B	1	3	67	25	4

Table S15. Orbital contributions for **12**.

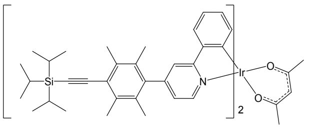

Orbital	Energy (eV)	Symmetry	Molecular Components Contributions (%)				
			Ir	acac	Ph	Py	R
L+3	-1.03	A	2	0	11	80	7
L+2	-1.06	A	2	88	2	7	1
L+1	-1.53	A	4	3	23	68	2
LUMO	-1.55	A	5	1	27	66	2
HOMO	-5.14	A	48	6	40	6	0
H-1	-5.52	A	45	43	4	8	0
H-2	-5.98	A	70	7	10	13	1
H-3	-6.20	A	1	3	72	23	1

Table S16. Orbital contributions for **13**.

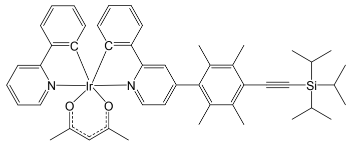

Orbital	Energy (eV)	Symmetry	Molecular Components Contributions (%)				
			Ir	acac	Ph	Py	R
L+3	-1.01	A	2	3	10	72	12
L+2	-1.06	A	2	88	2	7	0
L+1	-1.50	A	4	2	25	68	0
LUMO	-1.54	A	5	1	26	66	2
HOMO	-5.13	A	48	6	40	6	0
H-1	-5.52	A	45	43	4	8	0
H-2	-5.97	A	70	7	10	13	0
H-3	-6.19	A	1	2	72	24	1

Table S17. Orbital contributions for **14**.

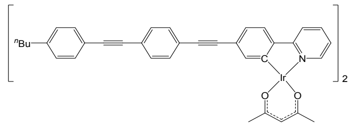

Orbital	Energy (eV)	Symmetry	Molecular Components Contributions (%)				
			Ir	acac	Ph	Py	R
L+3	-1.40	B	3	0	9	41	47
L+2	-1.42	A	2	3	8	43	44
L+1	-2.08	A	2	1	23	22	53
LUMO	-2.11	B	1	0	25	23	51
HOMO	-5.27	A	44	6	39	7	5
H-1	-5.55	B	25	23	13	7	32
H-2	-5.67	A	7	2	17	2	72
H-3	-5.68	B	17	24	11	7	41

Table S18. Orbital contributions for **15**.

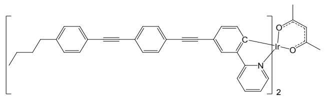

**Orbital**	**Energy (eV)**	**Symmetry**	**Molecular Components Contributions (%)**				
			**Ir**	**acac**	**Ph**	**Py**	**R**
L+3	-1.64	A	4	3	24	68	2
L+2	-1.65	B	4	0	26	61	8
L+1	-1.79	A	2	0	11	1	86
LUMO	-1.84	B	1	0	16	4	79
HOMO	-5.12	A	31	4	33	4	28
H-1	-5.57	B	1	1	24	1	73
H-2	-5.67	B	40	48	4	7	1
H-3	-5.95	A	14	2	6	2	76

Table S19. Orbital contributions for **16**.

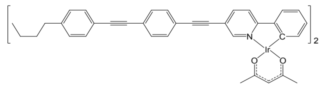

**Orbital**	**Energy (eV)**	**Symmetry**	**Molecular Components Contributions (%)**				
			**Ir**	**acac**	**Ph**	**Py**	**R**
L+3	-1.45	B	4	0	11	44	42
L+2	-1.47	A	3	3	10	49	36
L+1	-2.21	A	1	0	11	33	55
LUMO	-2.24	B	1	0	12	33	53
HOMO	-5.19	A	44	6	39	7	5
H-1	-5.60	B	39	42	6	8	5
H-2	-5.76	B	2	7	12	12	67
H-3	-5.82	A	7	1	9	10	73

Table S20. Orbital contributions for **17**.

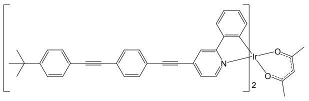

Orbital	Energy (eV)	Symmetry	Molecular Components Contributions (%)				
			Ir	acac	Ph	Py	R
L+3	-1.39	A	2	2	17	32	47
L+2	-1.42	B	2	0	24	37	37
L+1	-2.34	B	3	0	3	37	56
LUMO	-2.35	A	3	1	3	38	55
HOMO	-5.19	A	47	6	41	5	0
H-1	-5.52	B	41	36	3	8	11
H-2	-5.81	A	26	2	2	10	59
H-3	-5.96	B	1	12	1	4	82

Table S21. Orbital contributions for **18**.

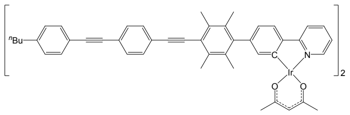

Orbital	Energy (eV)	Symmetry	Molecular Components Contributions (%)				
			Ir	acac	Ph	Py	R
L+3	-1.52	A	4	3	24	68	1
L+2	-1.54	B	4	1	29	65	1
L+1	-1.84	A	0	0	0	0	100
LUMO	-1.84	B	0	0	0	0	100
HOMO	-5.17	A	48	6	40	6	0
H-1	-5.53	B	40	39	4	7	10
H-2	-5.60	A	1	0	1	0	98
H-3	-5.61	B	4	5	1	1	90

Table S22. Orbital contributions for **19**.

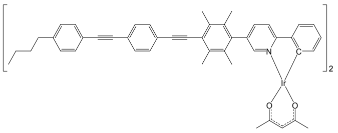

Orbital	Energy (eV)	Symmetry	Molecular Components Contributions (%)				
			Ir	acac	Ph	Py	R
L+3	-1.55	B	5	1	27	66	2
L+2	-1.55	A	4	3	22	69	2
L+1	-1.90	B	0	0	0	1	99
LUMO	-1.90	A	0	0	0	1	99
HOMO	-5.13	A	47	6	41	6	0
H-1	-5.52	B	43	44	4	8	1
H-2	-5.68	A	0	0	0	1	99
H-3	-5.68	B	0	1	0	1	98

Table S23. Orbital contributions for **20**

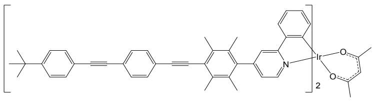

Orbital	Energy (eV)	Symmetry	Molecular Components Contributions (%)				
			Ir	acac	Ph	Py	R
L+3	-1.54	A	4	3	22	68	2
L+2	-1.55	A	5	1	27	66	2
L+1	-1.90	A	0	0	0	1	99
LUMO	-1.90	A	0	0	0	1	99
HOMO	-5.14	A	47	6	41	6	0
H-1	-5.52	A	45	43	4	8	0
H-2	-5.68	A	0	0	0	1	99
H-3	-5.68	A	0	0	0	1	99

Table S24. Orbital contributions for **21**

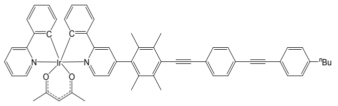

**Orbital**	**Energy (eV)**	**Symmetry**	**Molecular Components Contributions (%)**				
			**Ir**	**acac**	**Ph**	**Py**	**R**
L+3	-1.06	A	2	88	2	7	0
L+2	-1.50	A	4	2	25	68	0
L+1	-1.54	A	5	1	26	67	2
LUMO	-1.90	A	0	0	0	0	99
HOMO	-5.13	A	48	6	40	6	0
H-1	-5.52	A	45	43	4	8	0
H-2	-5.68	A	0	0	0	1	99
H-3	-5.98	A	70	7	10	13	0

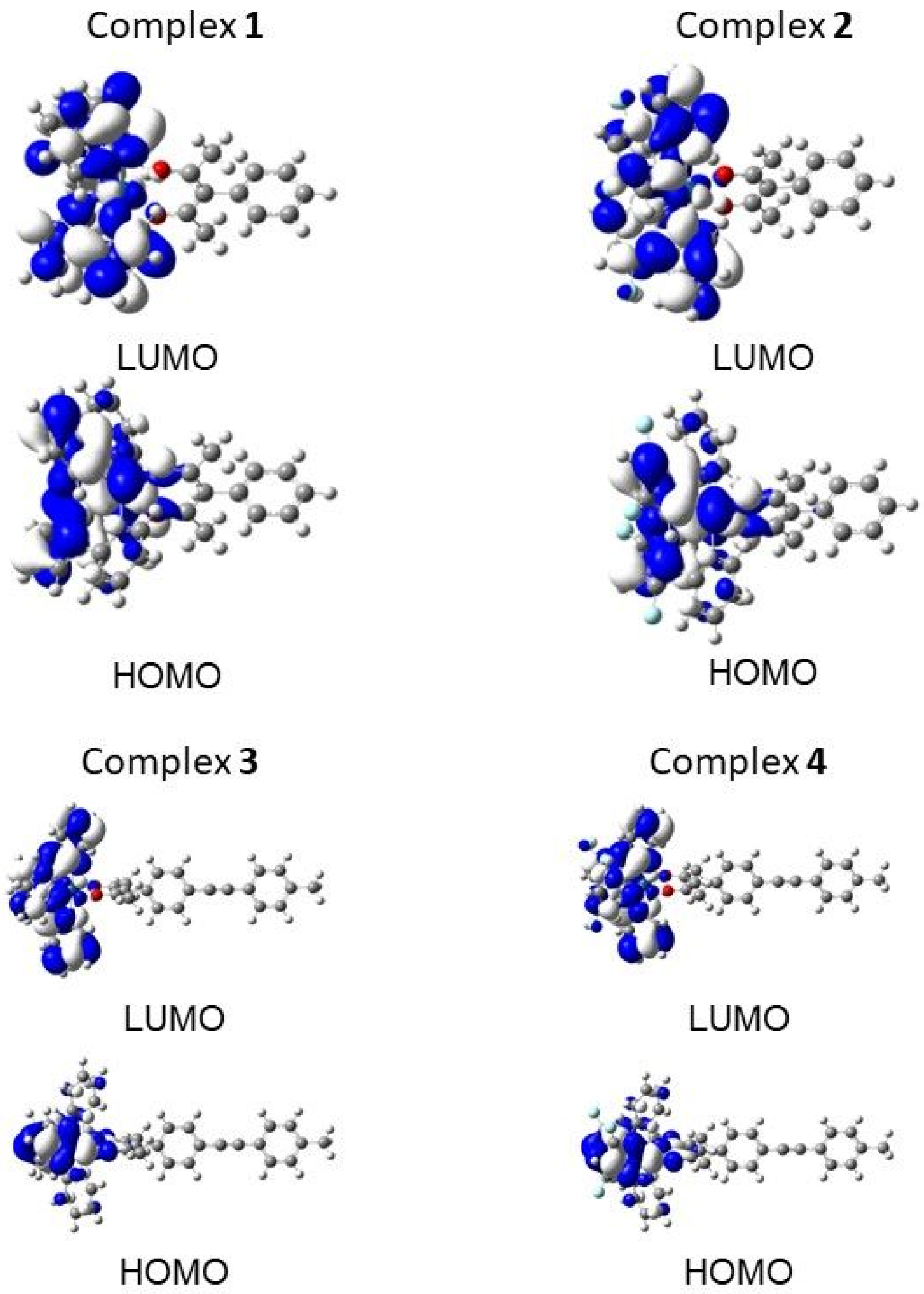

Figure S53. Frontier molecular orbitals for the fully optimised geometry complexes **1**-**4**.

Isocontours at 0.055 e bohr$^{-3/2}$.

Complex **5**

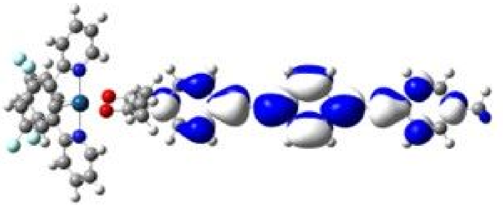

LUMO

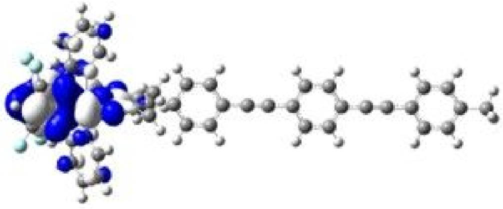

HOMO

Figure S54. Frontier molecular orbitals for the fully optimised geometry complexes **5**.

Isocontours at 0.055 e bohr$^{-3/2}$.

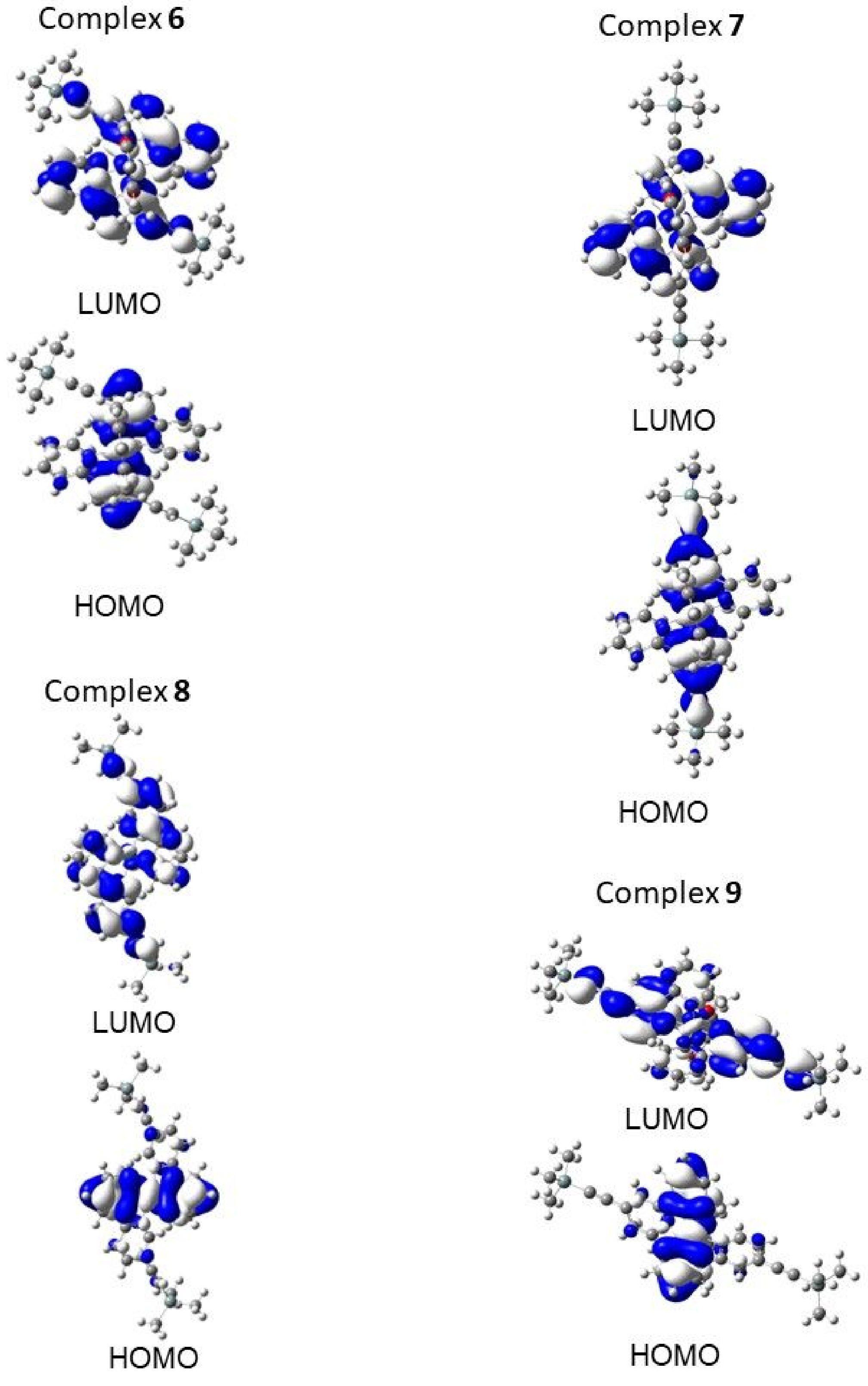

Figure S55. Frontier molecular orbitals for the fully optimised geometry complexes **6-9**.

Isocontours at 0.055 e bohr$^{-3/2}$.

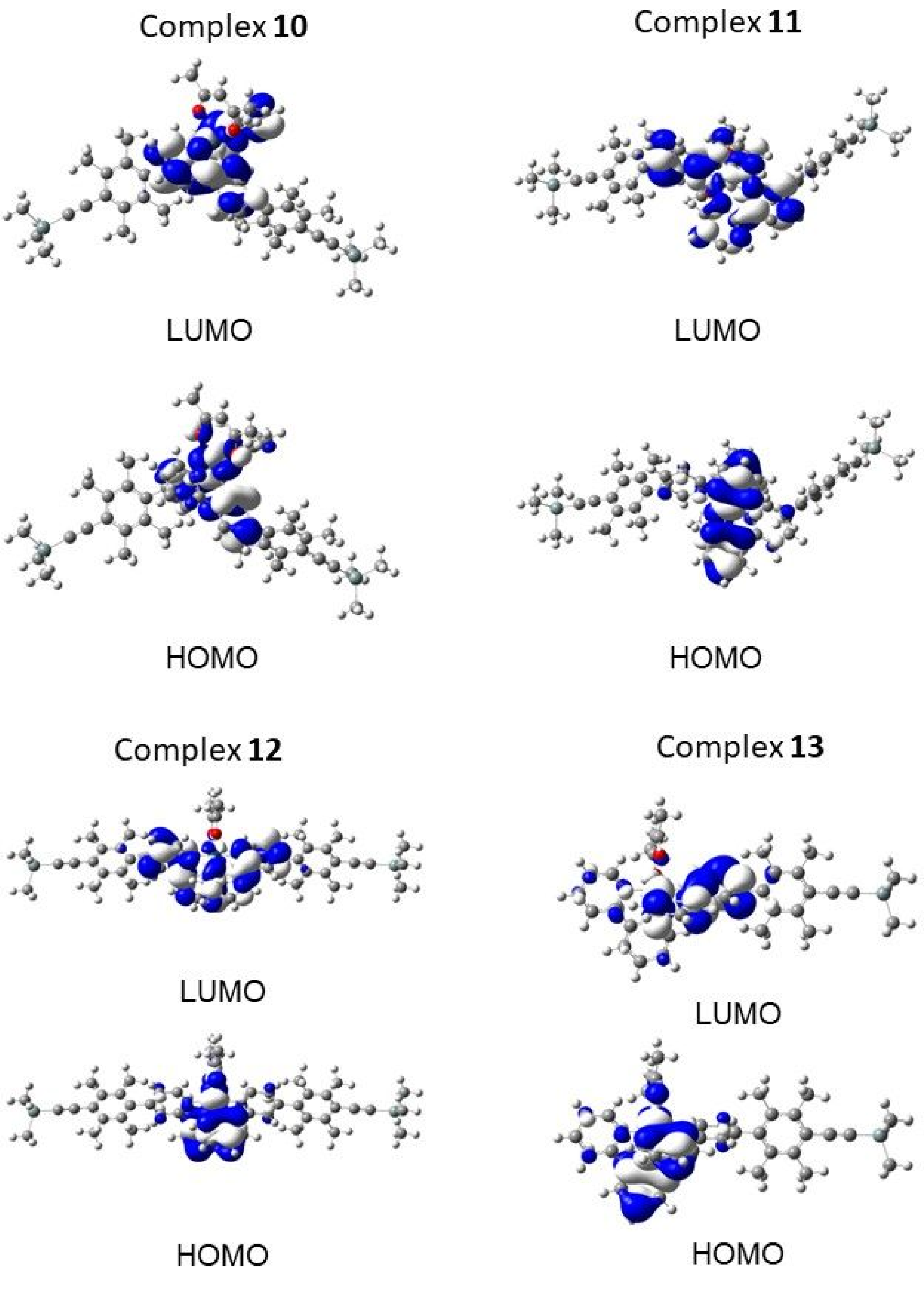

Figure S56. Frontier molecular orbitals for the fully optimised geometry complexes **10-13**. Isocontours at 0.055 e bohr$^{-3/2}$.

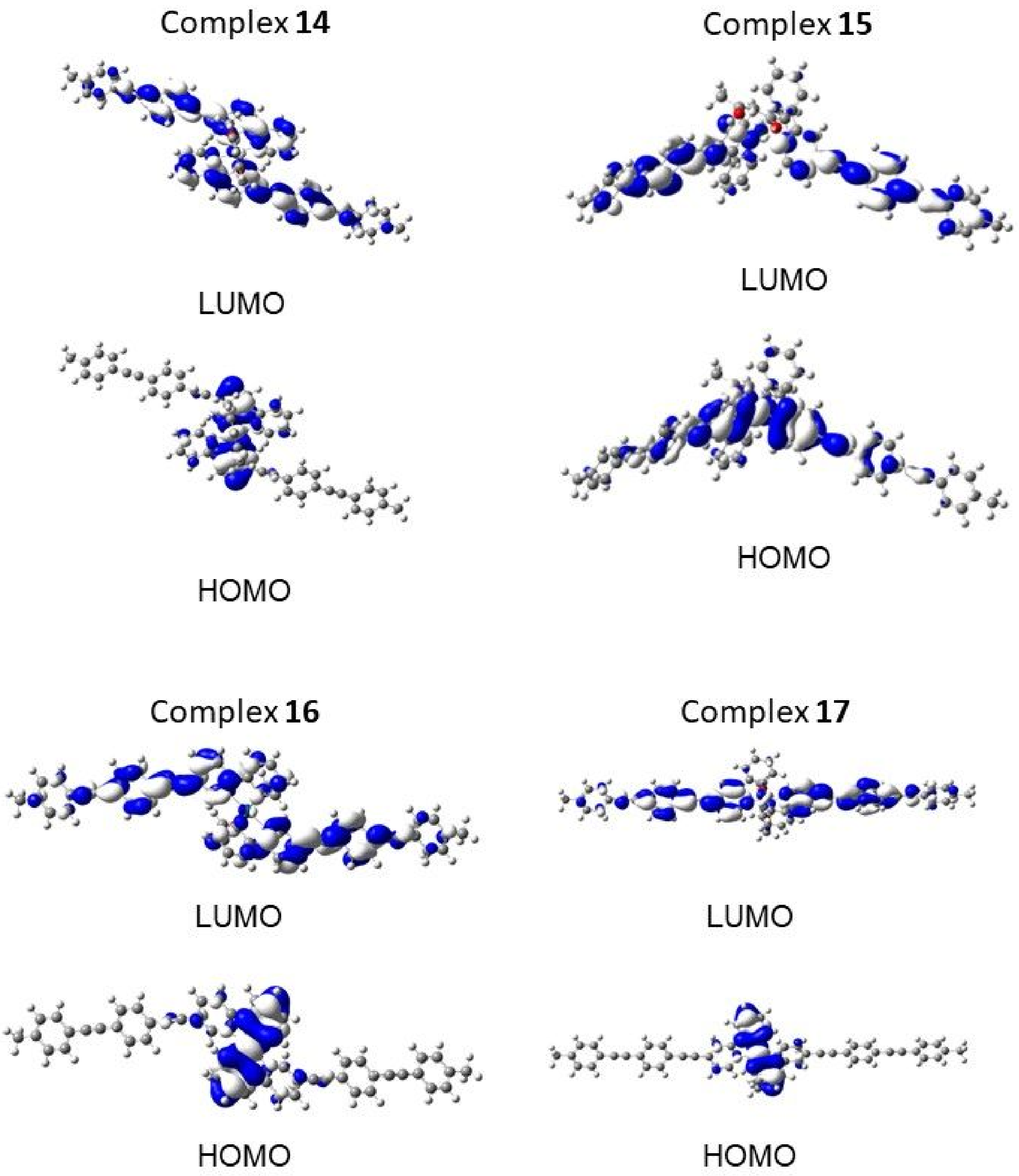

Figure S57. Frontier molecular orbitals for the fully optimised geometry complexes **14**-**17**. Isocontours at 0.055 e bohr$^{-3/2}$.

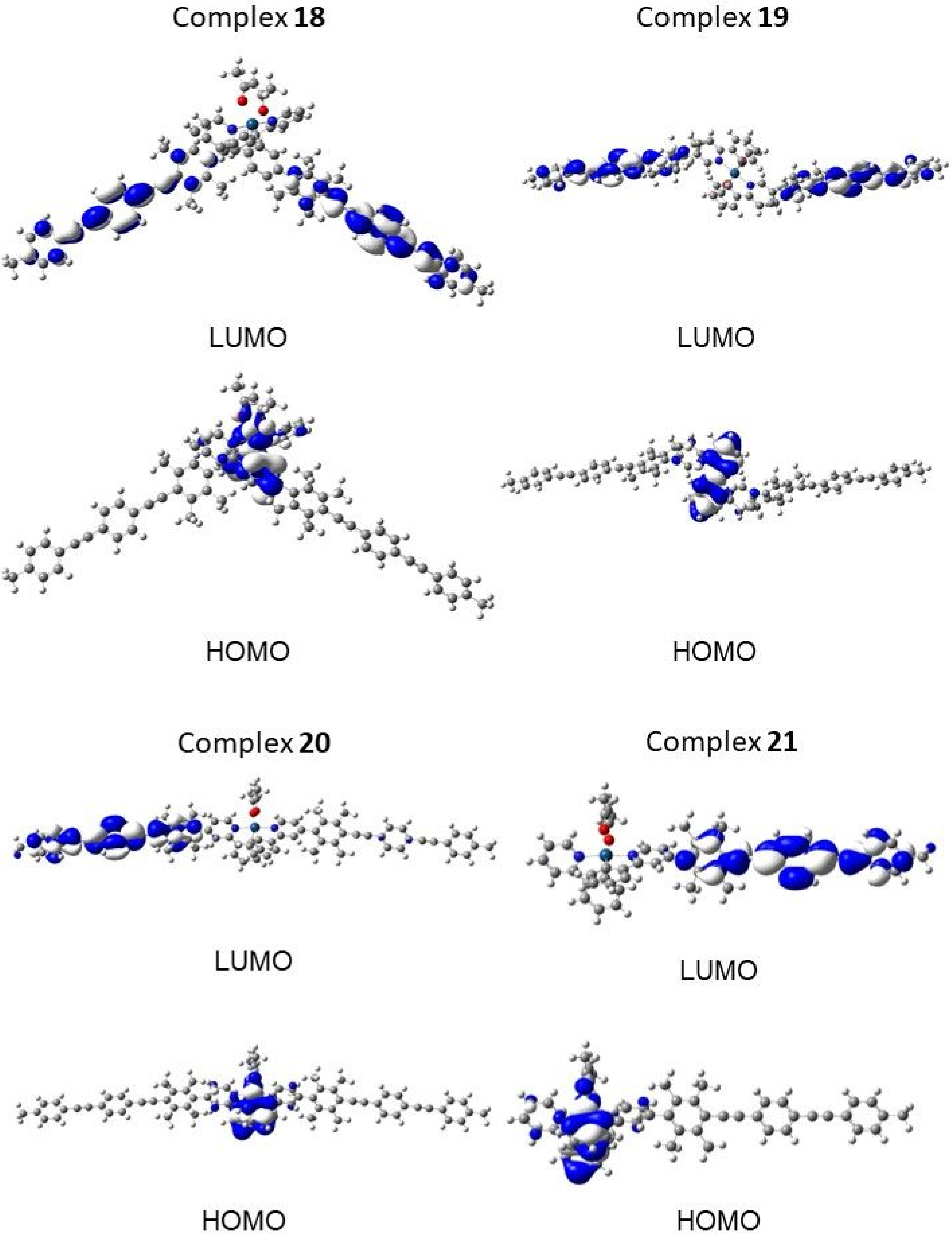

Figure S58. Frontier molecular orbitals for the fully optimised geometry complexes **18-20**. Isocontours at 0.055 e $bohr^{-3/2}$.

Electronic absorbance assignment

Table S25. Singlet and triplet transitions for **1**

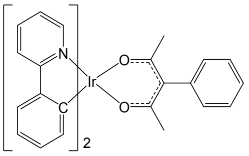

Energy (eV)	Wavelength (nm)	Osc. Strength	Transition	Major Contributions (%)		Assignment	Energy (eV)
2.592	478.29	0.0000	$S_0 \rightarrow T_1$	HOMO→LUMO	89	Ir + ph → ppy	^{3}MLCT
2.641	469.42	0.0000	$S_0 \rightarrow T_2$	HOMO→L1	87	Ir + ph → ppy	^{3}MLCT
2.807	441.60	0.0000	$S_0 \rightarrow T_3$	H-1→L+2	72	Ir + acac → ppy	^{3}MLLCT
2.813	440.67	0.0450	$S_0 \rightarrow S_1$	HOMO→LUMO	97	Ir + ph → ppy	^{1}MLCT
2.880	430.47	0.0002	$S_0 \rightarrow S_2$	HOMO→L+1	96	Ir + ph → ppy	^{1}MLCT
3.202	387.12	0.0497	$S_0 \rightarrow S_3$	H-1→L+1	95	Ir + acac → ppy	^{1}MLLCT

Table S26. Singlet and triplet transitions for **2**

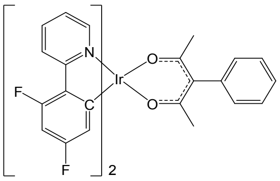

Energy (eV)	Wavelength (nm)	Osc. Strength	Transition	Major Contributions (%)		Assignment	
2.794	443.71	0.0000	$S_0 \rightarrow T_1$	HOMO→LUMO	76	Ir + ph → ppy	^{3}MLCT
2.835	437.19	0.0000	$S_0 \rightarrow T_2$	H-3→LUMO	11	ppy→ ppy	$^3\pi \rightarrow \pi^*$
				HOMO→L+1	71	Ir + ph → ppy	^{3}MLCT
2.836	437.13	0.0000	$S_0 \rightarrow T_3$	H-1→L+2	83	Ir + ph → acac	^{3}MLLCT
3.025	409.86	0.0397	$S_0 \rightarrow S_1$	HOMO→LUMO	96	Ir + ph → ppy	^{1}MLCT
3.086	401.73	0.0002	$S_0 \rightarrow S_2$	HOMO→L+1	94	Ir + ph → ppy	^{1}MLCT
3.270	379.14	0.0500	$S_0 \rightarrow S_3$	H-1→L+1	95	Ir + ph → ppy	^{1}MLCT

Table S27. Singlet and triplet transitions for **3**

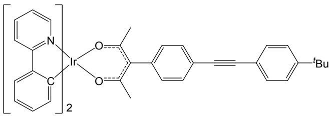

Energy (eV)	Wavelength (nm)	Osc. Strength	Transition	Major Contributions (%)		Assignment	
2.594	477.81	0.0000	$S_0 \rightarrow T_1$	HOMO→LUMO	88	Ir + ph → ppy	^{3}MLCT
2.634	470.70	0.0000	$S_0 \rightarrow T_2$	HOMO→L+1	87	Ir + ph → ppy	^{3}MLCT
2.764	448.51	0.0000	$S_0 \rightarrow T_3$	H-2→L+2	87	acac → acac	$^3\pi \rightarrow \pi^*$
2.817	440.01	0.0457	$S_0 \rightarrow S_1$	HOMO->LUMO	96	Ir + ph → ppy	^{1}MLCT
2.879	430.54	0.0001	$S_0 \rightarrow S_2$	HOMO→L+1	96	Ir + ph → ppy	^{1}MLCT
3.218	385.23	0.0508	$S_0 \rightarrow S_3$	H-1→L+1	95	Ir + acac → ppy	^{1}MLLCT

Table S28. Singlet and triplet transitions for **4**

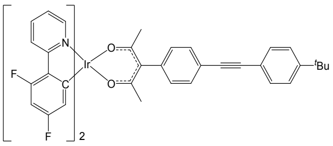

Energy (eV)	Wavelength (nm)	Osc. Strength	Transition	Major Contributions (%)		Assignment	
2.763	448.60	0.0000	$S_0 \rightarrow T_1$	H-2→L+2	88	acac → acac	$^3\pi \rightarrow \pi^*$
2.794	443.73	0.0000	$S_0 \rightarrow T_2$	HOMO→LUMO	76	Ir + ph → ppy	^{3}MLCT
2.830	438.02	0.0000	$S_0 \rightarrow T_3$	H-4→LUMO	11	ppy → ppy	^{3}MLCT / $^3\pi \rightarrow \pi^*$
				HOMO→L+1	69	Ir + ph → ppy	
3.026	409.72	0.0401	$S_0 \rightarrow S_1$	HOMO→LUMO	96	Ir + ph → ppy	^{1}MLCT
3.095	400.54	0.0000	$S_0 \rightarrow S_2$	HOMO→L+1	94	Ir + ph → ppy	^{1}MLCT
3.291	376.72	0.0481	$S_0 \rightarrow S_3$	H-1→L+1	95	Ir + acac → ppy	^{1}MLLCT

Table S29. Singlet and triplet transitions for **5**

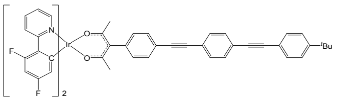

Energy (eV)	Wavelength (nm)	Osc. Strength	Transition	Major Contributions (%)		Assignment	
2.362	524.82	0.0000	$S_0 \rightarrow T_1$	H-2→LUMO	82	acac → acac	$^3\pi \rightarrow \pi^*$
2.796	443.43	0.0000	$S_0 \rightarrow T_2$	HOMO→L+1	76	Ir + ph → ppy	^{3}MLCT
2.834	437.48	0.0000	$S_0 \rightarrow T_3$	H-4→L+1	12	ppy → ppy	^{3}MLCT / $^3\pi \rightarrow \pi^*$
				HOMO→L+2	67	Ir + ph → ppy	
3.028	409.39	0.0402	$S_0 \rightarrow S_1$	HOMO→L+1	96	Ir + ph → ppy	^{1}MLCT
3.095	400.54	0.0004	$S_0 \rightarrow S_2$	HOMO→L+2	94	Ir + ph → ppy	^{1}MLCT
3.282	377.71	0.0004	$S_0 \rightarrow S_3$	HOMO→LUMO	98	Ir + ph → acac	^{1}MLLCT

Table S30. Singlet and triplet transitions for **6**

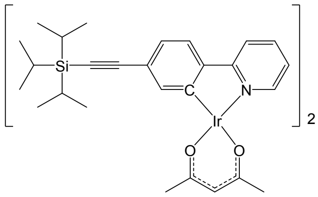

Energy (eV)	Wavelength (nm)	Osc. Strength	Transition	Major Contributions (%)		Assignment	
2.437	508.56	0.0000	$S_0 \rightarrow T_1$	H-3→L+1	10	ppy + R → ppy + R	^{3}MLCT / $^3\pi \rightarrow \pi^*$
				HOMO→LUMO	79	Ir + ph → ppy + R	
2.475	500.84	0.0000	$S_0 \rightarrow T_2$	H-3→LUMO	13	ppy + R → ppy + R	^{3}MLCT / $^3\pi \rightarrow \pi^*$
				HOMO→L+1	75	Ir + ph → ppy + R	
2.783	445.39	0.0000	$S_0 \rightarrow T_3$	H-3→LUMO	10	ppy + R → ppy + R	^{3}MLCT / $^3\pi \rightarrow \pi^*$
				H-2→L+1	13	Ir + ph → ppy + R	
				H-1→LUMO	44	Ir + acac → ppy + R	
				HOMO→L+1	17	Ir + ph → ppy + R	
2.730	454.00	0.0594	$S_0 \rightarrow S_1$	HOMO→LUMO	96	Ir + ph → ppy + R	^{1}MLCT
2.788	444.57	0.0027	$S_0 \rightarrow S_2$	HOMO→L+1	95	Ir + ph → ppy + R	^{1}MLCT
3.057	405.53	0.0400	$S_0 \rightarrow S_3$	H-1→L+1	96	Ir + acac → ppy + R	^{1}MLLCT

Table S31. Singlet and triplet transitions for **7**

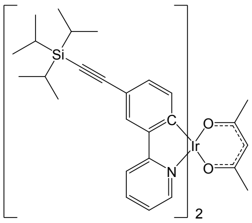

Energy (eV)	Wavelength (nm)	Osc. Strength	Transition	Major Contributions (%)		Assignment	
2.585	479.62	0.0000	$S_0 \rightarrow T_1$	HOMO→LUMO	84	Ir + ph + R → ppy	^{3}MLCT
2.624	472.44	0.0000	$S_0 \rightarrow T_2$	HOMO→L+1	83	Ir + ph + R → ppy	^{3}MLCT
2.846	435.53	0.0000	$S_0 \rightarrow T_3$	H-1→L+2	76	Ir + acac → acac	^{3}MLCT
2.825	438.78	0.0596	$S_0 \rightarrow S_1$	HOMO→LUMO	96	Ir + ph + R → ppy	^{1}MLCT
2.873	431.41	0.0004	$S_0 \rightarrow S_2$	HOMO→L+1	96	Ir + ph + R → ppy	^{1}MLCT
3.182	389.61	0.0637	$S_0 \rightarrow S_3$	H-1→L+1	95	Ir + acac → ppy	^{1}MLLCT

Table S32. Singlet and triplet transitions for **8**

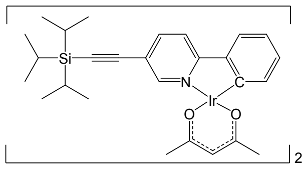

Energy (eV)	Wavelength (nm)	Osc. Strength	Transition	Major Contributions (%)		Assignment	
2.365	524.11	0.0000	$S_0 \rightarrow T_1$	HOMO→LUMO	84	Ir + ph → ppy + R	³MLCT
2.400	516.53	0.0000	$S_0 \rightarrow T_2$	H-3→LUMO	10	ppy + R → ppy + R	³MLCT / ³π→π*
				HOMO→L+1	81	Ir + ph → ppy + R	
2.710	457.50	0.0000	$S_0 \rightarrow T_3$	H-3→LUMO	10	ppy + R → ppy + R	³MLLCT / ³π→π*
				H-2→L+1	12	Ir + acac → ppy + R	
				H-1→LUMO	47	Ir + acac → ppy + R	
				HOMO→L+1	12	Ir + ph → ppy + R	
2.588	479.05	0.1202	$S_0 \rightarrow S_1$	HOMO→LUMO	97	Ir + ph → ppy + R	¹MLCT
2.659	466.21	0.0073	$S_0 \rightarrow S_2$	HOMO→L+1	96	Ir + ph → ppy + R	¹MLCT
3.014	411.27	0.0427	$S_0 \rightarrow S_3$	H-1→L+1	96	Ir + acac → ppy + R	¹MLLCT

Table S33. Singlet and triplet transitions for **9**

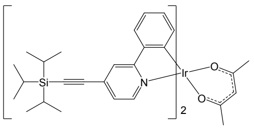

Energy (eV)	Wavelength (nm)	Osc. Strength	Transition	Major Contributions (%)		Assignment	
2.341	529.62	0.0000	$S_0 \rightarrow T_1$	HOMO→L+1	91	Ir + ph → py + R	³MLCT
2.356	526.18	0.0000	$S_0 \rightarrow T_2$	HOMO→LUMO	92	Ir + ph → py + R	³MLCT
2.541	487.93	0.0000	$S_0 \rightarrow T_3$	H-2→LUMO	15	Ir → py + R	³MLLCT
				H-1→L+1	66	Ir + acac → py + R	
2.541	497.07	0.0404	$S_0 \rightarrow S_1$	HOMO→L+1	98	Ir + ph → py + R	¹MLCT
2.516	492.72	0.0004	$S_0 \rightarrow S_2$	HOMO→LUMO	97	Ir + ph → py + R	¹MLCT
2.864	432.83	0.1603	$S_0 \rightarrow S_3$	H-1→LUMO	96	Ir + acac → py + R	¹MLLCT

Table S34. Singlet and triplet transitions for **10**

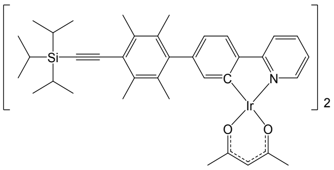

Energy (eV)	Wavelength (nm)	Osc. Strength	Transition	Major Contributions (%)		Assignment	
2.611	474.70	0.0000	$S_0 \rightarrow T_1$	HOMO→LUMO	86	Ir + ph → ppy	^{3}MLCT
2.656	466.75	0.0000	$S_0 \rightarrow T_2$	HOMO→L+1	84	Ir + ph → ppy	^{3}MLCT
2.828	438.35	0.0000	$S_0 \rightarrow T_3$	H-1→L+2	75	Ir + acac → acac	^{3}MLCT
2.844	435.85	0.0470	$S_0 \rightarrow S_1$	HOMO→LUMO	96	Ir + ph → ppy	^{1}MLCT
2.896	428.09	0.0021	$S_0 \rightarrow S_2$	HOMO→L+1	96	Ir + ph → ppy	^{1}MLCT
3.230	383.80	0.0446	$S_0 \rightarrow S_3$	H-1→L+1	95	Ir + acac → ppy	^{1}MLLCT

Table S35. Singlet and triplet transitions for **11**

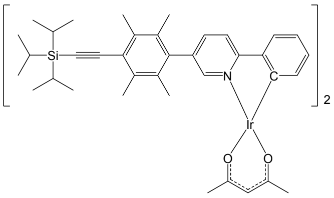

Energy (eV)	Wavelength (nm)	Osc. Strength	Transition	Major Contributions (%)		Assignment	
2.574	481.66	0.0000	$S_0 \rightarrow T_1$	HOMO→LUMO	87	Ir + ph → ppy	^{3}MLCT
2.612	474.52	0.0000	$S_0 \rightarrow T_2$	HOMO→L+1	85	Ir + ph → ppy	^{3}MLCT
2.833	437.64	0.0000	$S_0 \rightarrow T_3$	H-1→L+1	11	Ir + acac → ppy	^{3}MLCT
				H-1→L+2	69	Ir + acac → acac	
2.792	443.92	0.0693	$S_0 \rightarrow S_1$	HOMO→LUMO	97	Ir + ph → ppy	^{1}MLCT
2.850	434.98	0.0023	$S_0 \rightarrow S_2$	HOMO→L+1	97	Ir + ph → ppy	^{1}MLCT
3.208	386.47	0.0590	$S_0 \rightarrow S_3$	H-1→L+1	95	Ir + acac → ppy	^{1}MLLCT

Table S36. Singlet and triplet transitions for **12**

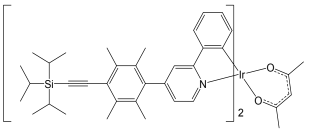

Energy (eV)	Wavelength (nm)	Osc. Strength	Transition	Major Contributions (%)		Assignment	
2.587	479.14	0.0000	$S_0 \to T_1$	HOMO→LUMO	88	Ir + ph → ppy	^{3}MLCT
2.607	475.45	0.0000	$S_0 \to T_2$	HOMO→L+1	87	Ir + ph → ppy	^{3}MLCT
2.825	438.80	0.0000	$S_0 \to T_3$	H-1→L+1	11	Ir + acac → ppy	^{3}MLCT
				H-1→L+2	71	Ir + acac → acac	
2.804	442.16	0.0753	$S_0 \to S_1$	HOMO→LUMO	97	Ir + ph → ppy	^{1}MLCT
2.858	433.67	0.0003	$S_0 \to S_2$	HOMO→L+1	96	Ir + ph → ppy	^{1}MLCT
3.206	386.62	0.1041	$S_0 \to S_3$	H-1→L+1	95	Ir + acac → ppy	^{1}MLLCT

Table S37. Singlet and triplet transitions for **13**

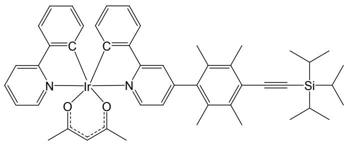

Energy (eV)	Wavelength (nm)	Osc. Strength	Transition	Major Contributions (%)		Assignment	
2.5923	478.27	0.0000	$S_0 \to T_1$	HOMO→LUMO	87	Ir + ph → ppy	^{3}MLCT
2.6199	473.24	0.0000	$S_0 \to T_2$	HOMO→L+1	87	Ir + ph → ppy′	^{3}MLCT
2.8261	438.71	0.0000	$S_0 \to T_3$	H-1→L+2	72	Ir + acac → acac	^{3}MLCT
2.8098	441.25	0.0552	$S_0 \to S_1$	HOMO→LUMO	90	Ir + ph → ppy	^{1}MLCT
2.8746	431.30	0.0034	$S_0 \to S_2$	HOMO→L+1	90	Ir + ph → ppy′	^{1}MLCT
3.2180	385.28	0.0689	$S_0 \to S_3$	H-1→LUMO	57	Ir + acac → ppy	^{1}MLLCT
				H-1→L+1	37	Ir + acac → ppy′	

Table S38. Singlet and triplet transitions for **14**

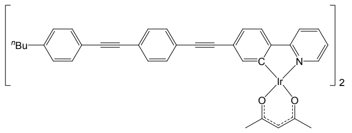

Energy (eV)	Wavelength (nm)	Osc. Strength	Transition	Major Contributions (%)		Assignment	
2.2100	561.01	0.0000	$S_0 \rightarrow T_1$	H-3→L+1	12	Ir + ph + R → ppy + R	^{3}MLCT
				H-2→LUMO	17	Ir + ph + R → ppy + R	
				H-1→L+1	18	Ir + ph + R → ppy + R	
				HOMO→LUMO	38	Ir + ph → ppy + R	
2.2210	558.23	0.0000	$S_0 \rightarrow T_2$	H-3→LUMO	14	Ir + ph + R → ppy+ R	^{3}MLCT
				H-2→L+1	19	Ir + ph + R → ppy + R	
				H-1→LUMO	22	Ir + ph + R → ppy + R	
				HOMO→L+1	31	Ir + ph → ppy + R	
2.4524	505.56	0.0000	$S_0 \rightarrow T_3$	H-2→LUMO	23	Ir + ph + R → ppy + R	^{3}MLCT
				HOMO→LUMO	44	Ir + ph → ppy + R	
2.6016	476.56	0.1228	$S_0 \rightarrow S_1$	HOMO→LUMO	89	Ir + ph → ppy + R	^{1}MLCT
2.6453	468.69	0.0263	$S_0 \rightarrow S_2$	HOMO→L+1	89	Ir + ph → ppy + R	^{1}MLCT
2.8643	432.86	0.2954	$S_0 \rightarrow S_3$	H-3→L+1	11	Ir + ph + R → ppy + R	^{1}MLCT
				H-1→L+1	78	Ir + ph + R → ppy + R	

Table S39. Singlet and triplet transitions for **15**

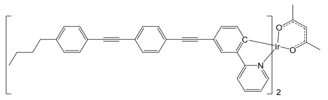

Energy (eV)	Wavelength (nm)	Osc. Strength	Transition	Major Contributions (%)		Assignment	
2.296	539.85	0.0000	$S_0 \rightarrow T_1$	H-1→L+1	30	ph + R → R	^{3}MLCT / $^3\pi \rightarrow \pi^*$
				HOMO→LUMO	49	Ir + ph + R → R	
2.312	536.12	0.0000	$S_0 \rightarrow T_2$	H-3→L+1	10	R → R	^{3}MLCT / $^3\pi \rightarrow \pi^*$
				H-1→LUMO	35	ph + R → R	
				HOMO→L+1	42	Ir + ph + R → R	
2.584	479.81	0.0000	$S_0 \rightarrow T_3$	HOMO→L+2	73	Ir + ph + R → ppy	^{3}MLCT
2.753	450.34	0.3685	$S_0 \rightarrow S_1$	HOMO→LUMO	42	Ir + ph + R → R	^{1}MLCT
				HOMO→L+2	52	Ir + ph + R → ppy	
2.841	436.39	0.0000	$S_0 \rightarrow S_2$	HOMO→L+3	90	Ir + ph + R → ppy	^{1}MLCT
2.957	419.24	1.2757	$S_0 \rightarrow S_3$	HOMO→LUMO	53	Ir + ph + R → R	^{1}MLCT
				HOMO→L+2	42	Ir + ph + R → ppy	

Table S40. Singlet and triplet transitions for **16**

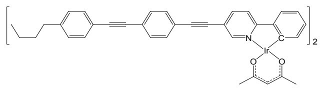

Energy (eV)	Wavelength (nm)	Osc. Strength	Transition	Major Contributions (%)		Assignment	
2.159	574.08	0.0000	$S_0 \rightarrow T_1$	H-3→LUMO	11	ppy + R → ppy + R	^{3}MLCT / $^3\pi\rightarrow\pi^*$
				H-2→L+1	17	ppy + R → ppy + R	
				HOMO→LUMO	56	Ir + ph → ppy + R	
2.176	569.57	0.0000	$S_0 \rightarrow T_2$	H-3→L+1	12	ppy + R → ppy + R	^{3}MLCT / $^3\pi\rightarrow\pi^*$
				H-2→LUMO	20	ppy + R → ppy + R	
				HOMO→L+1	49	Ir + ph → ppy + R	
2.416	513.05	0.0000	$S_0 \rightarrow T_3$	H-3→LUMO	26	ppy + R → ppy + R	^{3}MLCT / $^3\pi\rightarrow\pi^*$
				H-2→L+1	17	ppy + R → ppy + R	
				HOMO→LUMO	29	Ir + ph → ppy + R	
2.400	516.51	0.3749	$S_0 \rightarrow S_1$	HOMO→LUMO	93	Ir + ph → ppy + R	^{1}MLCT
2.472	501.39	0.0550	$S_0 \rightarrow S_2$	HOMO→L+1	93	Ir + ph → ppy + R	^{1}MLCT
2.837	437.02	0.0108	$S_0 \rightarrow S_3$	H-1→LUMO	92	Ir + acac → ppy + R	^{1}MLLCT

Table S41. Singlet and triplet transitions for **17**

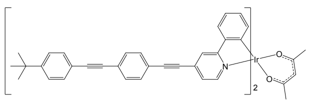

Energy (eV)	Wavelength (nm)	Osc. Strength	Transition	Major Contributions (%)		Assignment	
2.1792	568.94	0.0000	$S_0 \rightarrow T_1$	HOMO→L+1	86	Ir + ph → py + R	^{3}MLCT
2.1887	566.47	0.0000	$S_0 \rightarrow T_2$	HOMO→LUMO	89	Ir + ph → py + R	^{3}MLCT
2.2114	560.65	0.0000	$S_0 \rightarrow T_3$	H-3→L+1	12	R → py + R	^{3}MLCT / ^{3}MLLCT
				H-2→LUMO	32	Ir + py + R → py + R	
				H-1→L+1	38	Ir + acac → py + R	
2.2868	542.17	0.0755	$S_0 \rightarrow S_1$	HOMO→L+1	94	Ir + ph → py + R	^{1}MLCT
2.3028	538.40	0.0003	$S_0 \rightarrow S_2$	HOMO→LUMO	95	Ir + ph → py + R	^{1}MLCT
2.6238	472.53	0.7846	$S_0 \rightarrow S_3$	H-1→LUMO	94	Ir + acac → py + R	^{1}MLLCT

Table S42. Singlet and triplet transitions for **18**

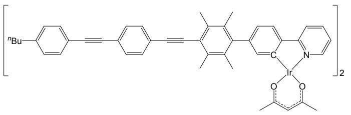

Energy (eV)	Wavelength (nm)	Osc. Strength	Transition	Major Contributions (%)		Assignment	
2.363	524.55	0.0000	$S_0 \rightarrow T_1$	H-3→LUMO	40	R → R	$^3\pi \rightarrow \pi^*$
				H-2→L+1	44	R → R	
2.363	524.55	0.0000	$S_0 \rightarrow T_2$	H-3→L+1	40	R → R	$^3\pi \rightarrow \pi^*$
				H-2→LUMO	44	R → R	
2.610	474.92	0.0000	$S_0 \rightarrow T_3$	HOMO→L+2	86	Ir + ph → ppy	^{3}MLCT
2.842	436.16	0.0555	$S_0 \rightarrow S_1$	HOMO→L+2	96	Ir + ph → ppy	^{1}MLCT
2.897	427.84	0.0048	$S_0 \rightarrow S_2$	HOMO→L+3	96	Ir + ph → ppy	^{1}MLCT
3.073	403.37	0.0005	$S_0 \rightarrow S_3$	HOMO→LUMO	99	Ir + ph → R	^{1}MLCT

Table S43. Singlet and triplet transitions for **19**

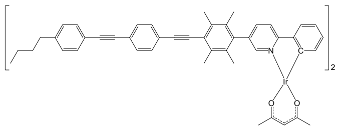

Energy (eV)	Wavelength (nm)	Osc. Strength	Transition	Major Contributions (%)		Assignment	
2.368	523.49	0.0000	$S_0 \rightarrow T_1$	H-3→LUMO	44	R → R	$^3\pi \rightarrow \pi^*$
				H-2→L+1	44	R → R	
2.368	523.49	0.0000	$S_0 \rightarrow T_2$	H-3→L+1	44	R → R	$^3\pi \rightarrow \pi^*$
				H-2→LUMO	44	R → R	
2.579	480.70	0.0000	$S_0 \rightarrow T_3$	HOMO→L+3	87	Ir + ph → ppy	^{3}MLCT
2.799	442.87	0.1123	$S_0 \rightarrow S_1$	HOMO→L+3	97	Ir + ph → ppy	^{1}MLCT
2.837	436.96	0.0044	$S_0 \rightarrow S_2$	HOMO→L+2	96	Ir + ph → R	^{1}MLCT
2.996	413.80	0.0000	$S_0 \rightarrow S_3$	HOMO→LUMO	99	Ir + ph → R	^{1}MLCT

Table S44. Singlet and triplet transitions for **20**

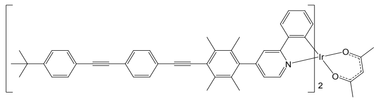

Energy (eV)	Wavelength (nm)	Osc. Strength	Transition	Major Contributions (%)		Assignment	
2.369	523.16	0.0000	$S_0 \rightarrow T_1$	H-2→L+1	85	R→ R	$^3\pi \rightarrow \pi^*$
2.370	523.14	0.0000	$S_0 \rightarrow T_2$	H-3→LUMO	85	R→ R	$^3\pi \rightarrow \pi^*$
2.585	479.49	0.0000	$S_0 \rightarrow T_3$	HOMO→L+2	88	Ir + ph → ppy	^{3}MLCT
2.800	442.67	0.1222	$S_0 \rightarrow S_1$	HOMO→L+2	96	Ir + ph → ppy	^{1}MLCT
2.853	434.43	0.0005	$S_0 \rightarrow S_2$	HOMO→L+3	95	Ir + ph → ppy	^{1}MLCT
2.978	416.33	0.0004	$S_0 \rightarrow S_3$	HOMO→LUMO	97	Ir + ph → R	^{1}MLCT

Table S45. Singlet and triplet transitions for **21**

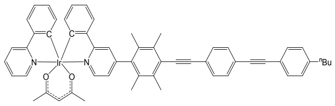

Energy (eV)	Wavelength (nm)	Osc. Strength	Transition	Major Contributions (%)		Assignment	
2.3710	522.91	0.0000	$S_0 \rightarrow T_1$	H-2→LUMO	89	R → R	$^3\pi \rightarrow \pi^*$
2.5909	478.53	0.0000	$S_0 \rightarrow T_2$	HOMO→L+1	88	Ir + ph → ppy	^{3}MLCT
2.6185	473.49	0.0000	$S_0 \rightarrow T_3$	HOMO→L+2	87	Ir + ph → ppy′	^{3}MLCT
2.8096	441.28	0.0740	$S_0 \rightarrow S_1$	HOMO→L+1	88	Ir + ph → ppy	^{1}MLCT
2.8717	431.74	0.0039	$S_0 \rightarrow S_2$	HOMO→L+2	88	Ir + ph → ppy′	^{1}MLCT
3.0110	411.77	0.0001	$S_0 \rightarrow S_3$	HOMO→LUMO	99	Ir + ph → R	^{1}MLCT

Table S46. Singlet and triplet transitions for 1,4-bis(phenylethynyl)benzene

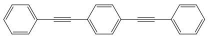

Energy (eV)	Wavelength (nm)	Osc. Strength	Transition	Major Contributions (%)		Assignment
2.390	518.11	0.0000	$S_0 \rightarrow T_1$	HOMO→LUMO	89	$^3\pi \rightarrow \pi^*$
3.155	392.41	0.0000	$S_0 \rightarrow T_2$	H-1→LUMO	49	$^3\pi \rightarrow \pi^*$
				HOMO→L+1	35	
3.794	326.66	0.0000	$S_0 \rightarrow T_3$	H-7→LUMO	24	$^3\pi \rightarrow \pi^*$
				H-3→L+4	10	
				H-2→L+3	10	
				H-1→L+1	22	
				HOMO→L+5	23	
3.483	355.83	2.1346	$S_0 \rightarrow S_1$	HOMO→LUMO	99	$^1\pi \rightarrow \pi^*$
4.335	285.27	0.0003	$S_0 \rightarrow S_2$	H-4→LUMO	47	$^1\pi \rightarrow \pi^*$
				HOMO→L+2	51	
4.341	270.51	0.0062	$S_0 \rightarrow S_3$	H-2→LUMO	70	$^1\pi \rightarrow \pi^*$
				HOMO→L+3	21	

Table S47. Singlet and triplet transitions for **L**^{1}H

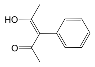

Energy (eV)	Wavelength (nm)	Osc. Strength	Transition	Major Contributions (%)		Assignment	
3.050	406.50	0.0000	$S_0 \rightarrow T_1$	HOMO→LUMO	99	acac→acac	$^3\pi\rightarrow\pi^*$
3.690	335.96	0.0000	$S_0 \rightarrow T_2$	H-3→LUMO	22	acac+R→ acac	$^3\pi\rightarrow\pi^*$
				H-1→LUMO	76	acac+R→ acac	
3.874	319.97	0.0000	$S_0 \rightarrow T_3$	H-3→L+1	21	acac+R→R	$^3\pi\rightarrow\pi^*$
				H-2→L+2	43	R→R	
				H-1→L+1	36	acac+R→R	
4.064	305.04	0.0006	$S_0 \rightarrow S_1$	H-3→LUMO	16	acac+R→acac	$^1\pi\rightarrow\pi^*$
				H-1→LUMO	83	acac+R→acac	
4.859	255.12	0.2487	$S_0 \rightarrow S_2$	HOMO→LUMO	98	acac→acac	$^1\pi\rightarrow\pi^*$
5.125	241.78	0.0051	$S_0 \rightarrow S_3$	H-2→LUMO	99	R→acac	^{1}CT

Table S48. Singlet and triplet transitions for **L**^{2}H

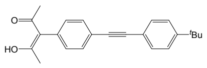

Energy (eV)	Wavelength (nm)	Osc. Strength	Transition	Major Contributions (%)		Assignment	
2.765	448.32	0.0000	$S_0 \rightarrow T_1$	HOMO→LUMO	89	R→R	$^3\pi\rightarrow\pi^*$
3.067	404.22	0.0000	$S_0 \rightarrow T_2$	H-1→L+1	99	acac→acac	$^3\pi\rightarrow\pi^*$
3.654	339.27	0.0000	$S_0 \rightarrow T_3$	H-2→L+1	64	acac+R→acac	$^3\pi\rightarrow\pi^*$
				HOMO→L+1	30	R→acac	
3.946	314.19	0.0005	$S_0 \rightarrow S_1$	H-2→L+1	36	acac+R→acac	^{1}CT
				HOMO→L+1	62	R→acac	
4.076	304.14	1.4520	$S_0 \rightarrow S_2$	HOMO→LUMO	98	R→R	$^1\pi\rightarrow\pi^*$
4.313	283.56	0.0001	$S_0 \rightarrow S_3$	H-2→L+1	54	acac+R→acac	^{1}CT
				HOMO→L+1	36	R→acac	

Table S49. Singlet and triplet transitions for **L**^{3}H

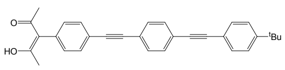

Energy (eV)	Wavelength (nm)	Osc. Strength	Transition	Major Contributions (%)		Assignment	
2.362	524.75	0.0000	$S_0 \rightarrow T_1$	HOMO→LUMO	89	R→R	$^3\pi\rightarrow\pi^*$
3.069	403.92	0.0000	$S_0 \rightarrow T_2$	H-1→L+1	97	acac→acac	$^3\pi\rightarrow\pi^*$
3.098	400.14	0.0000	$S_0 \rightarrow T_3$	H-2→LUMO	45	acac+R→R	$^3\pi\rightarrow\pi^*$
				HOMO→L+2	35	R→ R	
3.401	364.55	2.3895	$S_0 \rightarrow S_1$	HOMO→LUMO	99	R→R	$^1\pi\rightarrow\pi^*$
3.926	315.80	0.0004	$S_0 \rightarrow S_2$	H-3→L+1	19	acac+R→acac	^{1}CT
				H-2→L+1	19	acac+R→acac	
				HOMO→L+1	61	R→acac	
4.006	309.46	0.0001	$S_0 \rightarrow S_3$	H-1→LUMO	95	acac→R	^{1}CT

Table S50. Singlet and triplet transitions for **L**^{4}H

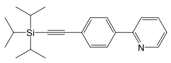

Energy (eV)	Wavelength (nm)	Osc. Strength	Transition	Major Contributions (%)		Assignment	
2.796	443.30	0.0000	$S_0 \rightarrow T_1$	HOMO→LUMO	88	ppy+R→ppy+R	$^{3}\pi\rightarrow\pi^*$
3.730	332.31	0.0000	$S_0 \rightarrow T_2$	H-1→LUMO	71	py→ppy+R	$^{3}\pi\rightarrow\pi^*$
				H-1→L+1	22	py→ppy	
3.912	316.93	0.0000	$S_0 \rightarrow T_3$	H-5→L+1	13	py→ppy	$^{3}\pi\rightarrow\pi^*$
				H-4→LUMO	37	ppy+R→ppy+R	
				HOMO→L+3	24	ppy+R→ppy+R	
4.155	298.39	1.2584	$S_0 \rightarrow S_1$	HOMO→LUMO	98	ppy+R→ppy+R	$^{1}\pi\rightarrow\pi^*$
4.226	293.33	0.0033	$S_0 \rightarrow S_2$	H-1→LUMO	96	py→ppy+R	$^{1}\pi\rightarrow\pi^*$
4.570	271.24	0.0121	$S_0 \rightarrow S_3$	H-2→LUMO	50	ph→ppy+R	$^{1}\pi\rightarrow\pi^*$
				HOMO→L+1	35	ppy+R→ppy	
				HOMO→L+2	12	ppy+R→ppy	

Table S51. Singlet and triplet transitions for **L**^{5}H

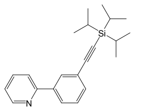

Energy (eV)	Wavelength (nm)	Osc. Strength	Transition	Major Contributions (%)		Assignment	
3.034	408.60	0.0000	$S_0 \rightarrow T_1$	H-1→L+1	16	ppy+R→ppy+R	$^{3}\pi\rightarrow\pi^*$
				HOMO→LUMO	61	ppy+R→ppy+R	
3.648	339.85	0.0000	$S_0 \rightarrow T_2$	H-1→LUMO	35	ppy+R→ppy+R	$^{3}\pi\rightarrow\pi^*$
				HOMO→L+1	44	ppy+R→ppy+R	
3.814	325.01	0.0000	$S_0 \rightarrow T_3$	H-2→LUMO	71	py→ppy+R	$^{3}\pi\rightarrow\pi^*$
				H-2→L+2	26	py→ ppy+R	
4.389	282.48	0.0025	$S_0 \rightarrow S_1$	H-2→LUMO	93	py→ppy+R	$^{1}\pi\rightarrow\pi^*$
4.431	279.79	0.0323	$S_0 \rightarrow S_2$	H-1→LUMO	29	ppy+R→ppy+R	$^{1}\pi\rightarrow\pi^*$
				HOMO→LUMO	40	ppy+R→ppy+R	
				HOMO→L+1	24	ppy+R→ ppy+R	
4.565	271.59	0.2496	$S_0 \rightarrow S_3$	H-1→LUMO	30	ppy+R→ppy+R	$^{1}\pi\rightarrow\pi^*$
				HOMO→LUMO	51	ppy+R→ppy+R	
				HOMO→L+1	11	ppy+R→ ppy+R	

Table S52. Singlet and triplet transitions for **L**^{6}H

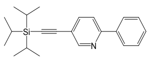

Energy (eV)	Wavelength (nm)	Osc. Strength	Transition	Major Contributions (%)		Assignment	
2.795	443.46	0.0000	$S_0 \rightarrow T_1$	HOMO→LUMO	89	ppy+R→ppy+R	$^3\pi \rightarrow \pi^*$
3.740	331.47	0.0000	$S_0 \rightarrow T_2$	H-1→LUMO	78	ppy+R→ppy+R	$^3\pi \rightarrow \pi^*$
				H-1→L+1	15	ppy+R→py	
3.818	324.71	0.0000	$S_0 \rightarrow T_3$	H-3→LUMO	45	ppy+R→ppy+R	$^3\pi \rightarrow \pi^*$
				H-2→L+2	17	ph→ph	
				HOMO→L+3	23	ppy+R→ppy+R	
4.094	302.80	0.0209	$S_0 \rightarrow S_1$	H-1→LUMO	97	ppy+R→ppy+R	$^1\pi \rightarrow \pi^*$
4.128	300.28	1.2369	$S_0 \rightarrow S_2$	HOMO→LUMO	96	ppy+R→ppy+R	$^1\pi \rightarrow \pi^*$
4.592	269.99	0.0161	$S_0 \rightarrow S_3$	H-2→LUMO	62	ph→ppy+R	$^3\pi \rightarrow \pi^*$
				HOMO→L+1	28	ppy+R→py	

Table S53. Singlet and triplet transitions for **L**^{7}H

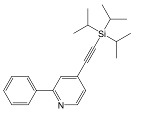

Energy (eV)	Wavelength (nm)	Osc. Strength	Transition	Major Contributions (%)		Assignment	
3.054	405.89	0.0000	$S_0 \rightarrow T_1$	H-1→LUMO	17	ppy+R→ppy+R	$^3\pi \rightarrow \pi^*$
				HOMO→LUMO	45	ppy→ppy+R	
				HOMO→L+1	19	ppy→ppy+R	
3.569	347.37	0.0000	$S_0 \rightarrow T_2$	H-1→LUMO	41	ppy+R→ppy+R	$^3\pi \rightarrow \pi^*$
				HOMO→LUMO	12	ppy→ppy+R	
3.574	346.89	0.0000	$S_0 \rightarrow T_3$	H-2→LUMO	83	py+R→ppy+R	$^3\pi \rightarrow \pi^*$
4.226	293.34	0.0034	$S_0 \rightarrow S_1$	H-2→LUMO	96	py+R→ppy+R	$^1\pi \rightarrow \pi^*$
4.303	288.08	0.2022	$S_0 \rightarrow S_2$	HOMO→LUMO	80	ppy→ppy+R	$^1\pi \rightarrow \pi^*$
4.644	266.93	0.0182	$S_0 \rightarrow S_3$	H-3→LUMO	43	ppy+R→ppy+R	$^1\pi \rightarrow \pi^*$
				H-1→LUMO	33	ppy+R→ppy+R	
				HOMO→L+1	10	ppy→ppy+R	

Table S54. Singlet and triplet transitions for **L**^{8}H

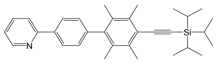

Energy (eV)	Wavelength (nm)	Osc. Strength	Transition	Major Contributions (%)		Assignment	
3.163	391.93	0.0000	$S_0 \rightarrow T_1$	H-2→LUMO	81	ppy→ppy	$^3\pi \rightarrow \pi^*$
3.232	383.54	0.0000	$S_0 \rightarrow T_2$	HOMO→L+1	79	R→R	$^3\pi \rightarrow \pi^*$
3.823	324.28	0.0000	$S_0 \rightarrow T_3$	H-3→LUMO	71	ppy→ppy	$^3\pi \rightarrow \pi^*$
				H-3→L+2	23	ppy→ppy	
4.324	286.73	0.0005	$S_0 \rightarrow S_1$	H-3→LUMO	16	ppy→ppy	$^1\pi \rightarrow \pi^*$
				HOMO→LUMO	83	R→ppy	
4.338	285.78	0.0023	$S_0 \rightarrow S_2$	H-3→LUMO	82	ppy→ppy	$^1\pi \rightarrow \pi^*$
				HOMO→LUMO	15	R→ppy	
4.350	285.01	0.0001	$S_0 \rightarrow S_3$	H-1→LUMO	99	R→ppy	$^1\pi \rightarrow \pi^*$

Table S55. Singlet and triplet transitions for **L**[9]H

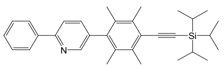

Energy (eV)	Wavelength (nm)	Osc. Strength	Transition	Major Contributions (%)		Assignment	
3.164	391.74	0.0000	$S_0 \rightarrow T_1$	H-2→LUMO	66	ppy+R→ppy	$^3\pi \rightarrow \pi^*$
				H-1→LUMO	14	ppy+R→ppy	
3.224	384.53	0.0000	$S_0 \rightarrow T_2$	HOMO→L+1	76	R→R	$^3\pi \rightarrow \pi^*$
3.777	328.24	0.0000	$S_0 \rightarrow T_3$	H-3→LUMO	53	ppy→ppy	$^3\pi \rightarrow \pi^*$
				H-3→L+2	18	ppy→ppy	
				HOMO→LUMO	20	R→ppy	
4.212	294.33	0.0039	$S_0 \rightarrow S_1$	H-3→LUMO	23	ppy→ppy	^{1}CT
				HOMO→LUMO	76	R→ppy	
4.281	289.56	0.0226	$S_0 \rightarrow S_2$	H-2→LUMO	10	ppy+R→ppy	$^1\pi \rightarrow \pi^*$
				H-1→LUMO	89	ppy+R→ppy	
4.385	282.69	0.2788	$S_0 \rightarrow S_3$	H-3→LUMO	59	ppy→ppy	$^1\pi \rightarrow \pi^*$
				H-2→LUMO	15	ppy+R→ppy	
				HOMO→LUMO	19	R→ppy	

Table S56. Singlet and triplet transitions for **L**[10]H

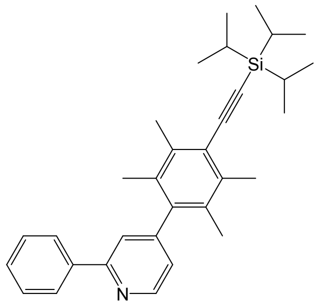

Energy (eV)	Wavelength (nm)	Osc. Strength	Transition	Major Contributions (%)		Assignment	
3.194	388.08	0.0000	$S_0 \rightarrow T_1$	H-2→LUMO	60	ppy+R→ppy	$^3\pi \rightarrow \pi^*$
				H-1→LUMO	16	ppy+R→ppy	
3.232	383.51	0.0000	$S_0 \rightarrow T_2$	H-1→L+3	10	ppy+R→ppy+R	$^3\pi \rightarrow \pi^*$
				HOMO→L+1	75	R→R	
3.838	322.96	0.0000	$S_0 \rightarrow T_3$	H-3→LUMO	71	ppy→ppy	$^3\pi \rightarrow \pi^*$
				H-3→L+2	25	ppy→ppy	
4.310	287.64	0.0010	$S_0 \rightarrow S_1$	HOMO→LUMO	92	R→ppy	^{1}CT
4.370	283.69	0.0017	$S_0 \rightarrow S_2$	H-3→LUMO	93	ppy→ppy	$^1\pi \rightarrow \pi^*$
4.383	282.86	0.0405	$S_0 \rightarrow S_3$	H-1→LUMO	92	ppy+R→ppy	$^1\pi \rightarrow \pi^*$

Table S57. Singlet and triplet transitions for **L**[11]H

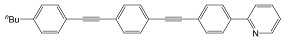

Energy (eV)	Wavelength (nm)	Osc. Strength	Transition	Major Contributions (%)		Assignment	
2.280	543.71	0.0000	$S_0 \rightarrow T_1$	HOMO→LUMO	85	ppy+R→ppy+R	$^3\pi\rightarrow\pi^*$
2.869	432.03	0.0000	$S_0 \rightarrow T_2$	H-1→LUMO	46	ppy+R→ppy+R	$^3\pi\rightarrow\pi^*$
				HOMO→L+1	38	ppy+R→ ppy+R	
3.494	354.84	0.0000	$S_0 \rightarrow T_3$	H-5→LUMO	20	ppy+R→ppy+R	$^3\pi\rightarrow\pi^*$
				H-1→L+1	27	ppy+R→ppy+R	
				HOMO→L+4	20	ppy+R→ppy+R	
3.217	385.40	2.6146	$S_0 \rightarrow S_1$	HOMO→LUMO	99	ppy+R→ppy+R	$^1\pi\rightarrow\pi^*$
3.965	312.63	0.0826	$S_0 \rightarrow S_2$	H-1→LUMO	72	ppy+R→ppy+R	$^1\pi\rightarrow\pi^*$
				HOMO→L+1	27	ppy+R→ppy+R	
4.043	306.62	0.0012	$S_0 \rightarrow S_3$	H-2→LUMO	82	py→ppy+R	$^1\pi\rightarrow\pi^*$
				H-2→L+1	17	py→ ppy+R	

Table S58. Singlet and triplet transitions for **L**[12]H

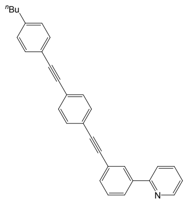

Energy (eV)	Wavelength (nm)	Osc. Strength	Transition	Major Contributions (%)		Assignment	
2.370	523.11	0.0000	$S_0 \rightarrow T_1$	HOMO→LUMO	87	ph+R→ph+R	$^3\pi\rightarrow\pi^*$
3.014	411.30	0.0000	$S_0 \rightarrow T_2$	H-1→LUMO	32	ppy+R→ph+R	$^3\pi\rightarrow\pi^*$
				H-1→L+1	12	ppy+R→ppy	
				HOMO→L+1	13	ph+R→ppy	
				HOMO→L+2	12	ph+R→ppy+R	
3.397	364.93	0.0000	$S_0 \rightarrow T_3$	H-2→LUMO	17	ppy+R→ph+R	$^3\pi\rightarrow\pi^*$
				H-1→L+1	31	ppy+R→ppy	
				HOMO→L+2	10	ph+R→ppy+R	
3.423	362.16	2.2935	$S_0 \rightarrow S_1$	HOMO→LUMO	98	ph+R→ph+R	$^1\pi\rightarrow\pi^*$
3.949	313.93	0.0442	$S_0 \rightarrow S_2$	H-1→LUMO	10	ppy+R→ph+R	$^1\pi\rightarrow\pi^*$
				HOMO→L+1	81	ph+R→ppy	
4.001	309.85	0.0217	$S_0 \rightarrow S_3$	H-1→LUMO	84	ppy+R→ph+R	$^1\pi\rightarrow\pi^*$

Table S59. Singlet and triplet transitions for $\mathbf{L^{13}}$H

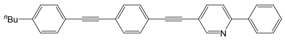

Energy (eV)	Wavelength (nm)	Osc. Strength	Transition	Major Contributions (%)		Assignment	
2.279	543.86	0.0000	$S_0 \rightarrow T_1$	HOMO→LUMO	85	ppy+R→ppy+R	$^3\pi \rightarrow \pi^*$
2.861	433.32	0.0000	$S_0 \rightarrow T_2$	H-1→LUMO	47	ppy+R→ppy+R	$^3\pi \rightarrow \pi^*$
				HOMO→L+1	37	ppy+R→ppy+R	
3.463	357.99	0.0000	$S_0 \rightarrow T_3$	H-5→LUMO	31	ppy+R→ppy+R	$^3\pi \rightarrow \pi^*$
				H-1→L+1	26	ppy+R→ppy+R	
				HOMO→L+4	20	ppy+R→ppy+R	
3.202	387.19	2.5809	$S_0 \rightarrow S_1$	HOMO→LUMO	99	ppy+R→ppy+R	$^1\pi \rightarrow \pi^*$
3.925	315.86	0.0956	$S_0 \rightarrow S_2$	H-1→LUMO	85	ppy+R→ppy+R	$^1\pi \rightarrow \pi^*$
				HOMO→L+1	13	ppy+R→ppy+R	
3.938	314.82	0.0005	$S_0 \rightarrow S_3$	H-2→LUMO	91	py+R→ppy+R	$^1\pi \rightarrow \pi^*$

Table S60. Singlet and triplet transitions for $\mathbf{L^{14}}$H

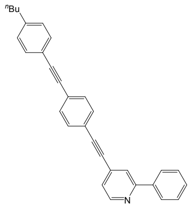

Energy (eV)	Wavelength (nm)	Osc. Strength	Transition	Major Contributions (%)		Assignment	
2.362	524.88	0.0000	$S_0 \rightarrow T_1$	HOMO→LUMO	82	py+R→py+R	$^3\pi \rightarrow \pi^*$
3.027	409.48	0.0000	$S_0 \rightarrow T_2$	H-2→LUMO	18	py+R→py+R	$^3\pi \rightarrow \pi^*$
				H-1→LUMO	19	ppy→py+R	
				H-1→L+1	24	ppy→ppy+R	
				HOMO→L+1	12	py+R→ppy+R	
				HOMO→L+2	10	py+R→ppy+R	
3.326	372.77	0.0000	$S_0 \rightarrow T_3$	H-2→LUMO	25	py+R→py+R	$^3\pi \rightarrow \pi^*$
				H-1→LUMO	13	ppy→py+R	
				H-1→L+1	25	ppy→ppy+R	
				HOMO→L+2	11	py+R→ppy+R	
3.352	369.78	2.0786	$S_0 \rightarrow S_1$	HOMO→LUMO	98	py+R→py+R	$^1\pi \rightarrow \pi^*$
3.906	317.34	0.0466	$S_0 \rightarrow S_2$	H-1→LUMO	88	ppy→py+R	$^1\pi \rightarrow \pi^*$
4.009	309.23	0.0020	$S_0 \rightarrow S_3$	H-3→LUMO	87	py→py+R	$^1\pi \rightarrow \pi^*$
				H-3→L+1	10	py→ppy+R	

Table S61. Singlet and triplet transitions for **L**[15]H

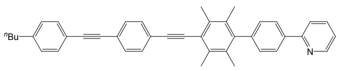

Energy (eV)	Wavelength (nm)	Osc. Strength	Transition	Major Contributions (%)		Assignment	
2.366	523.82	0.0000	$S_0 \rightarrow T_1$	HOMO→LUMO	89	R→R	$^3\pi \rightarrow \pi^*$
3.106	399.15	0.0000	$S_0 \rightarrow T_2$	H-3→LUMO	49	R→R	$^3\pi \rightarrow \pi^*$
				HOMO→L+3	33	R→R	
3.164	391.79	0.0000	$S_0 \rightarrow T_3$	H-2→L+1	81	ppy→ppy	$^3\pi \rightarrow \pi^*$
3.386	366.09	2.5866	$S_0 \rightarrow S_1$	HOMO→LUMO	98	R→R	$^1\pi \rightarrow \pi^*$
3.988	310.80	0.0186	$S_0 \rightarrow S_2$	H-1→LUMO	94	R→R	$^1\pi \rightarrow \pi^*$
3.989	305.50	0.0042	$S_0 \rightarrow S_3$	H-3→LUMO	92	R→R	$^1\pi \rightarrow \pi^*$

Table S62. Singlet and triplet transitions for **L**[16]H

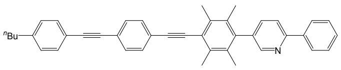

Energy (eV)	Wavelength (nm)	Osc. Strength	Transition	Major Contributions (%)		Assignment	
2.367	523.80	0.0000	$S_0 \rightarrow T_1$	HOMO→LUMO	89	R→R	$^3\pi \rightarrow \pi^*$
3.103	399.49	0.0000	$S_0 \rightarrow T_2$	H-3→LUMO	47	R→R	$^3\pi \rightarrow \pi^*$
				HOMO→L+3	33	R→R	
3.163	391.88	0.0000	$S_0 \rightarrow T_3$	H-2→L+1	18	ppy+R→ppy	$^3\pi \rightarrow \pi^*$
				H-1→L+1	64	ppy+R→ppy	
3.385	366.18	2.5973	$S_0 \rightarrow S_1$	HOMO→LUMO	98	R→R	$^1\pi \rightarrow \pi^*$
3.958	313.17	0.0001	$S_0 \rightarrow S_2$	HOMO→L+1	90	R→ppy	^{1}CT
4.042	306.72	0.0195	$S_0 \rightarrow S_3$	H-2→LUMO	67	ppy+R→R	$^1\pi \rightarrow \pi^*$
				H-1→LUMO	25	ppy+R→R	

Table S63. Singlet and triplet transitions for **L**[17]H

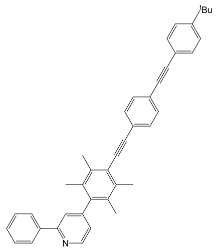

Energy (eV)	Wavelength (nm)	Osc. Strength	Transition	Major Contributions (%)		Assignment	
2.369	523.31	0.0000	$S_0 \rightarrow T_1$	HOMO→LUMO	89	R→R	$^3\pi \rightarrow \pi^*$
3.108	398.85	0.0000	$S_0 \rightarrow T_2$	H-3→LUMO	49	R→R	$^3\pi \rightarrow \pi^*$
				HOMO→L+3	31	R→R	
3.198	387.62	0.0000	$S_0 \rightarrow T_3$	H-2→L+1	52	ppy+R→ppy	$^3\pi \rightarrow \pi^*$
				H-1→L+1	27	ppy+R→ppy	
3.395	365.15	2.4929	$S_0 \rightarrow S_1$	HOMO→LUMO	98	R→R	$^1\pi \rightarrow \pi^*$
3.995	310.30	0.0001	$S_0 \rightarrow S_2$	HOMO→L+1	92	R→ppy	^{1}CT
4.001	309.86	0.0193	$S_0 \rightarrow S_3$	H-2→LUMO	17	ppy+R→R	$^1\pi \rightarrow \pi^*$
				H-1→LUMO	73	ppy+R→R	

Calculated singlet and triplet state energies

The $S_0 \rightarrow T_n$ and $S_0 \rightarrow S_n$ transition energies calculated by time-dependent DFT (TD-DFT) calculations to determine the T_n and S_n states energy respectively.

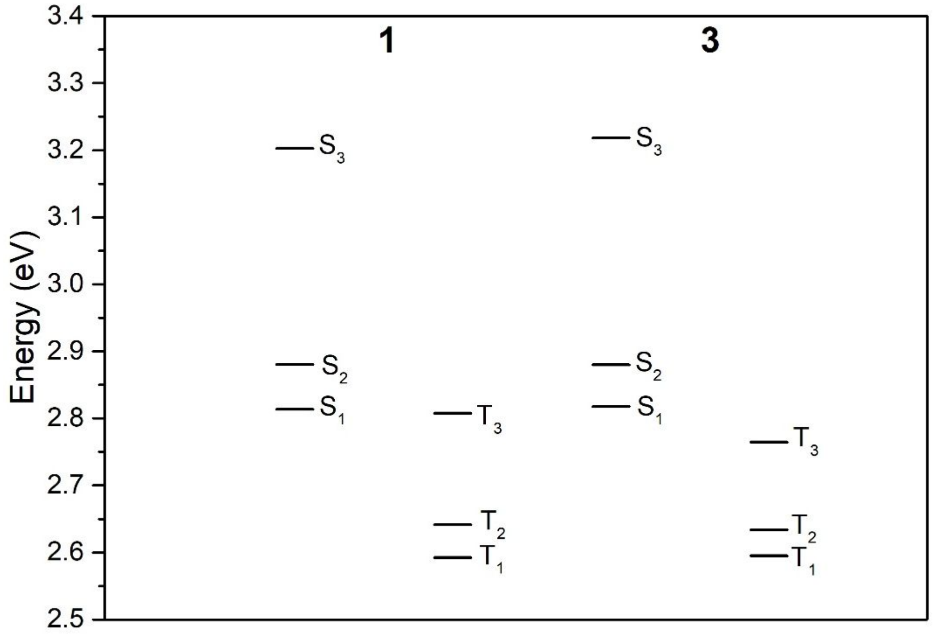

Figure S59. Calculated singlet and triplet state energies of complexes **1** and **3**.

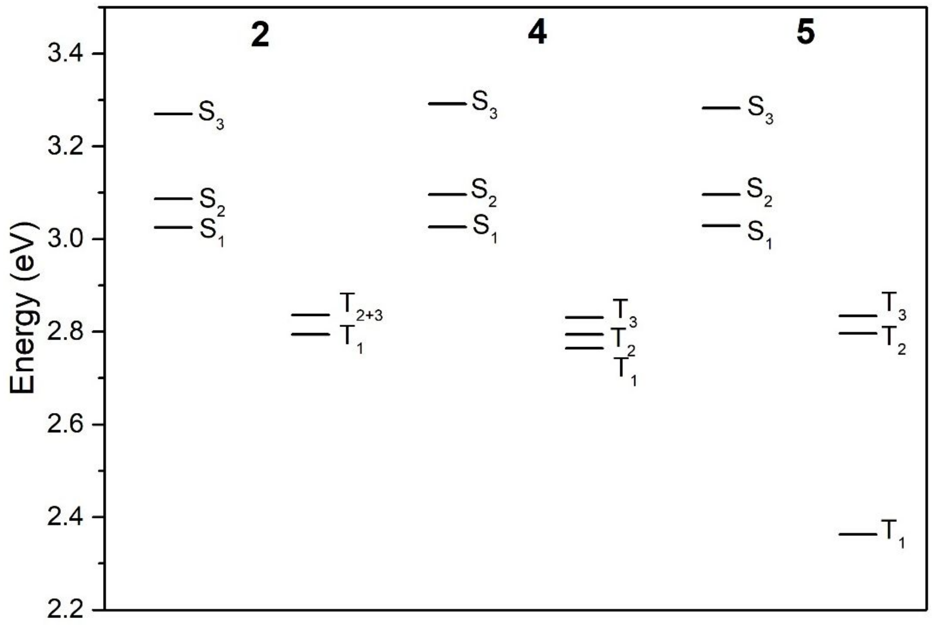

Figure S60. Calculated singlet and triplet state energies of complexes **2**, **4** and **5**.

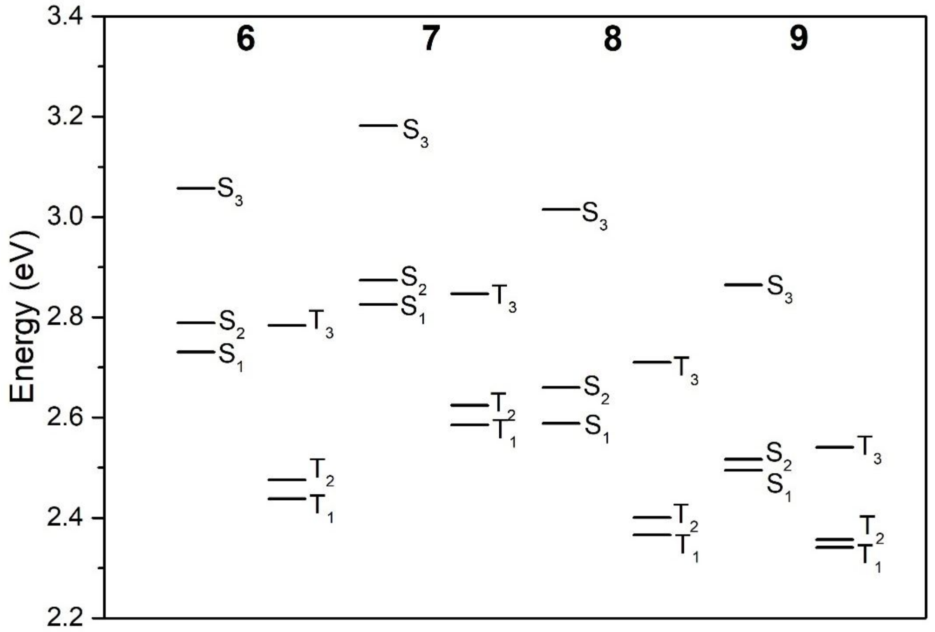

Figure S61. Calculated singlet and triplet state energies of complexes **6**, **7**, **8** and **9**.

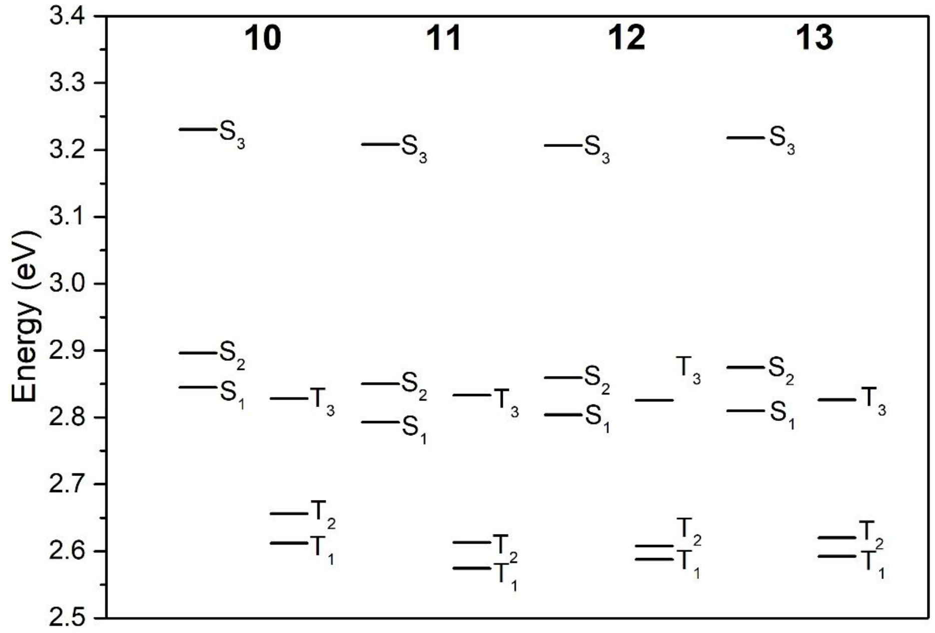

Figure S62. Calculated singlet and triplet state energies of complexes **10**, **11**, **12** and **13**.

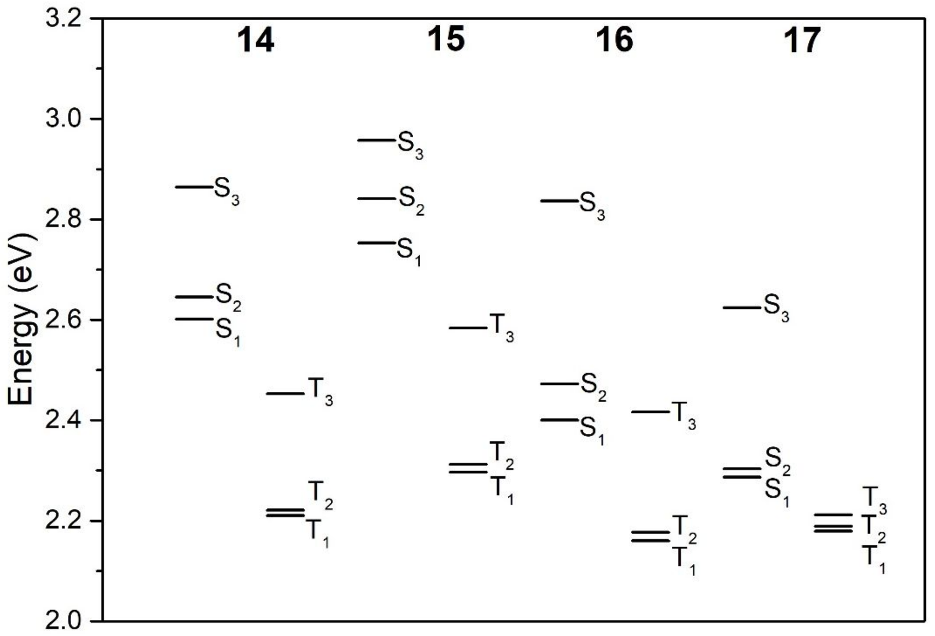

Figure S63. Calculated singlet and triplet state energies of complexes **14**, **15**, **16** and **17**.

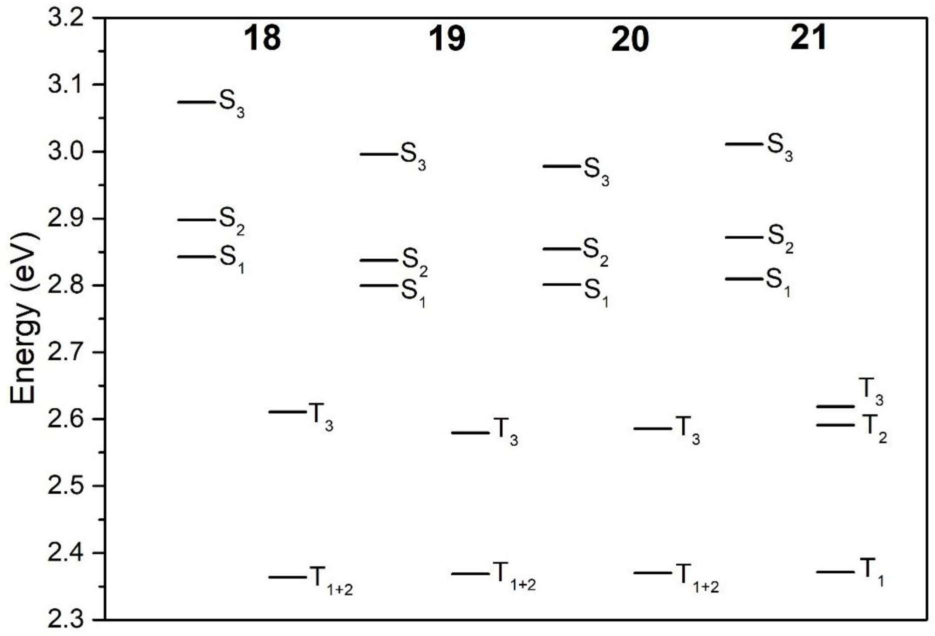

Figure S64. Calculated singlet and triplet state energies of complexes **18**, **19**, **20** and **21**.

Table S64. Summary of $S_0 \rightarrow T_1$ transitions including largest contribution

Complex	Wavelength (nm)	Largest contribution (%)	
**1**	478.29	HOMO→LUMO	89
**2**	443.71	HOMO→LUMO	76
**3**	477.81	HOMO→LUMO	88
**4**	448.60	H-2→L+2	88
**5**	524.82	H-2→LUMO	82
**6**	508.56	HOMO→LUMO	79
**7**	479.62	HOMO→LUMO	84
**8**	524.11	HOMO→LUMO	84
**9**	529.62	HOMO→L+1	91
**10**	474.70	HOMO→LUMO	86
**11**	481.66	HOMO→LUMO	87
**12**	479.14	HOMO→LUMO	88
**13**	478.27	HOMO→LUMO	87
**14**	561.01	HOMO→LUMO	38
**15**	539.85	HOMO→LUMO	49
**16**	574.08	HOMO→LUMO	56
**17**	568.94	HOMO→L+1	86
**18**	524.55	H-2→L+1	44
**19**	523.49	H-3→LUMO<br>H-2→L+1	44<br>44
**20**	523.16	H-2→L+1	85
**21**	522.91	H-2→LUMO	89

Transition dipole moments

Dipole moments (DM) and transition dipole moments (TDM) were calculated using the Orca package[4] and figures of dipole and transition moments below were generated with Avogadro software[5]. Both moments in each complex were predicted using the model chemistry B3LYP/TZVP:def2-TZVP on the B3LYP/3-21G*:LANL2DZ optimised geometries generated from the Gaussian09 package. The model chemistry CAM-B3LYP/TZVP:def2-TZVP was also used where every complex had the TDM perpendicular to the corresponding DM. Table S65 lists the varied angles between the DM and TDM at B3LYP/TZVP:def2-TZVP and compared with the observed emission lifetimes. No clear relationship between the moments and lifetimes was found.

Table S65. Dipole moments and angles between dipole moments and transition dipole moments compared with observed emission lifetimes in aOPE3, pOPE and dOPE complexes.

Complex	DM (Debye)	Angle DM vs TDM deg (°)	Direction-independent angle deg (°)	Lifetime µs
**5**	0.59	3.95	3.95	180.00
**14**	2.89	96.84	83.16	5.95
**15**	5.68	0.74	0.74	32.80
**16**	3.02	102.47	77.53	11.50
**17**	6.60	177.34	2.66	0.69
**18**	6.54	0.86	0.86	49.50
**19**	4.24	179.90	0.10	81.90
**20**	5.62	178.60	1.40	625.00
**21**	5.62	80.80	80.80	181.80

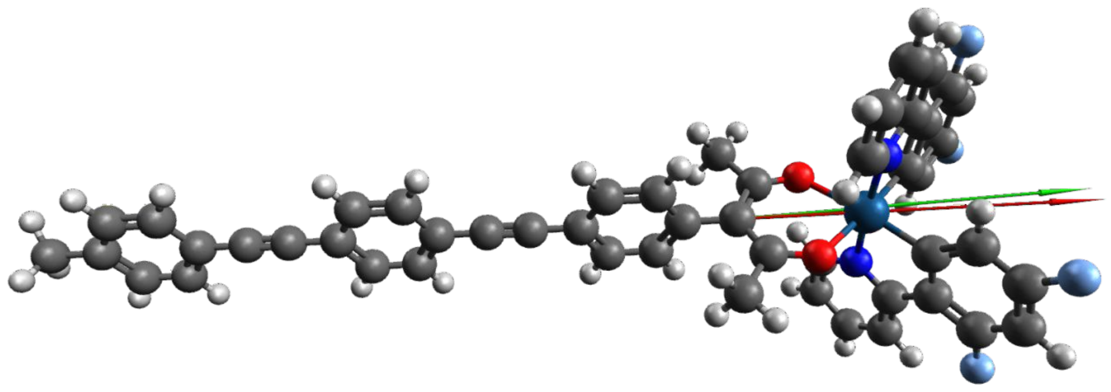

Figure S65. Complex **5**, dipole moment (green arrow) and transition dipole moment for the lowest energy transition (red arrow), not to scale.

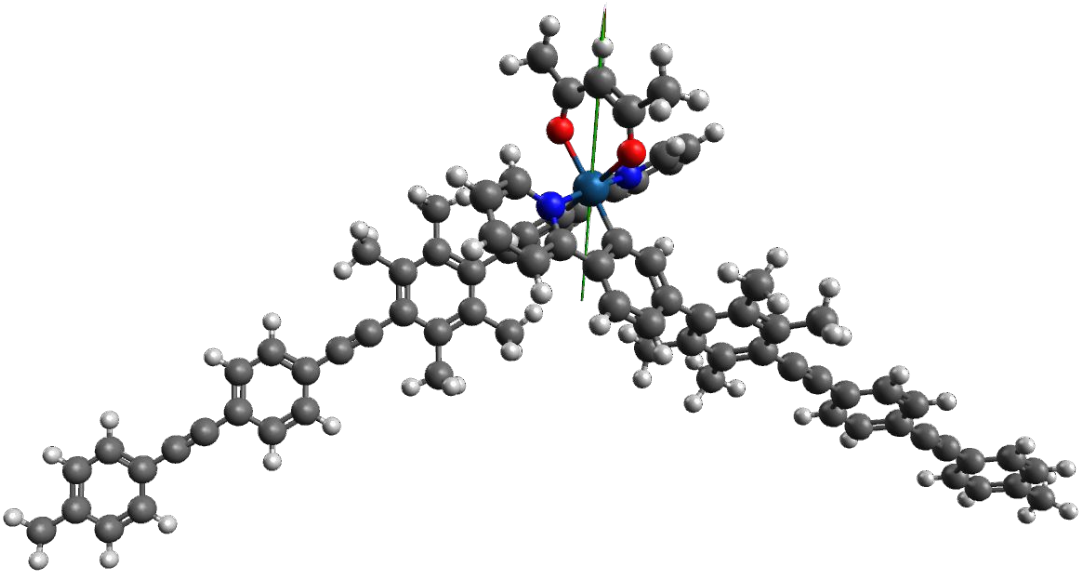

Figure S66. Complex **18**, dipole moment (green arrow) and transition dipole moment for the lowest energy transition (red arrow), not to scale.

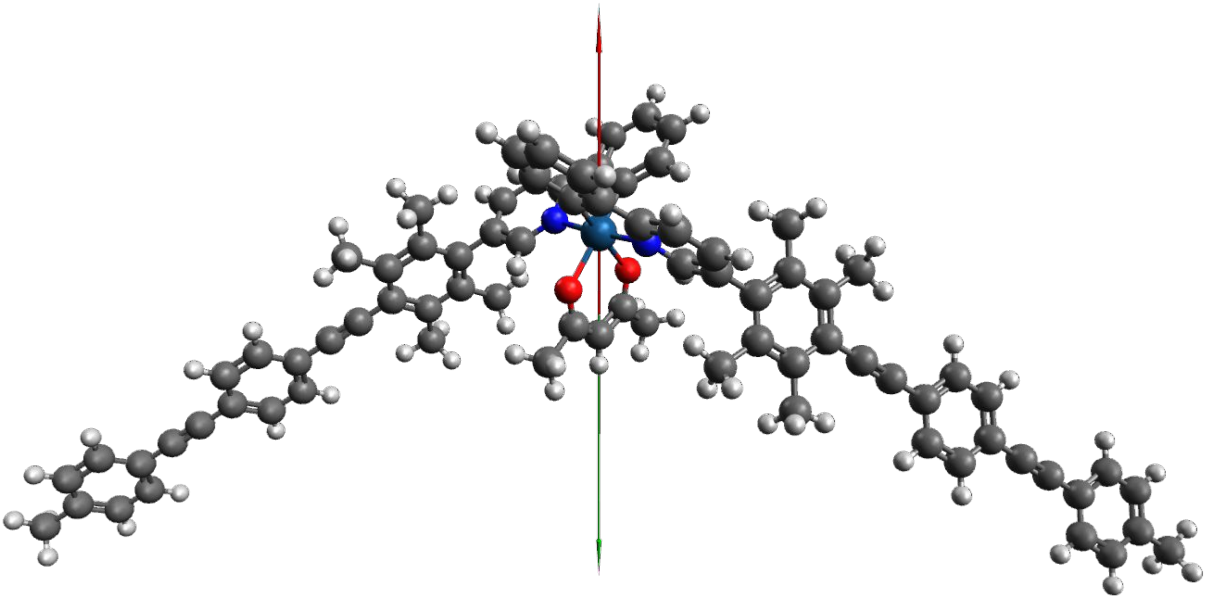

Figure S67. Complex **19**, dipole moment (green arrow) and transition dipole moment for the lowest energy transition (red arrow), not to scale.

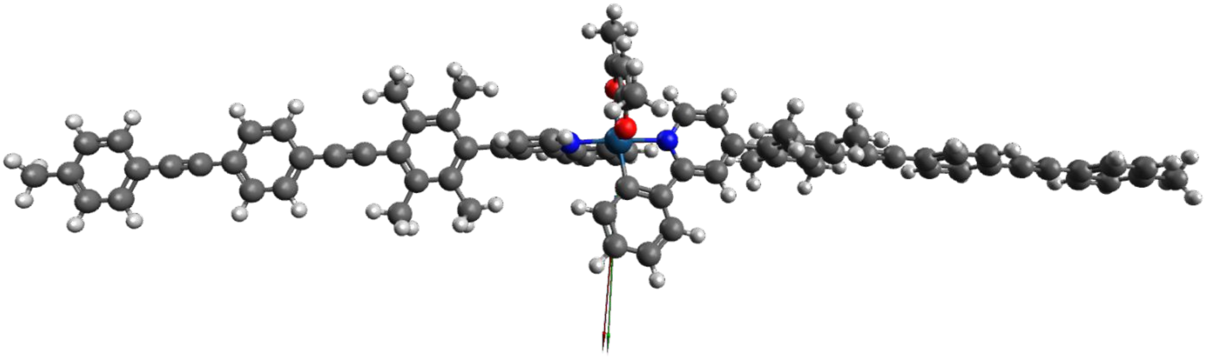

Figure S68. Complex **20**, dipole moment (green arrow) and transition dipole moment for the lowest energy transition (red arrow), not to scale.

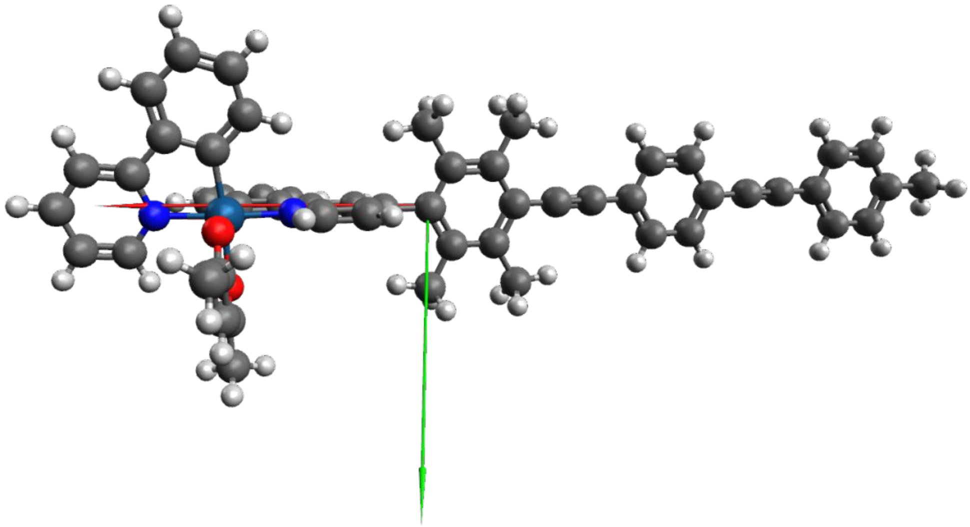

Figure S69. Complex **21**, dipole moment (green arrow) and transition dipole moment for the lowest energy transition (red arrow), not to scale.

S6. Physical Measurements

Instrumentation. All photophysical measurements of iridium complexes were performed using DCM as the solvent.

The UV-Visible spectra were measured on a Unicam UV2-100 spectrometer operated with the Unicam Vison software in quartz cuvettes with path length l = 1 cm.

Excitation and emission photoluminescence spectra were recorded on a Horiba Jobin Yvon SPEX Fluorolog 3-22 spectrofluorometer. Samples were degassed by repeated freeze-pump-thaw cycles using a turbomolecular pump until the pressure was stable in quartz cuvettes, l = 1 cm. The solutions had absorbance below 0.15 to minimise inner filter effects. PLQYs were measured following our previously reported method:[6]

Quinine sulfate in 0.1 M H_2SO_4 (Φ_F: 0.546)[7 7 5] or rhodamine 101 in ethanol (Φ_F: 1.00)[8] as the references, the emission spectra of quinine sulfate were collected by exciting the samples at 360 nm. The PLQY of each complex was measured in duplicate and determined by the following method:

1. The UV-vis absorbance spectrum was recorded in quartz cuvette with path length l = 2 cm (to improve spectrum signal-to-noise), recording the absorbance at the excitation wavelength.
2. The same sample solution was transferred to the quartz fluorescence cell (a standard 1 cm cell modified with a Teflon Young's tap) degassed via repeated freeze-pump-thaw cycles before the emission spectrum was recorded.
3. The fully corrected fluorescence spectrum was integrated and the integrated intensity (the area of the fluorescence spectrum) was recorded.
4. Steps 1 to 3 were repeated for five additional solutions with increasing concentrations (with absorbance ranging from 0.02 to 0.1).
5. The integrated fluorescence intensity was plotted verse absorbance, which resulted a linear plot with gradient X ($Grad_X$).
6. Steps 1, 3, 4 and 5 were repeated for the chosen standard (quinine sulfate).

7. Fluorescence quantum yield for each complex was calculated using the following equation

$$\Phi_X = \Phi_{ST}\left(\frac{Grad_X}{Grad_{ST}}\right)\left(\frac{\eta^2{}_X}{\eta^2{}_{ST}}\right)$$

Where ST and X denote standard and the measured complex, Φ is the fluorescence quantum yield, Grad the gradient from the plot of integrated fluorescence intensity vs. absorbance, and η the refractive index of the solvent.

Emission lifetimes were determined by using a custom spectrometer; measured by time-correlated single photon counting (TCSPC) using a pulsed diode laser (371 nm), made by IBH Ltd, running at 1 MHz. The fluorescence emission was collected at right angles to the excitation source. The emission wavelength was selected using a Horiba Jobin Yvon Triax 190 monochromator and detected by a cooled IBH TBX-04 PMT. Timing was achieved using an Ortec 567 time-to-amplitude converter and an E. G. & G Trumpcard pulse height analyser (PHA), and data was recorded using Maestro (ver.510) software. The data were transferred to a PC and analysed using non-linear regression to a single exponential decay, and the quality of fit established by reduced χ^2 and random residuals. The samples were degassed by repeated freeze-pump-thaw cycles in duplicates. The decay data were fitted to a single exponential function. Low temperature emission spectra and lifetime data were measured in a DN1704 optical cryostat (Oxford Instruments) with a ITC601 temperature controller (Oxford Instruments).

Electrochemical analyses of the iridium complexes were carried out using a PalmSens EmStat^2 potentiometer, with platinum working, platinum counter and platinum pseudo reference electrodes, from solutions in DCM containing 0.1 M supporting electrolyte (tetrabutylammonium hexafluorophosphate, $TBAPF_6$), scan rate = 100 mV s^{-1}. The ferrocene/ferrocenium couple was used as the internal reference at 0.0 V.

Electrochemical data

Table S66. electrochemical data for complexes **1-21** recorded in DCM

Compound	$E_{½}$(ox) ($V_{FeCp_2/FeCp_2^+}$)	Observed HOMO (eV)	Calculated HOMO (eV)
**1**	0.39	-5.19	-5.11
**2**	0.71	-5.51	-5.47
**3**	0.41	-5.21	-5.13
**4**	0.72	-5.52	-5.49
**5**	0.71	-5.51	-5.49
**6**	0.52	-5.32	-5.27
**7**	0.50	-5.30	-5.20
**8**	0.51	-5.31	-5.19
**9**[7]	0.38	-5.18	-5.20
**10**	0.42	-5.22	-5.16
**12**[7]	0.35	-5.15	-5.14
**13**	0.44	-5.24	-5.13
**14**	0.53	-5.33	-5.27
**15**	0.49	-5.29	-5.12
**16**	0.51	-5.31	-5.19
**17**[7]	0.38	-5.18	-5.19
**18**	0.44	-5.24	-5.17
**19**	0.43	-5.23	-5.13
**20**[7]	0.35	-5.15	-5.14
**21**	0.44	-5.24	-5.13

Observed HOMO values were determined using $FeCp_2$ HOMO = -4.8 eV[8] which approximates to the formula Observed HOMO(eV) = -[4.8 + $E_{½}$(ox)].

Calculated HOMO(eV) at B3LYP/3-21G*:LANL2DZ (see Tables S4-S24).

UV-Visible electronic absorbance

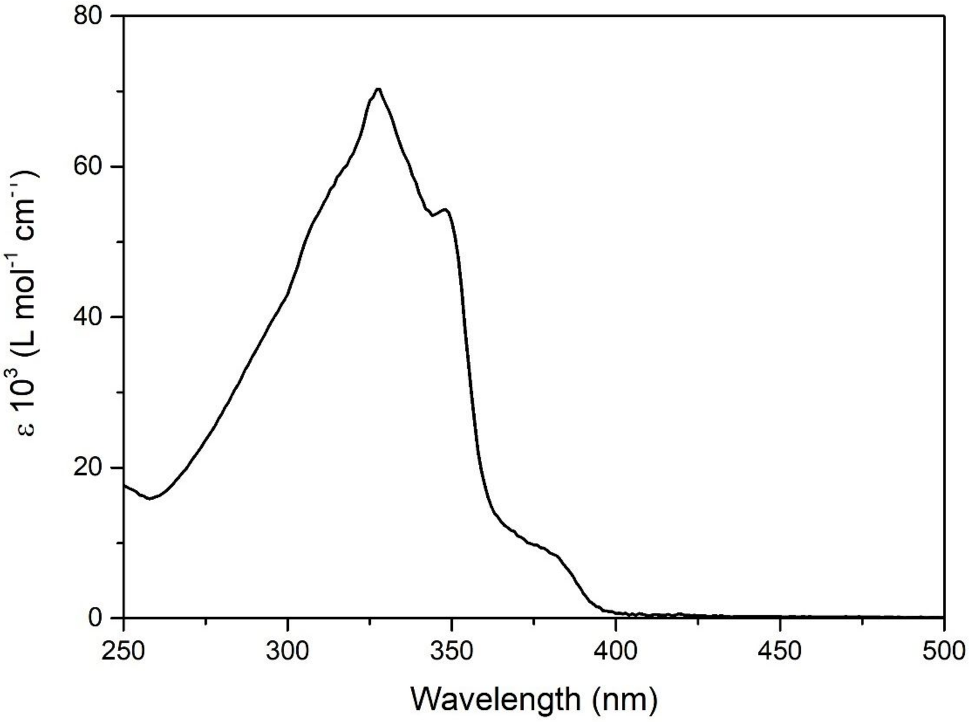

Figure S70. UV-Visible electronic absorbance of **L**^{3}H recorded in DCM.

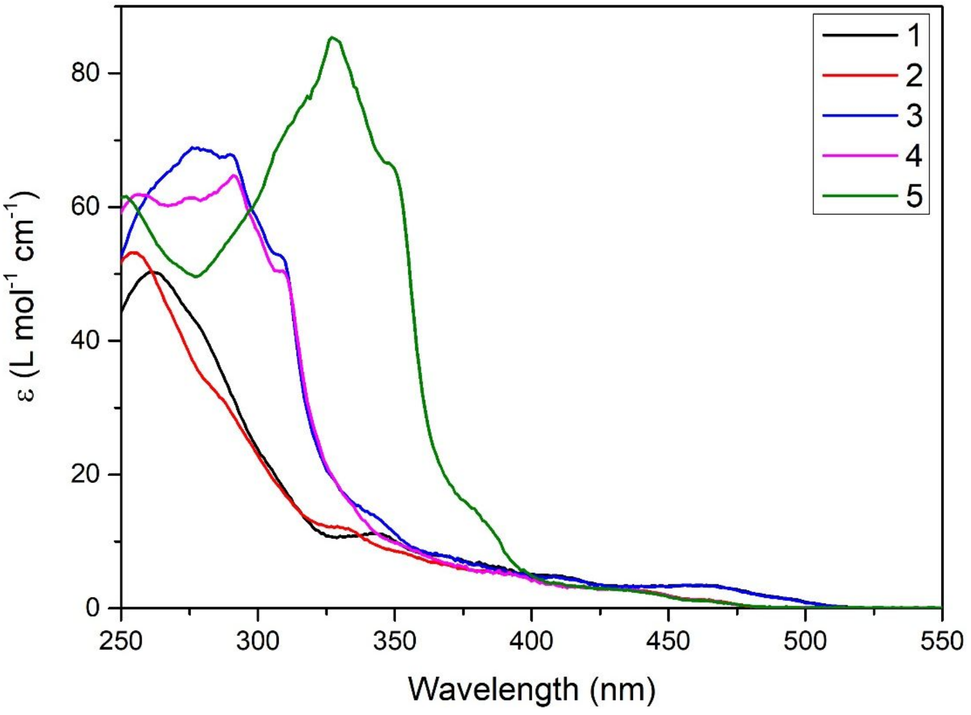

Figure S71. UV-Visible electronic absorbance of complexes **1**-**5** recorded in DCM.

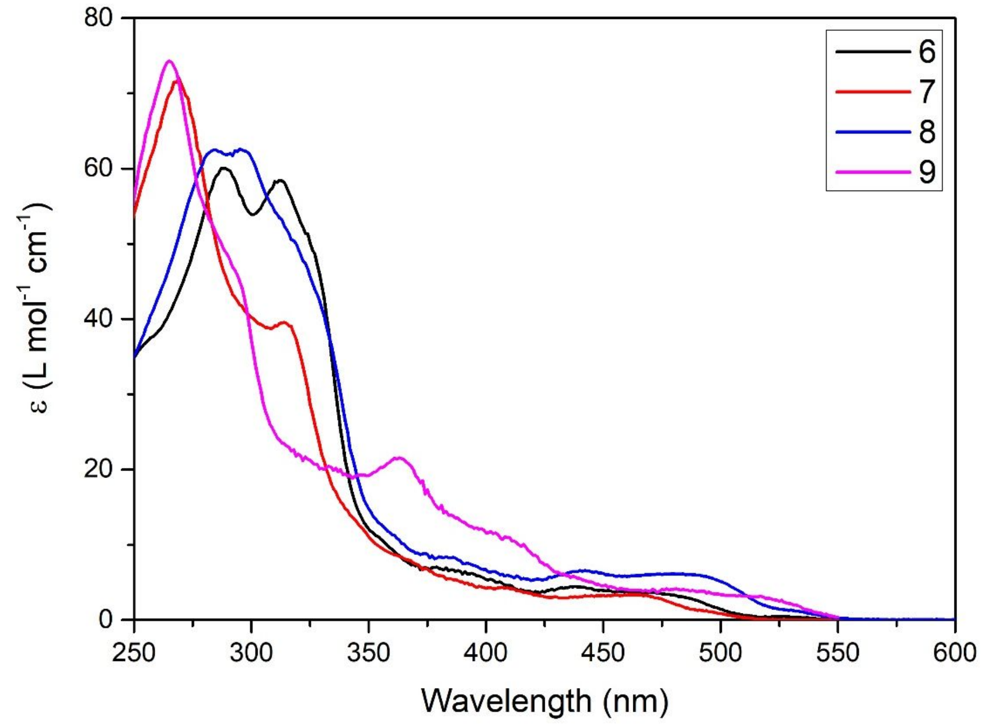

Figure S72. UV-Visible electronic absorbance of complexes **6-9** recorded in DCM.

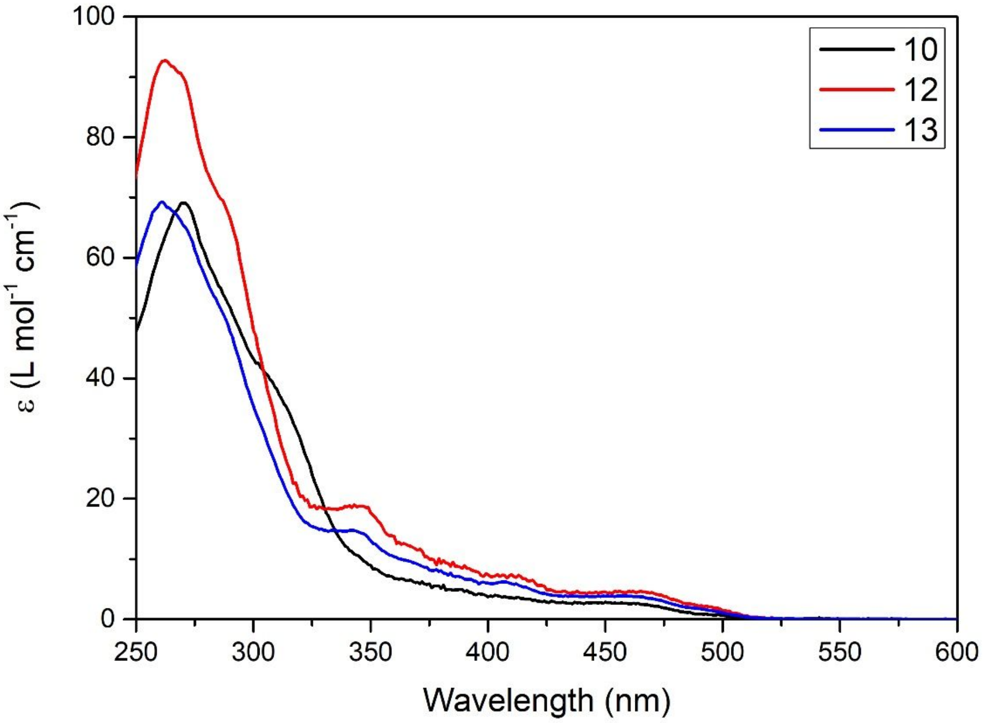

Figure S73. UV-Visible electronic absorbance of complexes **10**, **12**, and **13** recorded in DCM.

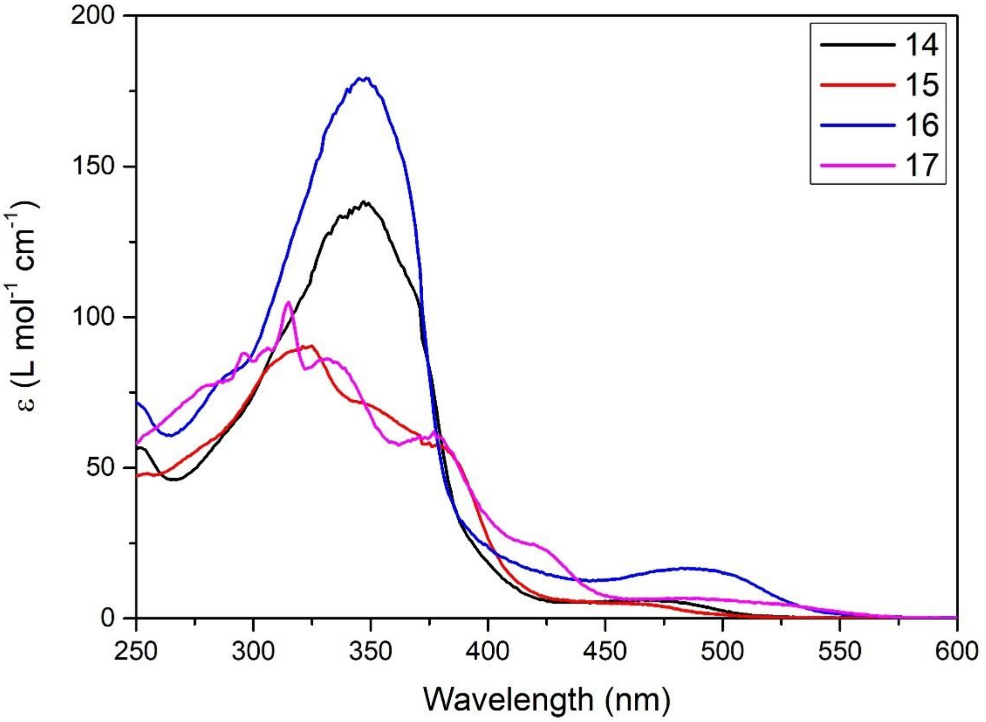

Figure S74. UV-Visible electronic absorbance of complexes **14-17** recorded in DCM.

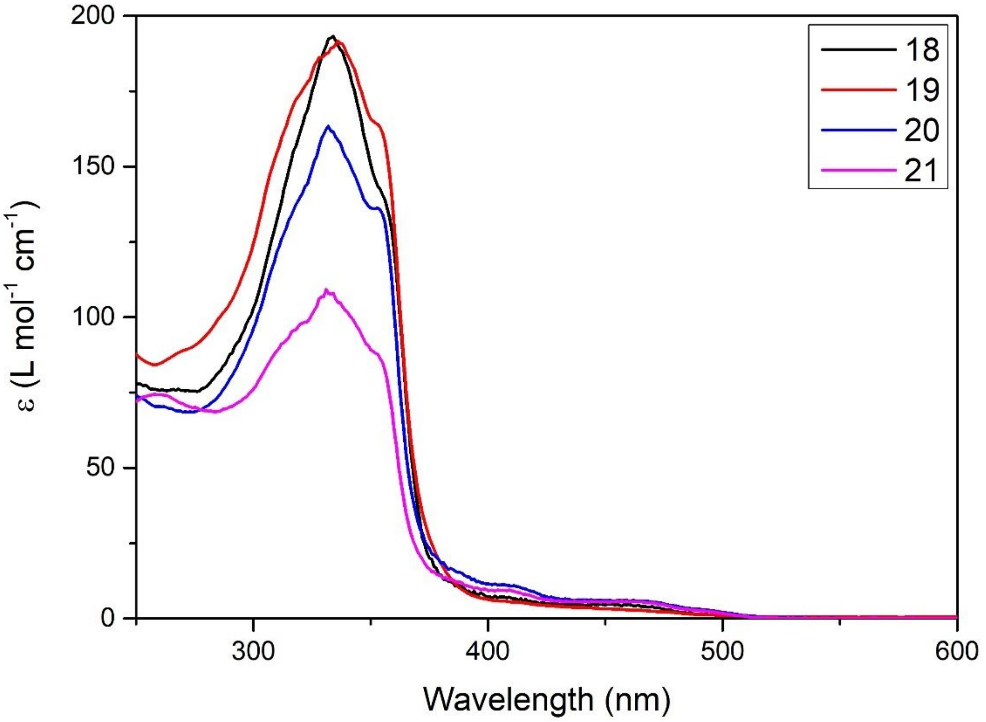

Figure S75. UV-Visible electronic absorbance of complexes **18-21** recorded in DCM.

Steady-state emission

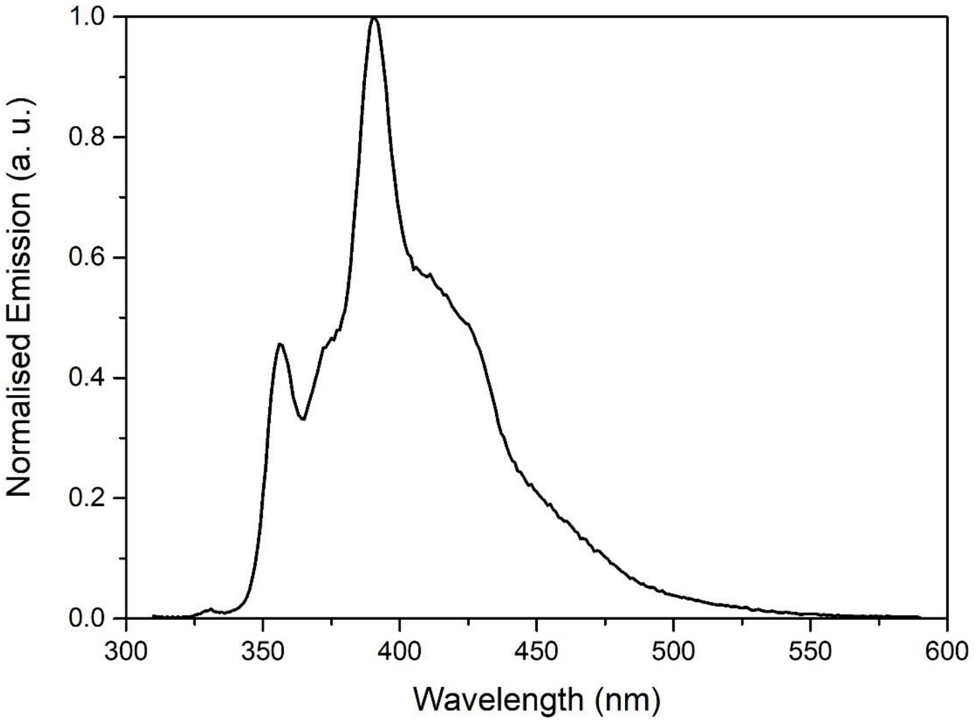

Figure S76. Steady-State emission of **L**^{3}H recorded in DCM.

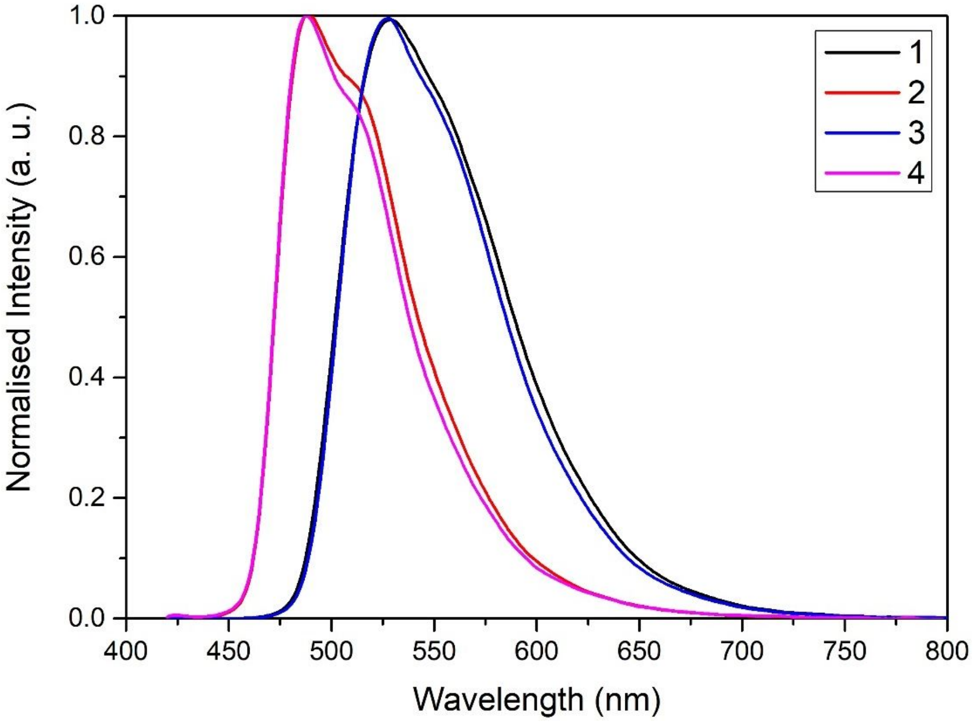

Figure S77. Steady-State emission of complexes **1-4** recorded in DCM.

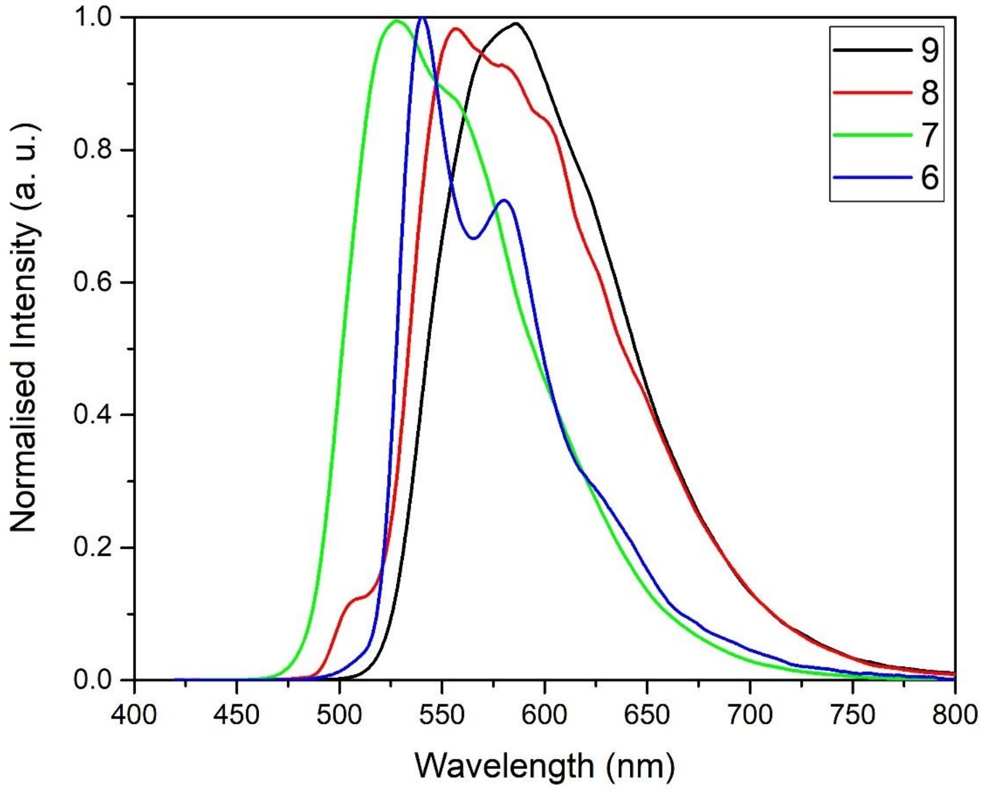

Figure S78. Steady-State emission of complexes **6-9** recorded in DCM.

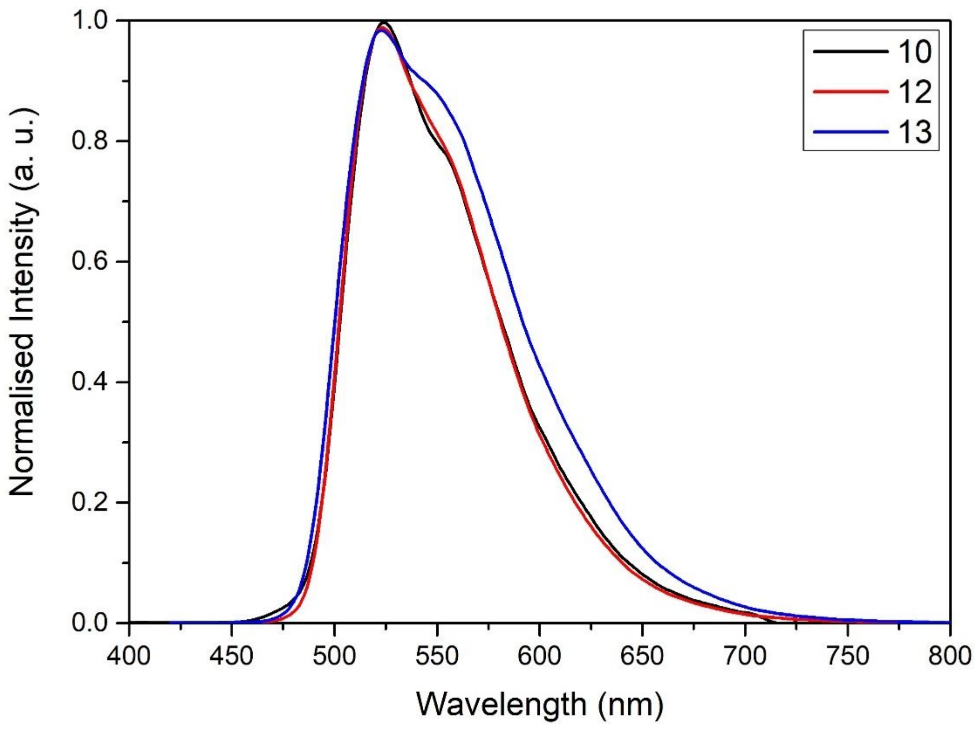

Figure S79. Steady-State emission of complexes **10**, **12**, and **13** recorded in DCM.

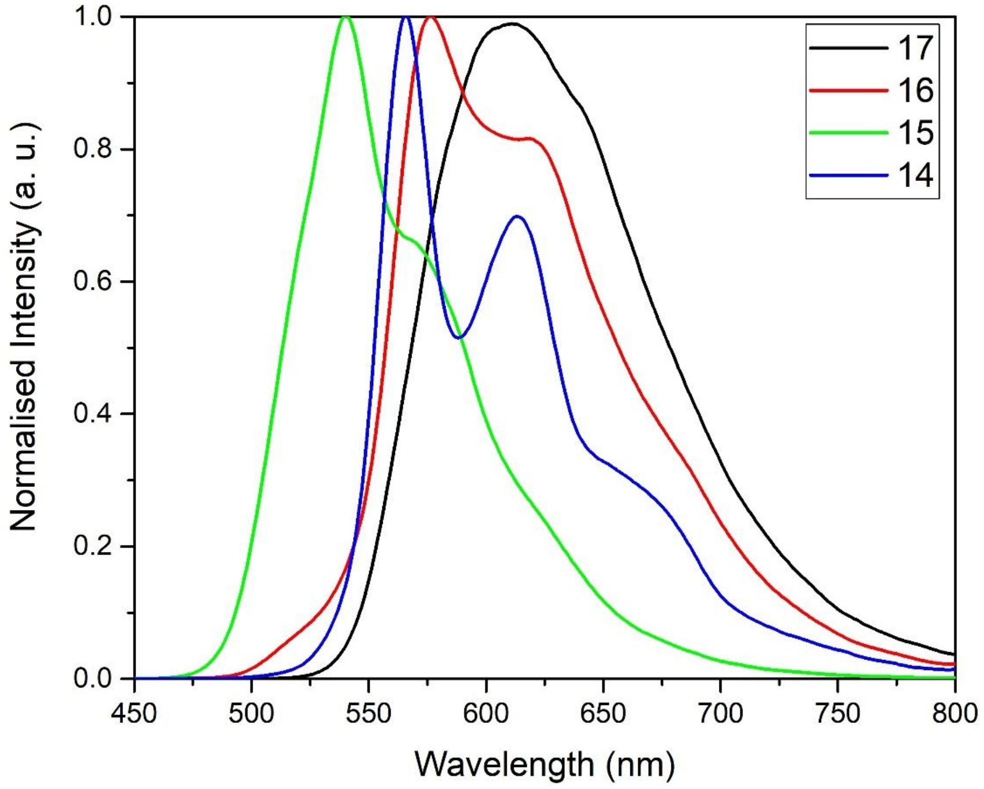

Figure S80. Steady-State emission of complexes **14-17** recorded in DCM.

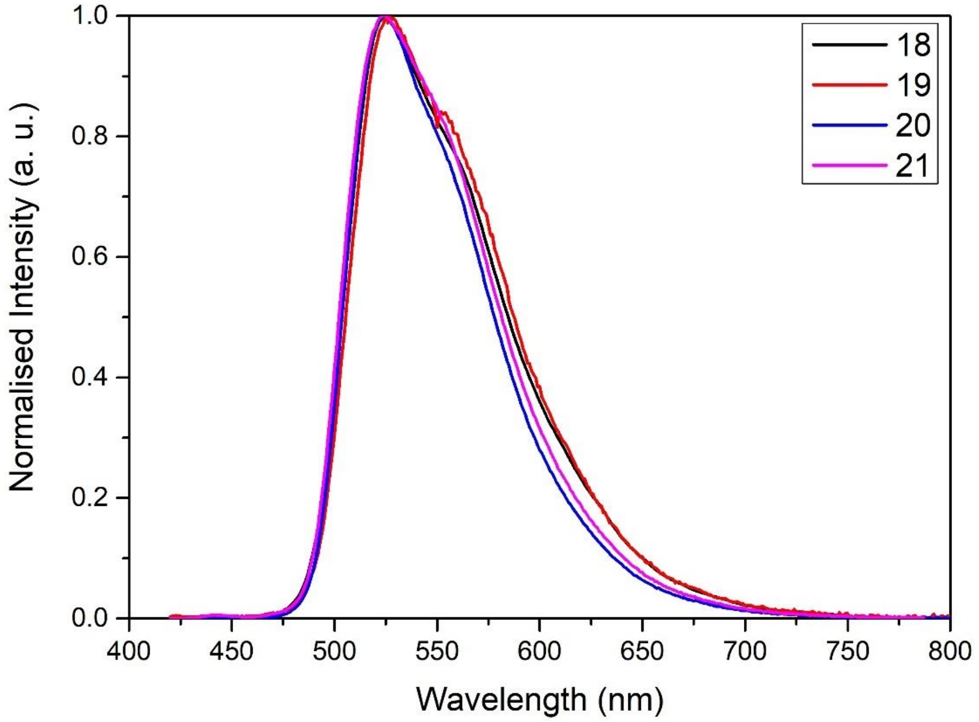

Figure S81. Steady-State emission of complexes **18-21** recorded in DCM.

Low Temperature Emission

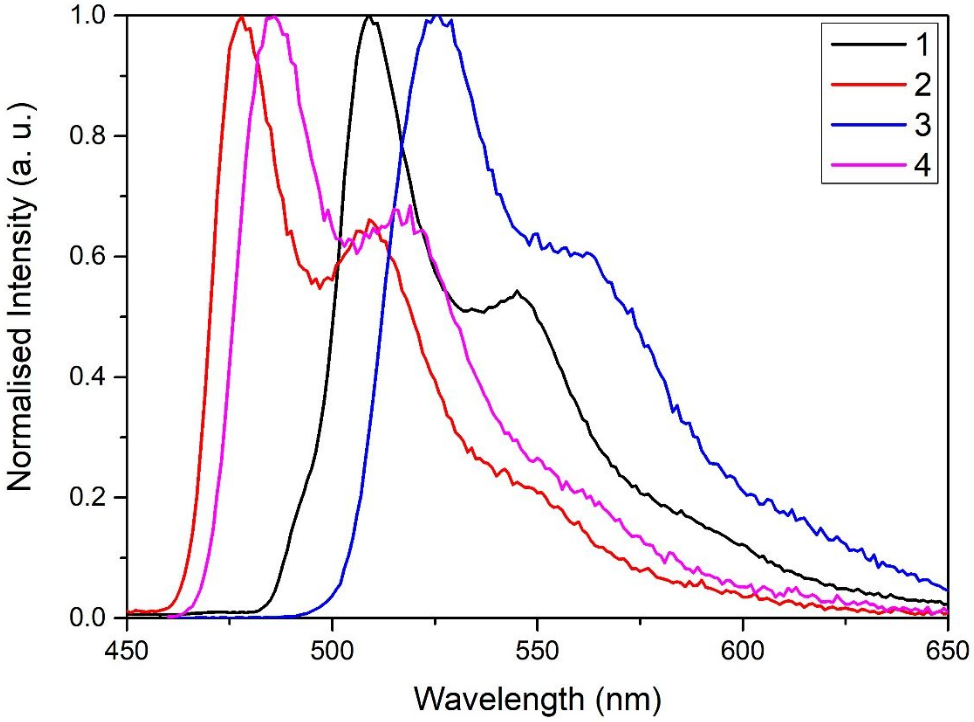

Figure S82. Emission of complexes **1**-**4** at 77K recorded in MeTHF.

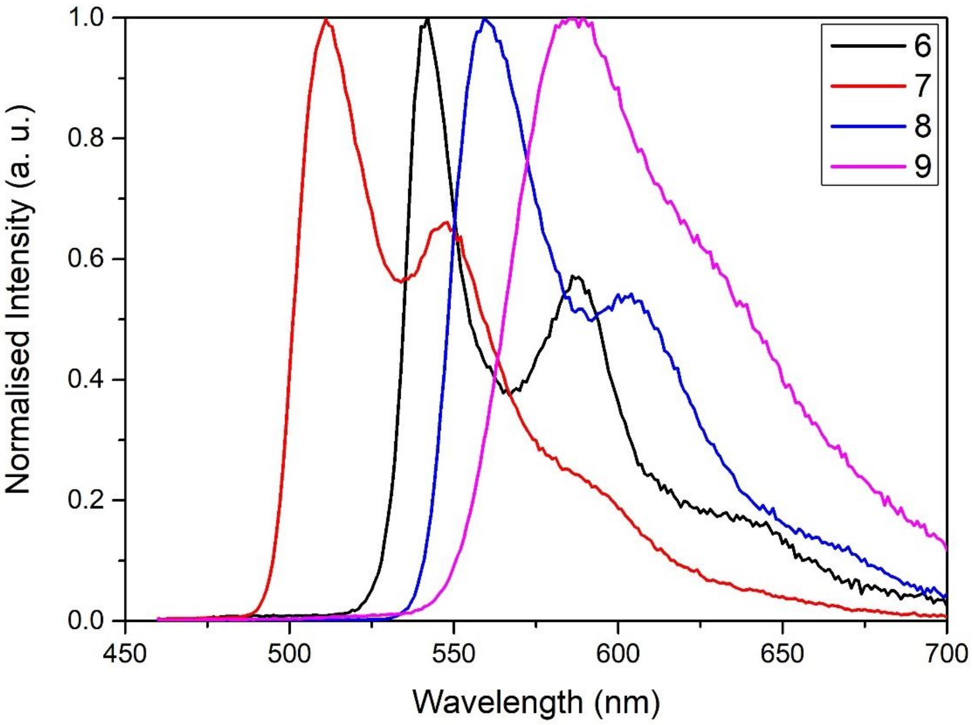

Figure S83. Emission of complexes **6-9** at 77K recorded in MeTHF.

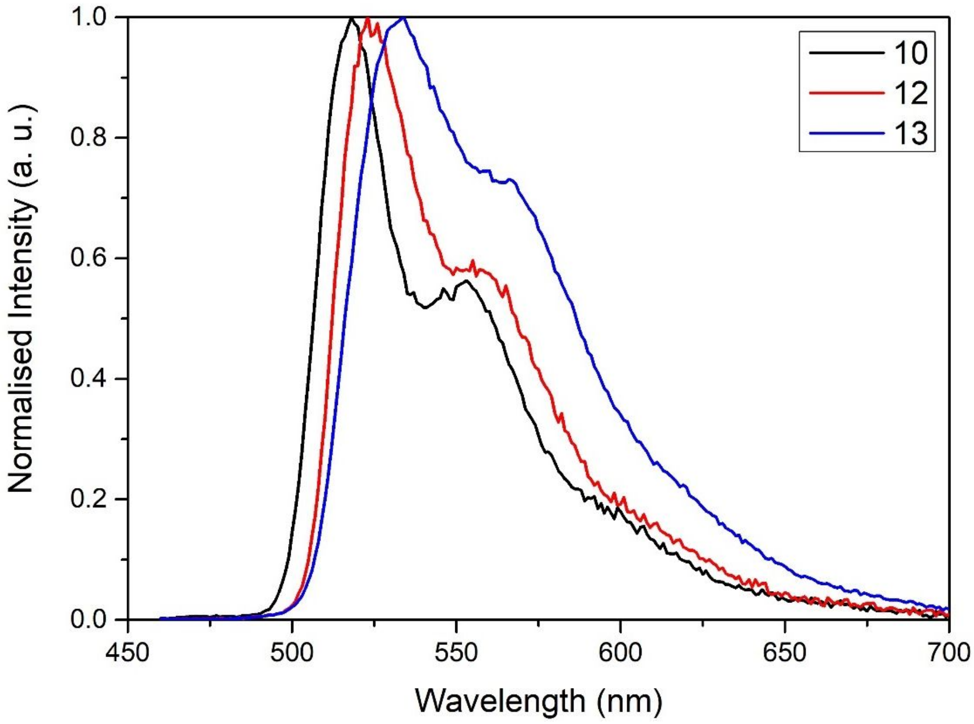

Figure S84. Emission of complexes **10,12**, and **13** at 77K recorded in MeTHF.

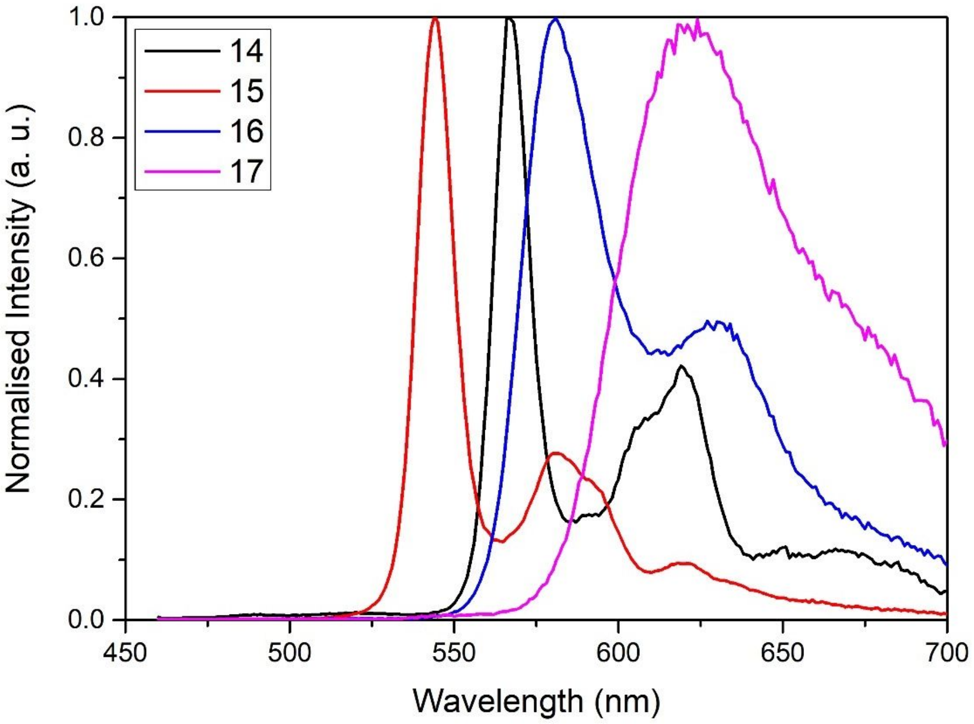

Figure S85. Emission of complexes **14-17** at 77K recorded in MeTHF.

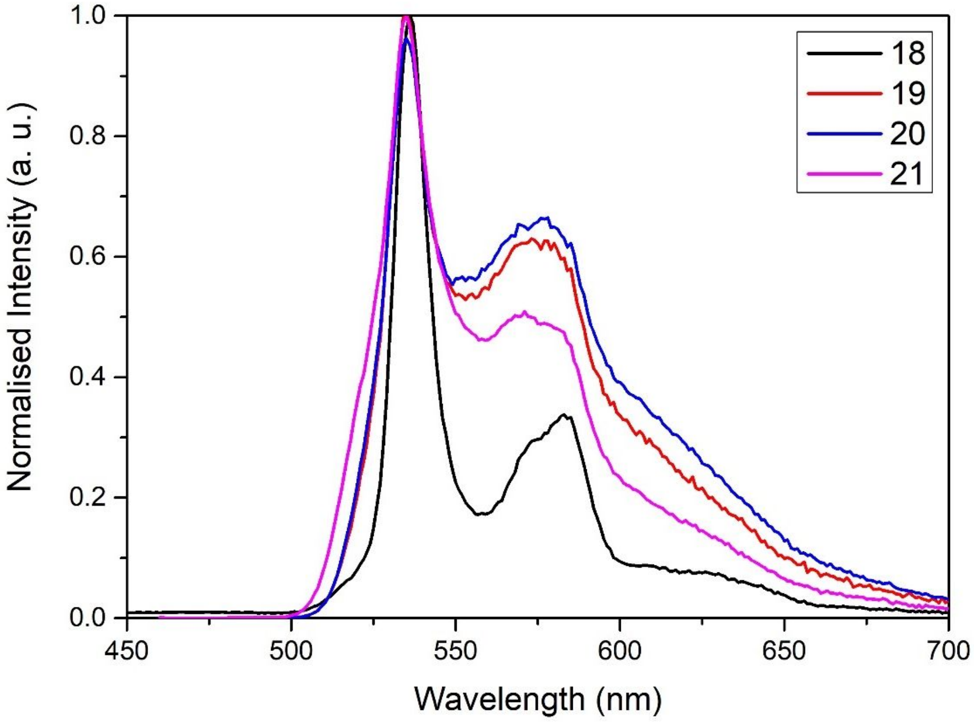

Figure S86. Emission of complexes **18**-**21** at 77K recorded in MeTHF.

Excitation dependence

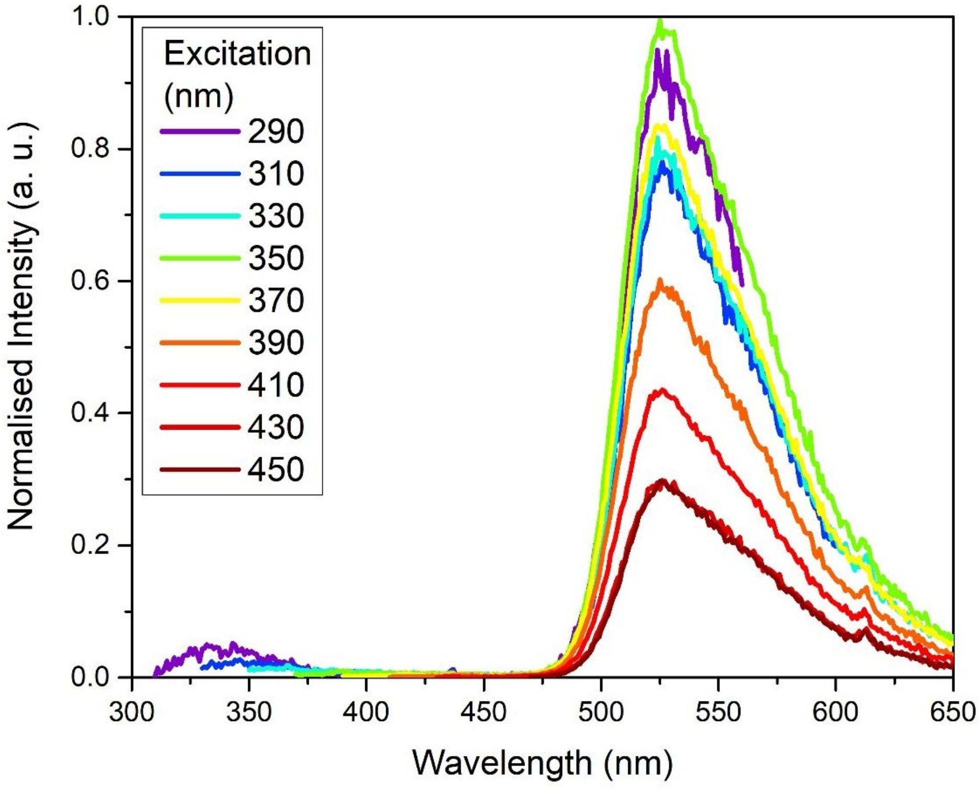

Figure S87. Excitation dependent emission for complex **1** recorded in MeTHF.

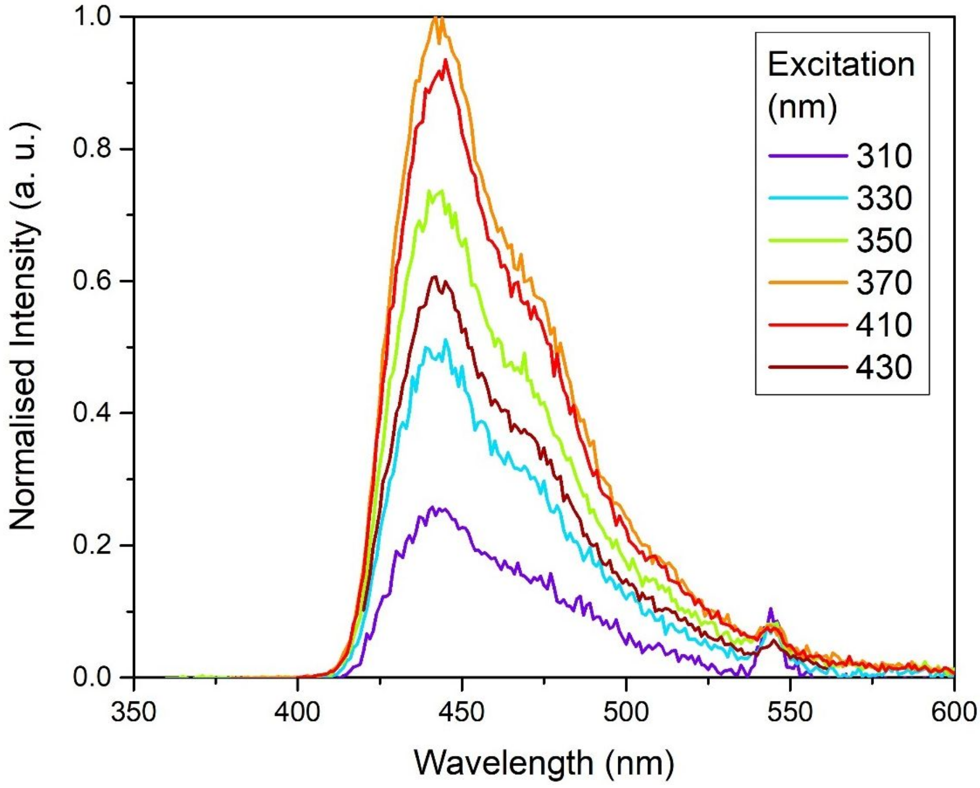

Figure S88. Excitation dependent emission for complex **2** recorded in MeTHF.

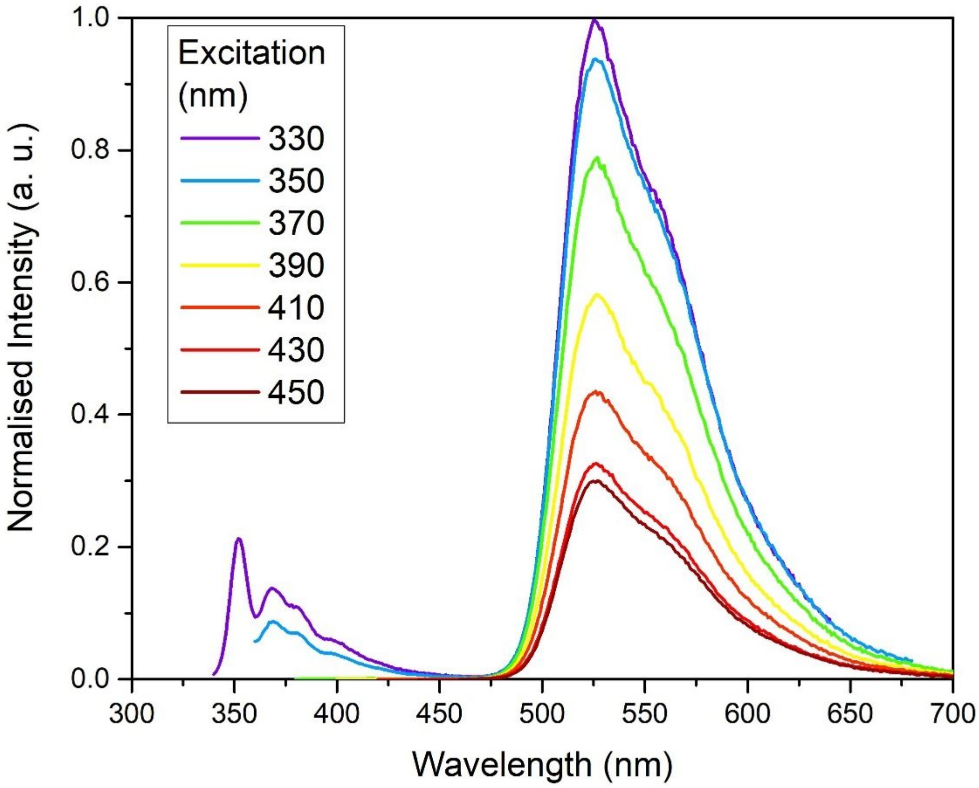

Figure S89. Excitation dependent emission for complex **3** recorded in MeTHF.

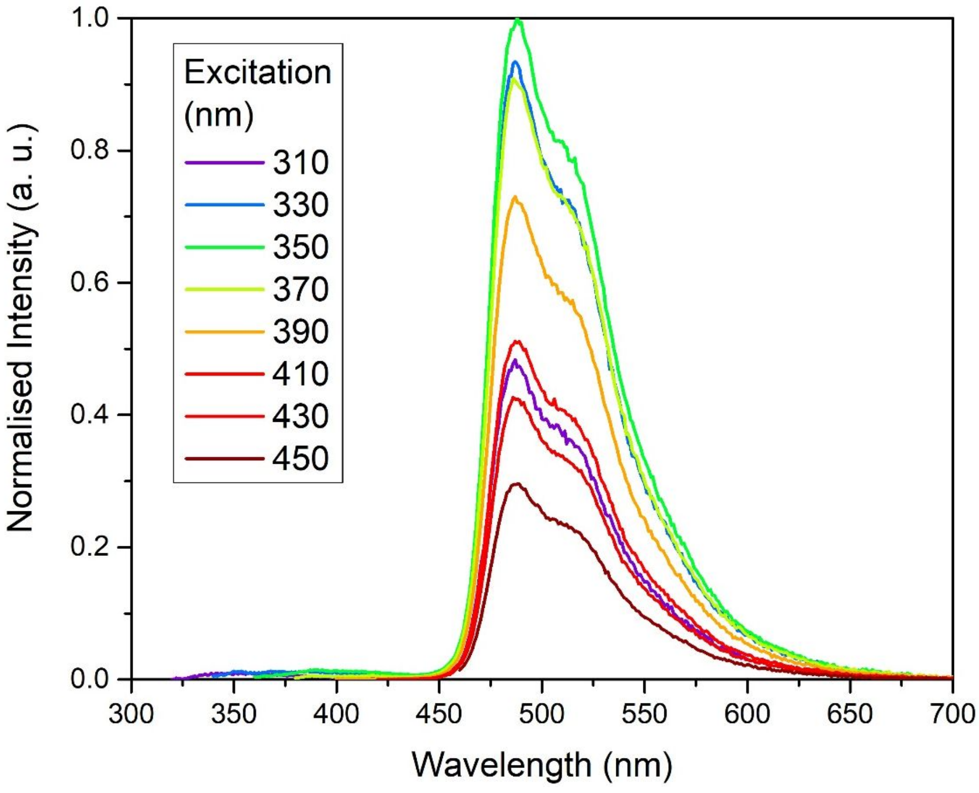

Figure S90. Excitation dependent emission for complex **4** recorded in MeTHF.

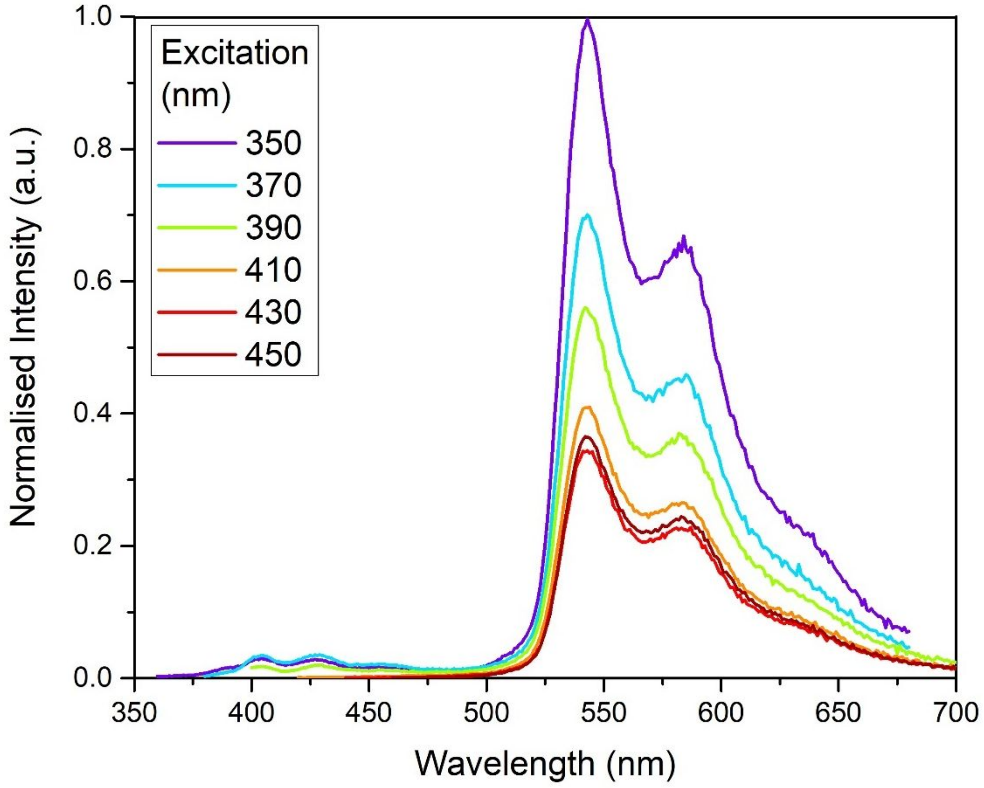

Figure S91. Excitation dependent emission for complex **6** recorded in MeTHF.

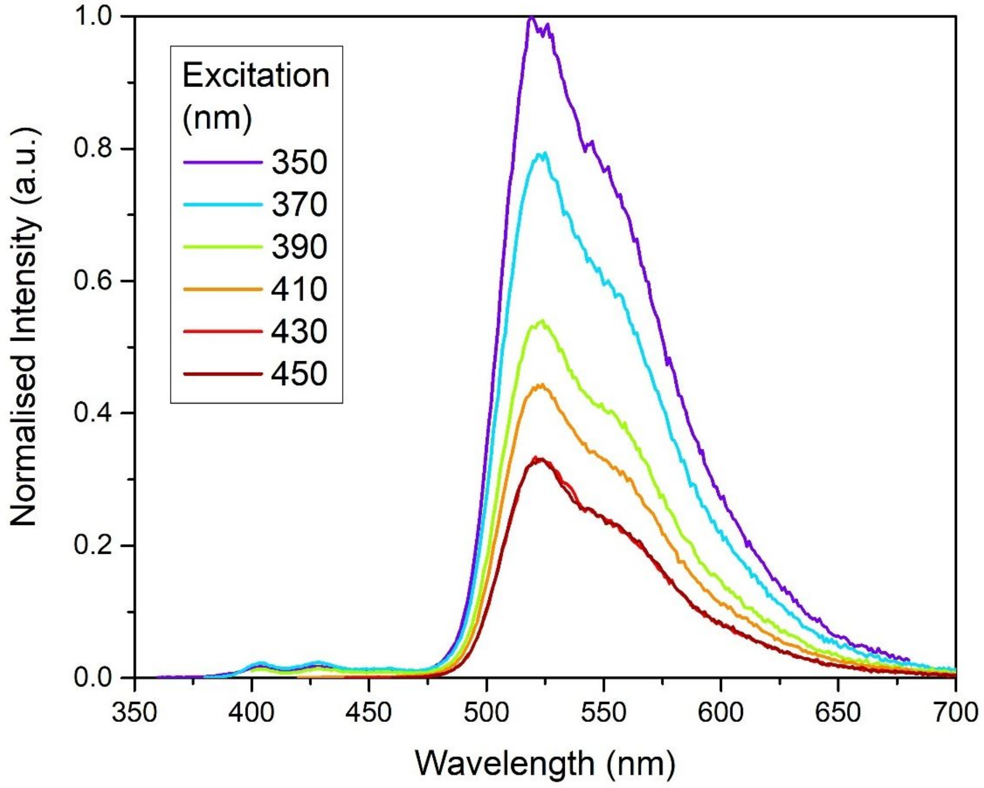

Figure S92. Excitation dependent emission for complex **7** recorded in MeTHF.

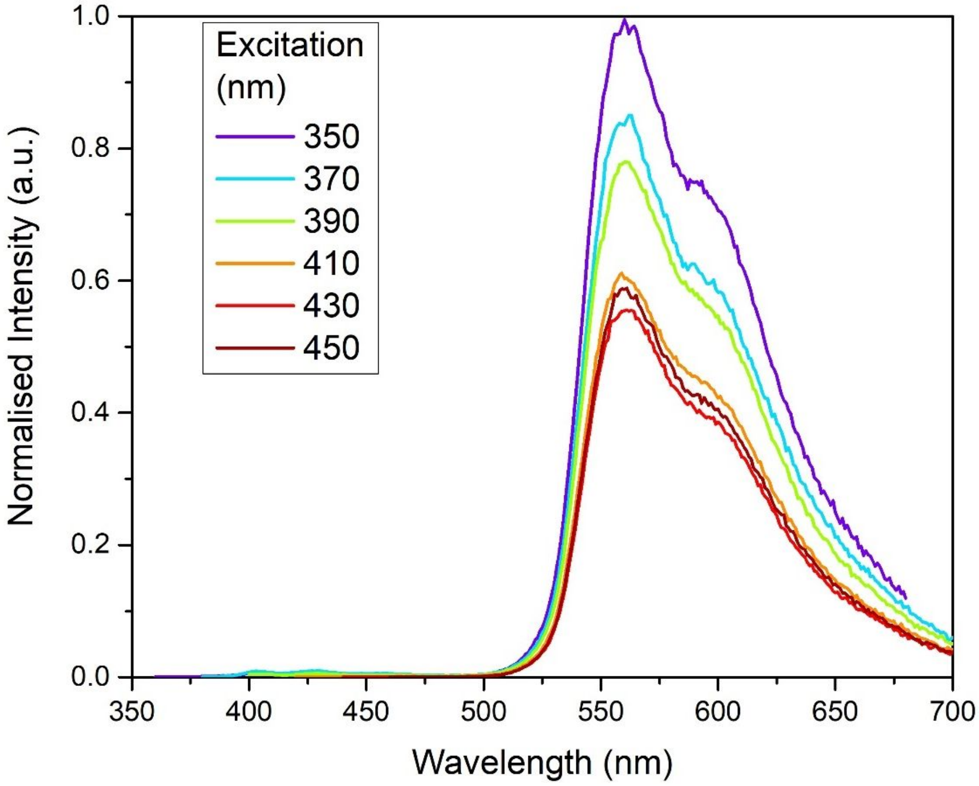

Figure S93. Excitation dependent emission for complex **8** recorded in MeTHF.

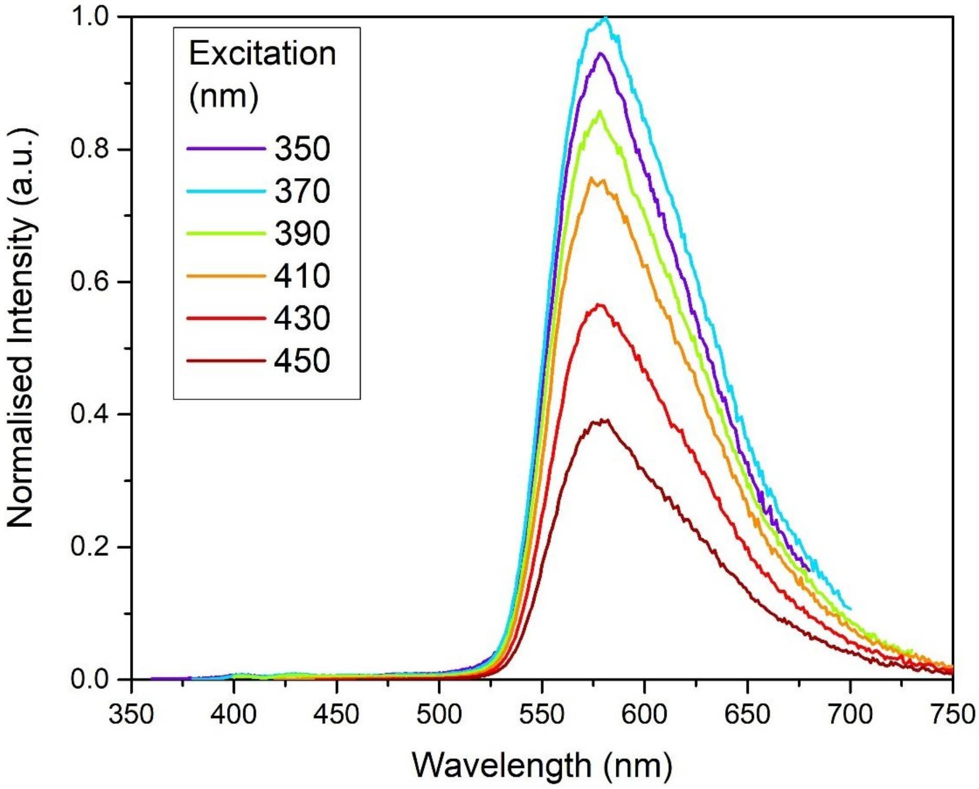

Figure S94. Excitation dependent emission for complex **9** recorded in MeTHF.

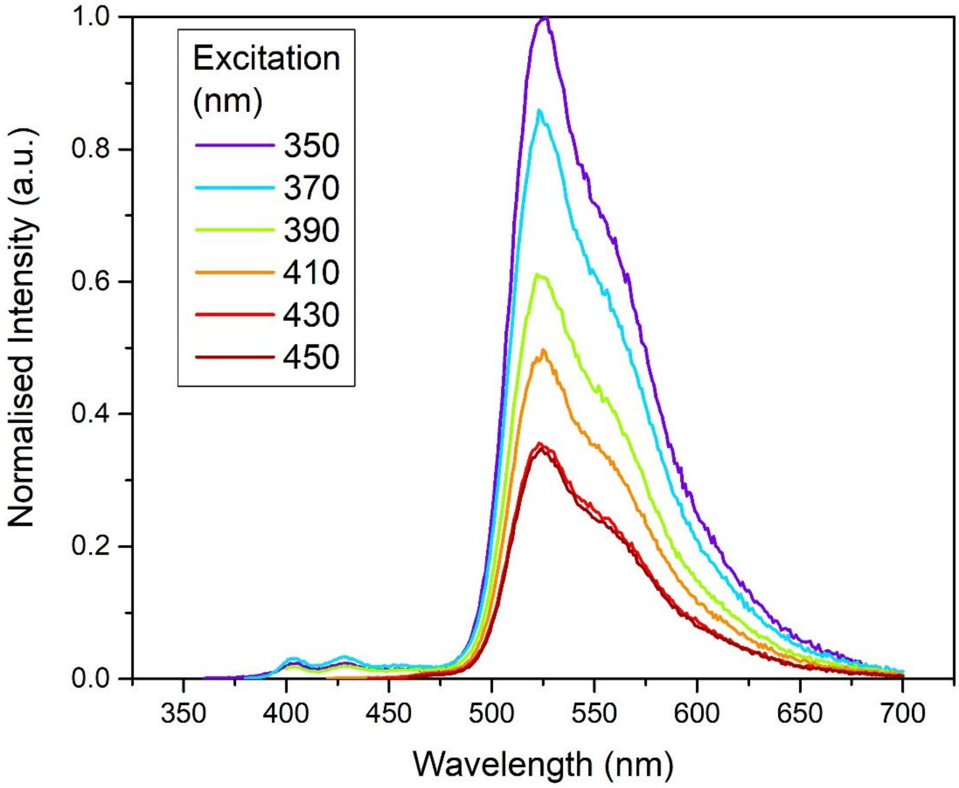

Figure S95. Excitation dependent emission for complex **10** recorded in MeTHF.

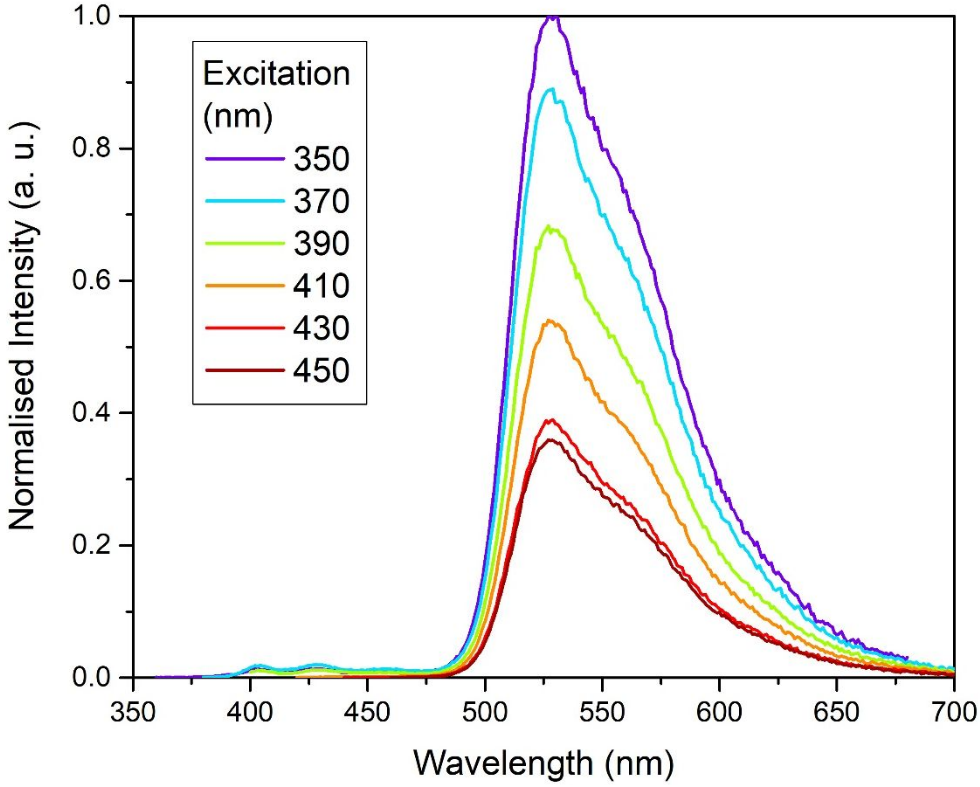

Figure S96. Excitation dependent emission for complex **12** recorded in MeTHF.

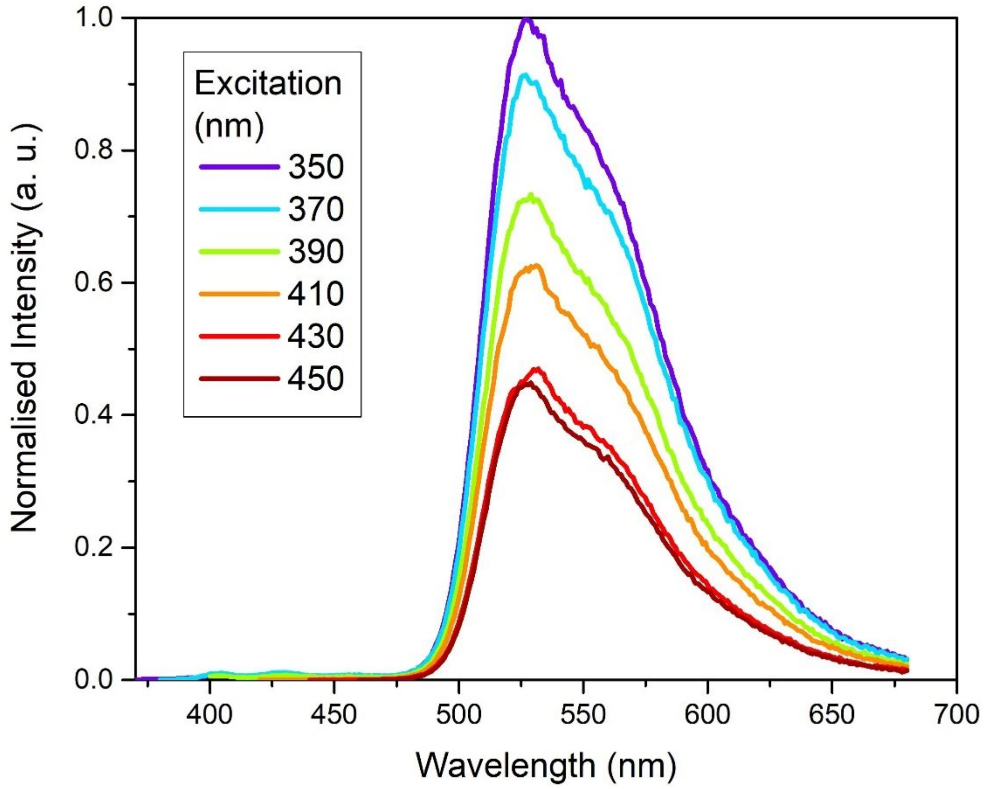

Figure S97. Excitation dependent emission for complex **13** recorded in MeTHF.

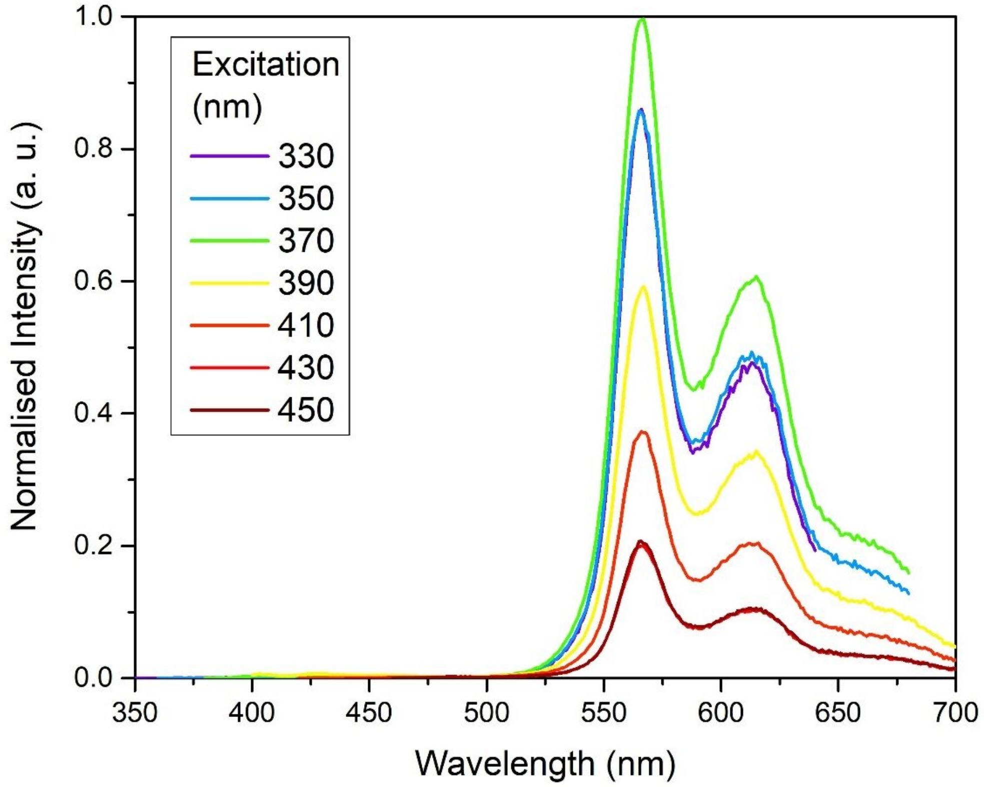

Figure S98. Excitation dependent emission for complex **14** recorded in MeTHF.

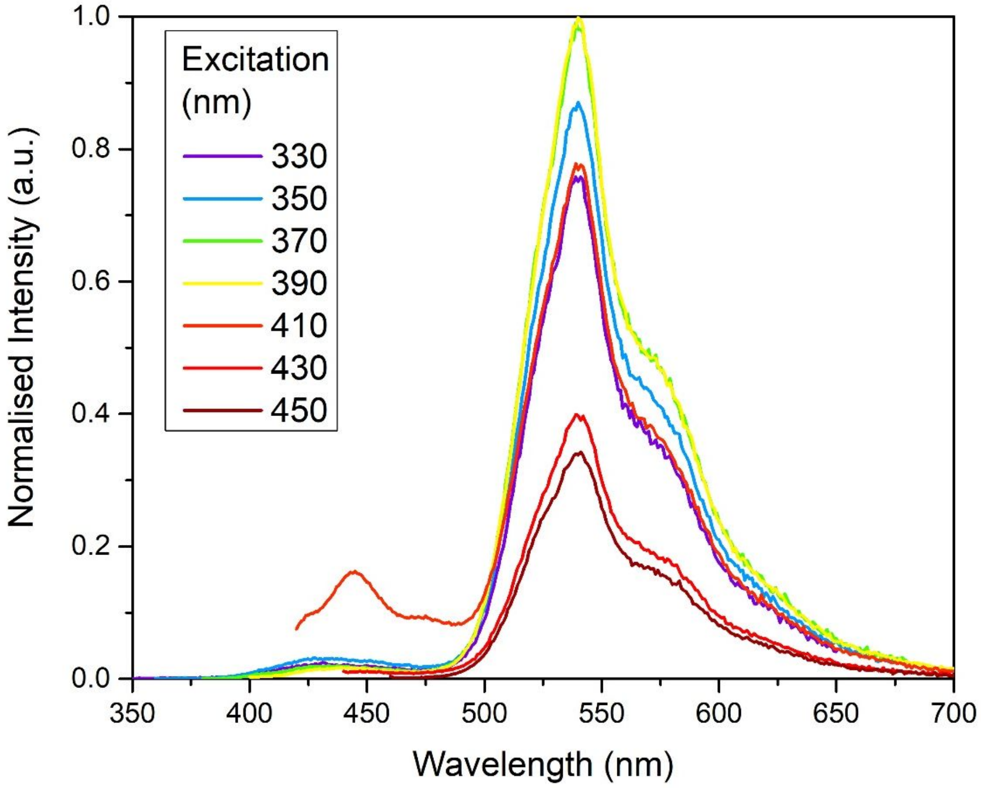

Figure S99. Excitation dependent emission for complex **15** recorded in MeTHF.

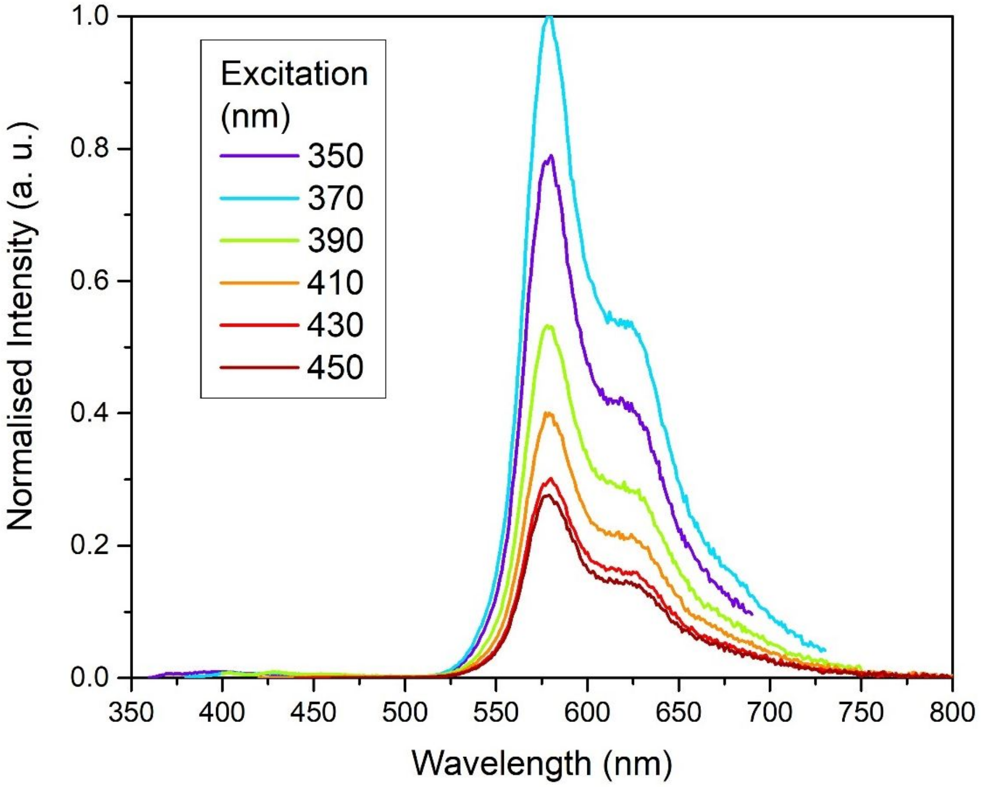

Figure S100. Excitation dependent emission for complex **16** recorded in MeTHF.

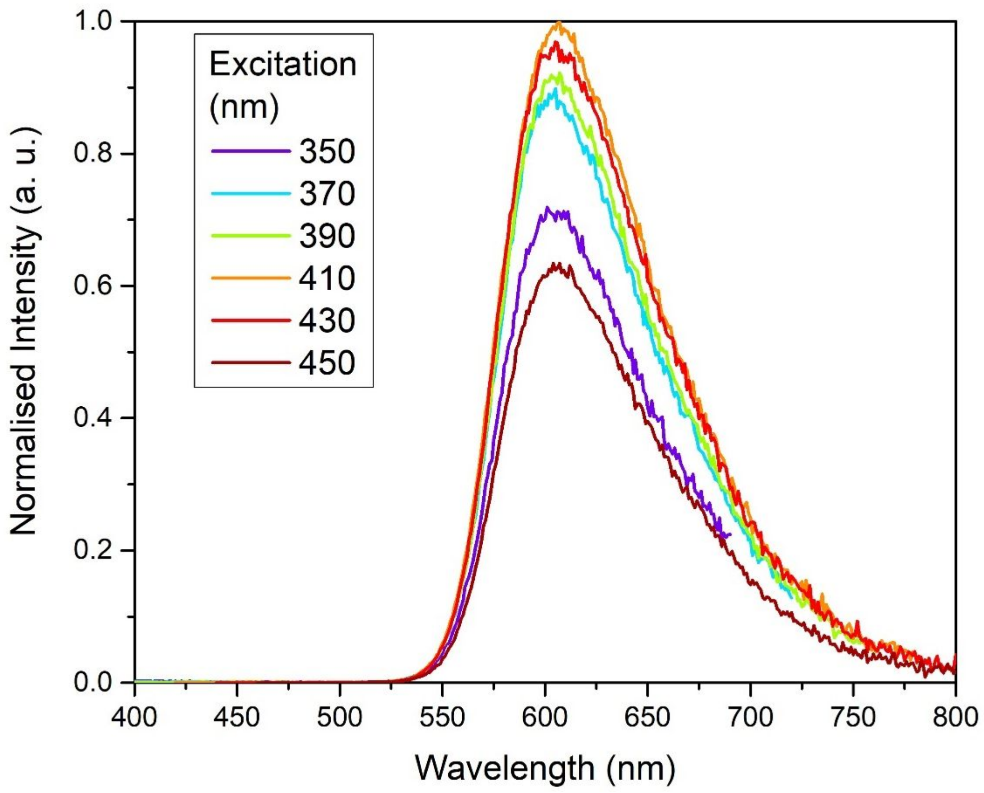

Figure S101. Excitation dependent emission for complex **17** recorded in MeTHF.

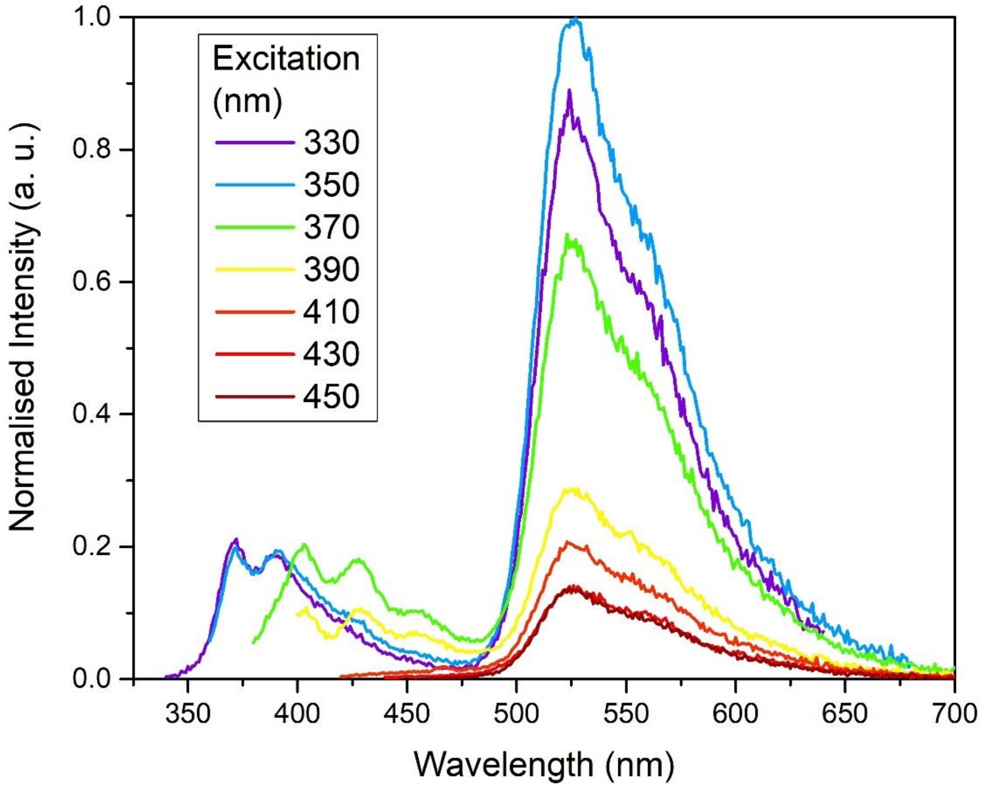

Figure S102. Excitation dependent emission for complex **18** recorded in MeTHF.

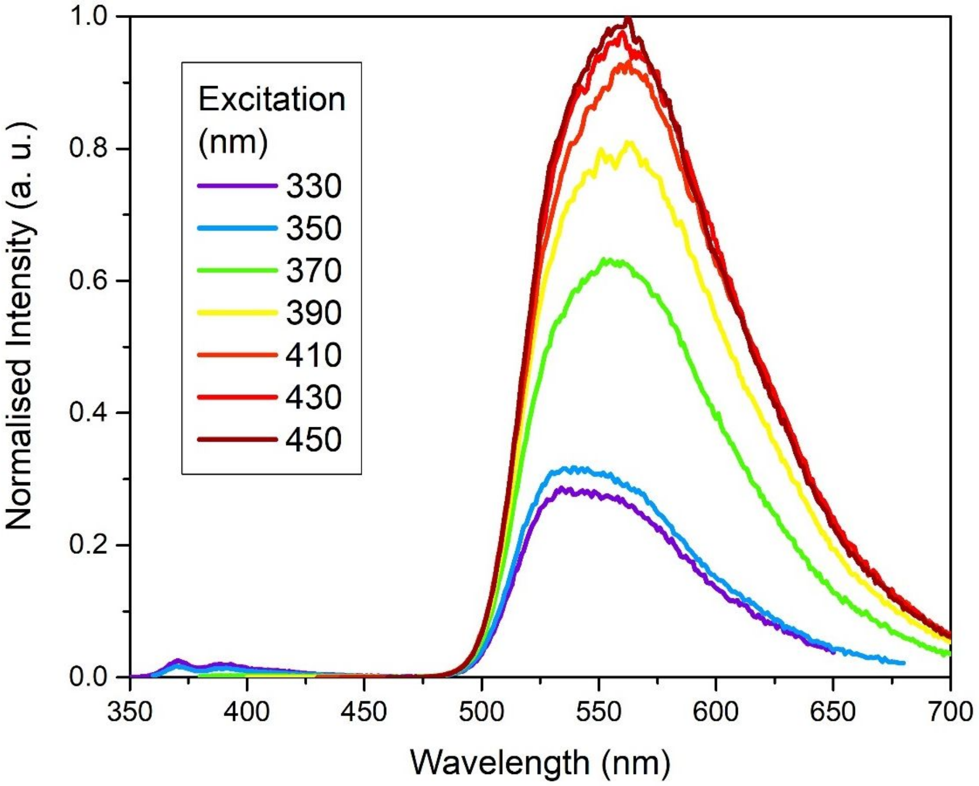

Figure S103. Excitation dependent emission for complex **19** recorded in MeTHF.

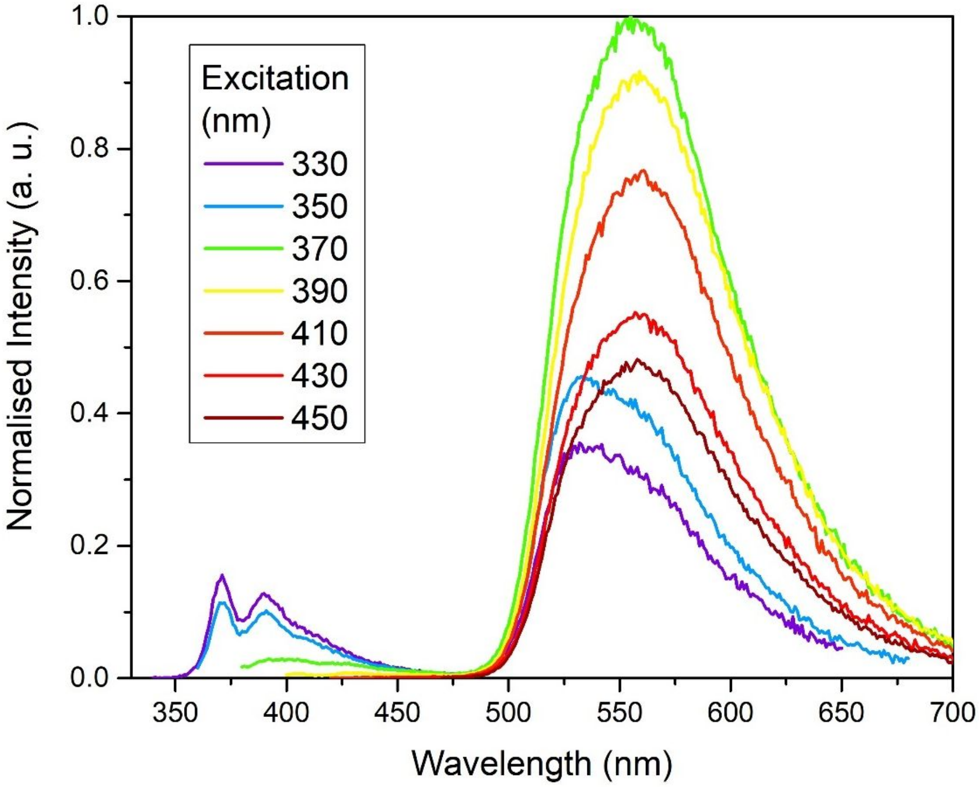

Figure S104. Excitation dependent emission for complex **20** recorded in MeTHF.

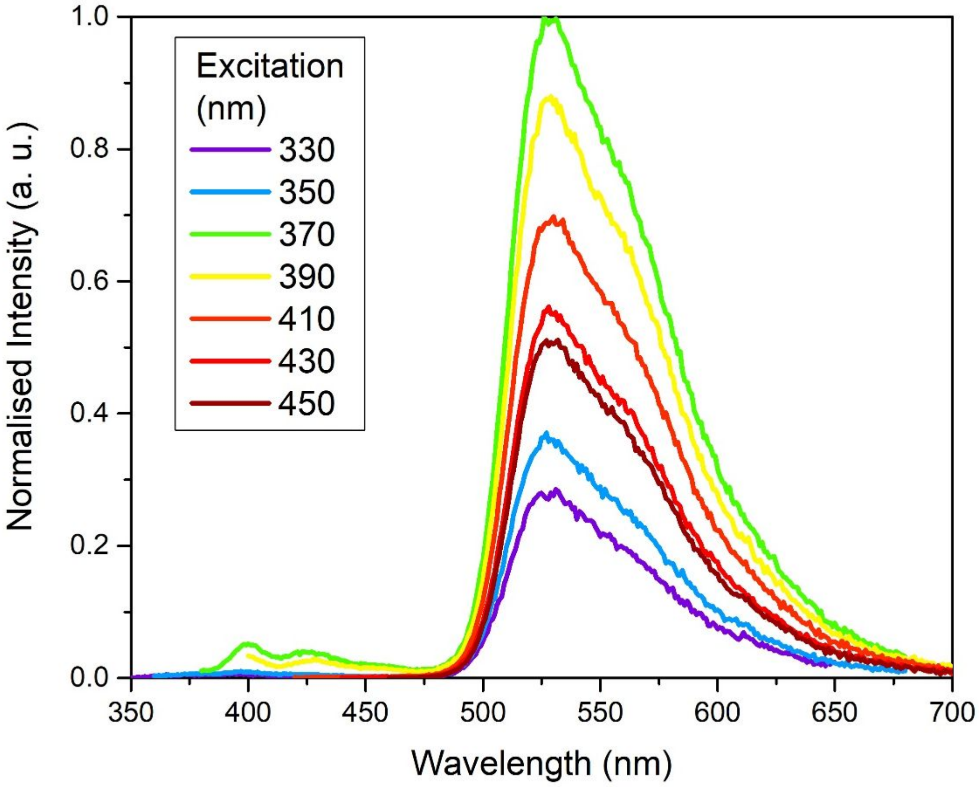

Figure S105. Excitation dependent emission for complex **21** recorded in MeTHF.

Lifetime trace

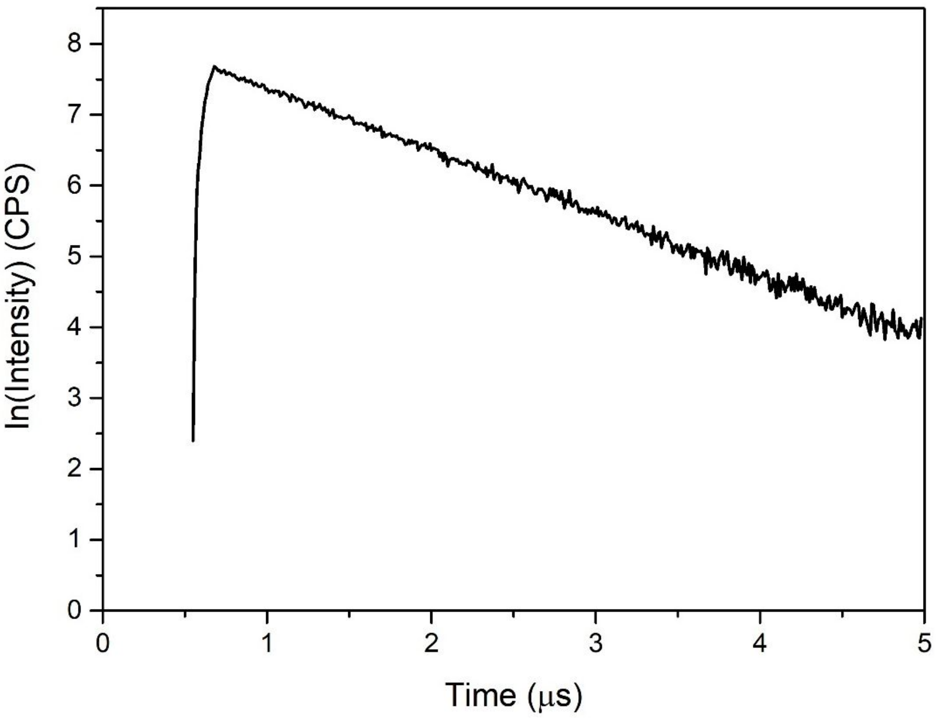

Figure S106. Emission lifetime trace of complex **1** excited by 405 nm.

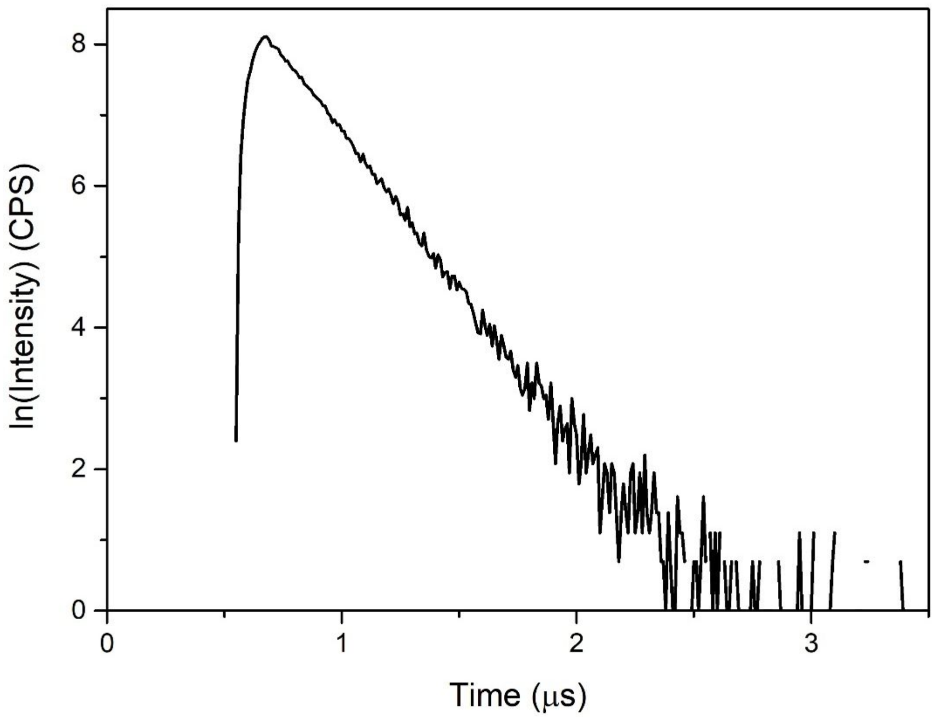

Figure S107. Emission lifetime trace of complex **2** excited by 405 nm.

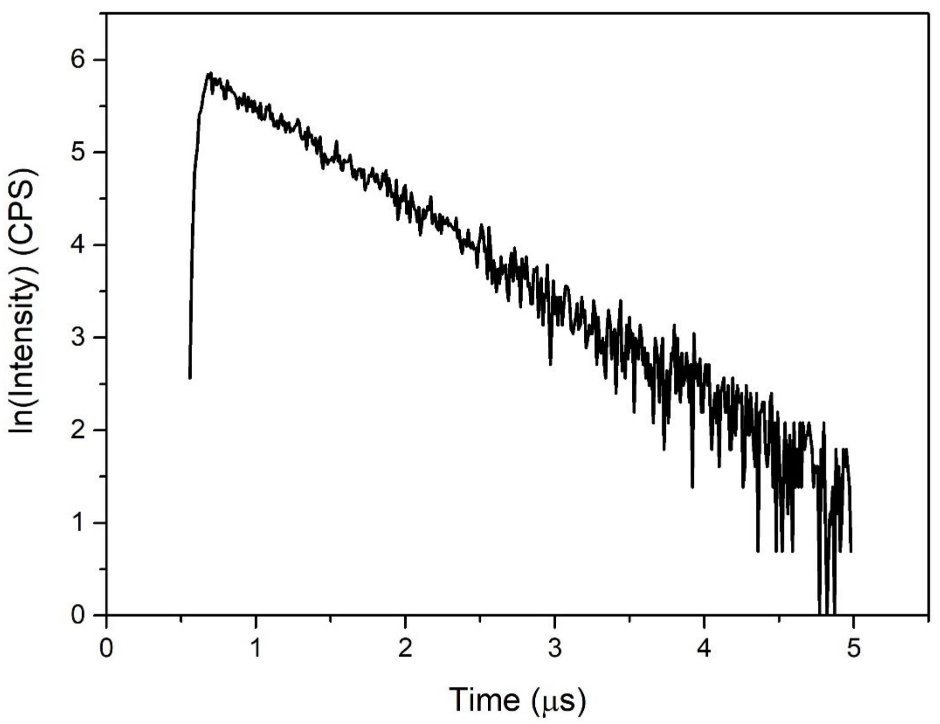

Figure S108. Emission lifetime trace of complex **3** excited by 405 nm.

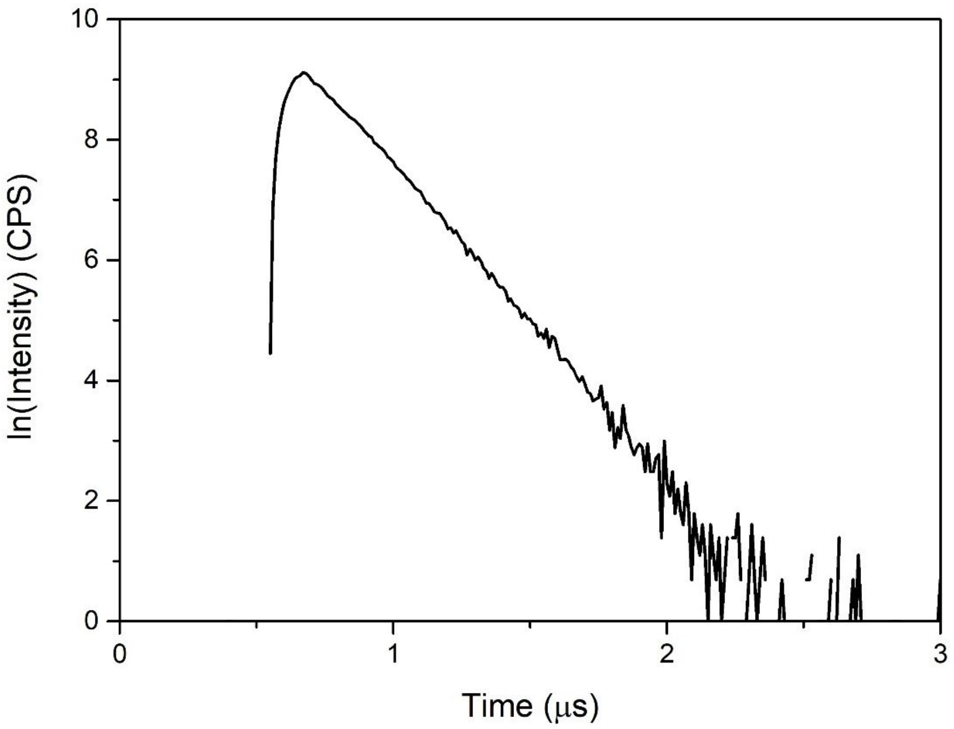

Figure S109. Emission lifetime trace of complex **4** excited by 405 nm.

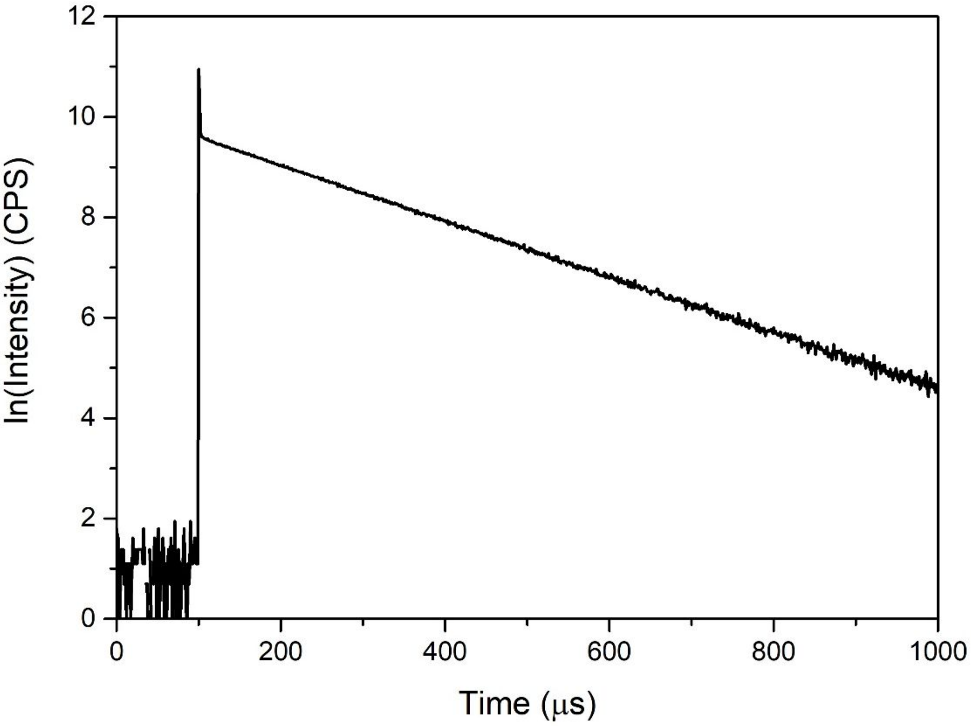

Figure S110. Emission lifetime trace of complex **5** excited by 337 nm.

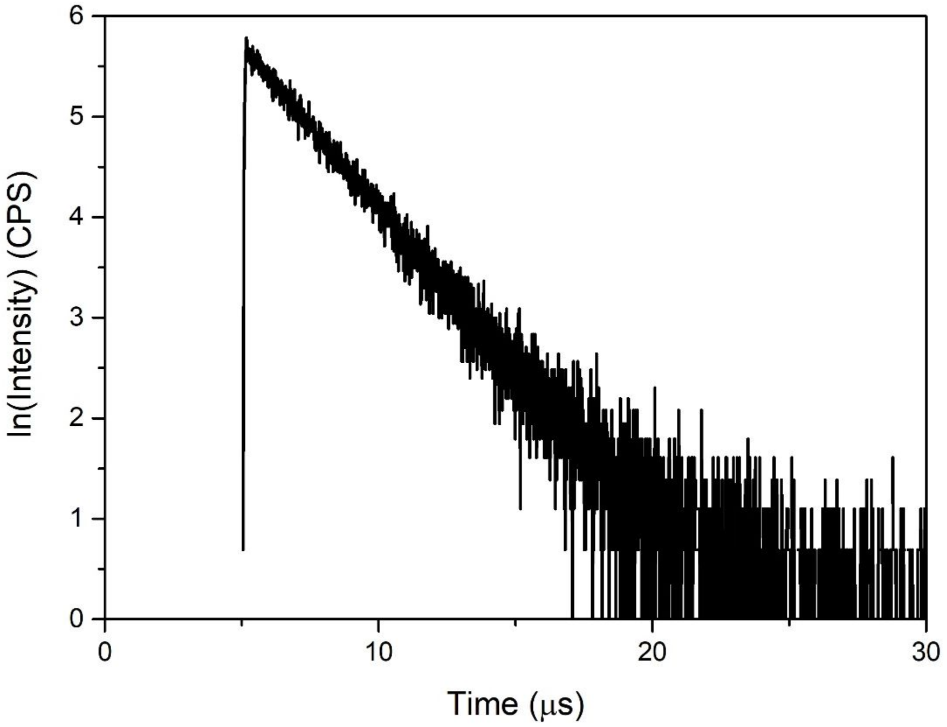

Figure S111. Emission lifetime trace of complex **6** excited by 405 nm.

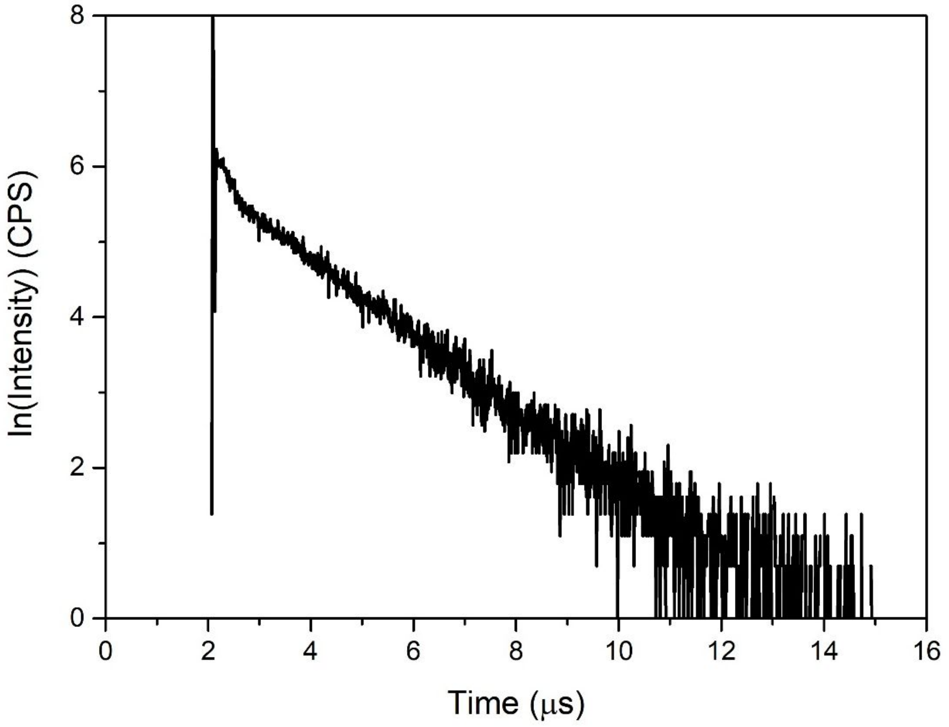

Figure S112. Emission lifetime trace of complex **7** excited by 405 nm.

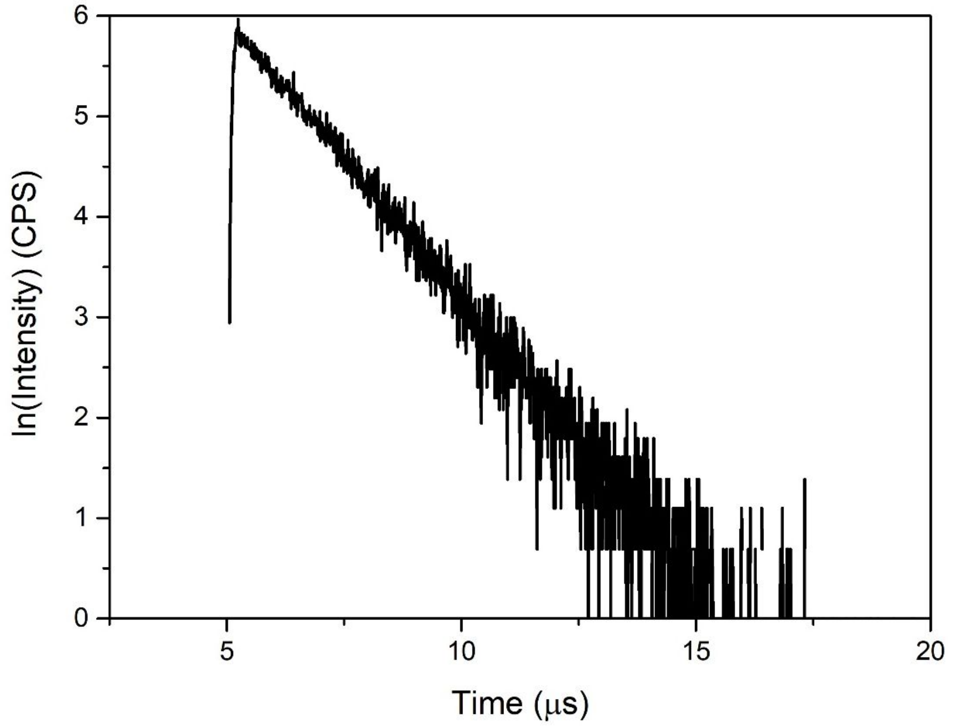

Figure S113. Emission lifetime trace of complex **8** excited by 405 nm.

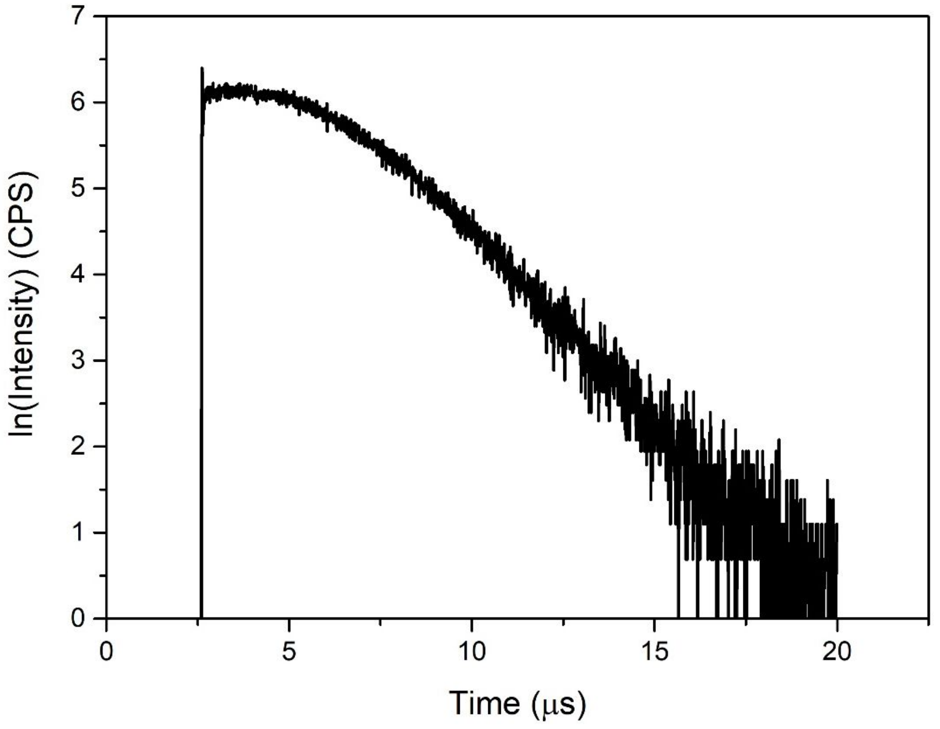

Figure S114. Emission lifetime trace of complex **10** excited by 405 nm.

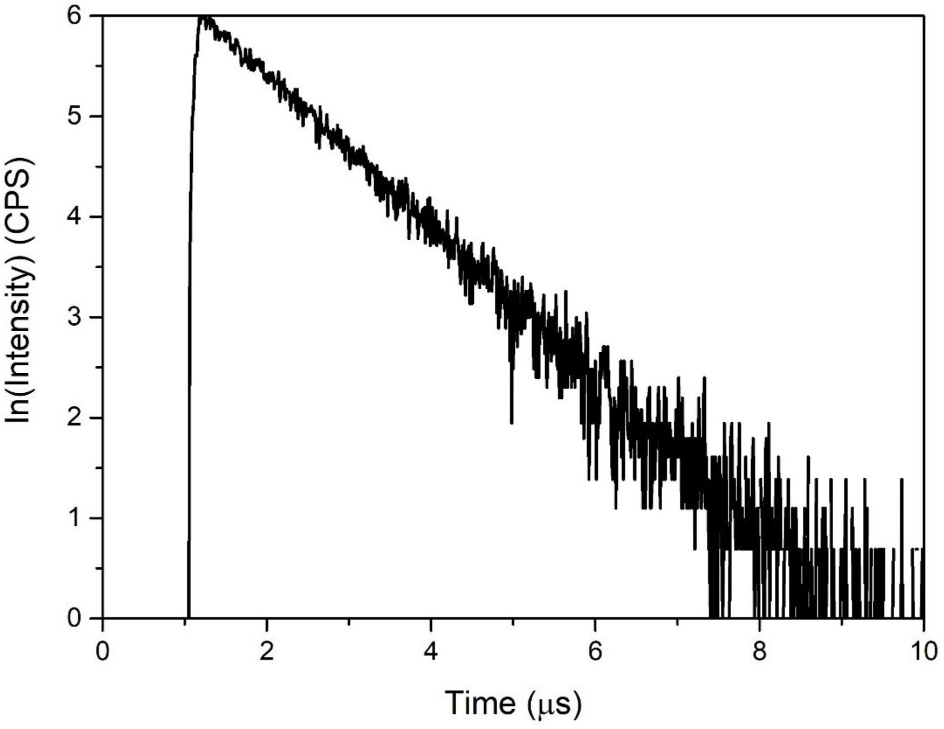

Figure S115. Emission lifetime trace of complex **13** excited by 405 nm.

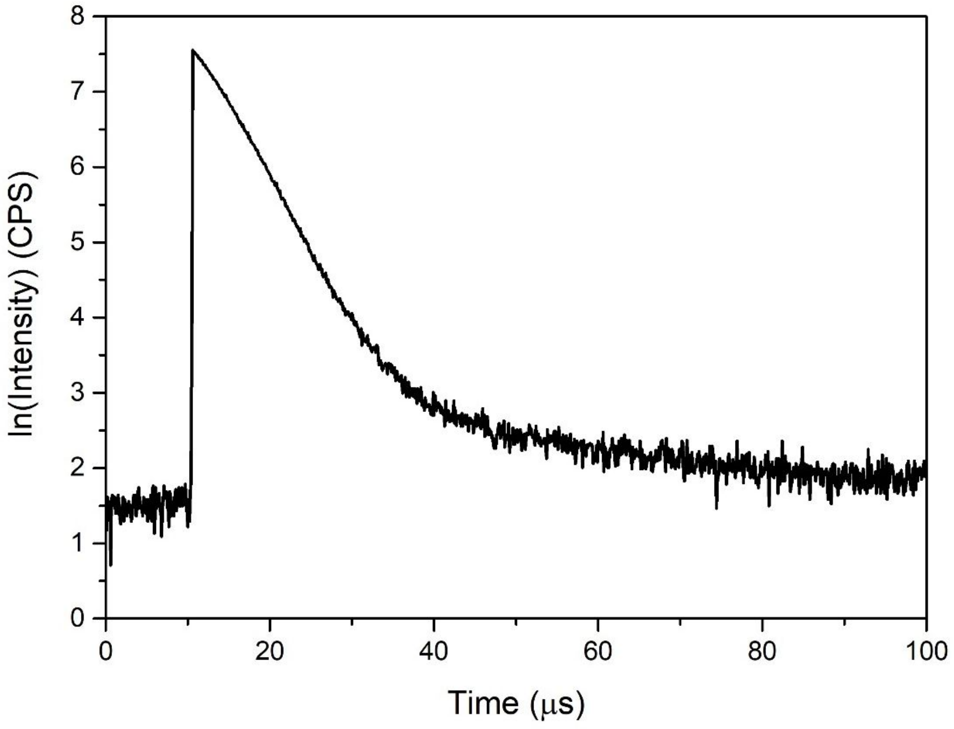

Figure S116. Emission lifetime trace of complex **14** excited by 405 nm.

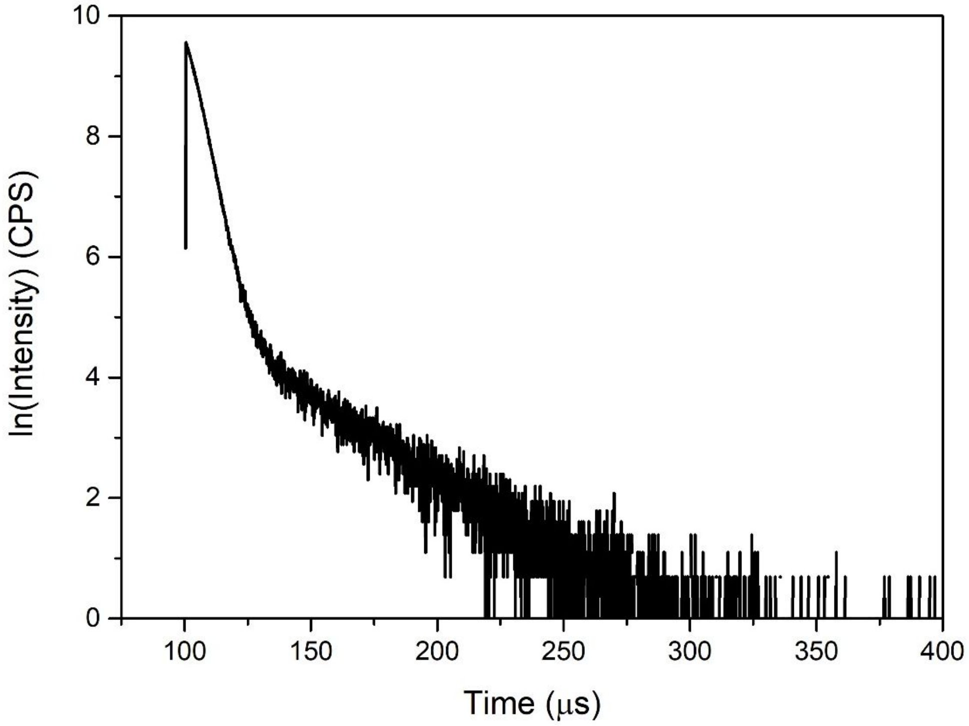

Figure S117. Emission lifetime trace of complex **14** excited by 337 nm.

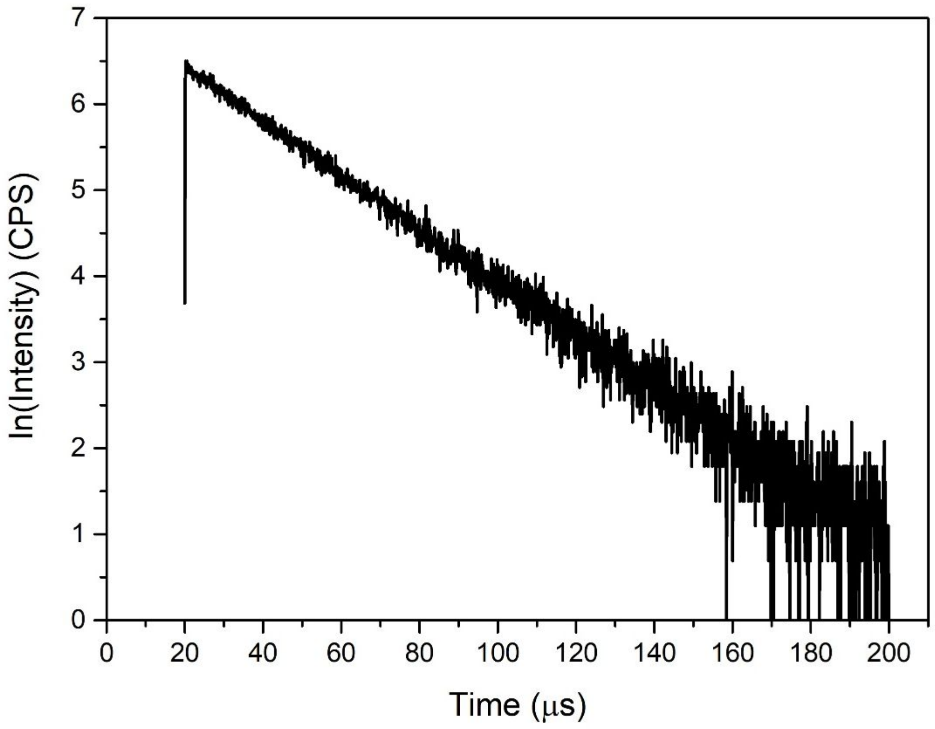

Figure S118. Emission lifetime trace of complex **15** excited by 405 nm.

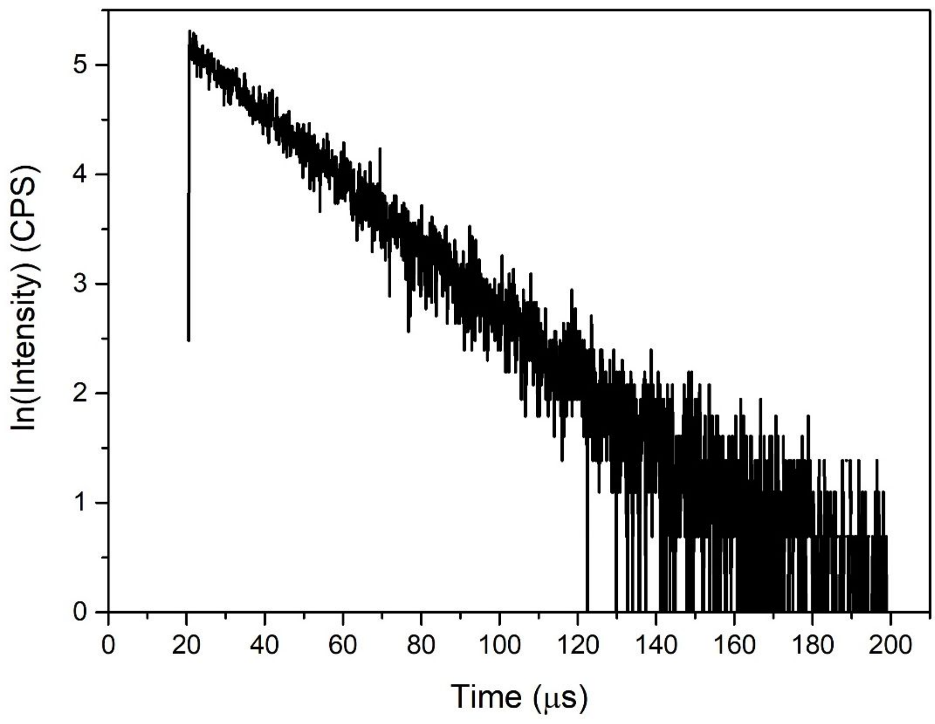

Figure S119. Emission lifetime trace of complex **15** excited by 337 nm

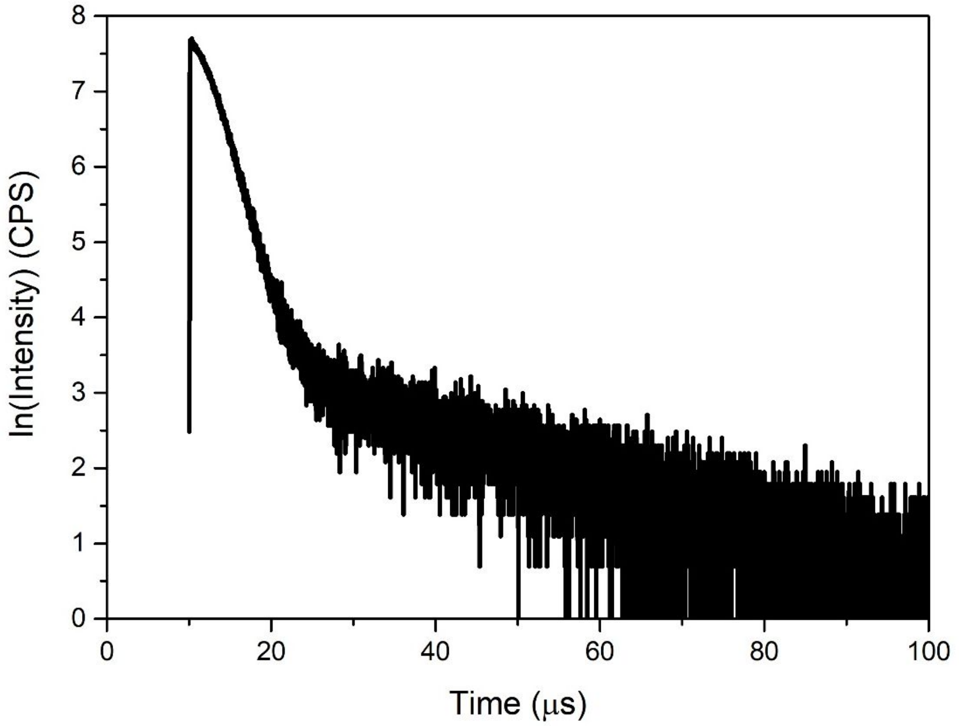

Figure S120. Emission lifetime trace of complex **16** excited by 405 nm.

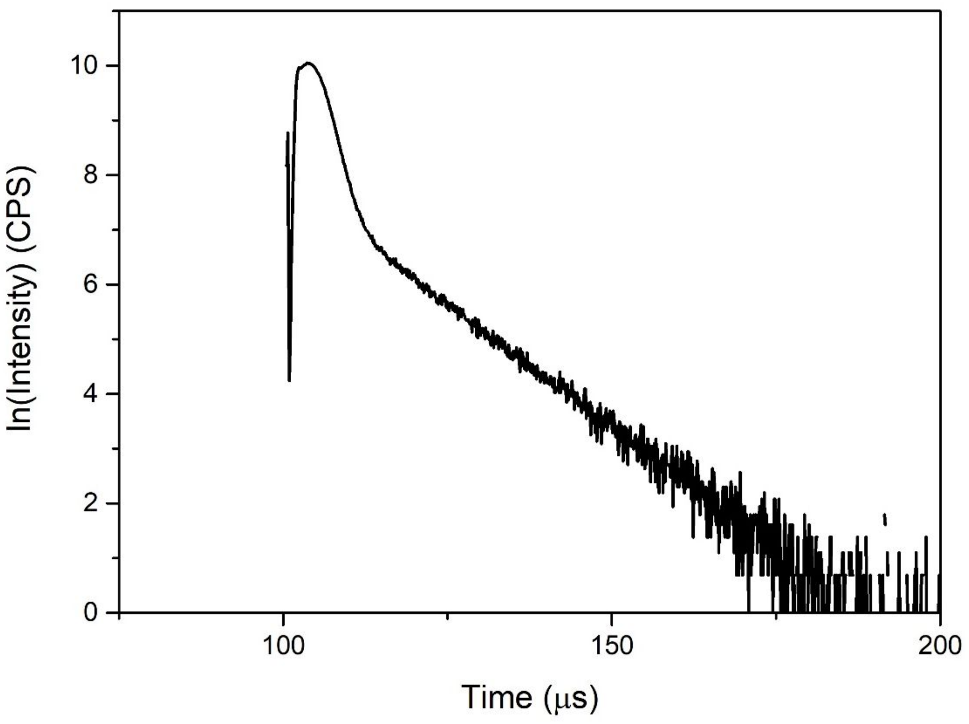

Figure S121. Emission lifetime trace of complex **16** excited by 337 nm.

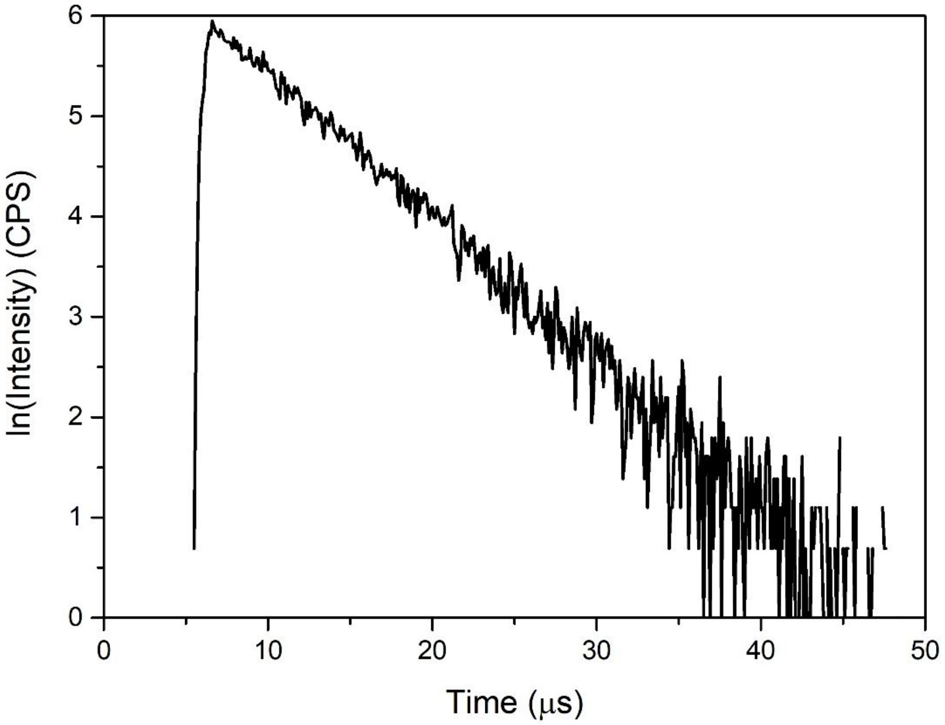

Figure S122. Emission lifetime trace of complex **17** excited by 337 nm.

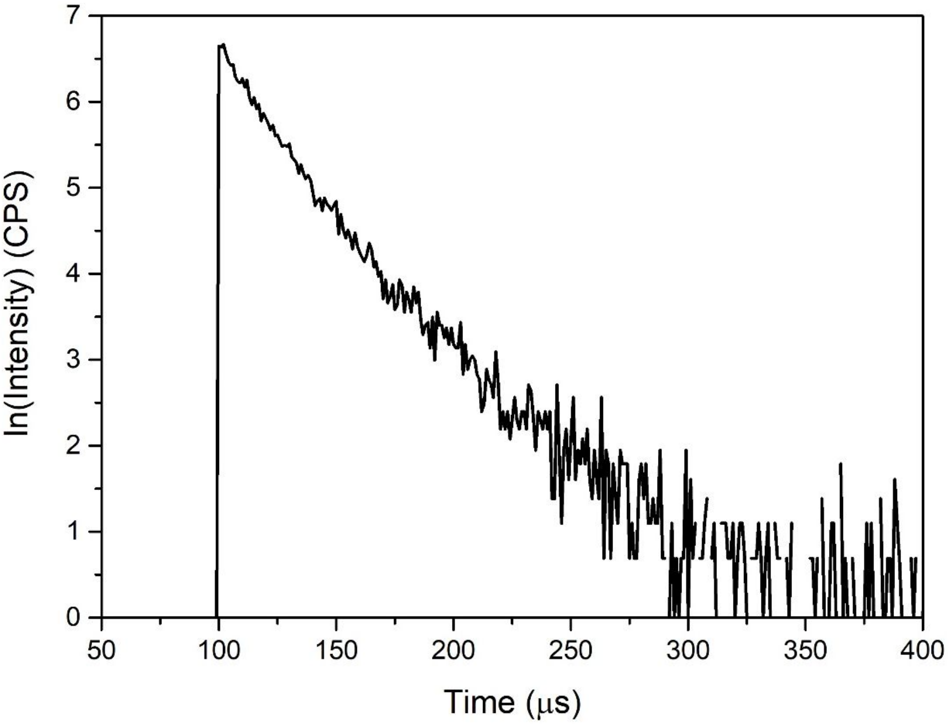

Figure S123. Emission lifetime trace of complex **18** excited by 405 nm.

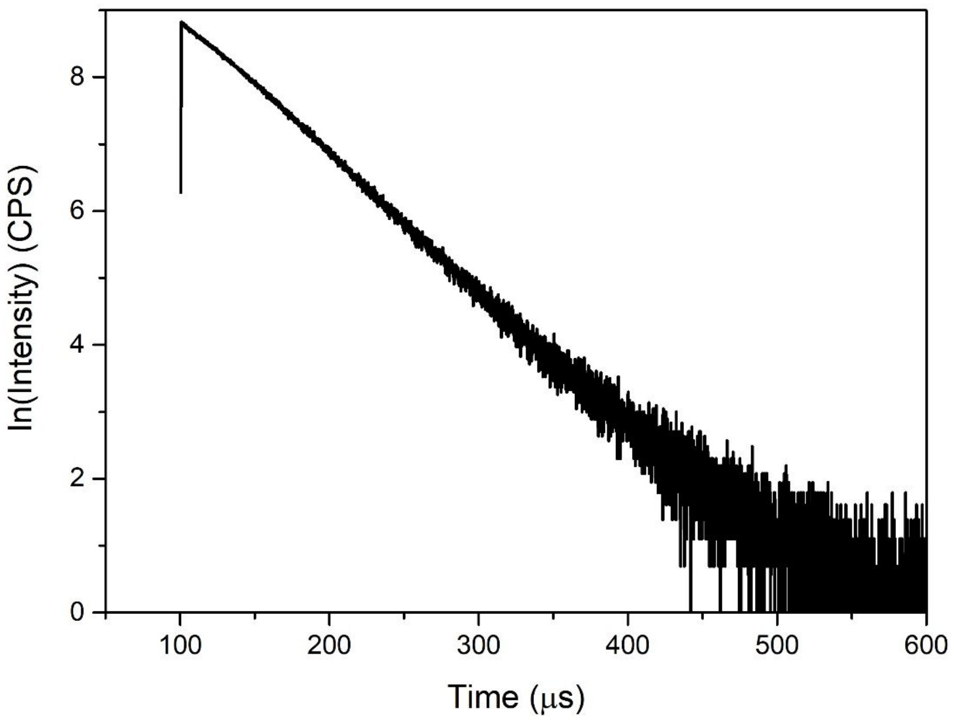

Figure S124. Emission lifetime trace of complex **18** excited by 337 nm.

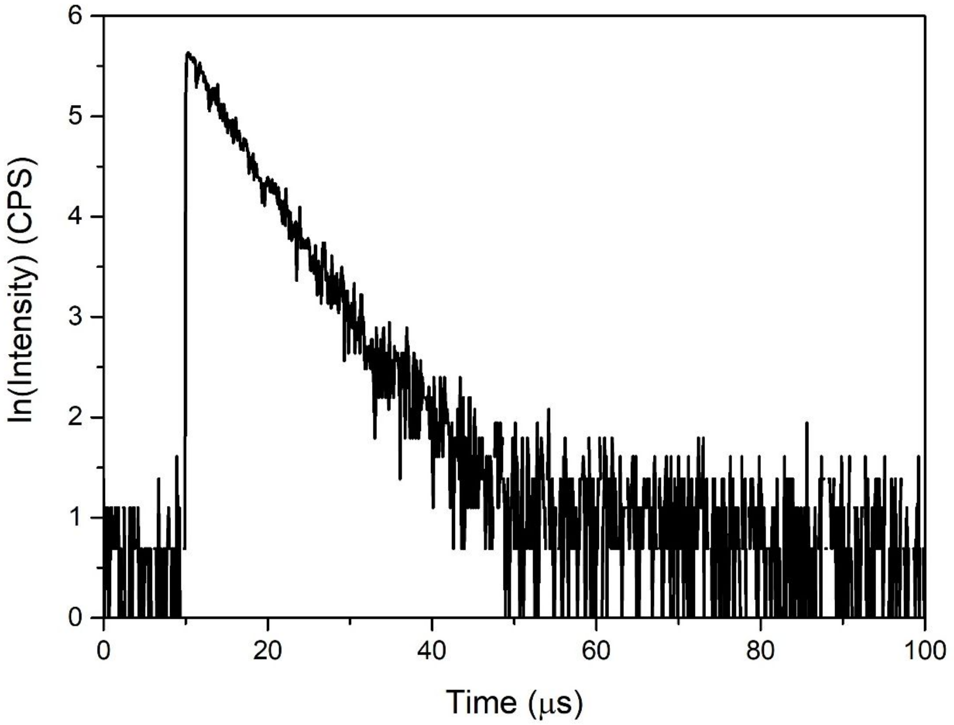

Figure S125. Emission lifetime trace of complex **19** excited by 405 nm.

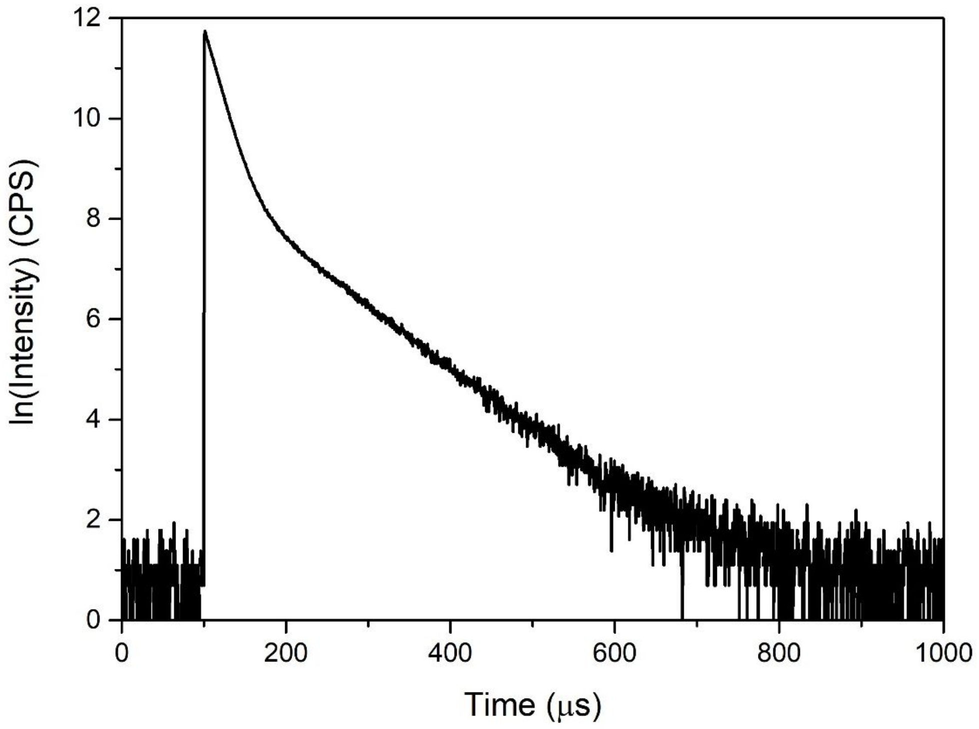

Figure S126. Emission lifetime trace of complex **19** excited by 337 nm.

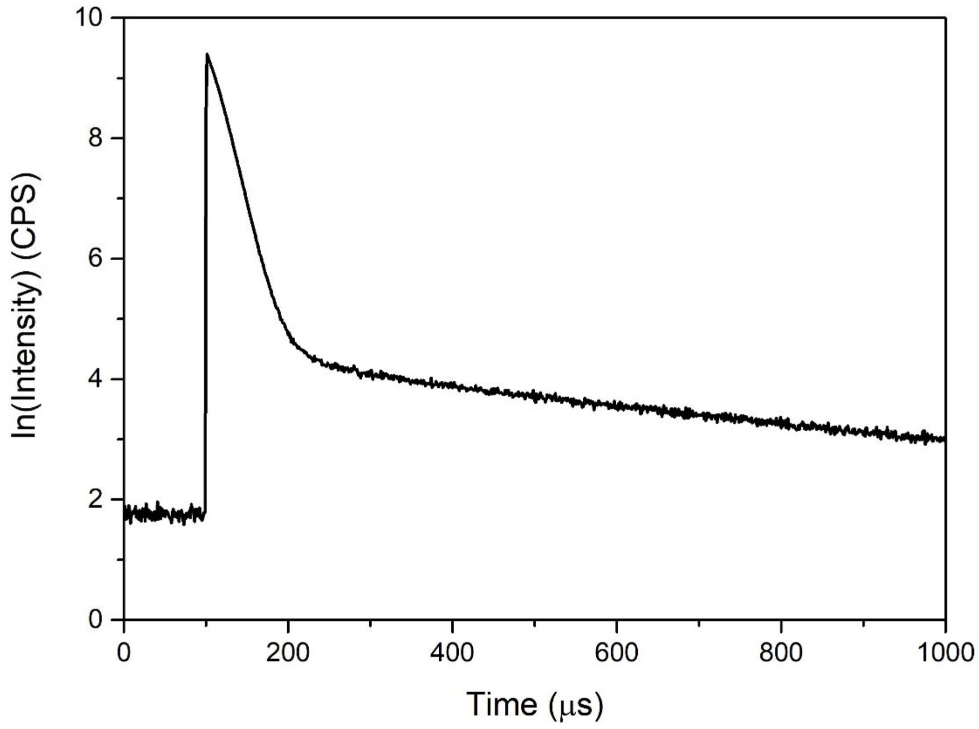

Figure S127. Emission lifetime trace of complex **20** excited by 337 nm.

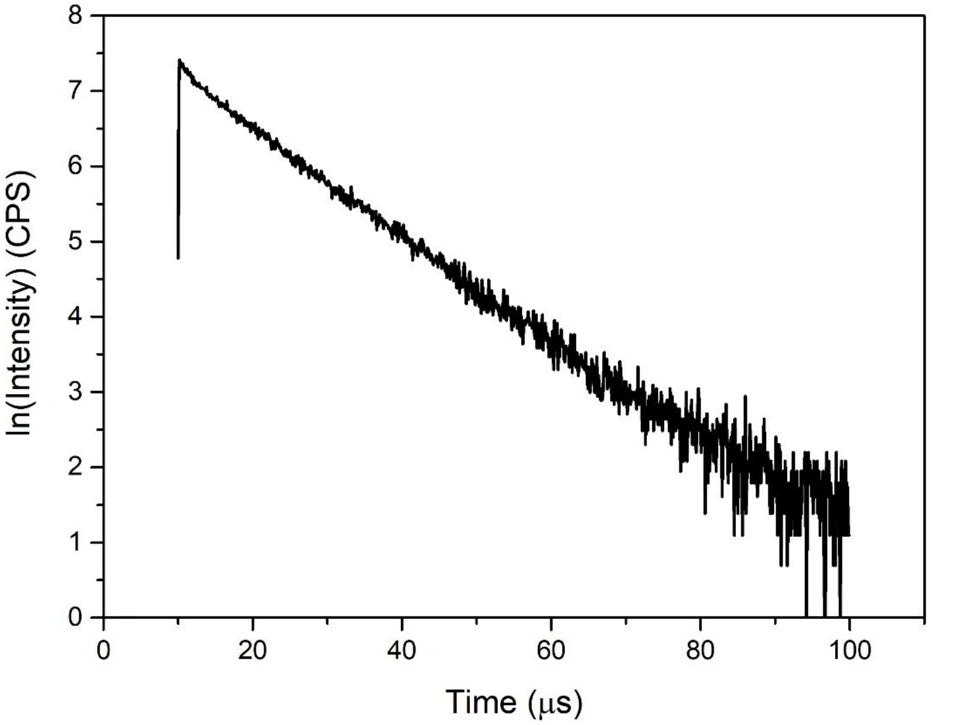

Figure S128. Emission lifetime trace of complex **21** excited by 405 nm.

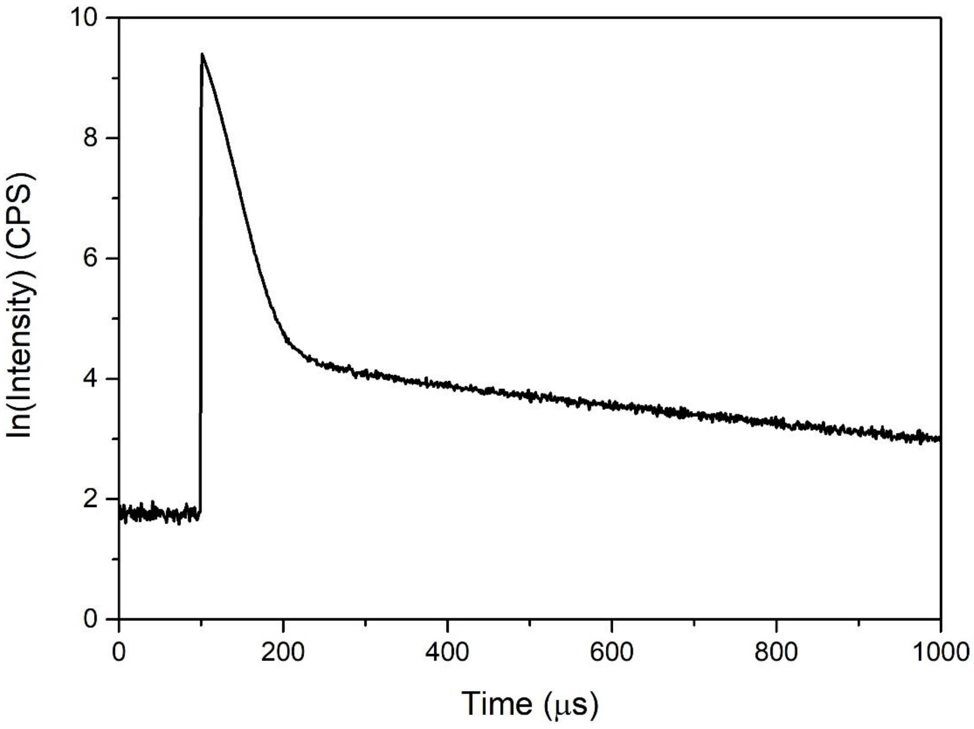

Figure S129. Emission lifetime trace of complex **21** excited by 337 nm.

OPE3 substituted complex excitation

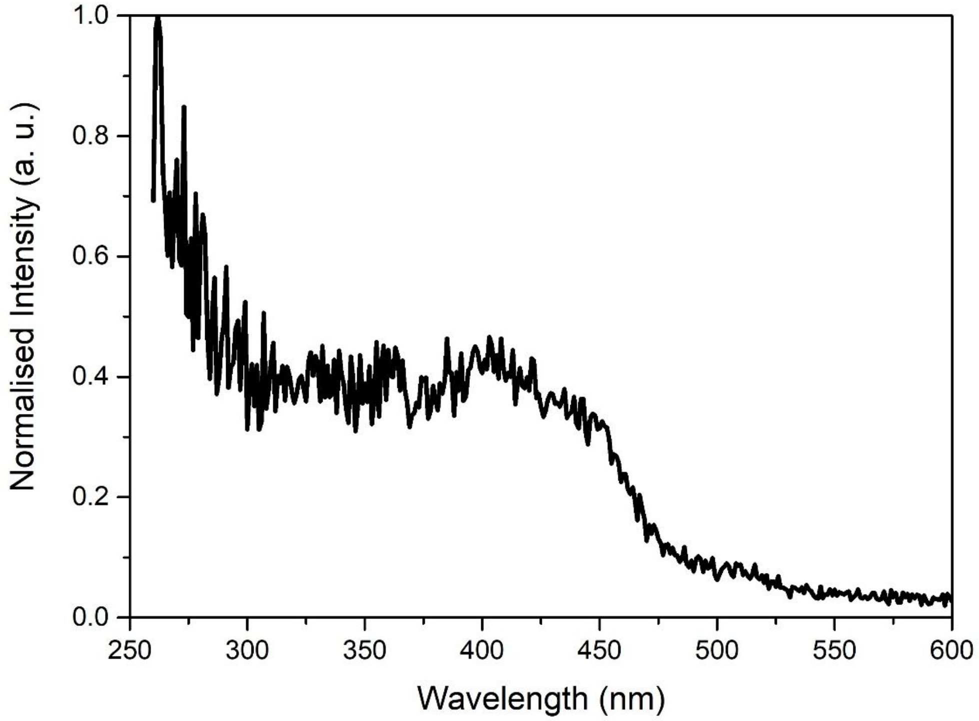

Figure S130. Excitation spectrum of complex **5**.

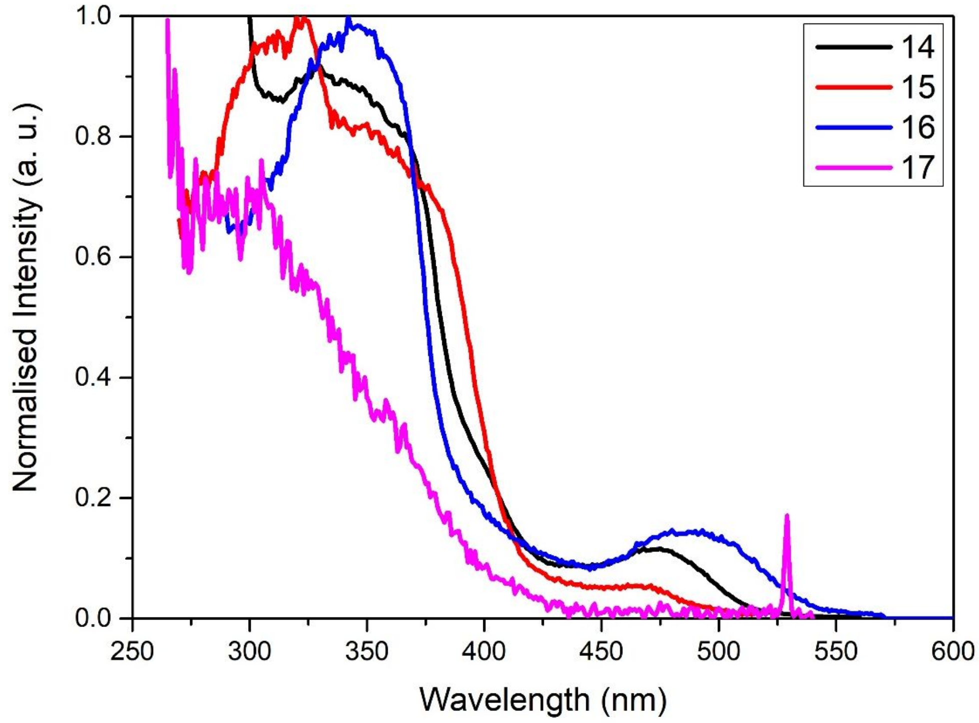

Figure S131. Excitation spectra of complexes **14-17**.

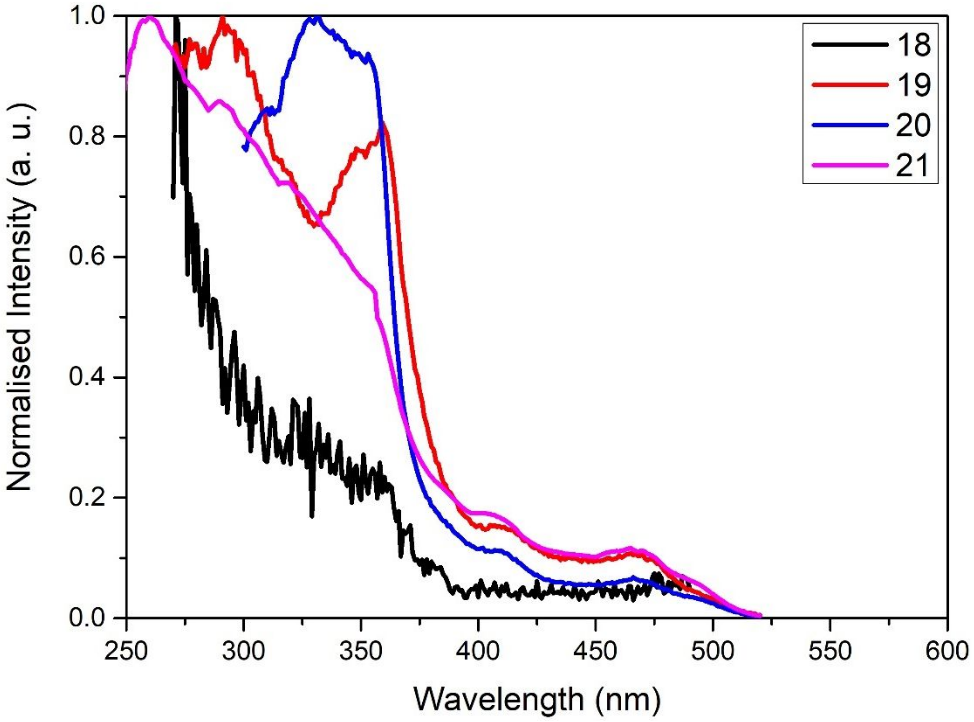

Figure S132. Excitation spectra of complexes **18**-**21**.

Time resolved spectra

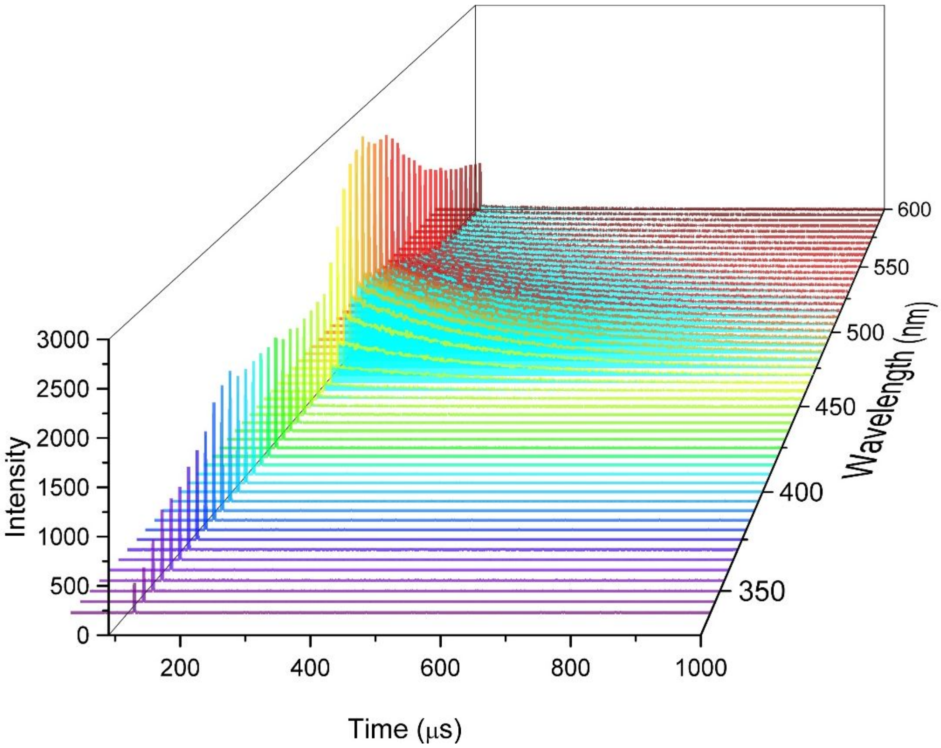

Figure S133. Time resolved emission spectra of **5** recorded in DCM excited as 337 nm.

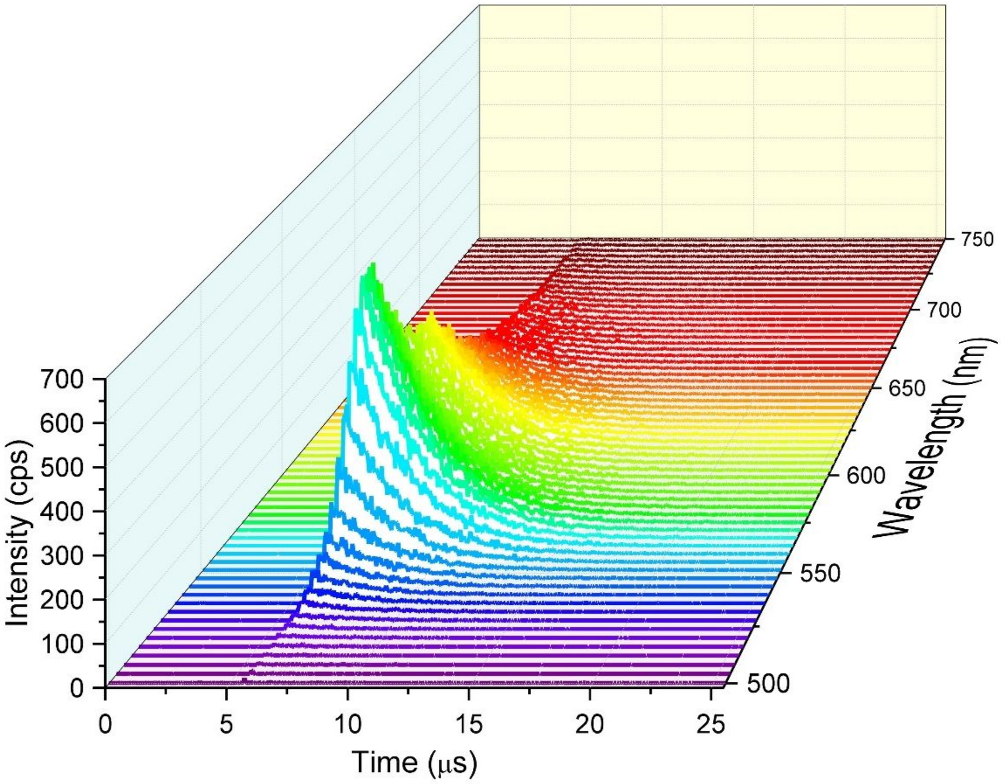

Figure 134. Time resolved emission spectra of **16** recorded in DCM excited as 337 nm.

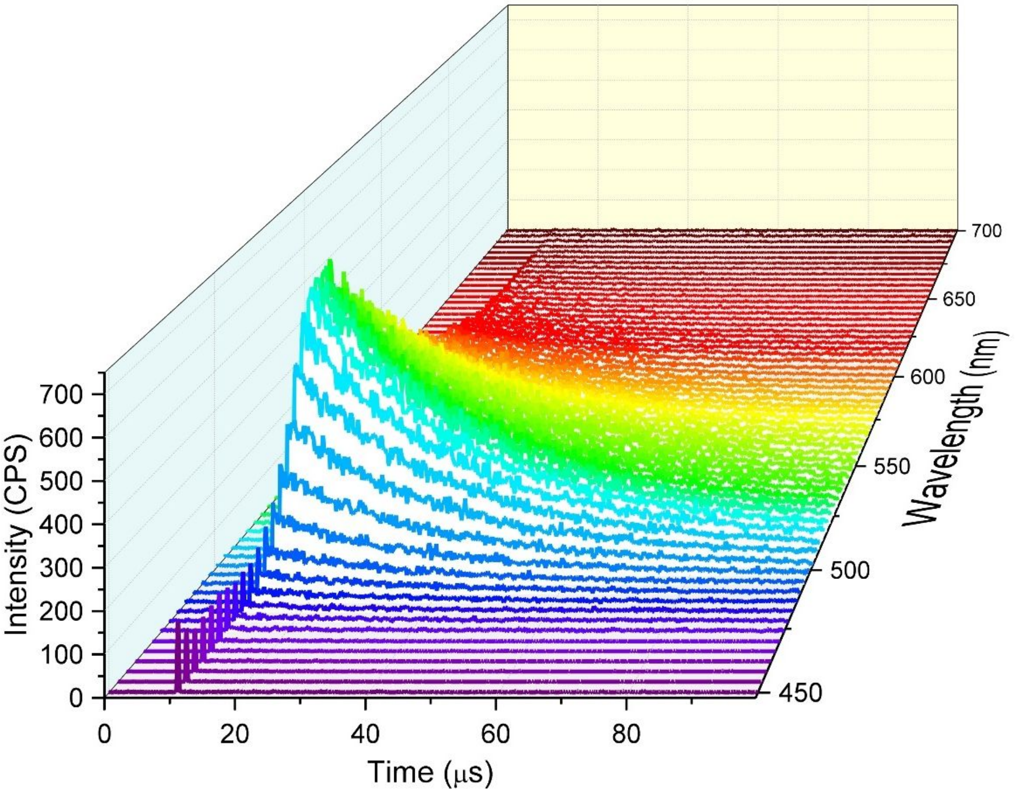

Figure S135. Time resolved emission spectra of **18** recorded in DCM excited as 337 nm.

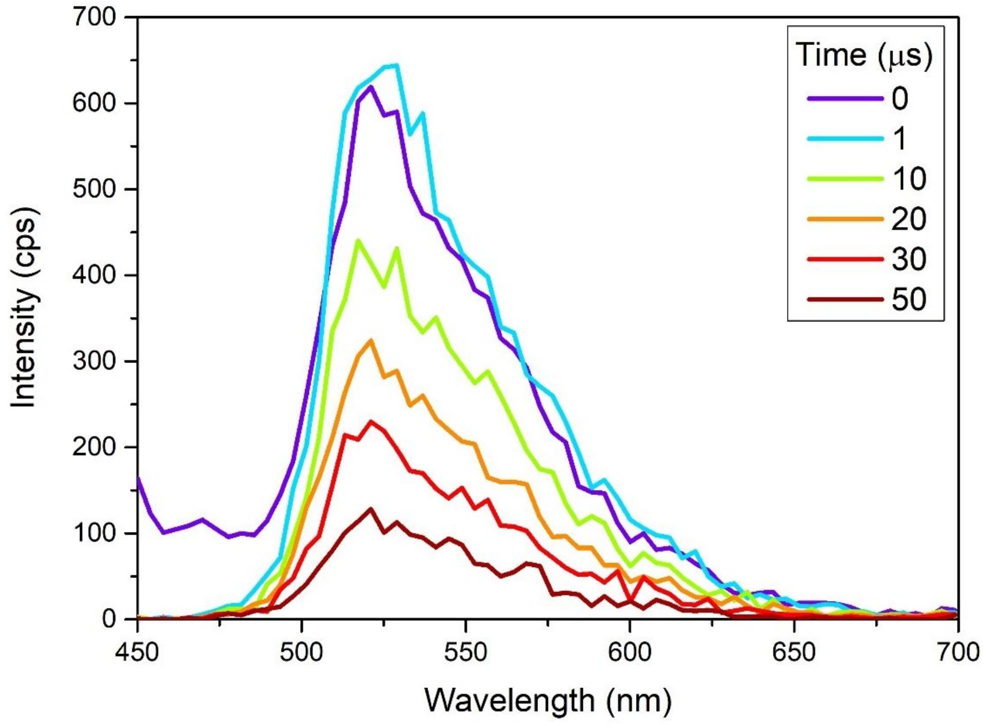

Figure S136. Emission spectra of **18** at specific time intervals after the initial emission, recorded in DCM excited at 337 nm.

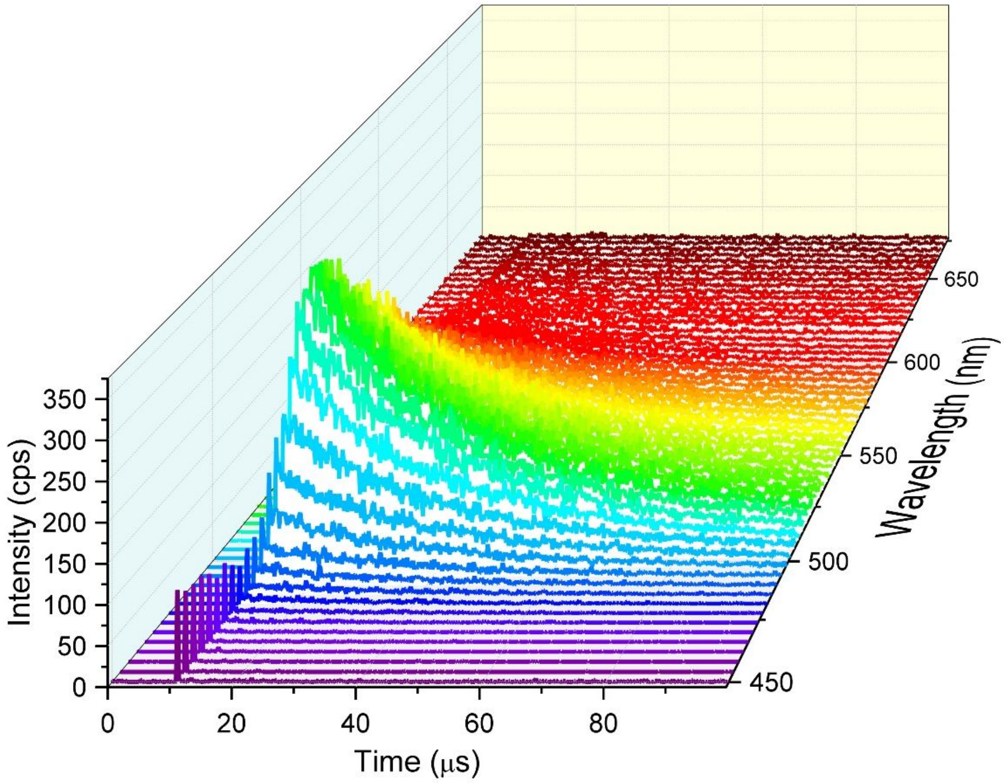

Figure S137. Time resolved emission spectra of **19** recorded in DCM excited as 337 nm.

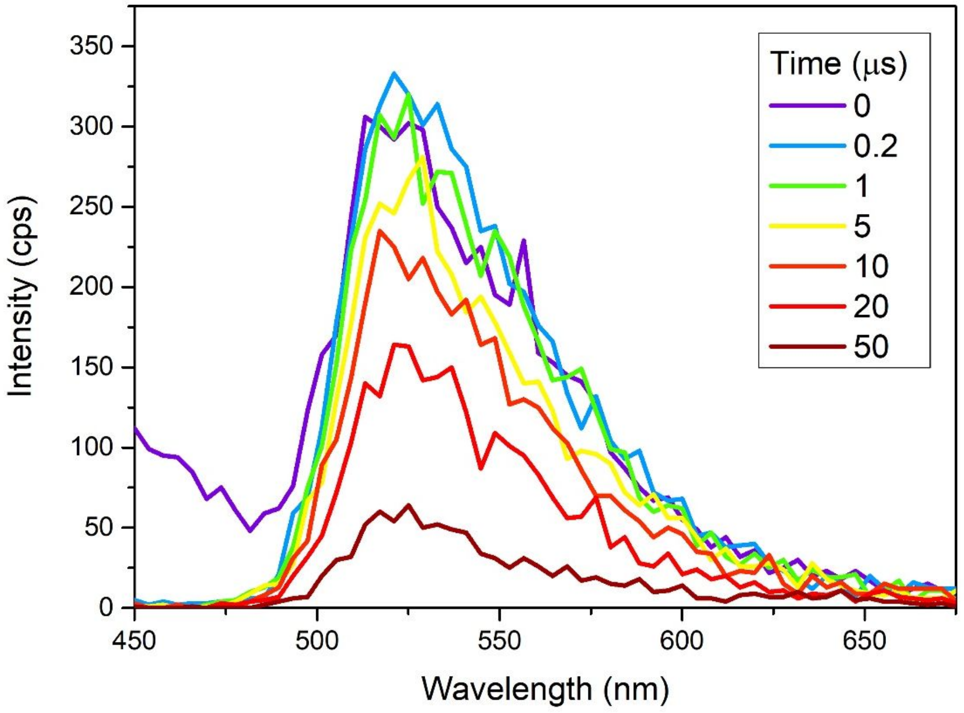

Figure S138. Emission spectra of **19** at specific time intervals after the initial emission, recorded in DCM excited at 337 nm.

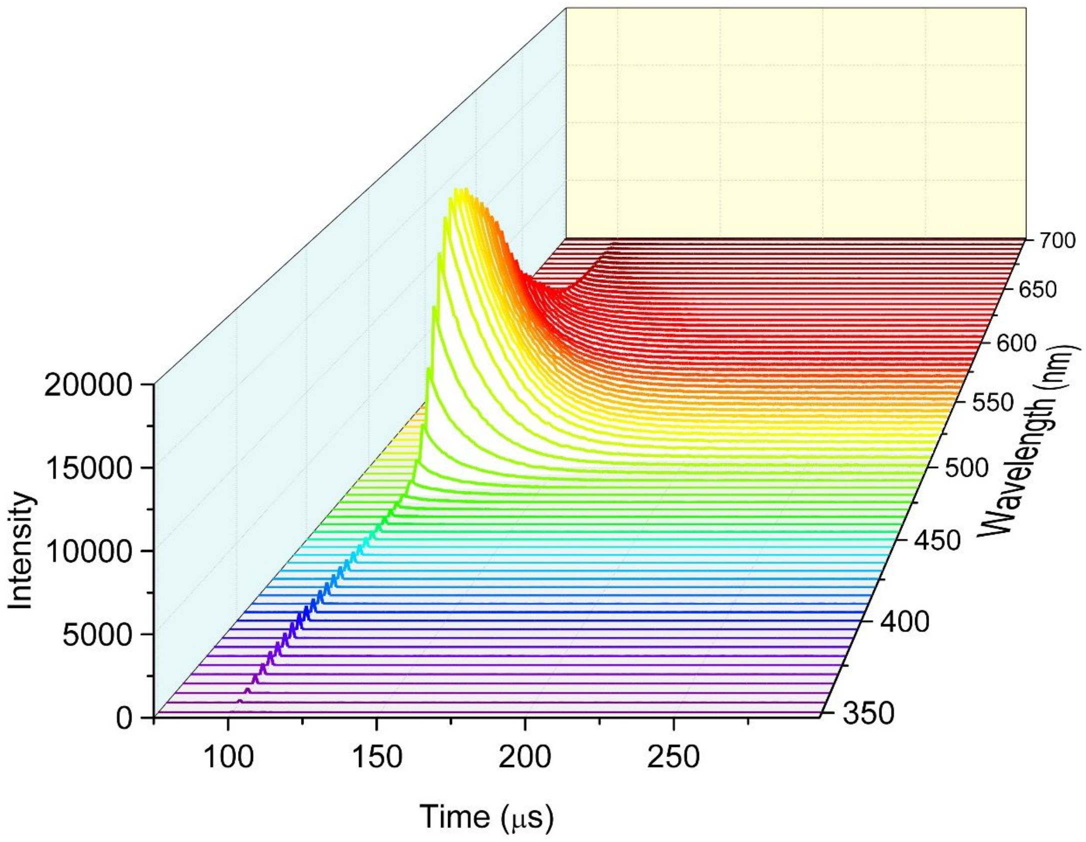

Figure S139. Time resolved emission spectra of **20** recorded in DCM excited as 337 nm.

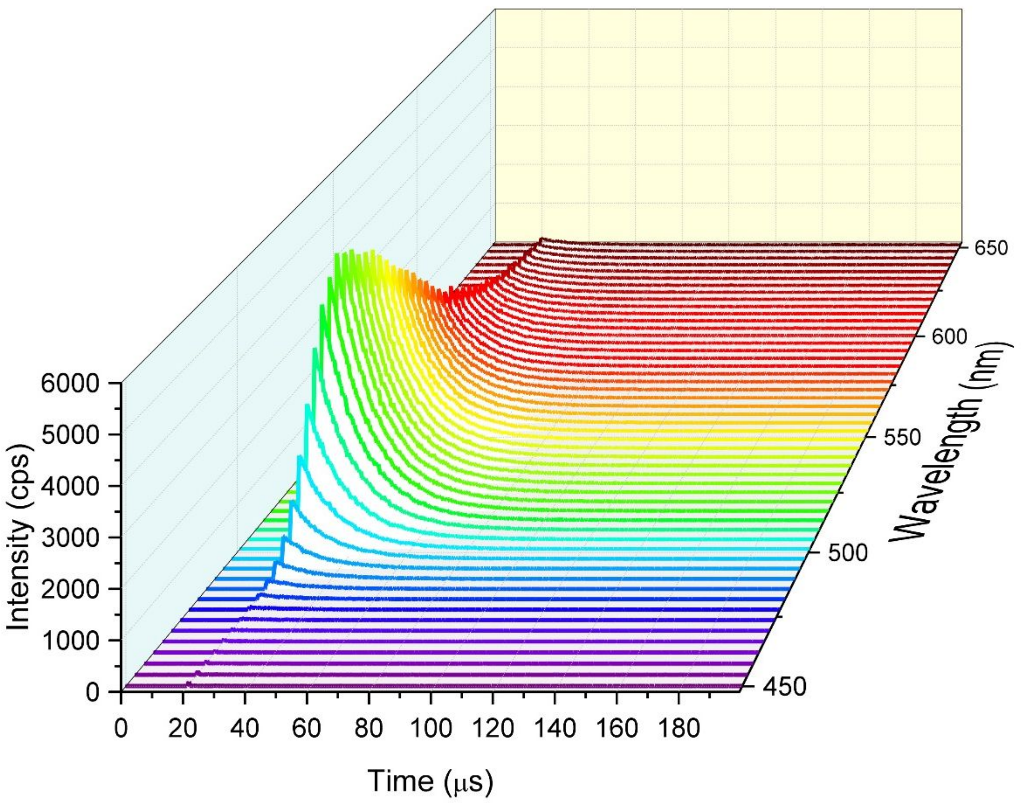

Figure S140. Time resolved emission spectra of **21** recorded in DCM excited as 337 nm.

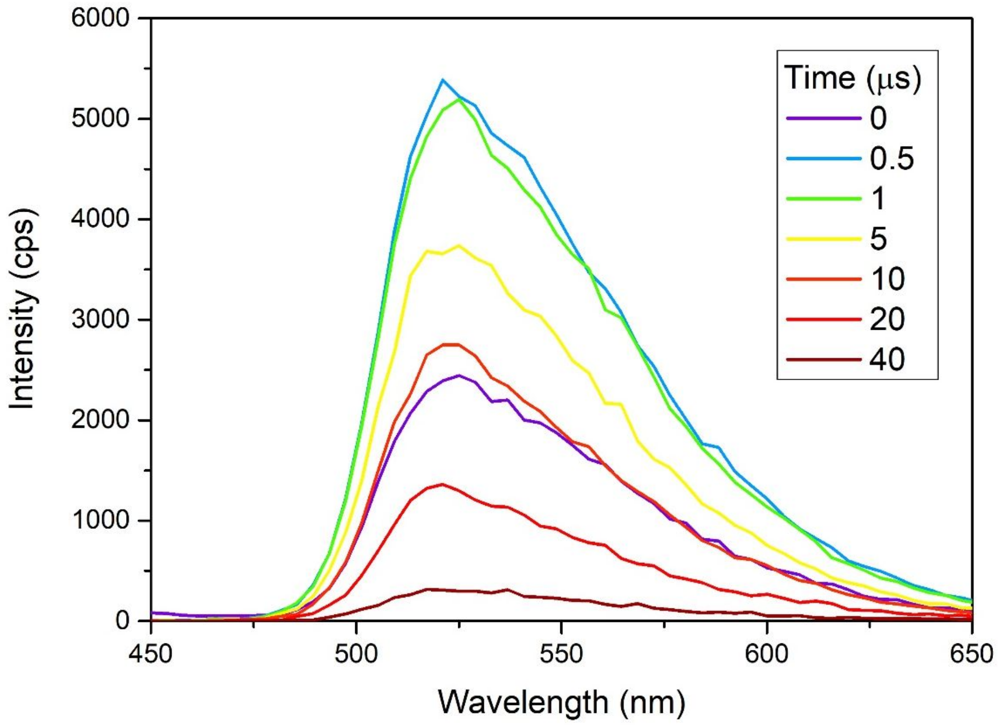

Figure S141. Emission spectra of **21** at specific time intervals after the initial emission, recorded in DCM excited at 337 nm.

References

1. M. J. Frisch; G. W. Trucks; H. B. Schlegel; G. E. Scuseria; M. A. Robb; J. R. Cheeseman; G. Scalmani; V. Barone; B. Mennucci; G. A. Petersson; H. Nakatsuji; M. Caricato; X. Li; H. P. Hratchian; A. F. Izmaylov; J. Bloino; G. Zheng; J. L. Sonnenberg; M. Hada; M. Ehara; K. Toyota; R. Fukuda; J. Hasegawa; M. Ishida; T. Nakajima; Y. Honda; O. Kitao; H. Nakai; T. Vreven; J. A. Montgomery, J., J. E. Peralta, F. Ogliaro, M. Bearpark, J. J. Heyd, E. Brothers, K. N. Kudin, V. N. Staroverov, R. Kobayashi, J. Normand, K. Raghavachari, A. Rendell, J. C. Burant, S. S. Iyengar, J. Tomasi, M. Cossi, N. Rega, J. M. Millam, M. Klene, J. E. Knox, J. B. Cross, V. Bakken, C. Adamo, J. Jaramillo, R. Gomperts, R. E. Stratmann, O. Yazyev, A. J. Austin, R. Cammi, C. Pomelli, J. W. Ochterski, R. L. Martin, K. Morokuma, V. G. Zakrzewski, G. A. Voth, P. Salvador, J. J. Dannenberg, S. Dapprich, A. D. Daniels, Ö. Farkas, J. B. Foresman, J. V. Ortiz, J. Cioslowski, and D. J. Fox. *Gaussian 09*, A.1; Gaussian, Inc: Wallingford CT, 2009.

2. Dennington, R.; Keith, T.; Millam, J. *GaussView*, Version 5; Semichem Inc.: Shawnee Mission KS, 2009.

3. O'Boyle, N. M.; Tenderholt, A. L.; Langner, K. M. cclib: A library for package-independent computational chemistry algorithms. *Journal of Computational Chemistry* **2008,** 29 (5), 839-845 DOI: 10.1002/jcc.20823.

4. Neese, F. The ORCA program system. *WIREs Computational Molecular Science* **2012,** 2 (1), 73-78 DOI: https://doi.org/10.1002/wcms.81.

5. Hanwell, M. D.; Curtis, D. E.; Lonie, D. C.; Vandermeersch, T.; Zurek, E.; Hutchison, G. R. Avogadro: an advanced semantic chemical editor, visualization, and analysis platform. *Journal of Cheminformatics* **2012,** 4 (1), 17 DOI: 10.1186/1758-2946-4-17.

6. Davidson, R.; Hsu, Y.-T.; Batchelor, T.; Yufit, D.; Beeby, A. The use of organolithium reagents for the synthesis of 4-aryl-2-phenylpyridines and their corresponding iridium(III) complexes. *Dalton Trans.* **2016,** 45 (28), 11496-11507 DOI: 10.1039/C6DT01461E.

7. Melhuish, W. H. Quantum effiencies of fluorescence of organic substances: effect of solvent and concentration of the fluorescent solute. *J. Phys. Chem.* **1961,** 65 (2), 229-235 DOI: 10.1021/j100820a009.

8. Karstens, T.; Kobs, K. Rhodamine B and rhodamine 101 as reference substances for fluorescence quantum yield measurements. *The Journal of Physical Chemistry* **1980,** 84 (14), 1871-1872 DOI: 10.1021/j100451a030.
